# Brain network dynamics codify heterogeneity in seizure evolution

**DOI:** 10.1093/braincomms/fcac234

**Published:** 2022-09-16

**Authors:** Nuttida Rungratsameetaweemana, Claudia Lainscsek, Sydney S Cash, Javier O Garcia, Terrence J Sejnowski, Kanika Bansal

**Affiliations:** Humans in Complex Systems Division, US DEVCOM Army Research Laboratory, Aberdeen Proving Ground, MD 21005, USA; Computational Neurobiology Laboratory, The Salk Institute for Biological Studies, La Jolla, CA 92037, USA; Neuroscience Center for Research and Innovation, Learning Institute, King Mongkut’s University of Technology Thonburi, Bangkok 10140, Thailand; Computational Neurobiology Laboratory, The Salk Institute for Biological Studies, La Jolla, CA 92037, USA; Institute for Neural Computation, University of California San Diego, La Jolla, CA 92093, USA; Department of Neurology, Massachusetts General Hospital and Harvard Medical School, MA 02114, USA; Humans in Complex Systems Division, US DEVCOM Army Research Laboratory, Aberdeen Proving Ground, MD 21005, USA; Computational Neurobiology Laboratory, The Salk Institute for Biological Studies, La Jolla, CA 92037, USA; Institute for Neural Computation, University of California San Diego, La Jolla, CA 92093, USA; Division of Biological Sciences, University of California San Diego, CA 92093, USA; Humans in Complex Systems Division, US DEVCOM Army Research Laboratory, Aberdeen Proving Ground, MD 21005, USA; Department of Biomedical Engineering, Columbia University, NY 10027, USA

**Keywords:** epilepsy, functional connectivity, seizure propagation, brain networks

## Abstract

Dynamic functional brain connectivity facilitates adaptive cognition and behaviour. Abnormal alterations within such connectivity could result in disrupted functions observed across various neurological conditions. As one of the most common neurological disorders, epilepsy is defined by the seemingly random occurrence of spontaneous seizures. A central but unresolved question concerns the mechanisms by which extraordinarily diverse propagation dynamics of seizures emerge. Here, we applied a graph-theoretical approach to assess dynamic reconfigurations in the functional brain connectivity before, during and after seizures that display heterogeneous propagation patterns despite sharing similar cortical onsets. We computed time-varying functional brain connectivity networks from human intracranial recordings of 67 seizures (across 14 patients) that had a focal origin—49 of these focal seizures remained focal and 18 underwent a bilateral spread (focal to bilateral tonic-clonic seizures). We utilized functional connectivity networks estimated from interictal periods across patients as control. Our results characterize network features that quantify the underlying functional dynamics associated with the observed heterogeneity of seizure propagation across these two types of focal seizures. Decoding these network features demonstrate that bilateral propagation of seizure activity is an outcome of the imbalance of global integration and segregation in the brain prior to seizure onset. We show that there exist intrinsic network signatures preceding seizure onset that are associated with the extent to which an impending seizure will propagate throughout the brain (i.e. staying within one hemisphere versus spreading transcallosally). Additionally, these features characterize an increase in segregation and a decrease in excitability within the brain network (i.e. high modularity and low spectral radius). Importantly, seizure-type-specific differences in these features emerge several minutes prior to seizure onset, suggesting the potential utility of such measures in intervention strategies. Finally, our results reveal network characteristics *after* the onset that are unique to the propagation mechanisms of two most common focal seizure subtypes, indicative of distinct reconfiguration processes that may assist termination of each seizure type. Together, our findings provide insights into the relationship between the temporal evolution of seizure activity and the underlying functional connectivity dynamics. These results offer exciting avenues where graph-theoretical measures could potentially guide personalized clinical interventions for epilepsy and other neurological disorders in which extensive heterogeneity is observed across subtypes as well as across and within individual patients.

## Teaser

Using a graph-theoretical technique, Rungratsameetaweemana *et al.* demonstrate that propagation mechanisms of seizure activity can be decoded from the network properties of functional brain connectivity as inferred from electrocorticography recordings.

## Graphical Abstract

**Graphical abstract:**
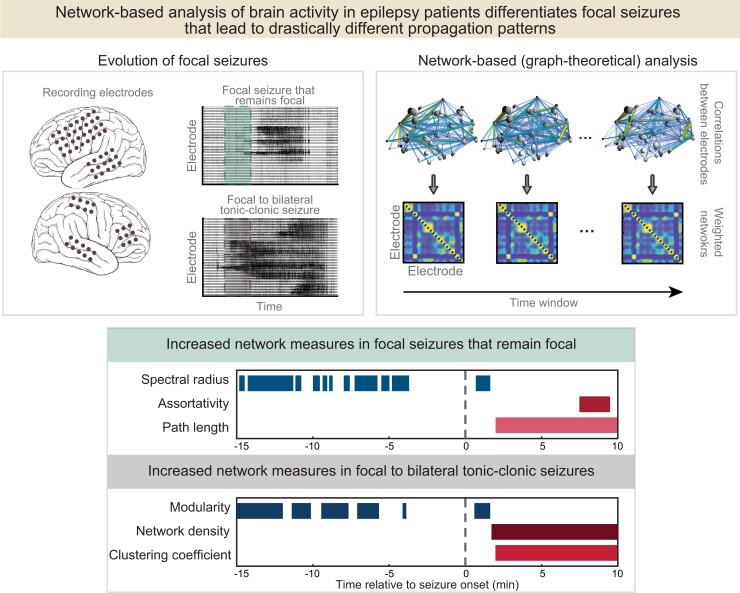

## Introduction

**Figure 1:**
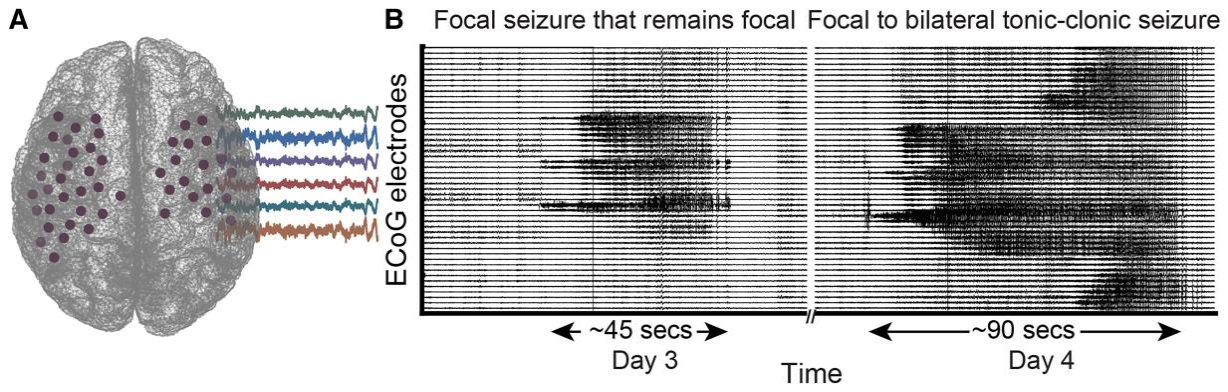
**Emergence of distinct seizure propagation patterns in a single patient.** (**A**) During a clinical monitoring procedure to identify a seizure onset zone of patients with medication-refractory (drug-resistant) epilepsy, intracranial recording electrodes are implanted. (**B**) Intracranial activity during two sample seizures recorded from a single patient, which exhibit distinct propagation dynamics. On the left, the seizure activity originates from a few electrodes and persists in the localized area within a single hemisphere (i.e. focal seizure that remains focal). On the right, the seizure activity originates from a few electrodes but diffuses bilaterally to involve electrodes in both hemispheres. This type of seizure is known as focal to bilateral tonic-clonic seizure or focal seizure with bilateral spread. Despite their similarly focal origin, these seizure types induce drastically differential clinical manifestations such that focal to bilateral tonic-clonic seizures are associated with more severe cognitive and behavioural deficits. We hypothesize that such heterogeneity in seizure dynamics emerges from distinct and measurable temporal alterations in the functional brain connectivity networks.

As one of the most common neurological disorders with roughly 50 million cases world-wide, epilepsy is characterized by its emerging spontaneous seizure activity.^1,2^ Critically, one-third of the patients do not respond to medications and rely on alternative interventions (e.g. surgical and neuromodulatory).^3-5^ However, seizures are remarkably diverse, and tailoring effective treatment strategies remain a substantial challenge at least partially due to the temporal spontaneity and lack of objective frameworks that could characterize the onset and propagation patterns of an impending seizure.^6,7^ Traditionally, the variability across seizures has been categorized based on the onset regions: *focal seizures* originate from a localized region within one hemisphere while *generalized seizures* begin simultaneously from both hemispheres. A variety of computational techniques from network science and dynamical systems have been employed to better localize the onset regions and thus improve the precision with which focal and generalized seizures can be identified.^8-10^ However, localizing onset regions does not fully capture the breadth of dynamics and inherent diversity associated with seizure subtypes. Adding to this complexity, once generated, a focal seizure can remain localized within the same hemisphere (i.e. *focal seizures that remain focal*) or propagate to the other hemisphere (i.e. *focal to bilateral tonic-clonic seizures* or *focal seizures with bilateral spread*).^11-13^ Notably, a single patient may experience both of these focal seizure subtypes (Fig. 1), where the seizures that spread bilaterally generally lead to more severe behavioural and cognitive deficits that could require several minutes to hours for patients to recover. However, the distinct propagation dynamics exhibited by different seizure types are largely ignored by traditional intervention approaches and it remains unknown if the mechanisms underlying the bilateral spread of focal seizure activity differ from those associated with focal seizures that remain localized. Critically, the field currently lacks an objective analytical framework that can be utilized to investigate, understand and predict the heterogeneity associated with propagation dynamics of seizure activity. Objective biomarkers that can identify whether or not an impending seizure will spread bilaterally could allow clinicians to implement an appropriate intervention strategy, thereby minimizing the severity of adverse cognitive impacts.

For the past several years, epilepsy has more frequently been viewed as a manifestation of network reorganizations and not just hypersynchrony, and a graph-theoretical framework in which brain dynamics is visualized as temporally evolving graphs or networks composed of nodes and edges that represent brain regions and their pairwise associations, respectively,^14-16^ has been useful in quantifying and understanding these network reorganizations.^17-19^ Here, we demonstrate that the long-standing challenges associated with the heterogeneity observed across subtypes of epileptic seizures can also be addressed through the lens of graph theory. This approach relies on a complex systems view of the brain where a single brain region interacts with many others and collectively, these interactions give rise to a wide variety of functional connectivity patterns underlying our adaptive cognitive abilities and subsequent behaviour. Investigating the temporal evolution of such connectivity patterns, within a graph theoretical framework, has provided better insight into the emergence of neural properties such as specialization and efficiency of information processing, learning and aging^18,20-24^ and has applications in clinical neuroscience for the potential to establish biomarkers of disease onset and progression.

Our study is built upon the idea that the manner in which functional brain connectivity networks reconfigure over time may carry information concerning the emergent seizure dynamics and cognitive behaviours that are unique to the underlying neurological processes. Consequently, we probed the time-varying changes within functional connectivity networks derived from multiple hours of electrocorticogram (ECoG) recordings across 14 patients as they experienced focal seizures that remain focal or focal to bilateral tonic-clonic seizures. With this analytical framework, we aimed to gain insight into how the unique heterogenous dynamic properties associated with different seizure types develop and unfold in the brain. Our results uncover key network features that characterize the different neural dynamics associated with each type of focal seizures. Further, our findings demonstrate that the emergence of different propagation patterns is an outcome of unique network-level changes and distinct mechanisms that regulate the extent of synchronization within the brain.

## Materials and methods

### Patient information and data acquisition

The seizures analysed in this study were recorded from 14 patients with medication-refractory epilepsy (Table 1) who underwent a clinical monitoring procedure to locate their seizure onset zone. Clinical electrode implantation, positioning, duration of recordings and medication schedules were based solely on clinical need as determined by an independent team of clinicians. As indicated in Table 1, seizures analysed in this study were of two types: (i) focal seizures that remain focal and (ii) focal to bilateral tonic-clonic seizures (or focal seizures with bilateral spread). Patients were implanted with intracranial subdural grids, strips and depth electrodes for several days in a specialized hospital setting and continuous multichannel ECoG data were recorded at a sampling rate of 500 Hz.

**Table 1:** Patient profiles

Patient	Sex	Age at onset/	Aetiology	Seizure type (#)	Seizure onset	Electrode type (#)	Resection areas	Outcome
		surgery			zone			
1	F	15/46	Dysplasia	Focal to bilateral (5)	Anterior	Depths/grids (113)	Right	I
					temporal		Anterior temporal	
2	F	42/55	n.a.	Focal to bilateral (3)	Temporal	Depths (56)	None	I
3	F	17/45	n.a.	Focal (1)	Temporal	Depths (40)	None	n.a.
				Focal to bilateral (2)				
4	M	8/23	n.a.	Focal (10)	Frontal	Depths (80)	None	III
5	M	14/35	n.a.	Focal (9);	Temporal	Depths (80)	Right	n.a
				Focal to bilateral (2)			Anterior temporal	
6	F	12/32	n.a.	Focal (15)	Temporal	Depths (112)	Right	II
							Anterior temporal	
7	F	7/23	n.a.	Focal (6)	Frontal	Depths (80)	Left frontal	IV
8	F	10/27	n.a.	Focal (1)	Unknown	Depths (80)	Left frontal	IV
9	F	8/19	MTS	Focal (1)	Anterior	Grids (60)	Left	III
					temporal		Anterior temporal	
10	F	14/31	n.a.	Focal to bilateral (2)	Temporal	Depths (48)	Right	I
							Anterior temporal	
11	F	1/21	Stroke	Focal (2)	Temporal	Depths (118)	Left temporal	IV
12	F	9/42	n.a.	Focal (2)	Frontal	Depths (76)	None	II
13	M	39/47	n.a.	Focal to bilateral (3)	Posterior	Depths (48)	Right temporal	I
					Temporal			
14	F	50/59	n.a.	Focal (2)	Posterior	Depths (80)	Left temporal	I
				Focal to bilateral (1)	Temporal			

Clinical characteristics of the patients. For each patient, we report sex, age at first reported seizures onset, as well as age at the monitoring phase and surgery. We also report the seizure aetiology, which was clinically determined through medical history, imaging and long-term invasive monitoring. Additionally, we indicate the number of observed seizures associated with the two different types of seizures which originated from one hemisphere: focal seizures that remained localized within the same hemisphere (focal seizure; focal) and focal seizures that propagate bilaterally to both hemispheres (focal to bilateral tonic-clonic seizure; focal to bilateral). Surgical outcome (outcome) was based on Engel score: seizure freedom to no improvement (I–V), and no follow-up (NF). M, male; F, female; MTS, mesial temporal sclerosis; n.a., not applicable.

Only seizures with an obvious ictal onset were selected for analysis. Experienced epileptologists, blind to this study, identified the seizure onset regions, seizure types and onset time through inspection of the ECoG recordings, referral to the clinical report and clinical manifestations recorded on video. A total of 67 seizures (49 focal seizures that remain focal and 18 focal to bilateral tonic-clonic seizures) were analysed. We note that multiple seizures from the same patients were treated as independent (see similar methods in Martinet *et al*.^4^).

### Ethics statement

All patients were enrolled after informed consent was obtained and approval was granted by local Institutional Review Boards at Massachusetts General Hospital according to National Institutes of Health guidelines.

### Data preprocessing

For each of the seizures, we considered ECoG data of the duration of 15 min before and 10 min after the seizure onset. Each of these 25 min data segments contained only one seizure. For comparison with relatively ‘seizure-free’ (interictal) activity, we extracted an equal number of interictal activity epochs with the same duration (i.e. 67 interictal epochs). Interictal epochs were selected from ECoG recordings at least an hour away from the onset and offset of any seizure. Specifically, for each of the 67 seizures (49 focal and 18 focal to bilateral tonic-clonic) from which we extracted a 25 min segment (from 15 min before to 10 min after the onset), we also epoched a 25 min segment of data that were at least 60 min away from the seizure onset and offset. The data were band-pass filtered between 1 and 70 Hz, and notch filtered at 60 Hz to exclude potential powerline interference.

Prewhitening was then performed using a first-order autoregressive model to account for slow dynamics and correct for autocorrelation in the time series signals.^25-28^ A common reference was used for data analysis and the reference electrode in each case was located far from the area of recording making the introduction of spurious correlation or elimination of actual correlation between cortical regions unlikely.^29^

### Functional connectivity networks

**Figure 2:**
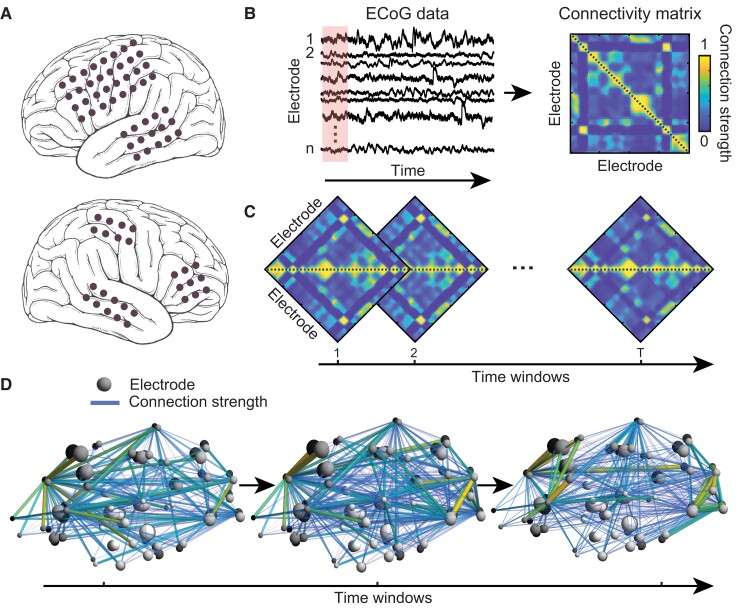
**Schematic of graph-theoretical analysis of functional brain dynamics.** (**A**) Locations of implanted intracranial electrodes of a sample patient. (**B**) We use electrocorticography (ECoG) time-series data from all intracranial electrodes from each patient recorded during a clinical monitoring procedure to locate the seizure onset zone. We estimate the instantaneous functional connectivity of the underlying brain network by computing pairwise correlations of ECoG data across electrodes in a sliding-window manner. The magnitudes of these correlations (restricted between 0 and 1) reflect the strength of connections between each pair of electrodes and are represented by a weighted adjacency or connectivity matrix (see Materials and methods). (**C**) To investigate time-varying changes in the functional brain connectivity during temporal evolution of each seizure type, we compute a series of connectivity matrices over time and use these as bases to construct functional connectivity networks. (**D**) A schematic of sample constructed networks, consisting of nodes (electrodes) and edges (connection strength). To quantify alterations within these complex networks over time, we evaluate changes of a series of graph-theoretical attributes which describe globally and locally defined properties of the constructed networks.

To evaluate functional connectivity representations associated with the temporal evolution of seizures, we employed a time-evolving network analysis. Here, we constructed time-varying functional connectivity networks of focal seizures and interictal activity where the inter-electrode relationships were represented by network edges and the electrodes themselves were represented by nodes in the corresponding networks of seizures and interictal activity (Fig. 2A–D).

Specifically, we computed symmetric functional connectivity *A_ij_* between two regions of the brain *i* and *j* as an averaged correlation of the neural signals recorded by the intracranial electrode contacts of those regions. To extract (at least approximately) the stationary aspects of ECoG data, we divided each of the 25 min ECoG data segments into consecutive 1 s windows, where each window overlapped the previous window by 0.5 s.^30,31^ The correlation was calculated within each of these 1 s segments. To account for noise, we applied a temporal smoothing to these correlation values by averaging consecutive 30 s windows such that a total of 98 correlation values representing the functional connectivity of 98 temporal windows were generated from each 25 min ECoG data segment. Note that different temporal smoothing parameters can be used without affecting the overall patterns of results, although a value too large may reduce the temporal precision of the observations (Supplementary Fig. 1).

All correlation values were bounded between −1 and +1. Negative correlation values were then set to zero following traditional modelling choices adopted in previous studies^3,32^ examining simulation of the epileptogenic effect. Temporal evolution of these correlations or connectivity matrices reflects the time-varying dynamics of the functional brain networks as recorded through the ECoG measurements.

### Graph-theoretical network analysis

For each of the seizures (49 focal and 18 focal to bilateral tonic-clonic) and interictal activity (67 epochs), we constructed a series of weighted, symmetric (undirected) connectivity matrices *A* representing functional correlations across all recording electrodes. From these network matrices, we computed a series of graph network measures (described below) as a function of seizure types to quantify changes in network dynamics associated with evolution of focal seizures with constrained (focal seizure that remain focal) and unconstrained propagation mechanisms (focal to bilateral tonic-clonic seizures). We focused our analyses on complex network measures that are sensitive not only to the overall extent of connectivity within a network (i.e. density; see below), but also to the relative distribution of connectivity weights within the network which reconfigure to enable various neurophysiological processes. Investigating and quantifying these network reconfigurations as a function of seizure types allows for a better understanding of the underlying processes that govern the bilateral spread of a focal seizure. We used various Brain Connectivity Toolbox functions implemented in MATLAB (R2020; MathWorks) for our computation of these network features unless noted otherwise.

### Network density

Network density describes the extent of connectedness in a network. Seizures have been shown to alter the synchronization, and therefore, overall connectivity and density of the functional network. In a binary network, where the elements of connectivity matrix are either 1, for a link between the nodes, or 0, for absence of a link, the density is calculated as the ratio of actual and possible connections. A density of 1 describes a fully connected network and a density of 0 describes no connectivity. In a weighted network, if many weak connections are binarized, the density of the network can become misleadingly high. Therefore, we calculated weighted network density as the ratio of total edge weights and number of possible edges. For a functional connectivity, matrix *A* representing an undirected network with *N* nodes, the total number of possible edges is *N*(*N* *−* 1)/2 and the total edge weight is the sum of the elements in the upper triangle of the connectivity matrix. Similar methods have previously been used to evaluate and successfully capture the degree of connectedness in a range of real-world brain networks across species (i.e. humans, macaques, cats and *Caenorhabditis elegans*).^33^

Networks with different density (and/or size) can have different network features.^34^ Therefore, we compared network density across individual patients during interictal period to ensure that our findings are truly driven by the underlying changes in the functional connectivity as a function of seizure types and are free of spurious results due to different network sizes and densities across patients (Supplementary Fig. 2).

### Clustering coefficient

Clustering coefficient (CC) is a measure of local cliquishness of a network and has been used to describe the segregation of information in brain networks. While a high CC has been reported to characterize the ictal phase for temporal lobe epilepsy patients,^35^ the role of CC in differentiating constrained and unconstrained seizure dynamics is not well understood. The CC is calculated as the ratio between the number of triangles present around a node and the maximum number of triangles that could possibly be formed around that node.^36,37^ For a given Node *X* and any other two Nodes *Y* and *Z* within the network, a triangle around *X* represents a scenario where *X*, *Y* and *Z*, all have a connectivity value of one with one another. We used the Brain Connectivity Toolbox function *clustering_coef_wu* for the calculation of CC.

### Characteristic path length

Characteristic path length (PL) describes the averaged shortest PL between all pairs of nodes in a network and has been associated with the brain’s ability to integrate information. The shortest PL between a pair of network nodes represents the shortest route between them through a combination of network edges. We calculated characteristic PL using the Brain Connectivity Toolbox function *charpath.*^38^ Both CC and characteristic PL are used to assess if a network is more ‘regular’ (only nearest neighbour connections) or ‘random’ in its connectivity. Regular networks have high clustering and long PL, whereas random networks have low clustering and short PL. Typically, brain networks exhibit a small-world architecture which is characterized by a combination of dense local clustering of connections between neighbouring nodes (like regular networks) and a short PL between distant node pairs due to the existence of relatively few long-range connections (like random networks).^14,15,18,39^

### Assortativity

Assortativity measures the propensity of nodes to connect to others with similar degree and is calculated as a correlation between the degrees of all the nodes.^40^ A positive assortativity value indicates that nodes tend to link to other nodes with similar degree, whereas a negative value indicates connected nodes with dissimilar degree. Networks with high assortativity tend to make a highly connected core of network hubs. Functional brain networks have been shown to display such an architecture with highly connected hub regions or core surrounded by low-connectivity peripheral nodes.^41,42^ Assortativity quantifies network robustness as a removal or failure of a single high-degree node would induce greater impact on communication efficiency of a network with low assortativity than on a network with high assortativity. We calculated assortativity using the Brain Connectivity Toolbox function *assortativity_wei.*^38^

### Modularity

Modularity describes the extent to which a graph can be divided into clearly separated communities (i.e. subgraphs or modules). Each module contains several interconnected nodes, and there are relatively few connections between nodes of different modules. In the context of brain networks, modularity has been used to describe and quantify efficient integration and segregation of information across distributed sets of brain regions as a function of cognitive task demands.^43,44^ Mathematically, the modularity metric (*Q*) represents the number of edges falling within modules minus the expected number in an equivalent network with edges placed at random. It is estimated as follows:

$$Q=\frac{1}{2m}\underset{ij}{\sum }\;\left(A_{ij}-\gamma \frac{k_{i}k_{j}}{2m}\right)\delta (c_{i},c_{j}),$$

where *A*_*ij*_ represents the connectivity between Nodes *i* and *j*, *k_i_* represents the degree of the Node *i*, *m* represents the total number of edges in the network, *γ* represents a resolution parameter and *δ*(*c*_*i*_, *c*_*j*_) equals 1 if Nodes *i* and *j* are in the same community, and equals 0 otherwise.^45^ Here, to detect modules and obtain a value of modularity for functional brain networks, we used the spectral approach described by Newman and implemented it through Brain Connectivity Toolbox function *modularity_und*.^45,46^ The resolution parameter *γ* was chosen to be 1.

### Spectral radius

Spectral radius is a global measure of network structure that is related to the spread of activity in a network.^23,47,48^ Computed as the largest eigenvalue of the connectivity matrix (*A*), spectral radius reflects the critical coupling strength required to synchronize the system.^49^ As such, spectral radius represents the principal component of the system and contains information about structural characteristics as well as dynamical behaviour and stability of the underlying network.^50-52^ In the network based models of brain dynamics, spectral radius has been associated with the ease with which the system can be transitioned into an excited state.^23^

### Synchronizability

Synchronizability relates to the viability of synchronized dynamics within a network. Particularly in the context of epilepsy, relatively larger value of synchronizability has been associated with greater ease for neural populations to synchronize their dynamics.^24^ Synchronizability is sensitive not only to the overall connectivity or density of a network but also to the relative distribution of edge weights across network nodes.^53^ Here, we leveraged the measure of synchronizability to assess network reconfigurations that may happen around the onset of different seizure types. Synchronizability (*S*) is calculated as the ratio of the second smallest and the largest eigenvalue of the Laplacian matrix (*L*), which is computed as the difference between the diagonal matrix of node strength (total degree) and the adjacency matrix such that *L* = *D*–*A*. Thus, synchronizability estimates the spread of the eigenvalues of the network Laplacian and is computed as $S=\lambda_{2}^{L}/\lambda_{\max}^{L}$, where $\lambda_{2}^{L}$ and $\lambda_{\max}^{L}$ denote the second smallest and the largest eigenvalue of *L*, respectively.

### Statistical analysis

Given the diverse nature of seizure activity and the patient-specific procedures by which the placement and number of ECoG electrodes is determined, the recorded signals associated with individual seizures are inherently variable. It is well-documented that even within a single patient, different occurrences of seizure activity can vary in their durations, phases and latencies of spread.^8,11,54^ To account for such variability across individual seizures and to minimize statistical biases that could arise, for each network feature we used a bootstrapping procedure^55^ to generate estimates of the mean and 95% confidence intervals (CIs), and made comparisons across seizure types in a time resolved manner. To perform these comparisons, we employed a rigorous statistical analysis as described in the following.

First, for each network measure and for each time point, we resampled with replacement at the level of individual seizures 10 000 times. Through these bootstrapping iterations, we generated an empirical estimate of the variability associated with each network measure of each seizure type over time (e.g. a bootstrapped distribution of the CC associated with focal seizures that remain focal, a bootstrapped distribution of the CC associated with focal to bilateral tonic-clonic seizures, and a bootstrapped distribution of the CC associated with interictal activity). Next, to compare a particular network feature between conditions (e.g. focal seizures that remain focal versus focal to bilateral tonic-clonic seizures), we calculated the difference at each time point between these bootstrapped data associated with each condition to obtain distributions of ‘differences’ with respective means and CIs specific to each time point for each comparison (e.g. difference between the CC of focal seizures that remain focal and that of focal to bilateral tonic-clonic seizures; Supplementary Figs 4 and 5). These distributions of ‘differences’ were used to assess the *P*-values and effect sizes, and to determine if a network feature significantly differentiates seizure types at a given time point.

To assess significance (though *P*-values), we evaluated the observed distribution of bootstrapped ‘differences’ against chance. To establish chance levels, we constructed null distributions non-parametrically for each complex network measure and for each time point by shuffling the condition labels 10 000 times, each time recomputing the difference between the seizure conditions on the shuffled data. Consequently, each null distribution centred at zero (e.g. the CC does not differ between focal seizures that stay focal and focal to bilateral tonic-clonic seizures; difference of zero). We compared the bootstrapped empirical and null distributions by conducting 2 one-tailed tests against zero [i.e. mean(difference in CCs) < 0 and mean(difference in CCs) > 0] and then doubling the smaller *P*-value. As the CIs for each comparison at each time point was achieved based on 10 000 bootstrapping iterations, the resolution of *P*-values was constrained to a lower limit of *P* ≤ 0.0001. A *P*-value <0.05 was deemed significant.

For each comparison, we estimated effect sizes in two different ways: (i) by calculating the mean of the bootstrapped ‘difference’ distributions [e.g. mean (the CC of focal seizures that remain focal *minus* the CC of focal to bilateral tonic-clonic seizures); Supplementary Figs 4 and 5]; and (ii) by directly computing Cohen’s *d*^56,57^ for the bootstrapped distributions of each network feature (e.g. the CC of focal seizures that remain focal versus the CC of focal to bilateral tonic-clonic seizures). For each of the significantly different statistical comparisons the smallest Cohen’s *d* (Cohen’s *d*_min_) is reported. For each network measure, this value indicates the effect size associated with the temporal window at which the difference between seizure types were statistically significant but smallest. For example, if the CC is lower in focal seizures that remain focal than in a focal to bilateral tonic-clonic seizures from time *t*_1_ to *t*_2_, the reported Cohen’s *d*_min_ signifies the smallest effect size within this [*t*_1_*t*_2_] period. The 95% CI associated with each Cohen’s *d*_min_ is also reported.

**Figure 3:**
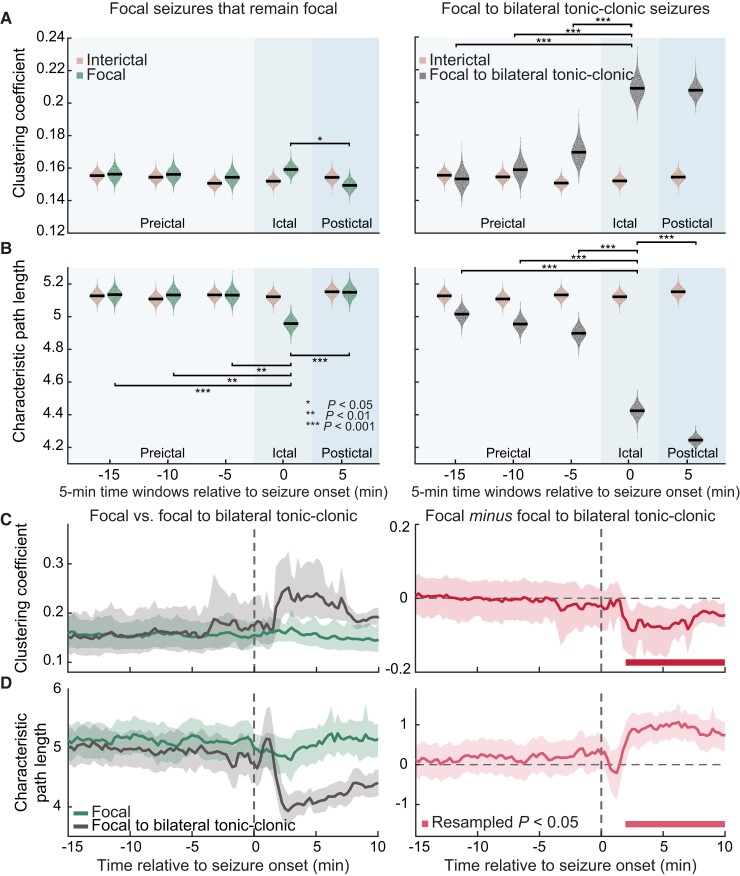
**Clustering coefficient and characteristic path length track diffusivity of seizure activity.** Focal to bilateral tonic-clonic seizures (*n* = 18) display simultaneous increase in the clustering coefficient (CC) and decrease in the characteristic path length (PL) than focal seizures that remain localized within one hemisphere (*n* = 49). (**A**) Averages of CC associated with each seizure type are plotted separately for preictal, ictal (during seizure) and postictal periods. CC of interictal (seizure-free) networks is also plotted as a baseline. (**B**) PL is plotted in the same manner. (**C**) CC of focal to bilateral tonic-clonic seizures is higher than that of focal seizures that remain focal, 2.25–10 min after seizure onset. (**D**) PL of focal to bilateral tonic-clonic seizures is lower than that of focal seizures that remain focal, 2–10 min after seizure onset. Statistical comparisons of network measures as a function of seizure types were computed through a bootstrapping procedure where the underlying data distribution of each network measure was resampled at the level of individual seizures to established 95% confidence intervals (CIs). For (**A**) and (**B**), significance of changes in the averages of CC and PL across time windows were evaluated by two-way analysis of variances (repeated measures) with a series of *post hoc t-*tests (two-tailed) for significant main effects. For (**C**) and (**D**), error bars indicate 95% CIs across individual seizures in each condition and solid bars show resampled *P* < 0.05.

In addition, we used two-way repeated-measures analysis of variances with within-subject factors for seizure type (three levels: focal seizures that remain focal, focal to bilateral tonic-clonic seizures and interictal activity) and time window (five levels: Preictal I, Preictal II, Preictal III, ictal and postictal) to evaluate the influence of these factors on CC and characteristic PL (Fig. 3A and B). In the case of significant main effects, follow-up two-tailed *t*-tests were performed.

### Data availability

All data that support the findings of this study are present in the main text and/or the Supplementary materials. These data are available on request from the corresponding author. The data are not publicly available as they contain information that could compromise privacy of the research participants.

## Results

### Increased clustering and shorter path length following bilateral spread of seizure activity

In the context of epilepsy, CC and characteristic PL are most commonly studied graph-theoretical features which are utilized to assess if and how seizure activity affects the small-world characteristics of the brain.^17,35,58^ Here, we did not observe any significant differences in the small-world characteristics of the functional connectivity networks associated with the constrained and unconstrained seizure propagation dynamics (Supplementary Fig. 3). We, therefore, independently evaluated CC and PL to investigate if each measure differed between the constrained and unconstrained propagation mechanisms associated with focal seizures that remain focal and focal seizures with bilateral spread, respectively. To accomplish this, we calculated the CC and the characteristic PL of each connectivity matrix (i.e. 98 matrices per each of the 25 min segments of seizure activity). First, to evaluate these results in the light of past studies, we computed averages of these values in a series of consecutive 5 min windows, separately for each seizure type. This resulted in three preictal, one ictal (during seizure) and one postictal windows (Fig. 3A and B). A similar analysis was applied to interictal data to estimate baseline values to which the seizure-related network measures could be compared.^38^

Our results revealed that there were significant main effects of seizure type and time window on CC and PL (*P*_min_ < 0.0001). Specifically, we found that both focal seizures that remain focal and focal to bilateral tonic-clonic seizures displayed lower PL during ictal periods when compared with preictal activity (Fig. 3A and B). *Post hoc* analyses demonstrated that the ictal activity associated with focal seizures that remain focal exhibited (i) higher CC when compared with postictal period (*P* = 0.01; Fig. 3A, left panel) and (ii) lower PL when compared with both preictal and postictal periods (preictal: *P* < 0.001, <0.01, <0.01; postictal: *P* < 0.001; Fig. 3B, left panel). Additionally, we observed similar changes for focal seizures with bilateral propagation where the ictal activity displayed (i) higher CC when compared with all the preictal periods (all *P* < 0.001; Fig. 3A, right panel) and (ii) shorter PL when compared with all the preictal periods (all *P* < 0.001; Fig. 3B, right panel). However, unlike the CC and PL associated with the postictal periods of focal seizures with constrained dynamics which returned to the preictal levels, the postictal PL of focal seizures with bilateral spread exhibited a continued decrease (*P*_min_ < 0.0001, Fig. 3B, right panel). These results supported the more unconstrained diffusivity associated with focal to bilateral tonic-clonic seizures. These observed differences regarding the manner in which the CC and PL changed in the networks of focal seizures with constrained and unconstrained dynamics were our first evidence in support of the notion that there may exist network-level signatures that contained information about the distinct propagation mechanisms of focal seizures.

Further, we directly compared the temporal profiles of CC and PL for focal seizures that remain localized and focal seizures with bilateral spread. To account for the unequal number of seizure samples of each seizure type, we implemented a rigorous bootstrapping procedure and established 95% CIs based on which significant difference was assessed (see Materials and methods). As expected from Fig. 3A and B, we observed that the dynamics of CC and PL differed in a *seizure-type-specific* manner only after the onset of seizures. Specifically, CC of focal seizures with bilateral spread was higher than that of focal seizures that remain focal, from 2.25 to 10 min after seizure onset [resampled *P* < 0.05, the smallest value of the Cohen’s *d* within this interval, or *d*_min_ = 1.06, 95% CI_min_ (−0.15, −0.02); Fig. 3C]. Such differences were accompanied by shorter PL associated with focal seizures with bilateral spread [resampled *P* < 0.05 for 2–10 min after seizure onset, Cohen’s *d*_min_ = 0.78, 95% CI_min_ (0.07, 1.06); Fig. 3D]. Notably, these observed differences emerged only after the onset and extended well beyond termination of seizures,^17^ suggesting that focal to bilateral tonic-clonic seizures differentially induced network reorganization that persisted even after the seizure activity ended.

Additionally, after seizure onset, persistent differences in CC and PL were also observed between focal seizures with bilateral spread and interictal activity (Supplementary Fig. 6, right panels). These persistent differences between post-onset activity and interictal periods were, however, not observed in the case of focal seizures that remain focal (Supplementary Fig. 6, left panels). An increase in CC illustrates increased local cliquishness and a decrease in PL implies better connectivity across the underlying network nodes. Together, these findings suggest that the unconstrained propagation dynamics of focal to bilateral tonic-clonic seizures are related to an increase in the overall network connectivity and consequently improved network communication shortly after seizure onset. Critically, these observed seizure-type-dependent network configurations emerged only after the onset, raising a question whether there also existed unique network alterations at other time points that may contribute to the distinct propagation mechanisms and clinical manifestations associated with each seizure type.

### Alterations in the *local connectivity* features after the onset reflect heterogeneous dynamics of focal seizures

**Figure 4:**
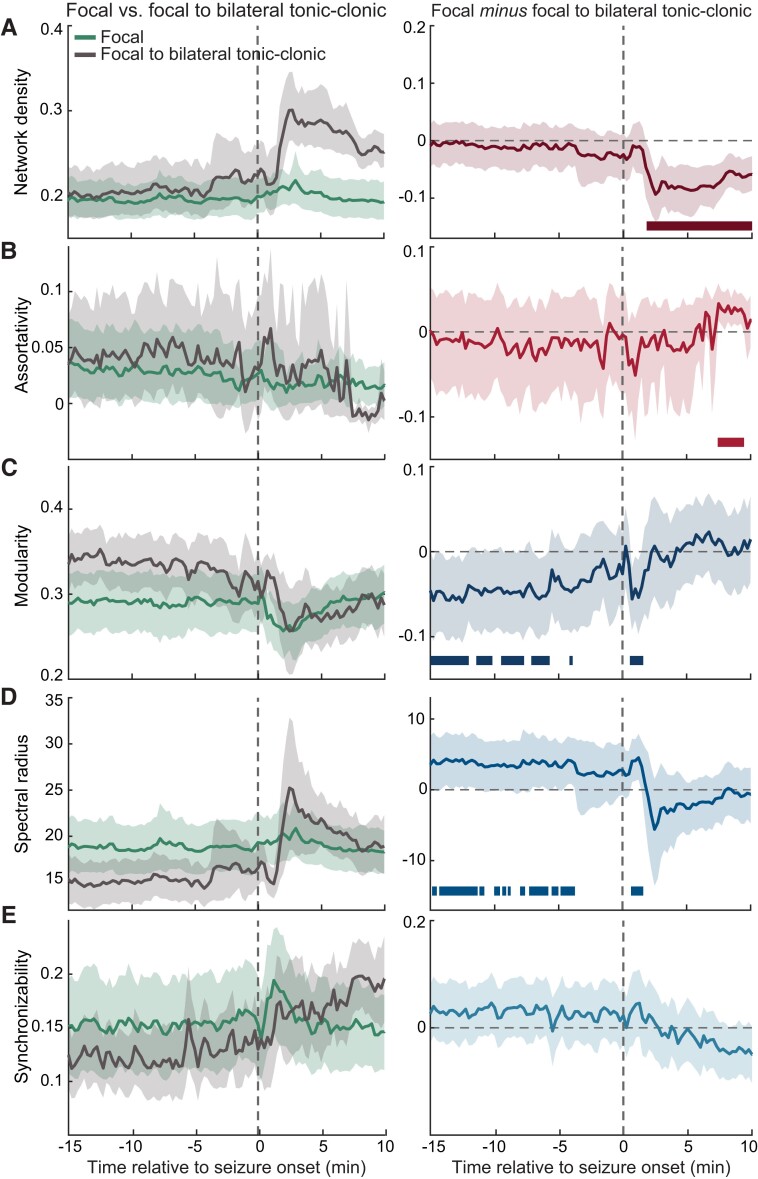
**Various features of functional connectivity networks display distinct temporal changes as a function of seizure propagation dynamics.** Left panels illustrate a series of graph-theoretical measures computed from networks of focal seizures that remain localized (*n* = 49) and from networks of focal to bilateral tonic-clonic seizures (*n* = 18). The time-varying differences observed in each of these features as a function of seizure types are plotted in the corresponding right panels. (**A**) The density of focal to bilateral tonic-clonic seizures is higher than that of focal seizures that remain focal, 1.75–10 min after seizure onset. (**B**) The assortativity, a measure of network robustness, is lower for focal to bilateral tonic-clonic seizures relative to focal seizures that remain focal, 7.5–9.25 min after seizure onset. (**C**) The modularity, which captures efficient network integration and global segregation, is higher for focal to bilateral tonic-clonic seizures when compared with focal seizures that remain focal during temporal windows between 14.75–5.75 min before seizure onset and 0.75–1.50 min after the onset. (**D**) The spectral radius, which relates to the global spread of synchronization in a network, is also higher for focal to bilateral tonic-clonic seizures when compared with focal seizures that remain focal during temporal windows between 14.75–3.75 min before seizure onset and 0.75–1.50 min after the onset. (**E**) The synchronizability, which estimates the propensity of information to diffuse in a network, shows an increasing trend post-seizure onset for unconstrained seizure dynamics. However, no statistically significant differences were observed in synchronizability across seizure types. Statistical comparisons of network measures as a function of seizure types were computed through a bootstrapping procedure where the underlying data distribution of each network measure was resampled at the level of individual seizures to established 95% confidence intervals (CIs). Error bars indicate 95% CIs across individual seizures in each condition and solid bars show resampled *P* < 0.05.

Given the post-onset differences in the CC and the characteristic PL between focal seizures of different propagation mechanisms, we hypothesized that seizure-type-dependent network changes should also be observed in other measures of node connectivity patterns such as the network density. A network with high density is well positioned to optimize integration of information and increase the efficiency of network communication.^38,39^ We expected, therefore, that the networks after the onset of focal seizures with bilateral spread would show higher density when compared with those after the onset of focal seizures that remain focal. Supporting our hypothesis, the network density associated with focal to bilateral tonic-clonic seizures was found to be higher than that of focal seizures that remain focal for 1.75–10 min after the onset [resampled *P* < 0.05, Cohen’s *d*_min_ = 0.73, 95% CI_min_ (−0.10, 0); Fig. 4A]. Notably, the timing of the sustained differences in the density mirrored that of the CC and the characteristic PL, which also extended several minutes beyond seizure termination as each seizure typically lasted between 30 s and 3 min.^59^

To further investigate network alterations unique to particular propagation mechanisms of focal seizures, we assessed the assortativity coefficient which measures the propensity of network nodes to connect to other nodes of similar degree.^40,60^ Our results revealed that the assortativity coefficient associated with focal to bilateral tonic-clonic seizures was lower than that of focal seizures that remain localized for 7.50–9.25 min after seizure onset [resampled *P* < 0.05, Cohen’s *d*_min_ = 0.84, 95% CI_min_ (0.01, 0.06); Fig. 4B]. Additionally, similar patterns of results were observed between focal to bilateral tonic-clonic seizures and interictal activity such that the seizure networks displayed higher network density [resampled *P* < 0.05 for 1.75–10 min after seizures onset, Cohen’s *d*_min_ = 0.92, 95% CI_min_ (0.01, 0.11); Supplementary Fig. 7A) and lower assortativity (resampled *P* < 0.05 for 6.50–7 and 7.50–9.75 min after seizures onset, Cohen’s *d*_min_ = 0.56, 95% CI_min_ (−0.05, 0); Supplementary Fig. 7B]. However, these network properties did not differ between interictal activity and focal seizures that remain localized. Importantly, the observed seizure-type differences emerged after the onset of seizures and were driven by the negative assortativity coefficient that was associated with focal seizures with bilateral propagation. This negative assortativity represents a disassortative network in which high-degree nodes, or network hubs, are less likely to connect with other high-degree nodes in the network.^60,61^

Thus far, we demonstrated that consistent with the differences in the CC and the characteristic PL after seizure onset, the density and the assortativity (i.e. the measures directly derived from local or nodal connectivity), also differed as a function of seizure propagation dynamics. These findings provided better understanding regarding the association between heterogeneous propagation mechanisms of seizure activity and the local connectivity within the underlying functional networks. Next, we asked if networks of different seizure types underwent distinct reconfigurations *prior to* seizure onset that shaped the global properties of the networks and ultimately determined the type of propagation dynamics an impending seizure would display.

### Alterations in subtle *global* network features preceding the onset differentiate propagation dynamics of focal seizures

Building upon the findings presented thus far, we next aimed to quantify the distinct network alterations which could differentiate the propagation patterns prior to seizure onset. To accomplish this, we assessed network attributes related to various aspects of information processing within a networked system, particularly, the brain connectivity network. Specifically, we focused on three network features: modularity,^45,62-64^ spectral radius and synchronizability.^48,65^ While modularity has recently been utilized in characterizing the efficiency associated with integration and segregation of information across distributed brain areas, the properties of spectral radius and synchronizability remain relatively unexplored in the context of brain networks. A couple of recent studies, however, have suggested the utility of synchronizability and spectral radius in describing dynamics of seizure activity within the brain^24^ and the extent of excitability of brain networks, respectively.^23^ Because modularity, synchronizability and spectral radius have been associated with different neural processes and are sensitive to changes in the network connectivity, we hypothesized that these measures would be powerful markers for prediction of seizure dynamics prior to the onset. As described in the Materials and methods, each of these attributes relate to overall network architecture and their values may differ across networks with similar distribution of node degrees. Consequently, we characterized modularity, synchronizability and spectral radius as *subtle global* network features and, in the following, investigated how they change over time as a function of seizure propagation dynamics.

Our results revealed that the information concerning the propagation patterns of focal seizures could be decoded from these global network attributes several minutes prior to seizure onset. Specifically, the modularity preceding the onset of focal seizures with bilateral spread was higher than that of focal seizures that remain localized [resampled *P* < 0.05 for 14.75–12.00, 11.00–10.75, 9.25–8.5, 8.25–8.00, 7.67–7.50 and 6.75–5.75 before seizure onset; Cohen’s *d*_min_ = 0.75, 95% CI_min_ (−0.10, −0.01); Fig. 4C]. This pattern of results was also observed in the spectral radius [resampled *P* < 0.05 for 14.75–14.50, 14.00–11.25, 11.00–10.75, 9.75–9.50, 9.25–9.00, 7.75–7.50, 7.00–5.75, 5.50–5.00 and 4.50–3.75 min before seizure onset; Cohen’s *d*_min_ = 0.66, 95% CI_min_ (0.27, 8.14); Fig. 4D]. These seizure-type-dependent differences in the network modularity and spectral radius re-emerged shortly after seizure onset (resampled *P* < 0.05 for 0.75–1.50 min after seizure onset for both modularity and spectral radius). As for the synchronizability, we observed an increasing trend post-seizure onset associated with unconstrained seizure dynamics; however, our statistical analyses revealed no significant difference in synchronizability across seizure types (Fig. 4E).

Similar patterns of results were also observed between focal seizures with bilateral spread and interictal activity such that preceding the onset, the seizure networks displayed higher modularity [resampled *P* < 0.05 for 14.75–14, 13.75–13, 12.75–12, 10.25–10, 9.75–9.50, 8.50–8.25 and 7.75–7.50 min before seizure onset; Cohen’s *d*_min_ = 0.71, 95% CI_min_ (0.01, 0.09); Supplementary Fig. 7C], and lower spectral radius [resampled *P* < 0.05 for 14.75–14.50, 14–12.25, 11–9.50, 7.75–7.50, 5.25–4.75 and 4.50–3.75 min before seizure onset; Cohen’s *d*_min_ = 0.2, 95% CI_min_ (−7.63, −0.35); Supplementary Fig. 7D]. These results were accompanied by post-onset effects where focal to bilateral tonic-clonic seizures exhibited higher spectral radius (resampled *P* < 0.05 for 0.75–1.50, 2.50–3 and 5–5.50 min after seizure onset; Supplementary Fig. 7D), and higher synchronizability (resampled *P* < 0.05 for 6–6.25 and 7.25–12.50 min after seizure onset; Supplementary Fig. 7E). However, focal seizures that remain localized only differed from interictal activity in the measure of modularity and synchronizability such that the modularity of the focal seizures was lower shortly after seizure onset [resampled *P* < 0.05 for 1–4.25 min after seizure onset; Cohen’s *d*_min_ = 0.41, 95% CI_min_ (−0.06, 0); Supplementary Fig. 7C] and the synchronizability of the focal seizures was higher shortly after the seizure onset [resampled *P* < 0.05 for 1.25–2.25 min after seizure onset; Cohen’s *d*_min_ = 0.59, 95% CI_min_ (0.01, 0.09); Supplementary Fig. 7E].

### Complementary temporal reconfigurations within the functional connectivity networks sculpt seizure dynamics

**Figure 5:**
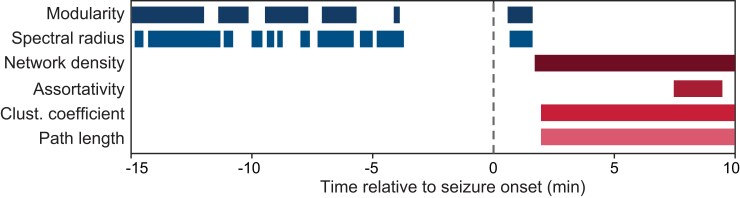
**Summary of graph-theoretical attributes probed across seizure types.** The network features investigated can be categorized into two groups based on the temporal windows at which differential changes in these features emerge as a function of seizure propagation patterns. The time windows where such differences are observed are plotted separately for each of the network measures (resampled *P* < 0.05). Global features, i.e. the modularity and spectral radius, primarily capture network alterations that occur prior to and shortly after seizure onset. In contrast, the density, assortativity, clustering coefficient and characteristic path length characterize post-onset network reconfigurations induced by different types of propagation dynamics.

Using a set of graph-theoretical features, we identified reconfigurations in the functional connectivity network that characterized the propagation dynamics of different seizure types. Our results revealed that such distinguishing features can be classified into two groups based on the distinct and complementary temporal windows at which the differences in these features emerged as a function of seizure types. The first group of network attributes includes the global features modularity and spectral radius, which primarily captures differences between focal seizures with constrained and unconstrained dynamics *prior to* seizure onset (Fig. 5). In contrast, the second group of network properties captured differences across seizure types *after* the onset, reflecting the network reconfigurations induced by distinct propagation mechanisms. Such features include the density, assortativity, CC and characteristic PL (Fig. 5).

**Figure 6:**
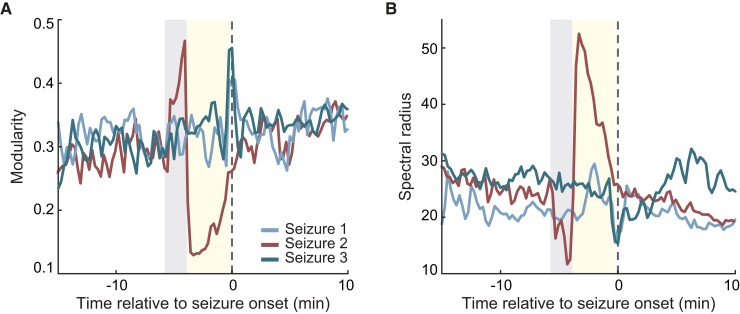
**Distinct patterns of network properties across seizure types at a single-seizure level.** Modularity **A** and spectral radius **B** extracted from networks associated with three seizures that share similar onset regions recorded from a sample patient. Seizures 1 and 3 are categorized by an epileptologist as focal seizures that remain focal (sample recordings of Seizure 1 is illustrated in Fig. 1B, left), whereas Seizure 2 is categorized as a focal to bilateral tonic-clonic seizure (sample recordings of Seizure 2 is also illustrated in Fig. 1B, right). Seizures 1 and 3 exhibit similar patterns of modularity and spectral radius overtime, and these temporal dynamics differ from those of Seizure 2. The grey-shaded region highlights the robust signatures associated with the bilateral spread of a focal seizure (i.e. increase in modularity and decrease in spectral radius) and is followed by network changes which are likely seizure and/or patient specific.

Notably, the first group of network features, i.e. modularity and spectral radius, can detect the possibility of an impending seizure to spread bilaterally and can be further investigated as potential biomarkers. To further highlight the utility and robustness of these features in assessing the likelihood of a focal seizure to spread bilaterally, we evaluated modularity and spectral radius at a single-seizure level in three seizures that shared similar onset regions in the left hemisphere and were recorded from a single patient (Figs 1 and 6). Specifically, seizure two bilaterally propagated after the onset, while Seizures 1 and 3 remained localized within the left hemisphere. We showed that Seizures 1 and 3 exhibit similar dynamics of modularity and spectral radius over time, and these temporal patterns differ from those of Seizure 2. For Seizure 2, we observe an early sharp increase in modularity and a decrease in spectral radius consistent with the findings across seizures (Fig. 5), further highlighting the robustness of our results (grey-shaded area). These patterns were followed by an abrupt decrease in modularity and a sharp increase in spectral radius (Fig. 6; yellow-shaded area) which could be seizure- and/or patient-specific features. These results further suggested that network measures could potentially be used to characterize distinct neural dynamics across different types of focal seizures, even on a single-seizure basis.

## Discussion

The goal of the present study was to investigate if the emergence of heterogeneity in seizure propagation is an outcome of mechanistically different disruptions and can be understood in terms of network-level changes within the brain before, during and after the onset. To accomplish this, we evaluated the temporal evolution of a series of graph-theoretical attributes which quantify various aspects of network organization and information processing within complex systems such as the brain. We demonstrated distinct network-level signatures that were associated with the extent of diffusion dynamics of an impending seizure as well as isolated architectural changes within the functional connectivity networks that emerged as the seizures terminated. These results advance our understanding of how heterogeneous seizure dynamics can arise from similar onset regions. Furthermore, our findings provide a rationale for a potential use of network features to help guide clinical diagnosis of focal seizures with different propagation mechanisms (focal seizures that remain local versus focal to bilateral tonic-clonic seizures). Incorporation of such network measures with other biomarkers of neural state changes^4,7,54^ could also potentially lead to improvement of effective intervention strategies to constrain propagation of seizure activity.

### Network alterations track temporal evolution of focal seizures

We demonstrate that dynamic reconfigurations within the functional connectivity networks during evolution of focal seizures give rise to the heterogeneity observed across seizures. While past work primarily evaluated network properties of epileptic brain by averaging the signals in large discrete time windows, we assess the continuous temporal evolution of network connectivity in combination with resampling statistical tests. This formulation enables us to characterize the temporal dynamics of network alterations that underlie the emerging dynamics of seizure activity in a rigorous manner. By examining *globally defined* network features, we observe distinct macroscopic signatures which could predict the extent of diffusivity of seizure propagation minutes *prior to* the onset. Our results indicate that processes leading to the emergence of distinct seizure propagation patterns are coded in the network activity minutes prior to the onset and can have mechanistically different underpinnings. We argue that our approach provides an objective framework not only for better understanding the neural dynamics underlying evolution of seizures but also for determining whether and when a clinical intervention should be implemented to manage and control a spread of an impending seizure.

Additionally, we further demonstrate that the heterogeneous dynamics exhibited across seizure types can be characterized by *post-onset* changes in local features of the functional connectivity networks such as the CC, characteristic PL and network density. Specifically, our findings revealed a relationship between the CC, characteristic PL and a binary measure of diffusivity of seizure activity after the onset (i.e. whether the seizure activity would remain within the same hemisphere or spread transcollosally). These measures indicate higher network connectivity following the seizure onset which could be correlated with more severe cognitive impacts that the seizures with unconstrained propagation dynamics typically induce. Any such correlation, however, needs to be confirmed with future experimentation. Notably, immediately after the onset where we observed higher network connectivity, no significant changes in the assortativity were observed. These results indicate that there was no particular reconfiguration in how the network hubs connect either with one another or with non-hubs. These observed patterns, however, changed closer to seizure termination—the networks of focal seizures with unconstrained dynamics showed negative assortativity indicative of a disassortative network, where highly connected nodes (hubs) are less connected to one another. Assortativity can be directly linked to the robustness of the network, in terms of its ability to remain connected.^60^ A failure of a hub node in an assortative network would leave other hub nodes connected to one another, minimizing the chance of the network as a whole to become disconnected. However, in case of a seizure that spreads bilaterally, emergence of a disassortative network likely reflects a mechanism to reduce this ‘robustness’ by restricting the connectivity among network hubs.

### Bilateral propagation of focal seizures reflects over-compensation for an imbalance in global integration and excitability

Preceding the onset, we reported increased modularity along with decreased spectral radius in focal to bilateral tonic-clonic seizures. In the light of classical accounts on the mechanistic underpinnings of seizures, we argue that our findings could reflect the chemical or dynamic imbalance within the underlying networks.^6,66^ Specifically, microscopic disproportion between excitation and inhibition or in the bistability of localized neural dynamics could lead to an emergence of a neural state with significantly low integration (or high segregation captured by high modularity^39^) and low excitability (captured by low spectral radius).^23^ To regain a more balanced state, it is likely that mechanisms enhancing the connectivity between segregated networks are recruited, leading to an over-compensation which manifests as more unconstrained seizure dynamics. Future studies utilizing large-scale non-invasive neuroimaging methods could seek validation and/or refinement to this hypothesis. Notably, we found that such signatures, i.e. increased modularity as well as decreased spectral radius, disappeared just before the onset, re-emerged shortly after and again disappeared (Fig. 4). It is, therefore, possible that these network features reflect the manifestations of the regulatory mechanisms that govern emergence and termination of seizures. In addition, the modularity of focal seizures that remain focal decreased shortly after the onset when compared with interictal activity (Supplementary Fig. 7C), further highlighting the link between this network feature and control mechanisms associated with seizure termination.

While we did not observe a significant difference of synchronizability between focal seizures with constrained and unconstrained dynamics, Khambhati *et al*.^24^ showed significantly high synchronizability for unconstrained dynamics within the gamma oscillations. Further, investigation of network reconfiguration within specific oscillatory bands can be helpful in drawing associations between network reconfigurations and cognitive implications of a specific seizure type.

### Time-dependent assessment of network attributes can guide development of personalized seizure treatment

The heterogeneity of epilepsy is a key confound to disease understanding and development of effective treatments. Here, we demonstrate graph-theoretical features as novel potential candidates of biomarkers that link differential reconfigurations of the functional connectivity networks to the heterogeneity in the emerging seizure dynamics. Specifically, our investigations of the global network dynamics suggest that interventions aiming to contain the spread of seizure activity may wish to situate the brain in a topological state where the modularity is lowered, while the spectral radius is increased. In addition, we also show that the information regarding the propagation patterns of seizures can be decoded through the seizure-type-dependent changes in the network properties several minutes before seizure onset allowing sufficient time for an intervention to be implemented. Furthermore, the seizure-type-dependent signatures observed post-onset can be used to validate the efficiency of a particular treatment approach in preventing evolution of seizures and may help determine the extent of cognitive and behavioural deficits induced by the residue seizure activity in a scenario where the intervention did not completely eliminate the seizures. Future studies that wish to characterize cognitive and behavioural changes induced by neurological disorders may also benefit from evaluating these network properties in relation to performance of patients on various test battery.^18,67^ Such analyses could uncover distinct underlying pathophysiological processes that give rise to diverse cognitive and behavioural impairments across disease subtypes and across individuals, thereby improving understanding of the disease heterogeneity.^68-71^ Finally, our single-seizure analyses suggest that network measures could potentially be used to characterize distinct neural dynamics across different types of seizures, even on a single-seizure basis. Such findings provide foundation for future investigation and development of effective personalized seizure treatment.

### Future directions

Given that the electrode placement was determined on a patient-to-patient basis by a neurologist for the purpose of identification of seizure onset zones, the data extracted from these electrodes inevitably provide an incomplete picture of the brain network due to the resulting partial coverage. In addition, the reported lack of differences between focal seizures that remain focal and interictal activity could be partially due to such spatial sampling of the recorded signals. To address this possibility, future studies may benefit from non-invasive recordings where whole-brain dynamics can be simultaneously evaluated.

Further, our analyses treated multiple seizures and interictal activity segments from the same patients as independent, and primarily disregarded individual variability in seizure heterogeneity at the patient level. This analytical choice was made based on traditional methods (e.g. see Martinet *et al*.^4^), and careful statistical comparisons were implemented to identify the seizure-type-dependent alteration patterns in the functional connectivity networks of seizures. To further extend our findings and improve the specificity of the interpretations, future studies may incorporate patient-level factor in their analytical frameworks. While we reported distinctive network features to distinguish constrained and unconstrained seizure dynamics, it is likely that some of the subtle differences remained undetected given our temporal resolution and strict statistical approach. By combining our analytical framework with a finer temporal resolution, future studies can look for unique network features associated with different phases of seizure propagation as a function of seizure types.

We illustrated the potential utility of complex network features in distinguishing seizures with constrained and unconstrained dynamics prior to the onset at a single-seizure level. However, in this sample patient, we also found patterns of network reconfigurations that were not observed at the population level. Using the analytical framework we presented, future studies can further validate the seizure- and patient-specific applications of network features in clinical settings.

## Conclusions

In summary, by using a graph-theoretical approach, we determined the extent to which distinct emerging dynamics of seizure networks were accounted for by temporal reconfigurations of the underlying functional connectivity. Collectively, our results illustrated a series of network metrics that can potentially be utilized as quantitative biomarkers to distinguish between focal seizures of distinct dynamics based on their propagation patterns as well as the differential extent of cognitive and behavioural effects accompanying the seizures. These results suggested that the networks of focal seizures with unconstrained dynamics undergo early network alterations, triggering processes which facilitate the bilateral diffusion of seizure activity. The propagation-type-dependent alterations in these metrics were observed again shortly after the onset, suggesting that these measures could also induce regulatory mechanisms necessary for the termination of seizures. Together, our findings provide objective means to gain better insight into the mechanisms by which seizure dynamics are regulated within the brain and provide exciting avenues where graph-theoretical measures could be used to guide personalized clinical interventions.

## Supplementary Material

fcac234_Supplementary_Data

## Abbreviations

CC=: clustering coefficient
CI=: confidence interval
ECoG=: electrocorticography
PL=: characteristic path length
