# Supplementary material for: Brain network dynamics codify heterogeneity in seizure evolution
Source: Brain Commun. 2022 Sep 16;4(5):fcac234. doi: 10.1093/braincomms/fcac234 (PMC9527667; doi:10.1093/braincomms/fcac234)
Supplement: fcac234_Supplementary_Data [file fcac234_supplementary_data.zip › Manuscript_original_submission.pdf]

OXFORD UNIVERSITY PRESS | Brain Communications

Brain network dynamics codify heterogeneity in seizure evolution

Journal:	*Brain Communications*
Manuscript ID	BRAINCOM-2021-255
Manuscript Type:	Original Article
Date Submitted by the Author:	21-Jul-2021

Complete List of Authors:	Rungratsameetaweemana, Nuttida ; US Army Research Laboratory Human Research and Engineering Directorate; Salk Institute for Biological Studies Computational Neurobiology Laboratory; King Mongkut's University of Technology Thonburi, Bangkok, Neuroscience Center for Research and Innovation<br>Lainscsek, Claudia ; Salk Institute for Biological Studies Computational Neurobiology Laboratory<br>Cash, Sydney; Massachusetts General Hospital Department of Neurology, Neurology<br>Garcia, Javier; US Army Research Laboratory Human Research and Engineering Directorate<br>Sejnowski, Terrence ; Salk Institute for Biological Studies Computational Neurobiology Laboratory<br>Bansal, Kanika; Columbia University, ; US Army Research Laboratory Human Research and Engineering Directorate,

Keywords:	epilepsy, functional connectivity, seizure propagation, brain networks

SCHOLARONE™
Manuscripts

Brain network dynamics codify heterogeneity in seizure evolution

Nuttida Rungratsameetaweemana[1,2,3], Claudia Lainscsek[2,4], Sydney S. Cash[5],

Javier O. Garcia[1], Terrence J. Sejnowski[2,4,6,†], Kanika Bansal[1,7,†]

[†]**Senior authors.**

Abstract

Dynamic functional brain connectivity facilitates adaptive cognition and behavior. Abnormal alterations within such connectivity could result in disrupted functions observed across various neurological conditions. As one of the most common neurological disorders, epilepsy is defined by the seemingly random occurrence of spontaneous seizures. A central but unresolved question concerns the mechanisms by which extraordinarily diverse propagation dynamics of seizures emerge.

Here, we apply a graph theoretical approach to assess dynamic reconfigurations in the functional brain connectivity before, during, and after seizures that display heterogeneous propagation patterns despite sharing similar cortical onsets. We computed time-varying functional brain connectivity networks from human intracranial recordings of 67 seizures (across 14 patients, 49 focal seizures that remain focal and 18 focal seizures with bilateral spread). In addition, functional connectivity networks estimated from 67 interictal periods were utilized as control. Our results characterize quantitative network features underlying the heterogeneity of seizure propagation dynamics and the accompanying clinical manifestations. Decoding these network properties demonstrate that bilateral propagation of seizure activity is an outcome of the imbalance of global integration and segregation in the brain prior to seizure onset. Further, our approach shed light on differential network mechanisms that lead to distinct propagation dynamics of seizures.

Our results suggest that (i) there exist intrinsic network signatures preceding seizure onset that are predictive of the extent to which seizure activity would diffuse through the brain; (ii) such features emerge several minutes prior to the onset and could aid successful intervention strategies; and (iii) distinct network characteristics emerge after seizure onset that are unique to the propagation mechanisms of focal seizure subtypes, indicative of reconfiguration processes which may assist termination of seizures. Together, our findings provide insights

For Review Only

into the associations between evolution of seizures and their underlying functional connectivity dynamics. These results offer exciting avenues where graph theoretical measures could be used to guide personalized clinical interventions for neurological disorders such as epilepsy, which displays extensive heterogeneity in its clinical and neurological manifestations across as well as within individual patients.

Author affiliations:

[1]Human Research and Engineering Directorate, US DEVCOM Army Research Laboratory, MD 21005, USA.

[2]Computational Neurobiology Laboratory, The Salk Institute for Biological Studies, CA 92037, USA.

[3]Neuroscience Center for Research and Innovation, Learning Institute, King Mongkut's University of Technology Thonburi, Bangkok, 10140, Thailand.

[4]Institute for Neural Computation, University of California San Diego, La Jolla, CA 92093, USA.

[5]Department of Neurology, Massachusetts General Hospital and Harvard Medical School, MA 02114, USA.

[6]Division of Biological Sciences, University of California San Diego, CA 92093, USA.

[7]Department of Biomedical Engineering, Columbia University, NY 10027, USA.

Correspondence to: Kanika Bansal

Full address: Department of Biomedical Engineering, Columbia University, New York, NY 10027

E-mail: phy.kanika@gmail.com

Running title (40 characters max): Network reconfigurations sculpt seizures

Keywords: epilepsy; functional connectivity; seizure propagation; brain networks

For Review Only

Abbreviations: ECoG = Electrocorticography

Introduction

As one of the most common neurological disorders with roughly 50 million cases world-wide, epilepsy is characterized by its emerging spontaneous seizure activity.[1,2] Critically, one-third of the patients do not respond to medications and rely on alternative interventions such as surgical and neuromodulatory.[3–5] However, seizures are remarkably diverse, and tailoring effective treatment strategies remains a challenge partly due to the temporal spontaneity and lack of objective frameworks that could characterize the onset and propagation patterns of an impending seizure.[6,7] Traditionally, the variability across seizures has been addressed through classification based on the onset regions: *focal seizures* originate from a localized region within one hemisphere while *generalized seizures* begin simultaneously from both hemispheres. A variety of computational techniques in the realm of network science and dynamical systems have been employed to better localize the onset regions and thus improve the precision with which focal and generalized seizures can be identified.[8–10] However, localizing onset regions does not fully capture the breadth of dynamics and diversity associated with seizure subtypes. Adding to this complexity, once generated, a focal seizure can remain localized within the same hemisphere (i.e., *focal seizures that remain focal*) or propagate to the other hemisphere (i.e., *focal to bilateral tonic-clonic seizures* or *focal seizures with bilateral spread).*[11–13] Notably, these subtypes of focal seizures can coexist in a single patient (Fig. 1), where the seizures with bilateral spread generally lead to more severe behavioral and cognitive deficits that could require several minutes to hours for patients to recover from. However, the distinct propagation dynamics exhibited by different seizure types are largely ignored by traditional intervention approaches and it remains unknown if the mechanisms underlying the bilateral spread of focal seizure activity differ from those associated with focal seizures that remain localized. Critically, the field currently lacks an objective analytical framework that can be utilized to investigate, understand, and predict the heterogeneity associated with propagation dynamics of seizure activity.

Here, we demonstrate that the long-standing challenges associated with the heterogeneity observed across subtypes of epileptic seizures can be addressed through the lens of graph theory, a formulation in which brain dynamics can be visualized as temporally evolving graphs or networks composed of nodes and edges that represent brain regions and their

For Review Only

pairwise associations, respectively.[14–16] This approach relies on a complex systems view of the brain where a single brain region interacts with many others and collectively, these interactions give rise to a wide variety of functional connectivity patterns serving adaptive cognition and behavior. Investigating the temporal evolution of such connectivity patterns, within a graph theoretical framework, has provided better insight into the emergence of neural properties such as specialization and efficiency of information processing, learning, and aging[17–22] and has applications in clinical neuroscience for the potential to establish biomarkers of disease onset and progression. Specifically, grounded in graph theory, such techniques can identify abnormal alterations in the dynamic functional brain connectivity that are caused by neurological conditions.[17,23–28]

Our study is built upon the idea that the manner in which functional brain connectivity networks reconfigure over time carries information concerning the emergent seizure dynamics and cognitive behaviors that are unique to the underlying neurological processes. Consequently, we probed the time-varying changes within functional connectivity networks derived from multiple hours of electrocorticogram (ECoG) recordings across 14 patients as they experienced focal seizures that remain focal or focal to bilateral tonic-clonic seizures. With this analytical framework, we aimed to gain insight into the unique nature of how the heterogeneous dynamics associated with different seizure types develop and unfold in the brain. Our results elucidate key network features that characterize the differential neural dynamics as well as the distinct cognitive and behavioral changes associated with each type of focal seizures. Further, our findings demonstrate that the emergence of different propagation patterns is an outcome of unique network-level changes and distinct mechanisms that regulate the extent of synchronization within the brain.

For Review Only

Materials and methods

Patient information and data acquisition

The seizures analyzed in this study were recorded from 14 patients with medication-refractory epilepsy (Table 1) who underwent a clinical monitoring procedure to locate their seizure onset zone. Clinical electrode implantation, positioning, duration of recordings, and medication schedules were based solely on clinical need as determined by an independent team of clinicians. As indicated in Table 1, seizures analyzed in this study are of two types: 1) focal

seizures that remain focal and 2) focal to bilateral tonic-clonic seizures or focal seizures with bilateral spread. Patients were implanted with intracranial subdural grids, strips, and depth electrodes for several days in a specialized hospital setting and continuous multichannel ECoG data were recorded at a sampling rate of 500 Hz.

Only seizures with an obvious ictal onset were selected for analysis. Experienced epileptologists, blind to this study, identified the seizure onset regions, seizure types, and onset time through inspection of the ECoG recordings, referral to the clinical report, and clinical manifestations recorded on video. A total of 67 seizures (49 focal seizures that remain focal and 18 focal to bilateral tonic-clonic seizures) were analyzed. We note that, multiple seizures from the same patients were treated as independent (see similar methods in[4]).

Ethics statement

All patients were enrolled after informed consent was obtained and approval was granted by local Institutional Review Boards (IRB) at Massachusetts General Hospital (MGH) according to National Institutes of Health (NIH) guidelines.

Data preprocessing

For each of these seizures, we considered ECoG data of the duration of 15 minutes before and 10 minutes after the seizure onset. Each of these 25-min data segments only contained one seizure. For comparison with relatively 'seizure-free' (interictal) activity, we extracted an equal number of interictal activity windows with the same duration. Interictal windows were selected from ECoG recordings at least an hour away from an onset and offset of any seizure. The data were band-pass filtered between 1 to 70 Hz, and notch filtered at 60 Hz to exclude potential powerline interference. A common reference was used for data analysis and the reference electrode in each case was located far from the area of recording making the introduction of spurious correlation or elimination of actual correlation between cortical regions unlikely[29].

Functional connectivity networks

To evaluate functional connectivity representations associated with the temporal evolution of seizures, we employed complex network analysis. Originated from the mathematical study of networks known as graph theory, such analytical framework represents a real-world complex

For Review Only

system as a network that is composed of a collection of nodes with edges connecting different node pairs. Here, we constructed time-varying functional connectivity networks of focal seizures and interictal activity where the inter-electrode relationships were represented by network edges and the electrodes themselves were represented by nodes in the corresponding networks of seizures and interictal activity (Fig. 2, A-D).

Specifically, we computed symmetric functional connectivity C_{ij} between two regions of the brain i and j as an averaged correlation of the neural signals recorded by the intracranial electrode contacts of those regions. To extract (at least approximately) the stationary aspects of ECoG data, we divided each of the 25-min ECoG data segments into consecutive 1-s windows, where each window overlapped the previous window by 0.5 seconds.[30,31] The correlation was calculated within each of these 1-s segments. To account for noise, we applied a temporal smoothing to these correlation values by averaging consecutive 30 seconds windows such that a total of 98 correlation values representing the functional connectivity of 98 temporal windows were generated from each 25-min ECoG data segment. Note that different temporal smoothing parameters can be use without affecting the overall patterns of results, although a value too large may reduce the temporal precision of the observations (Supplementary Fig. 1).

All correlation values were bounded between −1 and +1. Negative correlation values implying long range inhibitions were then set to zero, as within our modelling framework and in line with previous studies[3,32], we do not consider the contribution of long range direct inhibitory connections to the simulation of the epileptogenic effect. Temporal evolution of these correlations or connectivity matrices reflects the time-varying dynamics of the functional brain networks as recorded through the ECoG measurements.

For Review Only

Graph theoretical network analysis

For each seizure, we constructed a series of weighted, symmetric (undirected) connectivity matrices C representing functional correlations across all recording electrodes. From these network matrices, we computed a series of graph theoretical network measures (described below) as a function of seizure types to quantify changes in network dynamics associated with evolution of focal seizures with constrained (focal seizure that remain focal) and unconstrained propagation mechanisms (focal to bilateral tonic-clonic seizures). We used various Brain Connectivity Toolbox functions implemented in MATLAB (R2020; MathWorks) for our computation of these network features unless noted otherwise.

Assessing small-world architecture

In general, a network can range from completely regular where each node connects to its nearest neighbors to fully random where node pairs are connected randomly with some probability.[33–36] Within this spectrum lies a small-world architecture which is characterized by a combination of dense local clustering of connections between neighboring nodes (like regular networks) and a short path length between distant node pairs due to the existence of relatively few long-range connections (like random networks).[14,15,17,37] This architectural scheme is known to facilitate both specialized and distributed information processing in a cost-effective manner. Mathematically, small-world architecture is characterized by high clustering coefficient and low characteristic path length as compared to a random network.

Clustering coefficient

Clustering coefficient is a measure of local connectedness of a network and has been used to describe the segregation of information in brain networks. The clustering coefficient is calculated as the ratio between the number of triangles present around a node and the maximum number of triangles that could possibly be formed around that node.[27,38] For a given node X and any other two nodes Y and Z within the network, a triangle around X represents a scenario where X, Y, and Z all have a connectivity value of one with one another. We used the Brain Connectivity Toolbox function *clustering_coef_wu* for the calculation of clustering coefficient.

Characteristic path length

Characteristic path length describes the averaged minimum distance between all pairs of nodes in a network and has been shown to associate with the integration of information within the brain. The minimum path length between a pair of network nodes represents the shortest route between them through a combination of network edges. We calculated characteristic path length using the Brain Connectivity Toolbox function *charpath*.[39]

Degree per node

Degree of a node represents the total sum of edge weights connected to a node in the network. To compare seizures across individuals who had different number of implanted electrodes, we computed the average degree per node which represents on an average, the total sum of edge weights connected to a node in the network. A high average degree per node indicates a large number of connections and this measure represents the 'wiring cost' of the network. A network

For Review Only

with high degree per node is well positioned to optimize integration of information and increase the efficiency of network communication.[37,40]

For a given node i, the degree is defined as $A_i = \sum_j C_{ij}$ where, C represents the connectivity matrix. Then, degree per node is calculated as $< A >= \frac{1}{N} \sum_{i=1}^{N} A_i$ where, N represents the total number of nodes in the network.

Assortativity

Assortativity measures the propensity of nodes to connect to others with similar degree and is calculated as a correlation coefficient between the degrees of all the nodes.[41] A positive assortativity value indicates that nodes tend to link to other nodes with similar degree, whereas a negative value indicates connected nodes with dissimilar degree. Networks with high assortativity tend to make a highly connected core of network hubs. Functional brain networks have been shown to display such architecture with highly connected hub regions or core surrounded by low-connectivity peripheral nodes.[42,43] Assortativity quantifies network robustness as a removal or failure of a single high-degree node would induce greater impact on communication efficiency of a network with low assortativity than on a network with high assortativity. We calculated assortativity using the Brain Connectivity Toolbox function *assortativity_wei.*[39]

Modularity

Modularity describes the extent to which a graph can be divided into clearly separated communities (i.e., subgraphs or modules). Each module contains several interconnected nodes, and there are relatively few connections between nodes of different modules. In the context of brain networks, modularity has been used to describe and quantify efficient integration and segregation of information across distributed sets of brain regions as a function of cognitive task demands.[44,45] We used the Brain Connectivity Toolbox function *modularity_und* to compute modularity of functional brain networks.[46,47]

Synchronizability

Synchronizability relates to the viability of synchronized dynamics within a network. Particularly in the context of epilepsy, relatively larger value of synchronizability has been

For Review Only

associated with greater ease for neural populations to synchronize their dynamics.[22] Synchronizability (S) is calculated as the ratio of the second smallest and the largest eigenvalue of the Laplacian matrix (L), which is computed as the difference between the diagonal matrix of node strength (total degree) and the adjacency matrix such that $L = D - C$. Thus, synchronizability estimates the spread of the eigenvalues of the network Laplacian and is computed as $S = \frac{\lambda_2^L}{\lambda_{\max}^L}$ where λ_2^L and $\lambda_{\max}^L$ denote the second smallest and the largest eigenvalue of L, respectively.

Spectral radius

Spectral radius is a global measure of network structure that is related to the spread of synchronization in a network.[21,48,49] Computed as the largest eigenvalue of the connectivity matrix (C), spectral radius reflects the critical coupling strength required to synchronize the system.[50] As such, spectral radius represents the principal component of the system and contains information about structural characteristics as well as dynamical behavior and stability of the underlying network.[51–53] In the network based models of brain dynamics, spectral radius has been associated with the ease with which the system can be transitioned into an excited state.[21]

Statistical analysis

To compare the computed network measures as a function of seizure types in a time-resolved manner and to assess significant differences, we performed a bootstrapping procedure and established 95% confidence intervals for each corresponding measure. For each of the bootstrapping iterations, we performed resampling with replacement at the level of individual seizures and computed averages for comparison of interest (e.g., the clustering coefficient of focal seizures that remain focal vs. the clustering coefficient of focal to tonic-clonic seizures). We performed 10,000 bootstrapping iterations in order to achieve the confidence intervals reported (CIs) for each comparison. Note that this approach constrains the resolution of P values to a lower limit of $P \leq 0.0001$. We generated permuted null distributions of each complex network measure for each individual seizure and each time point. For tests comparing a bootstrapped distribution against zero, P values were computed by conducting 2 one-tailed tests against zero (e.g., mean[difference in clustering coefficients < 0] and mean[difference in clustering coefficients > 0] and doubling the smaller P value).

For Review Only

Data availability

All data that support the findings of this study are present in the main text and/or the Supplementary Materials. These data are available on request from the corresponding author. The data are not publicly available as they contain information that could compromise privacy of the research participants.

Results

More prominent small-world connectivity links to bilateral spread of seizure activity

In the context of epilepsy, increased small-world connectivity has been proposed as a potential driver for the propagation of pathological synchronous activity across brain regions.[54–56] Consequently, we first assessed the clustering coefficients and the characteristic path lengths to examine whether such properties relate to the constrained and unconstrained propagation mechanisms associated with focal seizures that remain focal and focal seizures with bilateral spread, respectively. To accomplish this, we calculated the clustering coefficient and the characteristic path length of each connectivity matrix (i.e., 98 matrices per each of the 25-minute segments of seizure activity). To evaluate these results in light of past studies, we computed averages of these values in a series of consecutive 5-minute windows, separately for each seizure type. This resulted in three preictal, one ictal (during seizure), and one postictal windows (Fig. 3, A and B). A similar analysis was applied to interictal data to estimate baseline values to which the seizure-related network measures could be compared.[39]

Our results reveal that both focal seizures that remain focal and focal to bilateral tonic-clonic seizures displayed higher small-world connectivity during ictal periods when compared to seizure-free (interictal) activity as demonstrated by higher clustering coefficients and shorter characteristic path lengths (Fig. 3, A and B). Further analyses demonstrated that the ictal activity associated with focal seizures that remain focal exhibited (i) higher clustering coefficients as compared to postictal period (P = 0.03; Fig. 3A, left panel) and (ii) lower characteristic path lengths as compared to both preictal and postictal periods (preictal: P = 0.0004, < 0.0001, 0.0002; postictal: P = 0.0006; Fig. 3B, left panel). Additionally, we observed similar changes for focal seizures with bilateral propagation where the ictal activity displayed

For Review Only

(i) higher clustering coefficients as compared to all the preictal periods (all $P < 0.0001$; Fig 3A, right panel) and (ii) shorter characteristic path lengths as compared to all the preictal periods (all $P < 0.0001$; Fig 3B, right panel). However, unlike the characteristic path lengths associated with the postictal periods of focal seizures with constrained dynamics which returned to the preictal levels, the postictal path length of focal seizures with bilateral spread exhibited a continued decrease ($P = 0.0006$, Fig 3B, right panel). These results supported the more unconstrained diffusivity associated with focal to bilateral tonic-clonic seizures. Notably, the observed differences regarding the manner in which the small-world architecture increased in the networks of focal seizures with constrained and unconstrained dynamics were our first evidence in support of the notion that there may exist network-level signatures that contained information about the distinct propagation mechanisms of focal seizures.

Further, we directly compared the temporal profiles of the small-world architecture for focal seizures that remain localized and focal seizures with bilateral spread. To account for the unequal number of seizure samples of each seizure type, we implemented a bootstrapping procedure and established 95% confidence intervals based on which significant difference was assessed (see Materials and methods). As expected from Figures 3A-B, we observed that the dynamics of small-world properties differed in a *seizure-type specific manner* only after the onset of seizures. Specifically, the clustering coefficient of focal seizures with bilateral spread was higher than that of focal seizures that remain focal, from 2 to 10 minutes after seizure onset (resampled $P < 0.05$; Fig. 3C). Such differences were accompanied by the shorter characteristic path length associated with focal seizures with bilateral spread (resampled $P < 0.05$ for 2-10 minutes after seizure onset; Fig. 3D). Notably, these observed differences emerged only after the onset and extended well beyond termination of seizures[55], suggesting that focal to bilateral tonic-clonic seizures differentially induced network reorganization that persisted even after the seizure activity ended.

Additionally, after seizure onset, persistent differences in the clustering coefficient and the characteristic path length were also observed between focal seizures with bilateral spread and interictal activity such that focal seizures with bilateral spread displayed more prominent small-world configuration (Supplementary Fig. 2, right panels). These persistent differences between post-onset activity and interictal periods were, however, not observed in the case of focal seizures that remain focal (Supplementary Fig. S2, left panels). Together, these findings suggested that the unconstrained propagation dynamics of focal to bilateral tonic-clonic seizures related to an increase in the efficiency of network communication, as illustrated by the

For Review Only

increased small-world characteristic shortly after seizure onset. Critically, these observed seizure-type dependent network configurations emerged only after the onset, raising a question whether there also existed unique network alterations at other time points that may contribute to the distinct propagation mechanisms and clinical manifestations associated with each seizure type.

Alterations in the *local* network connectivity features after the onset reflect heterogeneous dynamics of focal seizures

Given the post-onset differences in the clustering coefficient and the characteristic path length between focal seizures of different propagation mechanisms, we hypothesized that seizure-type dependent network changes should also be observed in other measures of node connectivity patterns such as the degree per node. A network with high degree per node is well positioned to optimize integration of information and increase the efficiency of network communication.[37,39] We expected, therefore, that the network nodes after the onset of focal seizures with bilateral spread would be of higher degree on average as compared to those after the onset of focal seizures that remain focal. Supporting our hypothesis, the degree per node associated with focal to bilateral tonic-clonic seizures was found to be higher than that of focal seizures that remain focal for 1.75-10 minutes after the onset (resampled $P < 0.05$, Fig. 4A). Notably, the timing of the sustained differences in the degree per node mirrored that of the clustering coefficient and the characteristic path length, which also extended several minutes beyond seizure termination as each seizure typically lasted between 30 seconds and 3 minutes.[57]

To further investigate network alterations unique to particular propagation mechanisms of focal seizures, we assessed the assortativity coefficient which measures the propensity of network nodes to connect to other nodes of similar degree.[41,58] Our results revealed that the assortativity coefficient associated with focal to bilateral tonic-clonic seizures was lower than that of focal seizures that remain localized for 7.50-9.50 minutes after seizure onset (resampled $P < 0.05$, Fig. 4B). Additionally, similar patterns of results were observed between focal to bilateral tonic-clonic seizures and interictal activity such that the seizure networks displayed higher degree per node (resampled $P < 0.05$ for 1.75-10 minutes after seizures onset; Supplementary Fig. 3A) and lower assortativity (resampled $P < 0.05$ for 6.50-7 and 7.50-9.75 minutes after seizures onset; Supplementary Fig. 3A). However, these network properties did not differ between interictal activity and focal seizures that remain localized.

For Review Only

Importantly, the observed seizure-type differences emerged after the onset of seizures and were contributed by the negative assortativity coefficient that was associated with focal seizures with bilateral propagation. These results suggested that close to seizure termination, the networks of focal seizures with unconstrained dynamics underwent reduced robustness, rendering them more susceptible to network disruptions.[58,59] These findings could potentially account for the more extensive behavioral abnormalities and cognitive deficits often observed after patients experience episodes of focal to bilateral tonic-clonic seizures.[12,60–62]

Thus far, we demonstrated that consistent with the differences in the clustering coefficient and the characteristic path length after seizure onset, the degree per node and the assortativity (i.e., the measures directly derived from local or nodal connectivity), also differed as a function of seizure propagation dynamics. These findings provided better understanding regarding the association between heterogeneous propagation mechanisms of seizure activity and the local connectivity within the underlying functional networks. Next, we asked if networks of different seizure types underwent distinct reconfigurations *prior to* seizure onset that shaped the global properties of the networks and ultimately determined the type of propagation dynamics an impending seizure would display.

Alterations in *global* network features preceding the onset predict propagation dynamics of focal seizures

Building upon the findings presented thus far, we next aimed to quantify the distinct network alterations prior to seizure onset which could differentiate the propagation patterns in a predictive manner. To accomplish this, we assessed network attributes related to various aspects of information processing within a networked system, particularly, the brain connectivity network. Specifically, we focused on three network features: modularity[47,63–65], synchronizability; and spectral radius.[49,66] While modularity has recently been utilized in characterizing the efficiency associated with integration and segregation of information across distributed brain areas, the properties of synchronizability and spectral radius remain relatively unexplored in the context of brain networks. A couple of recent studies, however, have suggested the utility of synchronizability and spectral radius in describing dynamics of seizure activity within the brain[22] and the extent of excitability of brain networks, respectively[21]. Because modularity, synchronizability, and spectral radius have been associated with different neural processes and are highly sensitive to changes in the network connectivity, we hypothesized that these measures would be powerful markers for prediction of seizure

dynamics prior to the onset. As described in the Materials and methods, each of these attributes relate to overall network architecture and their values may differ across networks with similar distribution of node degrees. Consequently, we characterized modularity, synchronizability, and spectral radius as *global* network features and, in the following, investigated how they change over time as a function of seizure propagation dynamics.

Our results revealed that the information concerning the propagation patterns of focal seizures could be decoded from these global network attributes several minutes prior to seizure onset. Specifically, the modularity preceding the onset of focal seizures with bilateral spread was higher than that of focal seizures that remain localized (resampled $P < 0.05$ for 14.75-11.75, 11.25-9.75, 9.25-8.50, 8.25-8.00, 7.75-7.50, 6.75-5.75, and 4.5-3.75 minutes before seizure onset; Fig. 4C). In addition, the synchronizability associated with focal to bilateral tonic-clonic seizures was lower than that of focal seizures that remain focal (resampled $P < 0.05$ for 14.75-13.50, 13.25-12.25, 12.00-9.50, 9.75-9.50, 8.50-8.25, 8.00-7.25, 7.00-5.75, and 5.50-3.50 minutes before seizure onset; Fig. 4D). This pattern of results was also observed in the spectral radius (resampled $P < 0.05$ for 14.75-14.50, 14.25-11.25, 11.00-11.25, 10.00-9.50, 9.25-8.75, 7.75-7.50, 7.00-5.75, 5.50-5.00, and 4.50-3.50 minutes before seizure onset; Fig. 4E). Additionally, these seizure-type dependent differences in the network modularity, synchronizability, and spectral radius re-emerged shortly after seizure onset (resampled $P < 0.05$ for 0.75-1.50 minutes, 0.75-1.75 minutes, and 0.75-1.50 minutes after seizure onset for modularity, synchronizability, and spectral radius, respectively).

Similar patterns of results were also observed between focal seizures with bilateral spread and interictal activity such that preceding the onset, the seizure networks displayed higher modularity (resampled $P < 0.05$ for 14.75-14, 13.75-12, 10.25-9.50, and 7.75-7.50 minutes before seizure onset; Supplementary Fig. 3C), lower synchronizability (resampled $P < 0.05$ for 15-13.5, 13.25-9.5, 8.75-8.25, 8-7.25, 7-6.50, and 5.25-3.75 minutes before seizure onset; Supplementary Fig. 3D), and lower spectral radius (resampled $P < 0.05$ for 14.75-14.50, 14-12.25, 11-9.25, 7.75-7.50, 5.25-4.75, and 4.50-3.75 minutes before seizure onset; Supplementary Fig. 3E). These results were accompanied by post-onset effects where focal to bilateral tonic-clonic seizures exhibited lower modularity (resampled $P < 0.05$ for 2.50-3 minutes after seizure onset; Supplementary Fig. 3C), higher synchronizability (resampled $P < 0.05$ for 1-1.50 and 2.50-3 minutes after seizure onset; Supplementary Fig. 3D), and higher spectral radius (resampled $P < 0.05$ for 1-1.50, 2.50-3, and 5.25-5.50 minutes after seizure onset; Supplementary Fig. S3E). However, focal seizures that remain localized only differed

For Review Only

from interictal activity in the measure of modularity such that the modularity of the focal seizures was lower shortly after seizure onset (resampled $P < 0.05$ for 0.75-4.50 minutes after seizure onset; Supplementary Fig. 3C). These results mimicked the trend observed in the modularity analyses of focal to bilateral tonic-clonic seizures relative to the interictal activity.

Complementary temporal reconfigurations within the functional connectivity networks sculpt seizure dynamics

Using a set of graph theoretical features, we identified reconfigurations in the functional connectivity network that characterized the propagation dynamics of different seizure types. Our results revealed that such distinguishing features can be classified into 2 groups based on the distinct and complementary temporal windows at which the differences in these features emerged as a function of seizure types. The first group of network attributes includes the global features, modularity, synchronizability, and spectral radius, which primarily captures differences between focal seizures with constrained and unconstrained dynamics *prior to* seizure onset (Fig. 5). In contrast, the second group of network properties captured differences across seizure types *after* the onset, reflecting the network reconfigurations induced by distinct propagation mechanisms. Such features include the degree per node, assortativity, clustering coefficient, and characteristic path length (Fig. 5). To further highlight the utility of these features, we evaluated both groups of network measures at a single-seizure level in 3 seizures that had similar onset regions and were recorded from an individual patient (Fig.1 and Fig. 6). Importantly, network features associated with the two seizures (seizure 1 and 3) that remained localized within the left hemisphere exhibited similar temporal patterns which differed from that of the focal seizure with bilateral spread (seizure 2). These results further suggested that network measures could potentially be used to characterize distinct neural dynamics across different types of focal seizures, even on a single-seizure basis.

For Review Only

Discussion

The goal of the present study was to investigate if the emergence of heterogeneity in seizure propagation is an outcome of mechanistically different disruptions and can be understood in terms of network-level changes within the brain before, during, and after the onset. To accomplish this, we evaluated the temporal evolution of a series of graph theoretical attributes which quantify various aspects of network organization and information processing within complex systems such as the brain. We demonstrated distinct network-level signatures that

predicted the extent of diffusion dynamics of an impending seizure as well as isolated architectural changes within the functional connectivity networks that emerged as the seizures terminated. These results advance our understanding of how heterogeneous seizure dynamics can arise from similar onset regions. Furthermore, our findings provide a rationale for utilizing network features to guide clinical diagnosis of seizure subtypes and effective intervention strategies to constrain propagation of seizure activity.

Network alterations track temporal evolution of focal seizures

We demonstrate that dynamic reconfigurations within the functional connectivity networks during evolution of focal seizures give rise to the heterogeneity observed across seizures. While past work primarily evaluated network properties of epileptic brain by averaging the signals in large discrete time windows, we assess the continuous temporal evolution of network connectivity in combination with resampling statistical tests. This formulation enables us to characterize the temporal dynamics of network alterations that underlie the emerging dynamics of seizure activity in a rigorous manner. By examining *globally defined* network features, we observe distinct macroscopic signatures which could predict the extent of diffusivity of seizure propagation minutes *prior to* the onset. Additionally, we further demonstrate that the heterogeneous dynamics exhibited across seizure types can be characterized by *post-onset* changes in local features of the functional connectivity networks such as the small-world connectivity and degree per node. Specifically, our findings revealed a relationship between the extent of small-world architecture and the diffusivity of seizure activity after the onset. Our results indicate that processes leading to the emergence of distinct seizure propagation patterns are coded in the network activity minutes prior to the onset and can have mechanistically different underpinnings. We argue that our approach provides an objective framework not only for better understanding the neural dynamics underlying evolution of seizures but also for determining whether and when a clinical intervention should be implemented to manage and control a spread of an impending seizure.

Bilateral propagation of focal seizures reflects imbalance in global integration and segregation in the connectivity brain network

Preceding the onset, we reported increased modularity along with decreased synchronizability and spectral radius in focal to bilateral tonic-clonic seizures. In light of classical accounts on

For Review Only

the mechanistic underpinnings of seizures, we argue that our findings could reflect the chemical or dynamic imbalance within the underlying networks.[6,67] Specifically, microscopic disproportion between excitation and inhibition or in the bistability of localized neural dynamics could lead to an emergence of a neural state with significantly low integration or compactness. This configuration could produce sustained high modularity along with low synchronizability and spectral radius. To regain a more balanced state, it is likely that mechanisms enhancing the connectivity between segregated networks are recruited, leading to an over-compensation which manifests as more unconstrained seizure dynamics. Future studies utilizing large-scale non-invasive neuroimaging methods could seek validation and/or refinement to this hypothesis. Notably, we found that such signatures, i.e., increased modularity as well as decreased synchronizability and spectral radius, re-emerged shortly after seizure onset and then disappeared (Fig. 4). It is, therefore, possible that these network features reflect the manifestations of the regulatory mechanisms that control reemergence of seizures. In addition, the modularity of focal seizures that remain focal increased shortly after the onset as compared to interictal activity (Supplementary Fig. 3C), further highlighting the link between local network features and control mechanisms associated with termination of seizures.

For Review Only

Time-dependent assessment of network attributes can guide development of personalized seizure treatment

The heterogeneity of epilepsy is a key confound to disease understanding and development of effective treatments. Here, we demonstrate graph theoretical features as novel biomarkers that link differential reconfigurations of the functional connectivity networks to the heterogeneity in the emerging seizure dynamics. Specifically, our investigations of the global network dynamics suggest that interventions aiming to contain the spread of seizure activity may wish to situate the brain in a topological state where the modularity is lowered, while the synchronizability and spectral radius are increased. In addition, we also show that the information regarding the propagation patterns of seizures can be decoded through the seizure-type dependent changes in the network properties several minutes before seizure onset allowing sufficient time for an intervention to be implemented. Furthermore, the seizure-type dependent signatures observed post-onset can be used to validate the efficiency of a particular treatment approach in preventing evolution of seizures and may help determine the extent of cognitive and behavioral deficits induced by the residue seizure activity in a scenario where the intervention did not completely eliminate the seizures. Future studies that wish to characterize

cognitive and behavioral changes induced by neurological disorders may also benefit from evaluating these network properties in relation to performance of patients on various test battery[17,68]. Such analyses could uncover distinct underlying pathophysiological processes that give rise to diverse cognitive and behavioral impairments across disease subtypes and across individuals, thereby improving understanding of the disease heterogeneity[69–72]. Finally, our single-seizure analyses suggest that network measures could potentially be used to characterize distinct neural dynamics across different types of seizures, even on a single-seizure basis. Such findings provide foundation for future investigation and development of effective personalized seizure treatment.

Methodological considerations and future directions

Given that the electrode placement was determined on a patient-to-patient basis by a neurologist for the purpose of identification of seizure onset zones, the data extracted from these electrodes inevitably provide an incomplete picture of the brain network due to the resulting partial coverage. In addition, the reported lack of differences between focal seizures that remain focal and interictal activity could be partially due to such spatial sampling of the recorded signals. To address this possibility, future studies may benefit from non-invasive recordings where whole-brain dynamics can be simultaneously evaluated. Further, our analyses treated multiple seizures and interictal activity segments from the same patients as independent, and primarily disregarded individual variability in seizure heterogeneity at the patient-level. This analytical choice was made based on traditional methods (e.g., see[4]), and careful statistical comparisons were implemented to identify the seizure-type dependent alteration patterns in the functional connectivity networks of seizures. To further extend our findings and improve the specificity of the interpretations, future studies may incorporate patient-level factor in their analytical frameworks.

Conclusions

In summary, by using a graph theoretical approach, we determined the extent to which distinct emerging dynamics of seizure networks were accounted for by temporal reconfigurations of the underlying functional connectivity. Collectively, our results illustrated a series of network metrics that can be utilized as quantitative biomarkers to distinguish between focal seizures of distinct dynamics based on their propagation patterns as well as the differential extent of

For Review Only

cognitive and behavioral effects accompanying the seizures. These results suggested that the networks of focal seizures with unconstrained dynamics undergo early network alterations triggering processes which facilitate the bilateral diffusion of seizure activity. The propagation-type dependent alterations in these metrics were observed again shortly after the onset, suggesting that these measures could also induce regulatory mechanisms necessary for the termination of seizures. Together, our findings provide objective means to gain better insight into the mechanisms by which seizure dynamics are regulated within the brain and provide exciting avenues where graph theoretical measures could be used to guide personalized clinical interventions.

Acknowledgements

We wish to thank Pariya Salami and Mia Borzello for assistance with data preprocessing. We are also grateful to the clinical team, technicians, and our research participants for their help in making this research possible.

Funding

This work was supported by NIH NIBIB R01EB026899-01 (T.J.S. and C.L.), NINDS R01NS104368 (T.J.S.), NIH/NINDS NS062092 (S.S.C.), Mission funding from the U.S. Army Research Laboratory (N.R., J.O.G.), and the Cooperative agreement under the U.S. Army Research Laboratory W911NF-16-2-0158 (K.B.). The views and conclusions contained in this document are those of the authors and should not be interpreted as representing the official policies, either expressed or implied, of the Army Research Laboratory or the U.S. Government.

Competing interests

The authors report no competing interests

Supplementary material

Supplementary material is available at *Brain* online.

For Review Only

References

1. Mormann F, Kreuz T, Rieke C, et al. On the predictability of epileptic seizures. *Clin Neurophysiol*. 2005;116(3):569-587.

2. Jiruska P, de Curtis M, Jefferys JGR, Schevon CA, Schiff SJ, Schindler K. Synchronization and desynchronization in epilepsy: Controversies and hypotheses. *J Physiol*. 2013;591(4):787-797.

3. Sinha N, Dauwels J, Kaiser M, et al. Predicting neurosurgical outcomes in focal epilepsy patients using computational modelling. *Brain*. 2017;140(2016):319-332.

4. Martinet LE, Kramer MA, Viles W, et al. Robust dynamic community detection with applications to human brain functional networks. *Nat Commun*. 2020;11(1):1-13.

5. Salami P, Peled N, Nadalin JK, et al. Seizure onset location shapes dynamics of initiation. *Clin Neurophysiol*. 2020;131(8):1782-1797. https://doi.org/10.1016/j.clinph.2020.04.168

6. Kuhlmann L, Lehnertz K, Richardson MP, Schelter B, Zaveri H. Seizure prediction -- ready for a new era. *Nat Rev Neurol*. 2018;14:618-630.

7. Lainscsek C, Rungratsameetaweemana N, Cash SS, Sejnowski TJ. Cortical chimera states predict epileptic seizures. *Chaos*. 2019;29(12).

8. Jirsa VK, Stacey WC, Quilichini PP, Ivanov AI, Bernard C. On the nature of seizure dynamics. *Brain*. 2014;137(8):2210-2230.

9. Saggio ML, Crisp D, Scott JM, et al. A taxonomy of seizure dynamotypes. *Elife*. 2020;9:1-56. doi:10.7554/eLife.55632

10. Li A, Chennuri B, Subramanian S, et al. Using network analysis to localize the epileptogenic zone from invasive EEG recordings in intractable focal epilepsy. *Netw Neurosci*. 2018;1(3):222-241.

11. Fisher RS, Cross JH, D'Souza C, et al. Instruction manual for the ILAE 2017 operational classification of seizure types. *Epilepsia*. 2017;58(4):531-542.

12. Fisher RS, Cross JH, French JA, et al. Operational classification of seizure types by the International League Against Epilepsy: Position Paper of the ILAE Commission for Classification and Terminology. *Epilepsia*. 2017;58(4):522-530.

For Review Only

13. Blumenfeld H, Varghese GI, Purcaro MJ, et al. Cortical and subcortical networks in human secondarily generalized tonic-clonic seizures. *Brain*. 2009;132(4):999-1012.

14. Bullmore E, Sporns O. Complex brain networks: Graph theoretical analysis of structural and functional systems. *Nat Rev Neurosci*. 2009;10(3):186-198.

15. Bassett DS, Sporns O. Network neuroscience. *Nat Neurosci*. 2017;20(3):353-364.

16. Wykes RC, Khoo HM, Caciagli L, et al. Wonoep appraisal: Network concept from an imaging perspective. 2019;60(7):1293-1305.

17. Stam CJ. Modern network science of neurological disorders. *Nat Rev Neurosci*. 2014;15(10):683-695.

18. Proix T, Bartolomei F, Guye M, Jirsa VK. Individual brain structure and modelling predict seizure propagation. *Brain*. 2017;31(3):13292-13300.

19. Ridley BGY, Rousseau C, Wirsich J, et al. Nodal approach reveals differential impact of lateralized focal epilepsies on hub reorganization. *Neuroimage*. 2015;118:39-48.

20. Garcia JO, Ashourvan A, Muldoon S, Vettel JM, Bassett DS. Applications of community detection techniques to brain graphs: Algorithmic considerations and implications for neural function. *Proc IEEE Inst Electr Electron Eng*. 2018;106(5):846-867.

21. Bansal K, Medaglia JD, Bassett DS, Vettel JM, Muldoon SF. Data-driven brain network models differentiate variability across language tasks. *PLoS Comput Biol*. 2018;14(10):1-25.

22. Khambhati AN, Davis KA, Lucas TH, Litt B, Bassett DS. Virtual Cortical Resection Reveals Push-Pull Network Control Preceding Seizure Evolution. *Neuron*. 2016;91(5):1170-1182.

23. Bassett DS, Bullmore E, Verchinski BA, Mattay VS, Weinberger DR, Meyer-Lindenberg A. Hierarchical organization of human cortical networks in health and Schizophrenia. *J Neurosci*. 2008;28(37):9239-9248.

24. Bernhardt BC, Bonilha L, Gross DW. Network analysis for a network disorder: The emerging role of graph theory in the study of epilepsy. *Epilepsy Behav*. 2015;50:162-170.

25. Stacey W, Kramer M, Gunnarsdottir K, et al. Emerging roles of network analysis for

For Review Only

epilepsy. *Epilepsy Res*. 2020;159:106255. doi:10.1016/j.eplepsyres.2019.106255

26. Braun U, Schaefer A, Betzel RF, Tost H, Meyer-Lindenberg A, Bassett DS. From maps to multi-dimensional network mechanisms of mental disorders. *Neuron*. 2018;97(1):14-31.

27. Huang LC, Wu PA, Lin SZ, Pang CY, Chen SY. Graph theory and network topological metrics may be the potential biomarker in Parkinson's disease. *J Clin Neurosci*. 2019;68:235-242.

28. Sinha N, Peternell N, Schroeder GM, et al. Focal to bilateral tonic–clonic seizures are associated with widespread network abnormality in temporal lobe epilepsy. *Epilepsia*. 2021;62(3):729-741.

29. Dauwels J, Eskandar E, Cash S. Localization of seizure onset area from intracranial non-seizure EEG by exploiting locally enhanced synchrony. *Proc 31st Annu Int Conf IEEE Eng Med Biol Soc Eng Futur Biomed EMBC 2009*. Published online 2009:2180-2183.

30. Kramer MA, Kolaczyk ED, Kirsch HE. Emergent network topology at seizure onset in humans. *Epilepsy Res*. 2008;79(2-3):173-186.

31. Antony AR, Alexopoulos A V., González-Martínez JA, et al. Functional connectivity estimated from intracranial EEG predicts surgical outcome in intractable temporal lobe epilepsy. *PLoS One*. 2013;8(10):1-7.

32. Petkov G, Goodfellow M, Richardson MP, Terry JR. A critical role for network structure in seizure onset: A computational modeling approach. *Front Neurol*. 2014;5(DEC):1-7.

33. Watts DJ, Strogatz SH. Collective dynamics of "small-world" networks. *Nature*. 1998;393(June):440-442.

34. Bassett DS, Bullmore E. Small-world brain networks. *Neuroscientist*. 2006;12(6):512-523.

35. Farahani F V., Karwowski W, Lighthall NR. Application of graph theory for identifying connectivity patterns in human brain networks: A systematic review. *Front Neurosci*. 2019;13(JUN):1-27.

36. Barahona M, Pecora LM. Synchronization in small-world systems. *Phys Rev Lett*. 2002;89(5).

37. Bullmore E, Sporns O. The economy of brain network organization. *Nat Rev Neurosci*.

For Review Only

2012;13(5):336-349.

38. Onnela JP, Saramäki J, Kertész J, Kaski K. Intensity and coherence of motifs in weighted complex networks. *Phys Rev E - Stat Nonlinear, Soft Matter Phys*. 2005;71(6).

39. Rubinov M, Sporns O. Complex network measures of brain connectivity: Uses and interpretations. *Neuroimage*. 2010;52(3):1059-1069.

40. Reijneveld JC, Ponten SC, Berendse HW, Stam CJ. The application of graph theoretical analysis to complex networks in the brain. *Clin Neurophysiol*. 2007;118(11):2317-2331.

41. Newman MEJ. Assortative Mixing in Networks. *Phys Rev Lett*. 2002;89(20):1-4.

42. Lim S, Radicchi F, van den Heuvel MP, Sporns O. Discordant attributes of structural and functional brain connectivity in a two-layer multiplex network. *Sci Rep*. 2019;9(1):1-13.

43. de Haan W, Pijnenburg YAL, Strijers RLM, et al. Functional neural network analysis in frontotemporal dementia and Alzheimer's disease using EEG and graph theory. *BMC Neurosci*. 2009;10:1-12.

44. Vaessen MJ, Braakman HMH, Heerink JS, et al. Abnormal modular organization of functional networks in cognitively impaired children with frontal lobe epilepsy. *Cereb Cortex*. 2013;23(8):1997-2006.

45. Godwin D, Barry RL, Marois R. Breakdown of the brain's functional network modularity with awareness. *Proc Natl Acad Sci U S A*. 2015;112(12):3799-3804.

46. Reichardt J, Bornholdt S. Statistical mechanics of community detection. *Phys Rev E - Stat Nonlinear, Soft Matter Phys*. 2006;74(1):1-14.

47. Newman MEJ. Modularity and community structure in networks. *Proc Natl Acad Sci U S A*. 2006;103(23):8577-8582.

48. Restrepo JG, Ott E, Hunt BR. Onset of synchronization in large networks of coupled oscillators. *Phys Rev E - Stat Nonlinear, Soft Matter Phys*. 2005;71(3):1-12.

49. Meghanathan N. Spectral Radius as a Measure of Variation in Node Degree for Complex Network Graphs. *Proc - 7th Int Conf u- e-Service, Sci Technol UNESST 2014*. Published online 2015:30-33.

50. Jamakovic A, Kooij RE, Van Mieghem P, Van Dam ER. Robustness of networks against viruses: The role of the spectral radius. *Proc - 2006 Symp Commun Veh Technol IEEE*

For Review Only

SCVT 2006; 13th Annu Symp Commun Veh Technol Benelux. 2006;(3):35-38.

51. Wang Y, Chakrabarti D, Wang C, Faloutsos C. Epidemic spreading in real networks: an eigenvalue viewpoint. *22nd Symp Reliab Distrib Comput Florence, Italy*. Published online 2003.

52. van Dam ER, Kooij RE. The minimal spectral radius of graphs with a given diameter. *Linear Algebra Appl*. 2007;423(2-3):408-419.

53. Wang R, Zhang ZZ, Ma J, Yang Y, Lin P, Wu Y. Spectral properties of the temporal evolution of brain network structure. *Chaos*. 2015;25(12). doi:10.1063/1.4937451

54. Ponten SC, Bartolomei F, Stam CJ. Small-world networks and epilepsy: Graph theoretical analysis of intracerebrally recorded mesial temporal lobe seizures. *Clin Neurophysiol*. 2007;118(4):918-927.

55. Kramer MA, Eden UT, Kolaczyk ED, Zepeda R, Eskandar EN, Cash SS. Coalescence and fragmentation of cortical networks during focal seizures. *J Neurosci*. 2010;30(30):10076-10085.

56. Kramer MA, Cash SS. Epilepsy as a disorder of cortical network organization. *Neuroscientist*. 2012;18(4):360-372.

57. Jenssen S, Gracely EJ, Sperling MR. How long do most seizures last? A systematic comparison of seizures recorded in the epilepsy monitoring unit. *Epilepsia*. 2006;47(9):1499-1503.

58. Noldus R, Mieghem P Van. Assortativity in complex networks. *J Complex Networks*. 2014;3(4):507-542.

59. Sone D, Matsuda H, Ota M, et al. Graph theoretical analysis of structural neuroimaging in temporal lobe epilepsy with and without psychosis. *PLoS One*. 2016;11(7).

60. Berg AT, Berkovic SF, Brodie MJ, et al. Revised terminology and concepts for organization of seizures and epilepsies: Report of the ILAE Commission on Classification and Terminology, 2005-2009. *Epilepsia*. 2010;51(4):676-685.

61. Helmstaedter C, Witt JA. Epilepsy and cognition – A bidirectional relationship? *Seizure*. 2017;49:83-89.

62. Garcia-Ramos C, Lin JJ, Kellermann TS, Bonilha L, Prabhakaran V, Hermann BP. Graph theory and cognition: A complementary avenue for examining

For Review Only

neuropsychological status in epilepsy. *Epilepsy Behav*. 2016;64:329-335.

63. Fukushima M, Betzel RF, He Y, et al. Fluctuations between high- and low-modularity topology in time-resolved functional connectivity. *Neuroimage*. 2018;180(July 2017):406-416.

64. Chavez M, Valencia M, Navarro V, Latora V, Martinerie J. Functional modularity of background activities in normal and epileptic brain networks. *Phys Rev Lett*. 2010;104(11).

65. Goulas A, Betzel RF, Hilgetag CC. Spatiotemporal ontogeny of brain wiring. *Sci Adv*. 2019;5(6).

66. Chen H, Zhao X, Liu F, Xu S, Lu W. Optimizing interconnections to maximize the spectral radius of interdependent networks. *Phys Rev E*. 2017;95(3):1-15.

67. Blumenfeld H. Cellular and network mechanisms of electrographic seizures. *Epilepsia*. 2005;46(9):21-33.

68. Xia CH, Ma Z, Ciric R, et al. Linked dimensions of psychopathology and connectivity in functional brain networks. *Nat Commun*. 2018;9(1):1-14.

69. Satterthwaite TD, Feczko E, Kaczkurkin AN, Fair DA. Parsing Psychiatric Heterogeneity Through Common and Unique Circuit-Level Deficits. *Biol Psychiatry*. 2020;88(1):4-5.

70. Cornblath EJ, Li HL, Changolkar L, et al. Computational modeling of tau pathology spread reveals patterns of regional vulnerability and the impact of a genetic risk factor. 2021;(June):1-16.

71. Frankle WG, Narendran R. Distinguishing Schizophrenia Subtypes: Can Dopamine Imaging Improve the Signal-to-Noise Ratio? *Biol Psychiatry*. 2020;87(3):197-199.

72. Karalunas SL, Nigg JT. Heterogeneity and Subtyping in Attention-Deficit/Hyperactivity Disorder—Considerations for Emerging Research Using Person-Centered Computational Approaches. *Biol Psychiatry*. 2020;88(1):103-110.

For Review Only

Figure legends

Figure 1 Emergence of distinct seizure propagation patterns in a single patient. (**A**) During a clinical monitoring procedure to identify a seizure onset zone of patients with medication-refractory (drug-resistant) epilepsy, intracranial recording electrodes are implanted. (**B**) Intracranial activity during two sample seizures recorded from a single patient, which exhibit distinct propagation dynamics. On the left, the seizure activity originates from a few electrodes and persists in the localized area within a single hemisphere (i.e., focal seizure that remains focal). On the right, the seizure activity originates from a few electrodes but diffuses bilaterally to involve electrodes in both hemispheres. This type of seizure is known as focal to bilateral tonic-clonic seizure or focal seizure with bilateral spread. Despite their similarly focal origin, these seizure types induce drastically differential clinical manifestations such that focal to bilateral tonic-clonic seizures are associated with more severe cognitive and behavioral deficits. We hypothesize that such heterogeneity in seizure dynamics emerges from distinct and measurable temporal alterations in the functional brain connectivity networks.

Figure 2 Schematic of graph theoretical analysis of functional brain dynamics. (**A**) Locations of implanted intracranial electrodes of a sample patient. (**B**) We use electrocorticography (ECoG) time-series data from all intracranial electrodes from each patient recorded during a clinical monitoring procedure to locate the seizure onset zone. We estimate the instantaneous functional connectivity of the underlying brain network by computing pairwise correlations of ECoG data across electrodes in a sliding-window manner. The magnitudes of these correlations (restricted between 0 and 1) reflect the strength of connections between each pair of electrodes and are represented by a weighted adjacency or connectivity matrix (see Materials and methods). (**C**) To investigate time-varying changes in the functional brain connectivity during temporal evolution of each seizure type, we compute a series of connectivity matrices over time and use these as bases to construct functional connectivity networks. (**D**) A schematic of sample constructed networks, consisting of nodes (electrodes) and edges (connection strength). To quantify alterations within these complex networks over time, we evaluate changes of a series of graph theoretical attributes which describe globally- and locally- defined properties of the constructed networks.

Figure 3 Small-world architecture tracks diffusivity of seizure activity. Focal to bilateral tonic-clonic seizures (n = 18) display more prominent small-world connectivity (simultaneous

For Review Only

increase in the clustering coefficient and decrease in the characteristic path length) than focal seizures that remain localized within one hemisphere (n = 49). (**A**) Averages of the clustering coefficients associated with each seizure type are plotted separately for preictal, ictal (during seizure), and postictal periods. The clustering coefficient of interictal (seizure-free) networks are also plotted as a baseline. (**B**) The characteristic path length is plotted in the same manner. (**C**) The clustering coefficient of focal to bilateral tonic-clonic seizures is higher than that of focal seizures that remain focal, 2-10 minutes after seizure onset. (**D**) The characteristic path length of focal to bilateral tonic-clonic seizures is lower than that of focal seizures that remain focal, 2-10 minutes after seizure onset. Error bars indicate 95% CIs computed by resampling the data distributions. Solid bars show resampled $P < 0.05$.

Figure 4 Various features of functional connectivity display distinct temporal changes as a function of seizure propagation dynamics. Left panels illustrate a series of graph theoretical measures computed from networks of focal seizures that remain localized (n = 49) and from networks of focal to bilateral tonic-clonic seizures (n = 18). The time-varying differences observed in each of these features as a function of seizure types are plotted in the corresponding right panels. (**A**) The degree per node of focal to bilateral tonic-clonic seizures is higher than that of focal seizures that remain focal, 1.75-10 minutes after seizure onset. (**B**) The assortativity, a measure of network robustness, is lower for focal to bilateral tonic-clonic seizures relative to focal seizures that remain focal, 7.5-9.5 minutes after seizure onset. (**C**) The modularity, which captures efficient network integration and global segregation, is higher for focal to bilateral tonic-clonic seizures when compared to focal seizures that remain focal during temporal windows between 14.75 to 3.75 minutes before seizure onset and 0.75-1.50 minutes after the onset. (**D**) The synchronizability, which estimates the propensity of information to diffuse in a network, is higher for focal to bilateral tonic-clonic seizures relative to focal seizures that remain focal during temporal windows between 14.75 to 3.50 minutes before seizure onset and 0.75-1.75 minutes after seizure onset. (**E**) The spectral radius, which relates to the global spread of synchronization in a network, is also higher for focal to bilateral tonic-clonic seizures as compared to focal seizures that remain focal during temporal windows between14.75 to 3.50 minutes before seizure onset and 0.75-1.50 minutes after the onset. Error bars indicated 95% CIs computed by resampling the data distribution. Solid bars show resampled $P < 0.05$.

Figure 5 Summary of graph theoretical attributes probed across seizure types. The network features investigated can be categorized into 2 groups based on the temporal windows

For Review Only

at which differential changes in these features emerge as a function of seizure propagation patterns. The time windows where such differences are observed are plotted separately for each of the network measures (resampled $P < 0.05$). Global features, i.e., the modularity, synchronizability, and spectral radius, primarily capture network alterations that occur prior to and shortly after seizure onset. In contrast, the degree per node, assortativity, clustering coefficient and characteristic path length characterize post-onset network reconfigurations induced by different types of propagation dynamics.

Figure 6 Distinct patterns of network properties across seizure types at a single-seizure level. Graph theoretical measures extracted from networks associated with three seizures that share similar onset regions recorded from a sample patient. Seizure 1 and seizure 3 are categorized by an epileptologist as focal seizures that remain focal (sample recordings of seizure 1 is illustrated in Fig. 1B (left)), whereas seizure 2 is categorized as a focal to bilateral tonic-clonic seizure (sample recordings of seizure 2 is also illustrated in Fig. 1B (right)). Seizure 1 (top panel) and seizure 3 (bottom panel) exhibit similar patterns of topological properties, which differ from the features corresponding to seizure 2 (middle panel).

For Review Only

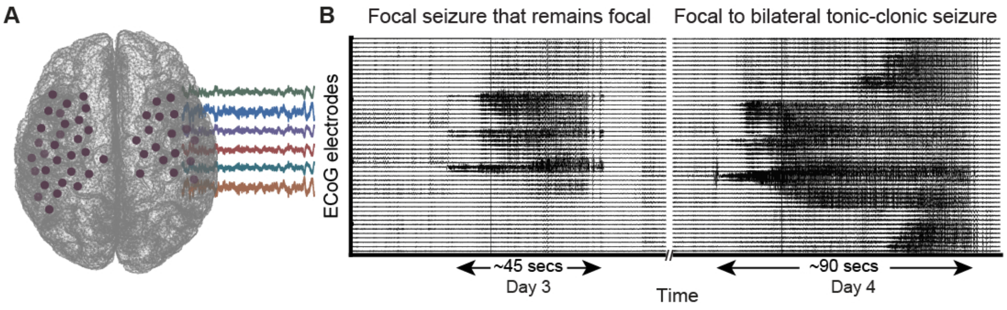

Figure 1 Emergence of distinct seizure propagation patterns in a single patient. (A) During a clinical monitoring procedure to identify a seizure onset zone of patients with medication-refractory (drug-resistant) epilepsy, intracranial recording electrodes are implanted. (B) Intracranial activity during two sample seizures recorded from a single patient, which exhibit distinct propagation dynamics. On the left, the seizure activity originates from a few electrodes and persists in the localized area within a single hemisphere (i.e., focal seizure that remains focal). On the right, the seizure activity originates from a few electrodes but diffuses bilaterally to involve electrodes in both hemispheres. This type of seizure is known as focal to bilateral tonic-clonic seizure or focal seizure with bilateral spread. Despite their similarly focal origin, these seizure types induce drastically differential clinical manifestations such that focal to bilateral tonic-clonic seizures are associated with more severe cognitive and behavioral deficits. We hypothesize that such heterogeneity in seizure dynamics emerges from distinct and measurable temporal alterations in the functional brain connectivity networks.

469x148mm (59 x 59 DPI)

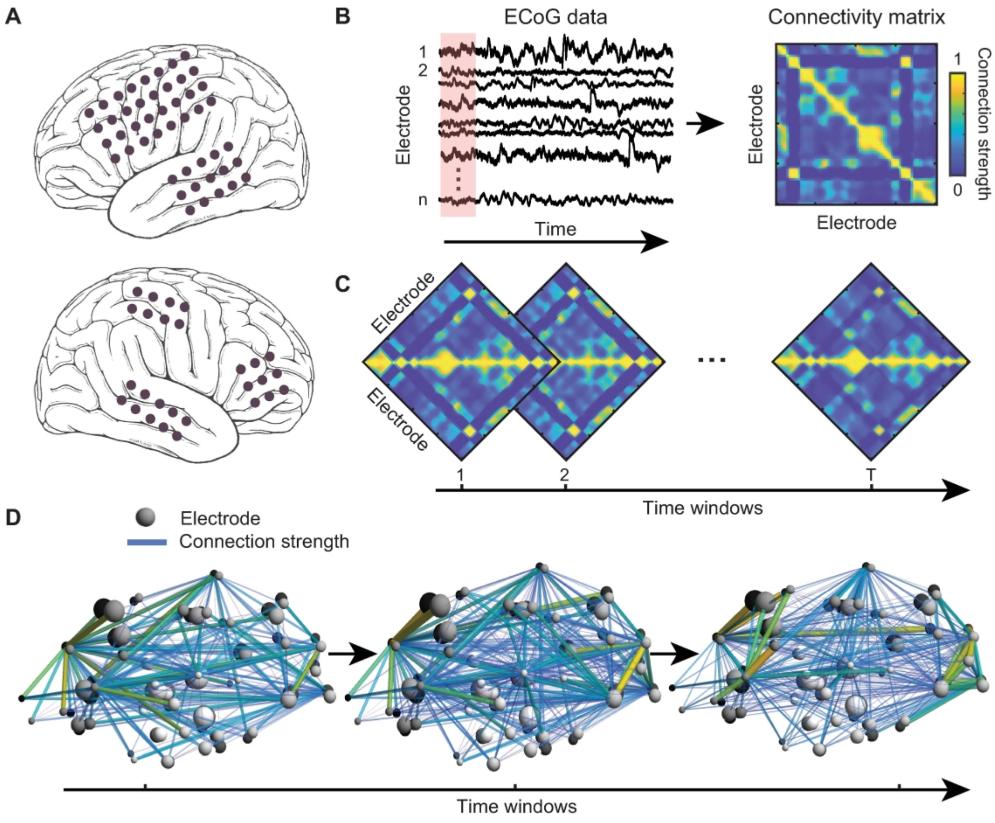

Figure 2 Schematic of graph theoretical analysis of functional brain dynamics. (A) Locations of implanted intracranial electrodes of a sample patient. (B) We use electrocorticography (ECoG) time-series data from all intracranial electrodes from each patient recorded during a clinical monitoring procedure to locate the seizure onset zone. We estimate the instantaneous functional connectivity of the underlying brain network by computing pairwise correlations of ECoG data across electrodes in a sliding-window manner. The magnitudes of these correlations (restricted between 0 and 1) reflect the strength of connections between each pair of electrodes and are represented by a weighted adjacency or connectivity matrix (see Materials and methods). (C) To investigate time-varying changes in the functional brain connectivity during temporal evolution of each seizure type, we compute a series of connectivity matrices over time and use these as bases to construct functional connectivity networks. (D) A schematic of sample constructed networks, consisting of nodes (electrodes) and edges (connection strength). To quantify alterations within these complex networks over time, we evaluate changes of a series of graph theoretical attributes which describe globally- and locally- defined properties of the constructed networks.

184x151mm (600 x 600 DPI)

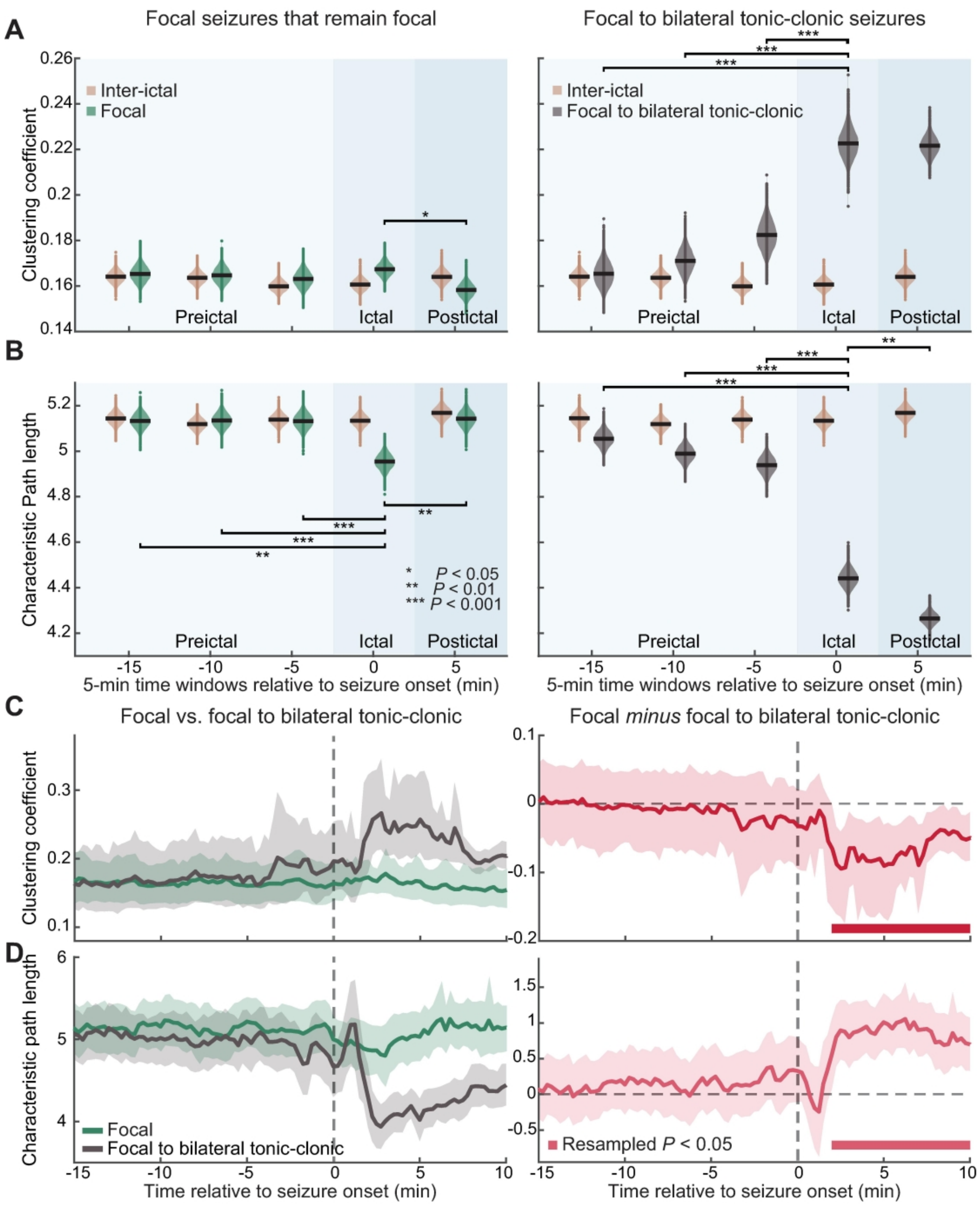

Figure 3 Small-world architecture tracks diffusivity of seizure activity. Focal to bilateral tonic-clonic seizures (n = 18) display more prominent small-world connectivity (simultaneous increase in the clustering coefficient and decrease in the characteristic path length) than focal seizures that remain localized within one hemisphere (n = 49). (A) Averages of the clustering coefficients associated with each seizure type are plotted separately for preictal, ictal (during seizure), and postictal periods. The clustering coefficient of interictal (seizure-free) networks are also plotted as a baseline. (B) The characteristic path length is plotted in the same manner. (C) The clustering coefficient of focal to bilateral tonic-clonic seizures is higher than that of focal seizures that remain focal, 2-10 minutes after seizure onset. (D) The characteristic path length of focal to bilateral tonic-clonic seizures is lower than that of focal seizures that remain focal, 2-10 minutes after seizure onset. Error bars indicate 95% CIs computed by resampling the data distributions. Solid bars show resampled P < 0.05.

182x224mm (600 x 600 DPI)

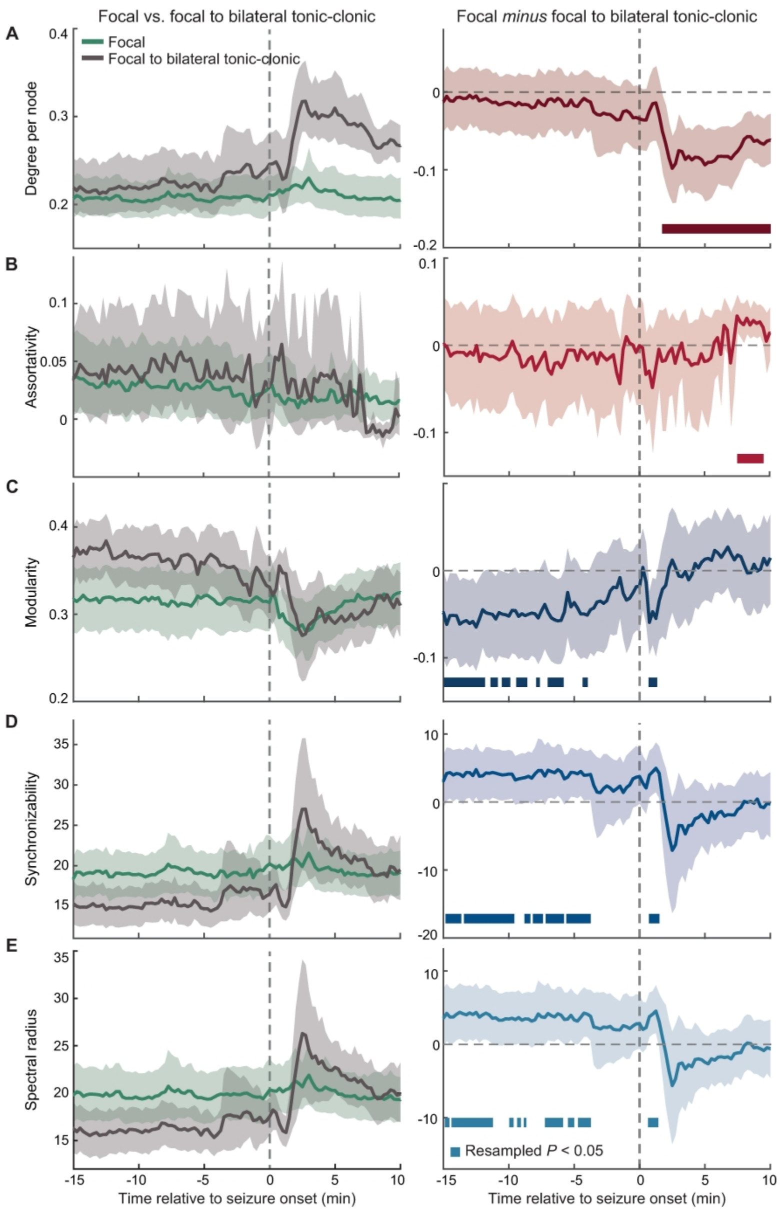

Figure 4 Various features of functional connectivity display distinct temporal changes as a function of seizure propagation dynamics. Left panels illustrate a series of graph theoretical measures computed from networks of focal seizures that remain localized (n = 49) and from networks of focal to bilateral tonic-clonic seizures (n = 18). The time-varying differences observed in each of these features as a function of seizure types are plotted in the corresponding right panels. (A) The degree per node of focal to bilateral tonic-clonic seizures is higher than that of focal seizures that remain focal, 1.75-10 minutes after seizure onset. (B) The assortativity, a measure of network robustness, is lower for focal to bilateral tonic-clonic seizures relative to focal seizures that remain focal, 7.5-9.5 minutes after seizure onset. (C) The modularity, which captures efficient network integration and global segregation, is higher for focal to bilateral tonic-clonic seizures when compared to focal seizures that remain focal during temporal windows between 14.75 to 3.75 minutes before seizure onset and 0.75-1.50 minutes after the onset. (D) The synchronizability, which estimates the propensity of information to diffuse in a network, is higher for focal to bilateral tonic-clonic seizures relative to focal seizures that remain focal during temporal windows between 14.75 to 3.50 minutes before seizure onset and 0.75-1.75 minutes after seizure onset. (E) The spectral radius, which relates to the global spread

of synchronization in a network, is also higher for focal to bilateral tonic-clonic seizures as compared to focal seizures that remain focal during temporal windows between14.75 to 3.50 minutes before seizure onset and 0.75-1.50 minutes after the onset. Error bars indicated 95% CIs computed by resampling the data distribution. Solid bars show resampled P < 0.05.

178x277mm (600 x 600 DPI)

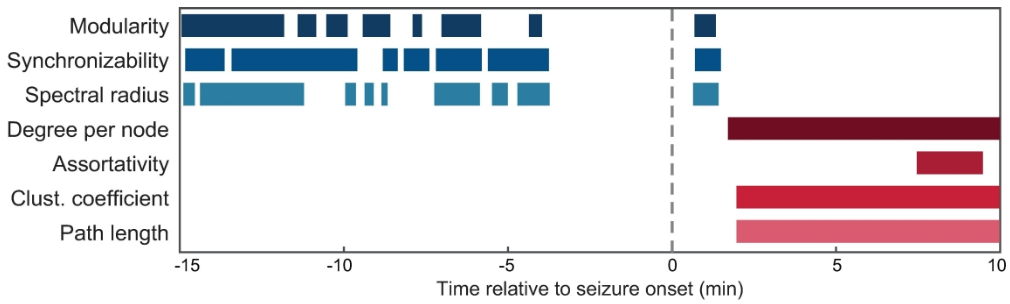

Figure 5 Summary of graph theoretical attributes probed across seizure types. The network features investigated can be categorized into 2 groups based on the temporal windows at which differential changes in these features emerge as a function of seizure propagation patterns. The time windows where such differences are observed are plotted separately for each of the network measures (resampled $P < 0.05$). Global features, i.e., the modularity, synchronizability, and spectral radius, primarily capture network alterations that occur prior to and shortly after seizure onset. In contrast, the degree per node, assortativity, clustering coefficient and characteristic path length characterize post-onset network reconfigurations induced by different types of propagation dynamics.

183x53mm (600 x 600 DPI)

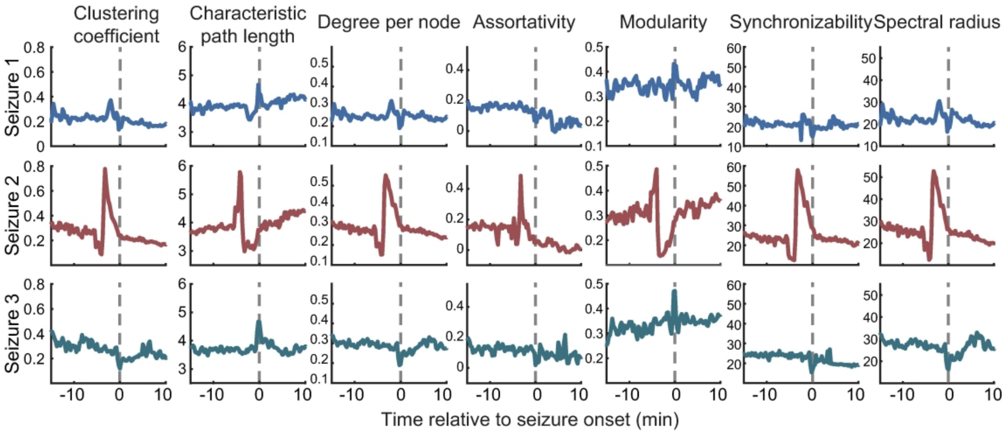

Figure 6 Distinct patterns of network properties across seizure types at a single-seizure level. Graph theoretical measures extracted from networks associated with three seizures that share similar onset regions recorded from a sample patient. Seizure 1 and seizure 3 are categorized by an epileptologist as focal seizures that remain focal (sample recordings of seizure 1 is illustrated in Fig. 1B (left)), whereas seizure 2 is categorized as a focal to bilateral tonic-clonic seizure (sample recordings of seizure 2 is also illustrated in Fig. 1B (right)). Seizure 1 (top panel) and seizure 3 (bottom panel) exhibit similar patterns of topological properties, which differ from the features corresponding to seizure 2 (middle panel).

184x79mm (600 x 600 DPI)

Brain network dynamics codify heterogeneity in seizure evolution

Nuttida Rungratsameetaweemana, Claudia Lainscsek, Sydney S. Cash, Javier O. Garcia, Terrence J. Sejnowski[†], Kanika Bansal[*,†]

Table I Patient Profiles

Patient	Sex	Age at onset/ surgery	Etiology	Seizure Type (#)	Seizure Onset Zone	Resection Areas	Outcome
1	F	15/46	Dysplasia	focal to bilateral (5)	Anterior temporal	Right anterior temporal	I
2	F	42/55	n.a	focal to bilateral (3)	Temporal	None	I
3	F	17/45	n.a	focal (1); focal to bilateral (2)	Temporal	None	n.a
4	M	8/23	n.a	focal (10)	Frontal	None	III
5	M	14/35	n.a	focal (9); focal to bilateral (2)	Temporal	Right anterior temporal	n.a
6	F	12/32	n.a	focal (15)	Temporal	Right anterior temporal	II
7	F	7/23	n.a	focal (6)	Frontal	Left frontal	IV
8	F	10/27	n.a	focal (1)	Unknown	Left frontal	IV
9	F	8/19	MTS	focal (1)	Anterior temporal	Left anterior temporal	III
10	F	14/31	n.a	focal to bilateral (2)	Temporal	Right anterior temporal	I
11	F	1/21	Stroke	focal (2)	Temporal	Left temporal	IV
12	F	9/42	n.a	focal (2)	Frontal	None	II
13	M	39/47	n.a	focal to bilateral (3)	Posterior temporal	Right temporal	I
14	F	50/59	n.a	focal (2); focal to bilateral (1)	Posterior temporal	Left temporal	I

For Review Only

Clinical characteristics of the patients. For each patient, we report sex, age at first reported seizures onset, as well as age at the monitoring phase and surgery. We also report the seizure etiology, which was clinically determined through medical history, imaging, and long-term invasive monitoring. Additionally, we indicate the number of observed seizures associated with the two different types of seizures which originated from one hemisphere: focal seizures that remained localized within the same hemisphere (focal seizure; focal) and focal seizures that propagate bilaterally to both hemispheres (focal to bilateral tonic-clonic seizure; focal to bilateral). Surgical outcome (outcome) was based on Engel score: seizure freedom to no improvement (I-V), and no follow-up (NF). Legend: M = male; F = female; MTS = Mesial Temporal Sclerosis; n.a = not applicable.

For Review Only

Brain network dynamics codify heterogeneity in seizure evolution

Nuttida Rungratsameetaweemana, Claudia Lainscsek, Sydney S. Cash, Javier O. Garcia, Terrence J. Sejnowski†, Kanika Bansal*,†

Supplementary Figures

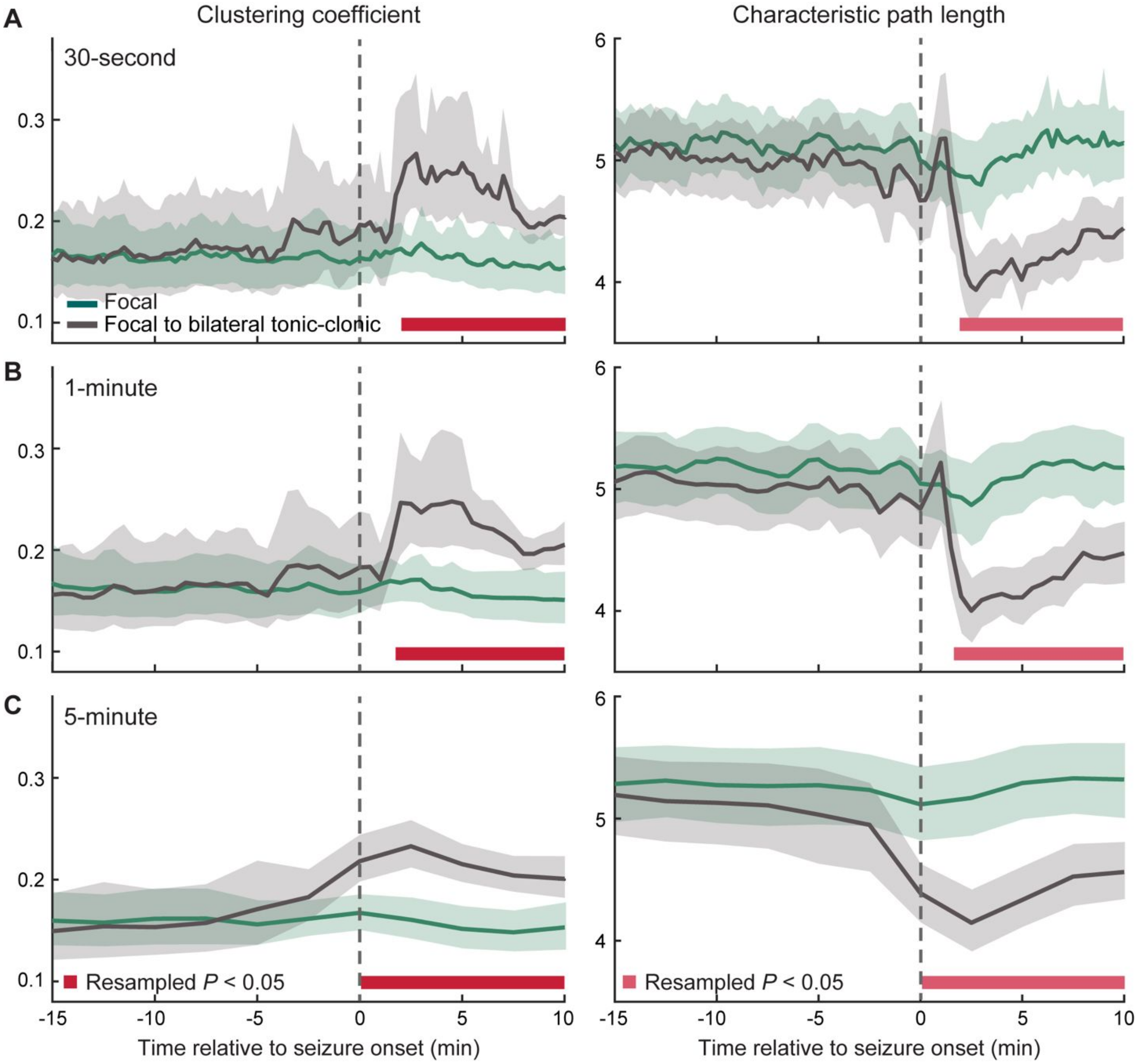

Fig. S1. The effects of temporal smoothing on sample network measures. Prior to computing graph theoretical features, a temporal smoothing parameter is first applied to the functional connectivity networks to approximate stationary pairwise connection strength signals. (**A**) The clustering coefficient and characteristic path length as computed after a smoothing parameter of 30 seconds has been applied to the adjacency matrices in a time-resolved fashion. These network measures are calculated separately for focal seizures that remain focal (n = 49) and focal to bilateral tonic-clonic seizures (n = 18). (**B**) Same network measures computed based on a temporal smoothing parameter of 1 minute. (**C**) Same network measures computed based on a temporal smoothing parameter of 5 minutes. The clustering coefficient of focal to bilateral tonic-clonic seizures are higher than that of focal seizures that remain focal (2-10 minutes; 2-10 minutes; seizure onset to 10 minutes after seizure onset for a smoothing parameter of 30 seconds, 1 minute, and 5 minutes, respectively). The characteristic path length of focal to bilateral tonic-clonic seizures are lower than that of focal seizures that remain focal (2-10 minutes; 2-10 minutes; seizure onset to 10 minutes after seizure onset for a smoothing parameter of 30 seconds, 1 minute, and 5 minutes, respectively). Error bars indicate 95% CIs computed by resampling the data distribution. Solid bars show resampled $P < 0.05$. All reported results in the main text are achieved based on a smoothing parameter of 30 seconds and the comparisons illustrated here suggest that our reported findings are robust and largely unaffected by the choice of this parameter.

For Review Only

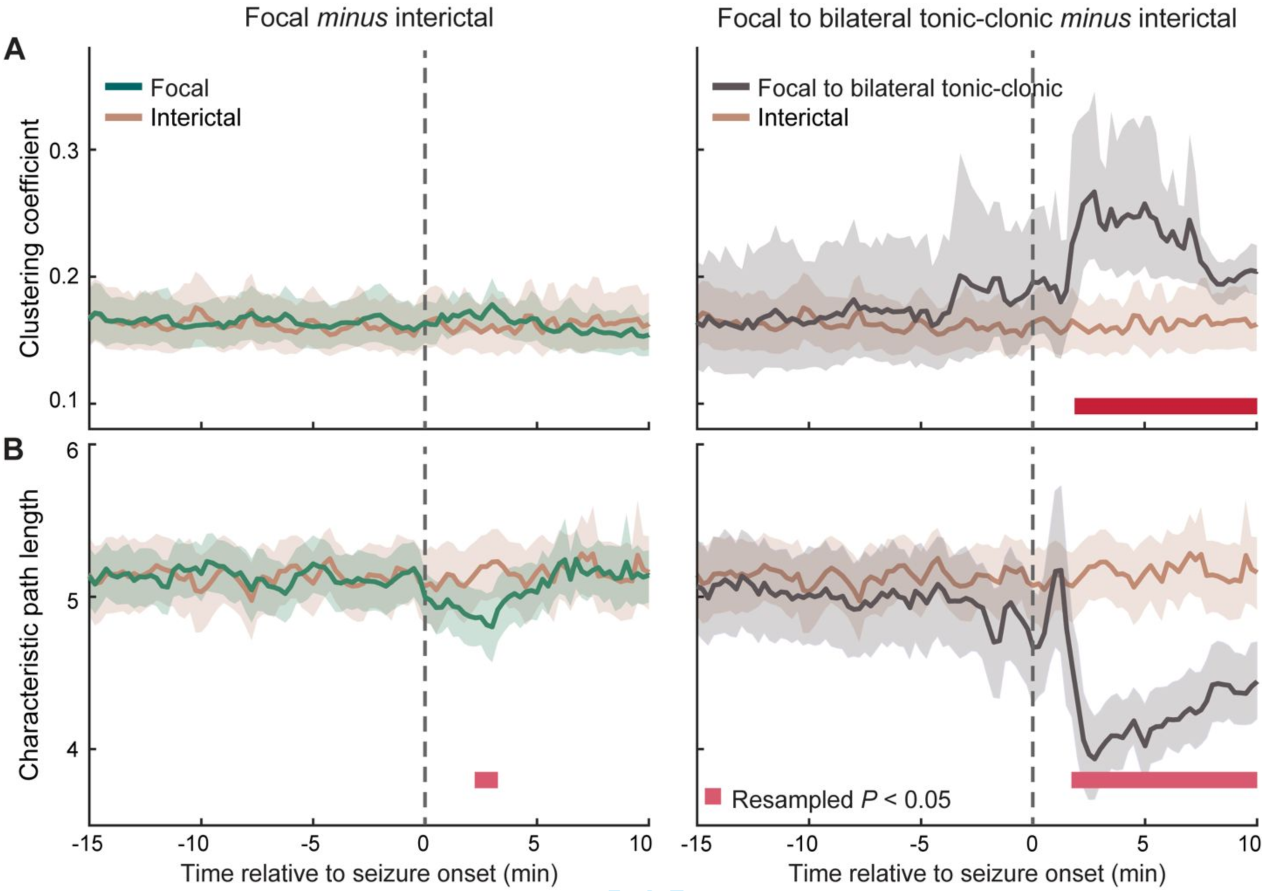

Fig. S2. Small-world properties of focal seizures with constrained and unconstrained propagation dynamics relative to those of interictal periods. (**A**) The clustering coefficient of focal seizures that remain focal ($n = 49$) is not different from that of interictal networks ($n = 49$). The clustering coefficient of focal to bilateral tonic-clonic seizures ($n = 18$) is higher than that of interictal networks, 2-10 minutes after seizure onset. (**B**) The characteristic path length of focal seizures that remain focal is lower than that of interictal networks, 2.50-3.50 minutes after seizure onset. The characteristic path length of focal to bilateral tonic-clonic seizures is lower than that of interictal networks, 1.75-10 minutes after seizure onset. Error bars indicate 95% CIs computed by resampling the data distribution. Solid bars show resampled $P < 0.05$.

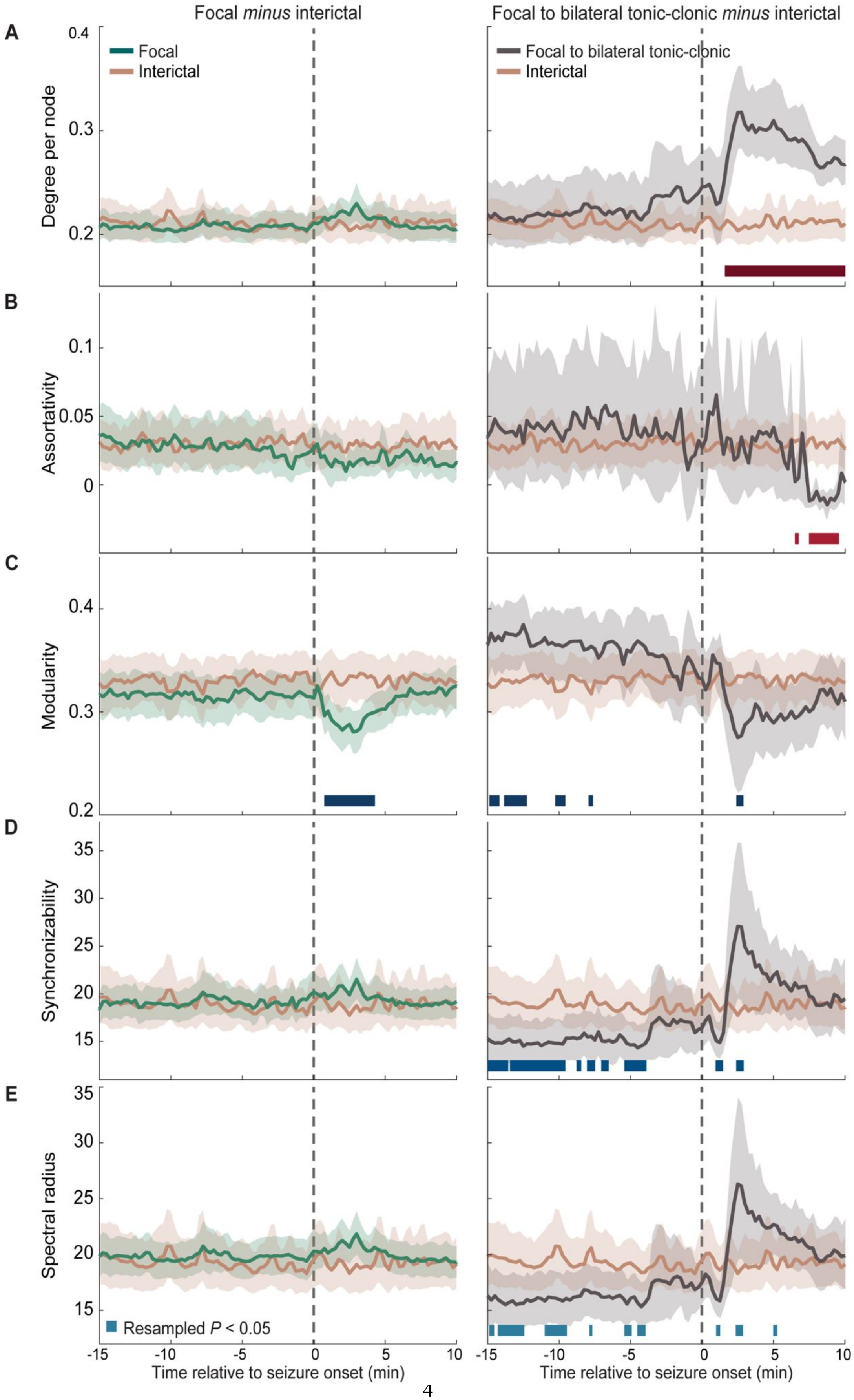

Focal *minus* interictal
Focal to bilateral tonic-clonic *minus* interictal
A
B
C
D
E
Focal
Interictal
Focal to bilateral tonic-clonic
Interictal
Degree per node
Assortativity
Modularity
Synchronizability
Spectral radius
Resampled *P* < 0.05
Time relative to seizure onset (min)
Time relative to seizure onset (min)

Fig. S3. Network features of focal seizure with constrained and unconstrained dynamics relative to that of interictal periods. (A) The degree per node of focal seizures that remain focal (n = 49) is not different from that of interictal networks (n = 49). The degree per of focal to bilateral tonic-clonic seizures (n = 18) is higher than that of interictal networks, 1.75-10 minutes after seizure onset. **(B)** The assortativity of focal seizures that remain focal is not different from that of inter-ictal networks. The assortativity of focal to bilateral tonic-clonic seizures is higher than that of interictal networks, 6.50-7 and 7.50-9.75 minutes after seizure onset. **(C)** The modularity of focal seizures that remain focal is lower than that of inter-ictal networks, 0.75-4.50 minutes after seizure onset. The modularity of focal to bilateral tonic-clonic seizures is higher than that of interictal networks, 14.75-14, 13.75-12, 10.25-9.50, and 7.75-7.50 minutes before seizure onset; and 2.50-3 minutes after onset. **(D)** The synchronizability of focal seizures that remain focal is not different from that of inter-ictal networks. The synchronizability focal to bilateral tonic-clonic seizures is lower than that of interictal networks, 15-13.5, 13.25-9.5, 8.75-8.25, 8-7.25, 7-6.50, and 5.25-3.75 minutes before seizure onset; and 1-1.5, and 2.50-3 minutes after seizure onset. **(E)** The spectral radius of focal seizures that remain focal is not different from that of interictal networks. The spectral radius of focal to bilateral tonic-clonic seizures is lower than that of interictal networks, 14.75-14.50, 14-12.25, 11-9.25, 7.75-7.50, 5.25-4.75, and 4.50-3.75 minutes before seizure onset; and 1-1.50, 2.50-3, and 5.25-5.50 minutes after seizure onset. Error bars indicate 95% CIs computed by resampling the data distribution. Solid bars show resampled $P < 0.05$.

For Review Only

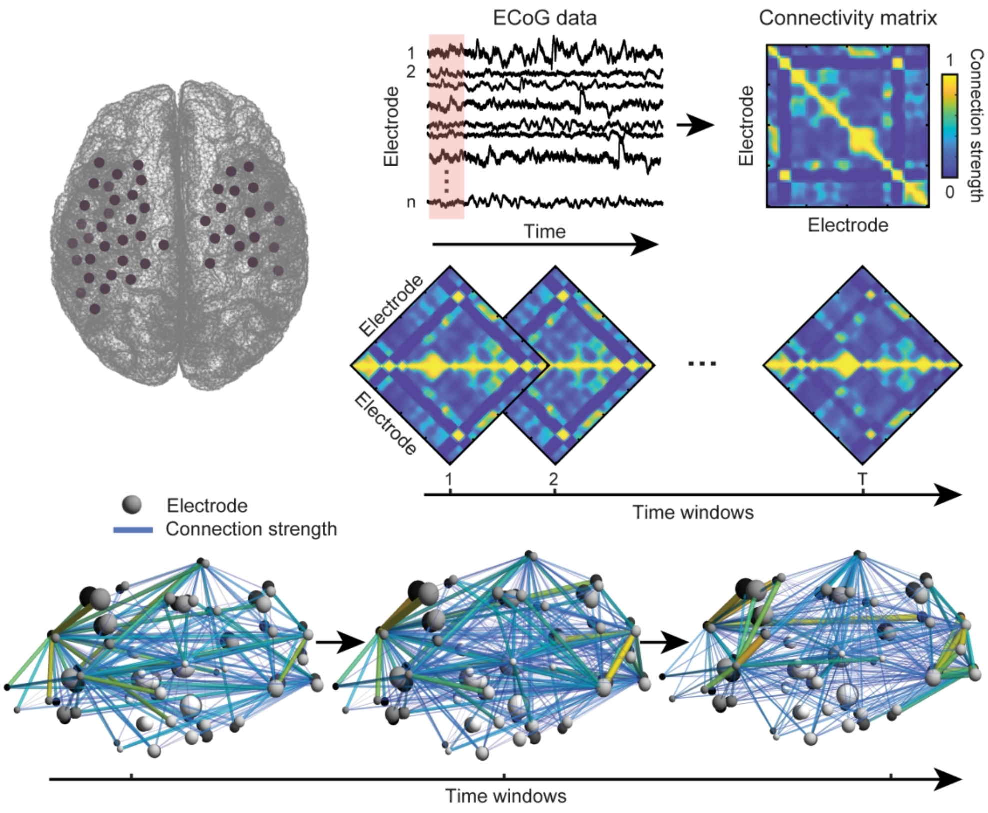

466x377mm (118 x 118 DPI)
