# Supplementary material for: Brain network dynamics codify heterogeneity in seizure evolution
Source: Brain Commun. 2022 Sep 16;4(5):fcac234. doi: 10.1093/braincomms/fcac234 (PMC9527667; doi:10.1093/braincomms/fcac234)
Supplement: fcac234_Supplementary_Data [file fcac234_supplementary_data.zip › Manuscript_revision_1.pdf]

OXFORD UNIVERSITY PRESS | Brain Communications

Brain network dynamics codify heterogeneity in seizure evolution

Journal:	*Brain Communications*
Manuscript ID	BRAINCOM-2021-255.R1
Manuscript Type:	Original Article
Date Submitted by the Author:	24-Feb-2022
Complete List of Authors:	Rungratsameetaweemana, Nuttida ; US Army Research Laboratory Human Research and Engineering Directorate; Salk Institute for Biological Studies Computational Neurobiology Laboratory; King Mongkut's University of Technology Thonburi, Bangkok, Neuroscience Center for Research and Innovation<br>Lainscsek, Claudia ; Salk Institute for Biological Studies Computational Neurobiology Laboratory<br>Cash, Sydney; Massachusetts General Hospital Department of Neurology, Neurology<br>Garcia, Javier; US Army Research Laboratory Human Research and Engineering Directorate<br>Sejnowski, Terrence ; Salk Institute for Biological Studies Computational Neurobiology Laboratory<br>Bansal, Kanika; Columbia University, ; US Army Research Laboratory Human Research and Engineering Directorate,
Keywords:	epilepsy, functional connectivity, seizure propagation, brain networks

SCHOLARONE™
Manuscripts

Brain network dynamics codify heterogeneity in seizure evolution

Nuttida Rungratsameetaweemana[1,2,3], Claudia Lainscsek[2,4], Sydney S. Cash[5],

Javier O. Garcia[1], Terrence J. Sejnowski[2,4,6,†], Kanika Bansal[1,7,†]

[†]**Senior authors.**

For Review Only

Abstract

Dynamic functional brain connectivity facilitates adaptive cognition and behavior. Abnormal alterations within such connectivity could result in disrupted functions observed across various neurological conditions. As one of the most common neurological disorders, epilepsy is defined by the seemingly random occurrence of spontaneous seizures. A central but unresolved question concerns the mechanisms by which extraordinarily diverse propagation dynamics of seizures emerge.

Here, we applied a graph theoretical approach to assess dynamic reconfigurations in the functional brain connectivity before, during, and after seizures that display heterogeneous propagation patterns despite sharing similar cortical onsets. We computed time-varying functional brain connectivity networks from human intracranial recordings of 67 seizures (across 14 patients) that had a focal origin — 49 of these focal seizures remained focal and 18 underwent a bilateral spread (focal to bilateral tonic-clonic seizures). We utilized functional connectivity networks estimated from interictal periods across patients as control. Our results characterize network features that quantify the underlying functional dynamics associated with the observed heterogeneity of seizure propagation across these two types of focal seizures. Decoding these network features demonstrate that bilateral propagation of seizure activity is an outcome of the imbalance of global integration and segregation in the brain prior to seizure onset.

We show that there exist intrinsic network signatures preceding seizure onset that are associated with the extent to which an impending seizure will propagate throughout the brain (i.e., staying within one hemisphere vs. spreading transcallosally). Additionally, these features characterize an increase in segregation and a decrease in excitability within the brain network (i.e., high modularity and low spectral radius). Importantly, seizure-type specific differences in these features emerge several minutes prior to seizure onset, suggesting the potential utility

of such measures in intervention strategies. Finally, our results reveal network characteristics *after* the onset that are unique to the propagation mechanisms of two most common focal seizure subtypes, indicative of distinct reconfiguration processes that may assist termination of each seizure type. Together, our findings provide insights into the relationship between the temporal evolution of seizure activity and the underlying functional connectivity dynamics. These results offer exciting avenues where graph theoretical measures could potentially guide personalized clinical interventions for epilepsy and other neurological disorders in which extensive heterogeneity is observed across subtypes as well as across and within individual patients.

Author affiliations:

[1]Humans In Complex Systems Division, US DEVCOM Army Research Laboratory, MD 21005, USA.

[2]Computational Neurobiology Laboratory, The Salk Institute for Biological Studies, CA 92037, USA.

[3]Neuroscience Center for Research and Innovation, Learning Institute, King Mongkut's University of Technology Thonburi, Bangkok, 10140, Thailand.

[4]Institute for Neural Computation, University of California San Diego, La Jolla, CA 92093, USA.

[5]Department of Neurology, Massachusetts General Hospital and Harvard Medical School, MA 02114, USA.

[6]Division of Biological Sciences, University of California San Diego, CA 92093, USA.

[7]Department of Biomedical Engineering, Columbia University, NY 10027, USA.

Correspondence to: Kanika Bansal

Full address: Department of Biomedical Engineering, Columbia University, New York, NY 10027

E-mail: phy.kanika@gmail.com

Running title (40 characters max): Network reconfigurations sculpt seizures

Keywords: epilepsy; functional connectivity; seizure propagation; brain networks

Abbreviations: ECoG = Electrocorticography

For Review Only

Introduction

As one of the most common neurological disorders with roughly 50 million cases world-wide, epilepsy is characterized by its emerging spontaneous seizure activity.[1,2] Critically, one-third of the patients do not respond to medications and rely on alternative interventions (e.g., surgical and neuromodulatory).[3–5] However, seizures are remarkably diverse, and tailoring effective treatment strategies remains a substantial challenge at least partially due to the temporal spontaneity and lack of objective frameworks that could characterize the onset and propagation patterns of an impending seizure.[6,7] Traditionally, the variability across seizures has been categorized based on the onset regions: *focal seizures* originate from a localized region within one hemisphere while *generalized seizures* begin simultaneously from both hemispheres. A variety of computational techniques from network science and dynamical systems have been employed to better localize the onset regions and thus improve the precision with which focal and generalized seizures can be identified.[8–10] However, localizing onset regions does not fully capture the breadth of dynamics and inherent diversity associated with seizure subtypes. Adding to this complexity, once generated, a focal seizure can remain localized within the same hemisphere (i.e., *focal seizures that remain focal*) or propagate to the other hemisphere (i.e., *focal to bilateral tonic-clonic seizures* or *focal seizures with bilateral spread).*[11–13] Notably, a single patient may experience both of these focal seizure subtypes (Fig. 1), where the seizures that spread bilaterally generally lead to more severe behavioral and cognitive deficits that could require several minutes to hours for patients to recover. However, the distinct propagation dynamics exhibited by different seizure types are largely ignored by traditional intervention approaches and it remains unknown if the mechanisms underlying the bilateral spread of focal seizure activity differ from those associated with focal seizures that remain localized. Critically, the field currently lacks an objective analytical framework that can be utilized to investigate, understand, and predict the heterogeneity associated with propagation dynamics of seizure activity. Objective biomarkers that can identify whether or not an impending seizure will spread bilaterally could allow clinicians to implement an appropriate intervention strategy, thereby minimizing the severity of adverse cognitive impacts.

For the past several years, epilepsy has more frequently been viewed as a manifestation of network reorganizations and not just hypersynchrony, and a graph theoretical framework in which brain dynamics is visualized as temporally evolving graphs or networks composed of nodes and edges that represent brain regions and their pairwise associations, respectively,[14–16]

For Review Only

has been useful in quantifying and understanding these network reorganizations.[17–19] Here, we demonstrate that the long-standing challenges associated with the heterogeneity observed across subtypes of epileptic seizures can also be addressed through the lens of graph theory. This approach relies on a complex systems view of the brain where a single brain region interacts with many others and collectively, these interactions give rise to a wide variety of functional connectivity patterns underlying our adaptive cognitive abilities and subsequent behavior. Investigating the temporal evolution of such connectivity patterns, within a graph theoretical framework, has provided better insight into the emergence of neural properties such as specialization and efficiency of information processing, learning, and aging[18,20–24] and has applications in clinical neuroscience for the potential to establish biomarkers of disease onset and progression.

Our study is built upon the idea that the manner in which functional brain connectivity networks reconfigure over time may carry information concerning the emergent seizure dynamics and cognitive behaviors that are unique to the underlying neurological processes. Consequently, we probed the time-varying changes within functional connectivity networks derived from multiple hours of electrocorticogram (ECoG) recordings across 14 patients as they experienced focal seizures that remain focal or focal to bilateral tonic-clonic seizures. With this analytical framework, we aimed to gain insight into how the unique heterogenous dynamic properties associated with different seizure types develop and unfold in the brain. Our results uncover key network features that characterize the different neural dynamics associated with each type of focal seizures. Further, our findings demonstrate that the emergence of different propagation patterns is an outcome of unique network-level changes and distinct mechanisms that regulate the extent of synchronization within the brain.

Materials and methods

Patient information and data acquisition

The seizures analyzed in this study were recorded from 14 patients with medication-refractory epilepsy (Table 1) who underwent a clinical monitoring procedure to locate their seizure onset zone. Clinical electrode implantation, positioning, duration of recordings, and medication schedules were based solely on clinical need as determined by an independent team of clinicians. As indicated in Table 1, seizures analyzed in this study were of two types:

For Review Only

1) focal seizures that remain focal and 2) focal to bilateral tonic-clonic seizures (or focal seizures with bilateral spread). Patients were implanted with intracranial subdural grids, strips, and depth electrodes for several days in a specialized hospital setting and continuous multichannel ECoG data were recorded at a sampling rate of 500 Hz.

Only seizures with an obvious ictal onset were selected for analysis. Experienced epileptologists, blind to this study, identified the seizure onset regions, seizure types, and onset time through inspection of the ECoG recordings, referral to the clinical report, and clinical manifestations recorded on video. A total of 67 seizures (49 focal seizures that remain focal and 18 focal to bilateral tonic-clonic seizures) were analyzed. We note that, multiple seizures from the same patients were treated as independent (see similar methods in[4]).

Ethics statement

All patients were enrolled after informed consent was obtained and approval was granted by local Institutional Review Boards (IRB) at Massachusetts General Hospital (MGH) according to National Institutes of Health (NIH) guidelines.

Data preprocessing

For each of the seizures, we considered ECoG data of the duration of 15 minutes before and 10 minutes after the seizure onset. Each of these 25-min data segments contained only one seizure. For comparison with relatively 'seizure-free' (interictal) activity, we extracted an equal number of interictal activity epochs with the same duration (i.e., 67 interictal epochs). Interictal epochs were selected from ECoG recordings at least an hour away from the onset and offset of any seizure. Specifically, for each of the 67 seizures (49 focal and 18 focal to bilateral tonic-clonic) from which we extracted a 25-minute segment (from 15 minutes before to 10 minutes after the onset), we also epoched a 25-minute segment of data that was at least 60 minutes away from the seizure onset and offset. The data were band-pass filtered between 1 to 70 Hz, and notch filtered at 60 Hz to exclude potential powerline interference. Prewhitening was then performed using a first-order autoregressive model to account for slow dynamics and correct for autocorrelation in the time series signals.[25–28] A common reference was used for data analysis and the reference electrode in each case was located far

For Review Only

from the area of recording making the introduction of spurious correlation or elimination of actual correlation between cortical regions unlikely.[29]

Functional connectivity networks

To evaluate functional connectivity representations associated with the temporal evolution of seizures, we employed a time-evolving network analysis. Here, we constructed time-varying functional connectivity networks of focal seizures and interictal activity where the inter-electrode relationships were represented by network edges and the electrodes themselves were represented by nodes in the corresponding networks of seizures and interictal activity (Fig. 2, A-D).

Specifically, we computed symmetric functional connectivity A_{ij} between two regions of the brain i and j as an averaged correlation of the neural signals recorded by the intracranial electrode contacts of those regions. To extract (at least approximately) the stationary aspects of ECoG data, we divided each of the 25-min ECoG data segments into consecutive 1-s windows, where each window overlapped the previous window by 0.5 seconds.[30,31] The correlation was calculated within each of these 1-s segments. To account for noise, we applied a temporal smoothing to these correlation values by averaging consecutive 30 seconds windows such that a total of 98 correlation values representing the functional connectivity of 98 temporal windows were generated from each 25-min ECoG data segment. Note that different temporal smoothing parameters can be used without affecting the overall patterns of results, although a value too large may reduce the temporal precision of the observations (Supplementary Fig. 1).

All correlation values were bounded between −1 and +1. Negative correlation values were then set to zero following traditional modeling choices adopted in previous studies[3,32] examining simulation of the epileptogenic effect. Temporal evolution of these correlations or connectivity matrices reflects the time-varying dynamics of the functional brain networks as recorded through the ECoG measurements.

Graph theoretical network analysis

For each of the seizures (49 focal and 18 focal to bilateral tonic-clonic) and interictal activity (67 epochs), we constructed a series of weighted, symmetric (undirected) connectivity

For Review Only

matrices *A* representing functional correlations across all recording electrodes. From these network matrices, we computed a series of graph network measures (described below) as a function of seizure types to quantify changes in network dynamics associated with evolution of focal seizures with constrained (focal seizure that remain focal) and unconstrained propagation mechanisms (focal to bilateral tonic-clonic seizures). We focused our analyses on complex network measures that are sensitive not only to the overall extent of connectivity within a network (i.e., density; see below), but also to the relative distribution of connectivity weights within the network which reconfigure to enable various neurophysiological processes. Investigating and quantifying these network reconfigurations as a function of seizure types allows for a better understanding of the underlying processes that govern the bilateral spread of a focal seizure. We used various Brain Connectivity Toolbox functions implemented in MATLAB (R2020; MathWorks) for our computation of these network features unless noted otherwise.

Network density

Network density describes the extent of connectedness in a network and is calculated as the ratio of actual and possible connections in a network. A density of 1 describes a fully connected network and a density of 0 describes no connectivity. Seizures have been shown to alter the synchronization, and therefore, overall connectivity and density of the functional network. We calculated network density as the ratio of total edge weights and number of possible edges. For a functional connectivity matrix *A* representing an undirected network with *N* nodes, the total number of possible edges is $N(N-1)/2$ and the total edge weight is the sum of the elements in the upper triangle of the connectivity matrix.

Networks with different density (and/or size) can have different network features[33]. Therefore, we compared network density across individual patients during interictal period to ensure that our findings are truly driven by the underlying changes in the functional connectivity as a function of seizure types and are free of spurious results due to different network sizes and densities across patients (Supplementary Fig. 2).

Clustering coefficient

Clustering coefficient (CC) is a measure of local cliquishness of a network and has been used to describe the segregation of information in brain networks. While a high CC has been reported to characterize the ictal phase for temporal lobe epilepsy patients[34], the role of CC in

For Review Only

differentiating constrained and unconstrained seizure dynamics is not well understood. The clustering coefficient is calculated as the ratio between the number of triangles present around a node and the maximum number of triangles that could possibly be formed around that node.[35,36] For a given node *X* and any other two nodes *Y* and *Z* within the network, a triangle around *X* represents a scenario where *X*, *Y*, and *Z* all have a connectivity value of one with one another. We used the Brain Connectivity Toolbox function *clustering_coef_wu* for the calculation of clustering coefficient.

Characteristic path length

Characteristic path length (PL) describes the averaged minimum distance between all pairs of nodes in a network and has been associated with the brain's ability to integrate information. The minimum path length between a pair of network nodes represents the shortest route between them through a combination of network edges. We calculated characteristic path length using the Brain Connectivity Toolbox function *charpath*.[37] Both clustering coefficient and characteristic path length are used to assess if a network is more "regular" (only nearest neighbor connections) or "random" in its connectivity. Regular networks have high clustering and long path length, whereas random networks have low clustering and short path length. Typically, brain networks exhibit a small-world architecture which is characterized by a combination of dense local clustering of connections between neighboring nodes (like regular networks) and a short path length between distant node pairs due to the existence of relatively few long-range connections (like random networks).[14,15,18,38]

Assortativity

Assortativity measures the propensity of nodes to connect to others with similar degree and is calculated as a correlation between the degrees of all the nodes.[39] A positive assortativity value indicates that nodes tend to link to other nodes with similar degree, whereas a negative value indicates connected nodes with dissimilar degree. Networks with high assortativity tend to make a highly connected core of network hubs. Functional brain networks have been shown to display such an architecture with highly connected hub regions or core surrounded by low-connectivity peripheral nodes.[40,41] Assortativity quantifies network robustness as a removal or failure of a single high-degree node would induce greater impact on communication efficiency of a network with low assortativity than on a network with high

For Review Only

assortativity. We calculated assortativity using the Brain Connectivity Toolbox function *assortativity_wei*.[37]

Modularity

Modularity describes the extent to which a graph can be divided into clearly separated communities (i.e., subgraphs or modules). Each module contains several interconnected nodes, and there are relatively few connections between nodes of different modules. In the context of brain networks, modularity has been used to describe and quantify efficient integration and segregation of information across distributed sets of brain regions as a function of cognitive task demands.[42,43] Mathematically, the modularity metric (Q) represents the number of edges falling within modules minus the expected number in an equivalent network with edges placed at random. It is estimated as follows: $Q = \frac{1}{2m} \sum_{ij}\left(A_{ij} - \gamma\frac{k_i k_j}{2m}\right)\delta(c_i, c_j)$, where A_{ij} represents the connectivity between node i and j, k_i represents the degree of the node i, m represents the total number of edges in the network, γ represents a resolution parameter, and $\delta(c_i, c_j)$ equals 1 if nodes i and j are in the same community, and equals 0 otherwise.[44] Here, to detect modules and obtain a value of modularity for functional brain networks, we used the spectral approach described by Newman and implemented it through Brain Connectivity Toolbox function *modularity_und*.[44,45] The resolution parameter γ was chosen to be 1.

For Review Only

Spectral radius

Spectral radius is a global measure of network structure that is related to the spread of activity in a network.[23,46,47] Computed as the largest eigenvalue of the connectivity matrix (A), spectral radius reflects the critical coupling strength required to synchronize the system.[48] As such, spectral radius represents the principal component of the system and contains information about structural characteristics as well as dynamical behavior and stability of the underlying network.[49–51] In the network based models of brain dynamics, spectral radius has been associated with the ease with which the system can be transitioned into an excited state.[23]

Synchronizability

Synchronizability relates to the viability of synchronized dynamics within a network. Particularly in the context of epilepsy, relatively larger value of synchronizability has been associated with greater ease for neural populations to synchronize their dynamics.[24] Synchronizability is sensitive not only to the overall connectivity or density of a network but also to the relative distribution of edge weights across network nodes.[52] Here, we leveraged the measure of synchronizability to assess network reconfigurations that may happen around the onset of different seizure types. Synchronizability (S) is calculated as the ratio of the second smallest and the largest eigenvalue of the Laplacian matrix (L), which is computed as the difference between the diagonal matrix of node strength (total degree) and the adjacency matrix such that $L = D - A$. Thus, synchronizability estimates the spread of the eigenvalues of the network Laplacian and is computed as $S = \frac{\lambda_2^L}{\lambda_{\max}^L}$ where λ_2^L and $\lambda_{\max}^L$ denote the second smallest and the largest eigenvalue of L, respectively.

Statistical analysis

Given the diverse nature of seizure activity and the patient-specific procedures by which the placement and number of ECoG electrodes is determined, the recorded signals associated with individual seizures are inherently variable. It is well-documented that even within a single patient, different occurrences of seizure activity can vary in their durations, phases, and latencies of spread[11,53,54]. To account for such variability across individual seizures and to minimize statistical biases that could arise, for each network feature we used a bootstrapping procedure[55] to generate estimates of the mean and 95% confidence intervals (CIs), and made comparisons across seizure types in a time resolved manner. To perform these comparisons, we employed a rigorous statistical analysis as described in the following.

First, for each network measure and for each timepoint, we resampled with replacement at the level of individual seizures 10,000 times. Through these bootstrapping iterations, we generated an empirical estimate of the variability associated with each network measure of each seizure type over time (e.g., a bootstrapped distribution of the clustering coefficient associated with focal seizures that remain focal, a bootstrapped distribution of the clustering coefficient associated with focal to bilateral tonic-clonic seizures, and a bootstrapped distribution of the clustering coefficient associated with interictal activity). Next, to compare a particular network feature between conditions (e.g., focal seizures that remain focal vs. focal to bilateral tonic-clonic seizures), we calculated the difference at each time point

For Review Only

between these bootstrapped data associated with each condition to obtain distributions of "differences" with respective means and CIs specific to each time point for each comparison (e.g., difference between the clustering coefficient of focal seizures that remain focal and that of focal to bilateral tonic-clonic seizures; Supplementary Fig. 4, Supplementary Fig. 5) . These distributions of "differences" were used to assess the *P* values and effect sizes, and to determine if a network feature significantly differentiates seizure types at a given time point.

To assess significance (though *P* values), we evaluated the observed distribution of bootstrapped "differences" against chance. To establish chance levels, we constructed null distributions non-parametrically for each complex network measure and for each time point by shuffling the condition labels 10,000 times, each time recomputing the difference between the seizure conditions on the shuffled data. Consequently, each null distribution centered at zero (e.g., the clustering coefficient does not differ between focal seizures that stay focal and focal to bilateral tonic-clonic seizures; difference of zero). We compared the bootstrapped empirical and null distributions by conducting 2 one-tailed tests against zero, i.e., mean[difference in clustering coefficients] < 0 and mean[difference in clustering coefficients] > 0) and then doubling the smaller *P* value. As the CIs for each comparison at each time point was achieved based on 10,000 bootstrapping iterations, the resolution of *P* values was constrained to a lower limit of $P \leq 0.0001$. A *P* values < 0.05 was deemed significant.

For each comparison, we estimated effect sizes in two different ways: (i) by calculating the mean of the bootstrapped "difference" distributions (e.g., mean[the clustering coefficient of focal seizures that remain focal *minus* the clustering coefficient of focal to bilateral tonic-clonic seizures]; Supplementary Fig. 4 and Supplementary Fig. 5); and (ii) by directly computing Cohen's *d*[56,57] for the bootstrapped distributions of each network feature (e.g., the clustering coefficient of focal seizures that remain focal vs the clustering coefficient of focal to bilateral tonic-clonic seizures). For each of the significantly different statistical comparisons the smallest Cohen's *d* (Cohen's d_{min}) is reported. For each network measure, this value indicates the effect size associated with the temporal window at which the difference between seizure types were statistically significant but smallest. For example, if the clustering coefficient is lower in focal seizures that remain focal than in a focal to bilateral tonic-clonic seizures from time t_1 to t_2, the reported Cohen's d_{min} signifies the

For Review Only

smallest effect size within this [t_1 t_2] period. The 95% CI associated with each Cohen's d_{min} is also reported.

Data availability

All data that support the findings of this study are present in the main text and/or the Supplementary Materials. These data are available on request from the corresponding author. The data are not publicly available as they contain information that could compromise privacy of the research participants.

For Review Only

Results

Increased clustering and shorter path length following bilateral spread of seizure activity

In the context of epilepsy, clustering coefficient (CC) and characteristic path length (PL) are most commonly studied graph theoretical features which are utilized to assess if and how seizure activity affects the small-world characteristics of the brain.[17,34,58] Here, we did not observe any significant differences in the small-world characteristics of the functional connectivity networks associated with the constrained and unconstrained seizure propagation dynamics (Supplementary Fig. 3). We, therefore, independently evaluated CC and PL to investigate if each measure differed between the constrained and unconstrained propagation mechanisms associated with focal seizures that remain focal and focal seizures with bilateral spread, respectively. To accomplish this, we calculated the clustering coefficient and the characteristic path length of each connectivity matrix (i.e., 98 matrices per each of the 25-minute segments of seizure activity). First, to evaluate these results in light of past studies, we computed averages of these values in a series of consecutive 5-minute windows, separately for each seizure type. This resulted in three preictal, one ictal (during seizure), and one postictal windows (Fig. 3, A and B). A similar analysis was applied to interictal data to estimate baseline values to which the seizure-related network measures could be compared.[37]

Our results reveal that both focal seizures that remain focal and focal to bilateral tonic-clonic seizures displayed lower PL during ictal periods when compared to preictal activity (Fig. 3, A and B). Further analyses demonstrated that the ictal activity associated with focal seizures

that remain focal exhibited (i) higher CC as compared to postictal period ($P = 0.01$; Fig. 3A, left panel) and (ii) lower PL as compared to both preictal and postictal periods (preictal: $P < 0.001$, < 0.01, < 0.01; postictal: $P < 0.001$; Fig. 3B, left panel). Additionally, we observed similar changes for focal seizures with bilateral propagation where the ictal activity displayed (i) higher CC as compared to all the preictal periods (all $P < 0.001$; Fig 3A, right panel) and (ii) shorter PL as compared to all the preictal periods (all $P < 0.001$; Fig 3B, right panel). However, unlike the CC and PL associated with the postictal periods of focal seizures with constrained dynamics which returned to the preictal levels, the postictal PL of focal seizures with bilateral spread exhibited a continued decrease ($P < 0.0001$, Fig 3B, right panel). These results supported the more unconstrained diffusivity associated with focal to bilateral tonic-clonic seizures. These observed differences regarding the manner in which the CC and PL changed in the networks of focal seizures with constrained and unconstrained dynamics were our first evidence in support of the notion that there may exist network-level signatures that contained information about the distinct propagation mechanisms of focal seizures.

For Review Only

Further, we directly compared the temporal profiles of CC and PL for focal seizures that remain localized and focal seizures with bilateral spread. To account for the unequal number of seizure samples of each seizure type, we implemented a rigorous bootstrapping procedure and established 95% confidence intervals based on which significant difference was assessed (see **Materials and methods**). As expected from Figures 3A-B, we observed that the dynamics of CC and PL differed in a *seizure-type specific* manner only after the onset of seizures. Specifically, CC of focal seizures with bilateral spread was higher than that of focal seizures that remain focal, from 2.25 to 10 minutes after seizure onset (resampled $P < 0.05$, the smallest value of the Cohen's d within this interval, or $d_{min} = 1.06$, 95% CI_{min} [-0.15, -0.02]; Fig. 3C). Such differences were accompanied by shorter PL associated with focal seizures with bilateral spread (resampled $P < 0.05$ for 2-10 minutes after seizure onset, Cohen's $d_{min} = 0.78$, 95% CI_{min} [0.07, 1.06]; Fig. 3D). Notably, these observed differences emerged only after the onset and extended well beyond termination of seizures[17], suggesting that focal to bilateral tonic-clonic seizures differentially induced network reorganization that persisted even after the seizure activity ended.

Additionally, after seizure onset, persistent differences in CC and PL were also observed between focal seizures with bilateral spread and interictal activity (Supplementary Fig. 6, right panels). These persistent differences between post-onset activity and interictal periods

were, however, not observed in the case of focal seizures that remain focal (Supplementary Fig. 6, left panels). An increase in CC illustrates increased local cliquishness and a decrease in PL implies better connectivity across the underlying network nodes. Together, these findings suggest that the unconstrained propagation dynamics of focal to bilateral tonic-clonic seizures are related to an increase in the overall network connectivity and consequently improved network communication shortly after seizure onset. Critically, these observed seizure-type dependent network configurations emerged only after the onset, raising a question whether there also existed unique network alterations at other time points that may contribute to the distinct propagation mechanisms and clinical manifestations associated with each seizure type.

Alterations in the *local connectivity* features after the onset reflect heterogeneous dynamics of focal seizures

Given the post-onset differences in the clustering coefficient and the characteristic path length between focal seizures of different propagation mechanisms, we hypothesized that seizure-type dependent network changes should also be observed in other measures of node connectivity patterns such as the network density. A network with high density is well positioned to optimize integration of information and increase the efficiency of network communication.[37,38] We expected, therefore, that the networks after the onset of focal seizures with bilateral spread would show higher density as compared to those after the onset of focal seizures that remain focal. Supporting our hypothesis, the network density associated with focal to bilateral tonic-clonic seizures was found to be higher than that of focal seizures that remain focal for 1.75-10 minutes after the onset (resampled $P < 0.05$, Cohen's $d_{min} = 0.73$, 95% CI_{min} [-0.10, 0]; Fig. 4A). Notably, the timing of the sustained differences in the density mirrored that of the clustering coefficient and the characteristic path length, which also extended several minutes beyond seizure termination as each seizure typically lasted between 30 seconds and 3 minutes.[59]

To further investigate network alterations unique to particular propagation mechanisms of focal seizures, we assessed the assortativity coefficient which measures the propensity of network nodes to connect to other nodes of similar degree.[39,60] Our results revealed that the assortativity coefficient associated with focal to bilateral tonic-clonic seizures was lower than that of focal seizures that remain localized for 7.50-9.25 minutes after seizure onset (resampled $P < 0.05$,

For Review Only

Cohen's d_{min} = 0.84, 95% CI_{min} [0.01, 0.06]; Fig. 4B). Additionally, similar patterns of results were observed between focal to bilateral tonic-clonic seizures and interictal activity such that the seizure networks displayed higher network density (resampled $P < 0.05$ for 1.75-10 minutes after seizures onset, Cohen's d_{min} = 0.92, 95% CI_{min} [0.01, 0.11]; Supplementary Fig. 7A) and lower assortativity (resampled $P < 0.05$ for 6.50-7 and 7.50-9.75 minutes after seizures onset, Cohen's d_{min} = 0.56, 95% CI_{min} [-0.05, 0]; Supplementary Fig. 7A). However, these network properties did not differ between interictal activity and focal seizures that remain localized. Importantly, the observed seizure-type differences emerged after the onset of seizures and were driven by the negative assortativity coefficient that was associated with focal seizures with bilateral propagation. This negative assortativity represents a disassortative network in which high-degree nodes, or network hubs, are less likely to connect with other high-degree nodes in the network.[60,61]

Thus far, we demonstrated that consistent with the differences in the clustering coefficient and the characteristic path length after seizure onset, the density and the assortativity (i.e., the measures directly derived from local or nodal connectivity), also differed as a function of seizure propagation dynamics. These findings provided better understanding regarding the association between heterogeneous propagation mechanisms of seizure activity and the local connectivity within the underlying functional networks. Next, we asked if networks of different seizure types underwent distinct reconfigurations *prior to* seizure onset that shaped the global properties of the networks and ultimately determined the type of propagation dynamics an impending seizure would display.

For Review Only

Alterations in subtle *global* network features preceding the onset differentiate propagation dynamics of focal seizures

Building upon the findings presented thus far, we next aimed to quantify the distinct network alterations which could differentiate the propagation patterns prior to seizure onset. To accomplish this, we assessed network attributes related to various aspects of information processing within a networked system, particularly, the brain connectivity network. Specifically, we focused on three network features: modularity[44,62–64], spectral radius, and synchronizability.[47,65] While modularity has recently been utilized in characterizing the efficiency associated with integration and segregation of information across distributed brain areas, the properties of spectral radius and synchronizability remain relatively unexplored in

the context of brain networks. A couple of recent studies, however, have suggested the utility of synchronizability and spectral radius in describing dynamics of seizure activity within the brain[24] and the extent of excitability of brain networks, respectively[23]. Because modularity, synchronizability, and spectral radius have been associated with different neural processes and are sensitive to changes in the network connectivity, we hypothesized that these measures would be powerful markers for prediction of seizure dynamics prior to the onset. As described in the Materials and methods, each of these attributes relate to overall network architecture and their values may differ across networks with similar distribution of node degrees. Consequently, we characterized modularity, synchronizability, and spectral radius as *subtle global* network features and, in the following, investigated how they change over time as a function of seizure propagation dynamics.

Our results revealed that the information concerning the propagation patterns of focal seizures could be decoded from these global network attributes several minutes prior to seizure onset. Specifically, the modularity preceding the onset of focal seizures with bilateral spread was higher than that of focal seizures that remain localized (resampled $P < 0.05$ for 14.75-12.00, 11.00-10.75, 9.25-8.5, 8.25-8.00, 7.67-7.50, and 6.75-5.75 before seizure onset; Cohen's $d_{min} = 0.75$, 95% CI_{min} [-0.10, -0.01]; Fig. 4C). This pattern of results was also observed in the spectral radius (resampled $P < 0.05$ for 14.75-14.50, 14.00-11.25, 11.00-10.75, 9.75-9.50, 9.25-9.00, 7.75-7.50, 7.00-5.75, 5.50-5.00, and 4.50-3.75 minutes before seizure onset; Cohen's $d_{min} = 0.66$, 95% CI_{min} [0.27, 8.14]; Fig. 4D). These seizure-type dependent differences in the network modularity and spectral radius re-emerged shortly after seizure onset (resampled $P < 0.05$ for 0.75-1.50 minutes after seizure onset for both modularity and spectral radius). As for the synchronizability, we observed an increasing trend post seizure onset associated with unconstrained seizure dynamics, however our statistical analyses revealed no significant difference in synchronizability across seizure types (Fig. 4E).

Similar patterns of results were also observed between focal seizures with bilateral spread and interictal activity such that preceding the onset, the seizure networks displayed higher modularity (resampled $P < 0.05$ for 14.75-14, 13.75-13, 12.75-12, 10.25-10, 9.75-9.50, 8.50-8.25 and 7.75-7.50 minutes before seizure onset; Cohen's $d_{min} = 0.71$, 95% CI_{min} [0.01, 0.09]; Supplementary Fig. 7C), and lower spectral radius (resampled $P < 0.05$ for 14.75-14.50, 14-12.25, 11-9.50, 7.75-7.50, 5.25-4.75, and 4.50-3.75 minutes before seizure onset; Cohen's $d_{min} = 0.2$, 95% CI_{min} [-7.63, -0.35]; Supplementary Fig. 7D). These results were

For Review Only

accompanied by post-onset effects where focal to bilateral tonic-clonic seizures exhibited higher spectral radius (resampled $P < 0.05$ for 0.75-1.50, 2.50-3, and 5-5.50 minutes after seizure onset; Supplementary Fig. 7D), and higher synchronizability (resampled $P < 0.05$ for 6-6.25 and 7.25-12.50 minutes after seizure onset; Supplementary Fig. 7E). However, focal seizures that remain localized only differed from interictal activity in the measure of modularity and synchronizability such that the modularity of the focal seizures was lower shortly after seizure onset (resampled $P < 0.05$ for 1-4.25 minutes after seizure onset; Cohen's $d_{min} = 0.41$, 95% CI_{min} [-0.06, 0]; Supplementary Fig. 7C) and the synchronizability of the focal seizures was higher shortly after the seizure onset (resampled $P < 0.05$ for 1.25-2.25 minutes after seizure onset; Cohen's $d_{min} = 0.59$, 95% CI_{min} [0.01, 0.09]; Supplementary Fig. 7E).

Complementary temporal reconfigurations within the functional connectivity networks sculpt seizure dynamics

Using a set of graph theoretical features, we identified reconfigurations in the functional connectivity network that characterized the propagation dynamics of different seizure types. Our results revealed that such distinguishing features can be classified into 2 groups based on the distinct and complementary temporal windows at which the differences in these features emerged as a function of seizure types. The first group of network attributes includes the global features modularity and spectral radius, which primarily captures differences between focal seizures with constrained and unconstrained dynamics *prior to* seizure onset (Fig. 5). In contrast, the second group of network properties captured differences across seizure types *after* the onset, reflecting the network reconfigurations induced by distinct propagation mechanisms. Such features include the density, assortativity, clustering coefficient, and characteristic path length (Fig. 5).

Notably, the first group of network features, i.e., modularity and spectral radius, can detect the possibility of an impending seizure to spread bilaterally and can be further investigated as potential biomarkers. To further highlight the utility and robustness of these features in assessing the likelihood of a focal seizure to spread bilaterally, we evaluated modularity and spectral radius at a single-seizure level in three seizures that shared similar onset regions in the left hemisphere and were recorded from a single patient (Fig.1 and Fig. 6). Specifically, seizure 2 bilaterally propagated after the onset while seizure 1 and 3 remained localized within the left

For Review Only

hemisphere. We showed that seizure 1 and 3 exhibit similar dynamics of modularity and spectral radius over time, and these temporal patterns differ from those of seizure 2. For seizure 2, we observe an early sharp increase in modularity and a decrease in spectral radius consistent with the findings across seizures (Fig. 5), further highlighting the robustness of our results (grey shaded area). These patterns were followed by an abrupt decrease in modularity and a sharp increase in spectral radius (Fig. 6; yellow shaded area) which could be seizure- and/or patient-specific features. These results further suggested that network measures could potentially be used to characterize distinct neural dynamics across different types of focal seizures, even on a single-seizure basis.

Discussion

For Review Only

The goal of the present study was to investigate if the emergence of heterogeneity in seizure propagation is an outcome of mechanistically different disruptions and can be understood in terms of network-level changes within the brain before, during, and after the onset. To accomplish this, we evaluated the temporal evolution of a series of graph theoretical attributes which quantify various aspects of network organization and information processing within complex systems such as the brain. We demonstrated distinct network-level signatures that were associated with the extent of diffusion dynamics of an impending seizure as well as isolated architectural changes within the functional connectivity networks that emerged as the seizures terminated. These results advance our understanding of how heterogeneous seizure dynamics can arise from similar onset regions. Furthermore, our findings provide a rationale for a potential use of network features to help guide clinical diagnosis of focal seizures with different propagation mechanisms (focal seizures that remain local vs. focal to bilateral tonic-clonic seizures). Incorporation of such network measures with other biomarkers of neural state changes[4,54,66] could also potentially lead to improvement of effective intervention strategies to constrain propagation of seizure activity.

Network alterations track temporal evolution of focal seizures

We demonstrate that dynamic reconfigurations within the functional connectivity networks during evolution of focal seizures give rise to the heterogeneity observed across seizures. While past work primarily evaluated network properties of epileptic brain by averaging the signals in large discrete time windows, we assess the continuous temporal evolution of network

connectivity in combination with resampling statistical tests. This formulation enables us to characterize the temporal dynamics of network alterations that underlie the emerging dynamics of seizure activity in a rigorous manner. By examining *globally defined* network features, we observe distinct macroscopic signatures which could predict the extent of diffusivity of seizure propagation minutes *prior to* the onset. Our results indicate that processes leading to the emergence of distinct seizure propagation patterns are coded in the network activity minutes prior to the onset and can have mechanistically different underpinnings. We argue that our approach provides an objective framework not only for better understanding the neural dynamics underlying evolution of seizures but also for determining whether and when a clinical intervention should be implemented to manage and control a spread of an impending seizure.

Additionally, we further demonstrate that the heterogeneous dynamics exhibited across seizure types can be characterized by *post-onset* changes in local features of the functional connectivity networks such as the clustering coefficient, characteristic path length, and network density. Specifically, our findings revealed a relationship between the clustering coefficient, characteristic path length, and a binary measure of diffusivity of seizure activity after the onset (i.e., whether the seizure activity would remain within the same hemisphere or spread transcollosally). These measures indicate higher network connectivity following the seizure onset which could be correlated with more severe cognitive impacts that the seizures with unconstrained propagation dynamics typically induce. Any such correlation, however, needs to be confirmed with future experimentation. Notably, immediately after the onset where we observed higher network connectivity, no significant changes in the assortativity were observed. These results indicate that there was no particular reconfiguration in how the network hubs connect either with one another or with non-hubs. These observed patterns, however, changed closer to seizure termination — the networks of focal seizures with unconstrained dynamics showed negative assortativity indicative of a disassortative network, where highly connected nodes (hubs) are less connected to one another. Assortativity can be directly linked to the robustness of the network, in terms of its ability to remain connected[60]. A failure of a hub node in an assortative network would leave other hub nodes connected to one another, minimizing the chance of the network as a whole to become disconnected. However, in case of a seizure that spreads bilaterally, emergence of a disassortative network likely reflects a mechanism to reduce this "robustness" by restricting the connectivity among network hubs.

For Review Only

Bilateral propagation of focal seizures reflects overcompensation for an imbalance in global integration and excitability

Preceding the onset, we reported increased modularity along with decreased spectral radius in focal to bilateral tonic-clonic seizures. In light of classical accounts on the mechanistic underpinnings of seizures, we argue that our findings could reflect the chemical or dynamic imbalance within the underlying networks.[6,67] Specifically, microscopic disproportion between excitation and inhibition or in the bistability of localized neural dynamics could lead to an emergence of a neural state with significantly low integration (or high segregation captured by high modularity[38]) and low excitability (captured by low spectral radius).[68] To regain a more balanced state, it is likely that mechanisms enhancing the connectivity between segregated networks are recruited, leading to an over-compensation which manifests as more unconstrained seizure dynamics. Future studies utilizing large-scale non-invasive neuroimaging methods could seek validation and/or refinement to this hypothesis. Notably, we found that such signatures, i.e., increased modularity as well as decreased spectral radius, disappeared just before the onset, re-emerged shortly after, and again disappeared (Fig. 4). It is, therefore, possible that these network features reflect the manifestations of the regulatory mechanisms that govern emergence and termination of seizures. In addition, the modularity of focal seizures that remain focal decreased shortly after the onset as compared to interictal activity (Supplementary Fig. 7C), further highlighting the link between this network feature and control mechanisms associated with seizure termination.

For Review Only

While we did not observe a significant difference of synchronizability between focal seizures with constrained and unconstrained dynamics, Khambhati et al.[24] showed significantly high synchronizability for unconstrained dynamics within the gamma oscillations. Further investigation of network reconfiguration within specific oscillatory bands can be helpful in drawing associations between network reconfigurations and cognitive implications of a specific seizure type.

Time-dependent assessment of network attributes can guide development of personalized seizure treatment

The heterogeneity of epilepsy is a key confound to disease understanding and development of effective treatments. Here, we demonstrate graph theoretical features as novel potential

candidates of biomarkers that link differential reconfigurations of the functional connectivity networks to the heterogeneity in the emerging seizure dynamics. Specifically, our investigations of the global network dynamics suggest that interventions aiming to contain the spread of seizure activity may wish to situate the brain in a topological state where the modularity is lowered, while the spectral radius are increased. In addition, we also show that the information regarding the propagation patterns of seizures can be decoded through the seizure-type dependent changes in the network properties several minutes before seizure onset allowing sufficient time for an intervention to be implemented. Furthermore, the seizure-type dependent signatures observed post-onset can be used to validate the efficiency of a particular treatment approach in preventing evolution of seizures and may help determine the extent of cognitive and behavioral deficits induced by the residue seizure activity in a scenario where the intervention did not completely eliminate the seizures. Future studies that wish to characterize cognitive and behavioral changes induced by neurological disorders may also benefit from evaluating these network properties in relation to performance of patients on various test battery.[18,69] Such analyses could uncover distinct underlying pathophysiological processes that give rise to diverse cognitive and behavioral impairments across disease subtypes and across individuals, thereby improving understanding of the disease heterogeneity.[70–73] Finally, our single-seizure analyses suggest that network measures could potentially be used to characterize distinct neural dynamics across different types of seizures, even on a single-seizure basis. Such findings provide foundation for future investigation and development of effective personalized seizure treatment.

Future directions

Given that the electrode placement was determined on a patient-to-patient basis by a neurologist for the purpose of identification of seizure onset zones, the data extracted from these electrodes inevitably provide an incomplete picture of the brain network due to the resulting partial coverage. In addition, the reported lack of differences between focal seizures that remain focal and interictal activity could be partially due to such spatial sampling of the recorded signals. To address this possibility, future studies may benefit from non-invasive recordings where whole-brain dynamics can be simultaneously evaluated.

Further, our analyses treated multiple seizures and interictal activity segments from the same patients as independent, and primarily disregarded individual variability in seizure

For Review Only

heterogeneity at the patient-level. This analytical choice was made based on traditional methods (e.g., see[4]), and careful statistical comparisons were implemented to identify the seizure-type dependent alteration patterns in the functional connectivity networks of seizures. To further extend our findings and improve the specificity of the interpretations, future studies may incorporate patient-level factor in their analytical frameworks. While we reported distinctive network features to distinguish constrained and unconstrained seizure dynamics, it is likely that some of the subtle differences remained undetected given our temporal resolution and strict statistical approach. By combining our analytical framework with a finer temporal resolution, future studies can look for unique network features associated with different phases of seizure propagation as a function of seizure types.

We illustrated the potential utility of complex network features in distinguishing seizures with constrained and unconstrained dynamics prior to the onset at a single-seizure level. However, in this sample patient, we also found patterns of network reconfigurations that were not observed at the population level. Using the analytical framework we presented, future studies can further validate the seizure- and patient-specific applications of network features in clinical settings.

For Review Only

Conclusions

In summary, by using a graph theoretical approach, we determined the extent to which distinct emerging dynamics of seizure networks were accounted for by temporal reconfigurations of the underlying functional connectivity. Collectively, our results illustrated a series of network metrics that can potentially be utilized as quantitative biomarkers to distinguish between focal seizures of distinct dynamics based on their propagation patterns as well as the differential extent of cognitive and behavioral effects accompanying the seizures. These results suggested that the networks of focal seizures with unconstrained dynamics undergo early network alterations, triggering processes which facilitate the bilateral diffusion of seizure activity. The propagation-type dependent alterations in these metrics were observed again shortly after the onset, suggesting that these measures could also induce regulatory mechanisms necessary for the termination of seizures. Together, our findings provide objective means to gain better insight into the mechanisms by which seizure dynamics are regulated within the brain and provide exciting avenues where graph theoretical measures could be used to guide personalized clinical interventions.

Acknowledgements

We wish to thank Pariya Salami and Mia Borzello for assistance with data preprocessing. We are also grateful to the clinical team, technicians, and our research participants for their help in making this research possible.

Funding

This work was supported by NIH NIBIB R01EB026899-01 (T.J.S. and C.L.), NINDS R01NS104368 (T.J.S.), NIH/NINDS NS062092 (S.S.C.), Mission funding from the U.S. Army Research Laboratory (J.O.G.), and cooperative agreements under the U.S. Army Research Laboratory W911NF-16-2-0158 (K.B.) and W911NF-17-S-0003-03 (N.R.). The views and conclusions contained in this document are those of the authors and should not be interpreted as representing the official policies, either expressed or implied, of the Army Research Laboratory or the U.S. Government.

For Review Only

Competing interests

The authors report no competing interests

Supplementary material

Supplementary material is available at *Brain Communications* online.

References

1. Mormann F, Kreuz T, Rieke C, et al. On the predictability of epileptic seizures. *Clin Neurophysiol*. 2005;116(3):569-587.
2. Jiruska P, de Curtis M, Jefferys JGR, Schevon CA, Schiff SJ, Schindler K. Synchronization and desynchronization in epilepsy: Controversies and hypotheses. *J Physiol*. 2013;591(4):787-797.
3. Sinha N, Dauwels J, Kaiser M, et al. Predicting neurosurgical outcomes in focal epilepsy patients using computational modelling. *Brain*. 2017;140(2016):319-332.

4. Martinet LE, Kramer MA, Viles W, et al. Robust dynamic community detection with applications to human brain functional networks. *Nat Commun*. 2020;11(1):1-13.

5. Salami P, Peled N, Nadalin JK, et al. Seizure onset location shapes dynamics of initiation. *Clin Neurophysiol*. 2020;131(8):1782-1797. https://doi.org/10.1016/j.clinph.2020.04.168

6. Kuhlmann L, Lehnertz K, Richardson MP, Schelter B, Zaveri H. Seizure prediction -- ready for a new era. *Nat Rev Neurol*. 2018;14:618-630.

7. Lainscsek C, Rungratsameetaweemana N, Cash SS, Sejnowski TJ. Cortical chimera states predict epileptic seizures. *Chaos*. 2019;29(12).

8. Jirsa VK, Stacey WC, Quilichini PP, Ivanov AI, Bernard C. On the nature of seizure dynamics. *Brain*. 2014;137(8):2210-2230.

9. Saggio ML, Crisp D, Scott JM, et al. A taxonomy of seizure dynamotypes. *Elife*. 2020;9:1-56. doi:10.7554/eLife.55632

10. Li A, Chennuri B, Subramanian S, et al. Using network analysis to localize the epileptogenic zone from invasive EEG recordings in intractable focal epilepsy. *Netw Neurosci*. 2018;1(3):222-241.

11. Fisher RS, Cross JH, D'Souza C, et al. Instruction manual for the ILAE 2017 operational classification of seizure types. *Epilepsia*. 2017;58(4):531-542.

12. Fisher RS, Cross JH, French JA, et al. Operational classification of seizure types by the International League Against Epilepsy: Position Paper of the ILAE Commission for Classification and Terminology. *Epilepsia*. 2017;58(4):522-530.

13. Blumenfeld H, Varghese GI, Purcaro MJ, et al. Cortical and subcortical networks in human secondarily generalized tonic-clonic seizures. *Brain*. 2009;132(4):999-1012.

14. Bullmore E, Sporns O. Complex brain networks: Graph theoretical analysis of structural and functional systems. *Nat Rev Neurosci*. 2009;10(3):186-198.

15. Bassett DS, Sporns O. Network neuroscience. *Nat Neurosci*. 2017;20(3):353-364.

16. Wykes RC, Khoo HM, Caciagli L, et al. Wonoep appraisal: Network concept from an imaging perspective. 2019;60(7):1293-1305.

17. Kramer MA, Eden UT, Kolaczyk ED, Zepeda R, Eskandar EN, Cash SS. Coalescence and fragmentation of cortical networks during focal seizures. *J Neurosci*.

For Review Only

2010;30(30):10076-10085.

18. Stam CJ. Modern network science of neurological disorders. *Nat Rev Neurosci*. 2014;15(10):683-695.

19. Stacey W, Kramer M, Gunnarsdottir K, et al. Emerging roles of network analysis for epilepsy. *Epilepsy Res*. 2020;159:106255. doi:10.1016/j.eplepsyres.2019.106255

20. Proix T, Bartolomei F, Guye M, Jirsa VK. Individual brain structure and modelling predict seizure propagation. *Brain*. 2017;31(3):13292-13300.

21. Ridley BGY, Rousseau C, Wirsich J, et al. Nodal approach reveals differential impact of lateralized focal epilepsies on hub reorganization. *Neuroimage*. 2015;118:39-48.

22. Garcia JO, Ashourvan A, Muldoon S, Vettel JM, Bassett DS. Applications of community detection techniques to brain graphs: Algorithmic considerations and implications for neural function. *Proc IEEE Inst Electr Electron Eng*. 2018;106(5):846-867.

23. Bansal K, Medaglia JD, Bassett DS, Vettel JM, Muldoon SF. Data-driven brain network models differentiate variability across language tasks. *PLoS Comput Biol*. 2018;14(10):1-25.

24. Khambhati AN, Davis KA, Lucas TH, Litt B, Bassett DS. Virtual Cortical Resection Reveals Push-Pull Network Control Preceding Seizure Evolution. *Neuron*. 2016;91(5):1170-1182.

25. Kelly C, De Zubicaray G, Di Martino A, et al. L-dopa modulates functional connectivity in striatal cognitive and motor networks: A double-blind placebo-controlled study. *J Neurosci*. 2009;29(22):7364-7378. doi:10.1523/JNEUROSCI.0810-09.2009

26. McGill ML, Devinsky O, Kelly C, et al. Default mode network abnormalities in idiopathic generalized epilepsy. *Epilepsy Behav*. 2012;23(3):353-359. doi:10.1016/j.yebeh.2012.01.013

27. Khambhati AN, Bassett DS, Oommen BS, et al. Recurring functional interactions predict network architecture of interictal and ictal states in neocortical epilepsy. *eNeuro*. 2017;4(1). doi:10.1523/ENEURO.0091-16.2017

28. Bullmore E, Long C, Suckling J, et al. Colored noise and computational inference in

For Review Only

fMRI time series analysis: resampling methods in time and wavelet domains. *Neuroimage*. 2001;13(6):86. doi:10.1016/s1053-8119(01)91429-6

29. Dauwels J, Eskandar E, Cash S. Localization of seizure onset area from intracranial non-seizure EEG by exploiting locally enhanced synchrony. *Proc 31st Annu Int Conf IEEE Eng Med Biol Soc Eng Futur Biomed EMBC 2009*. Published online 2009:2180-2183.

30. Kramer MA, Kolaczyk ED, Kirsch HE. Emergent network topology at seizure onset in humans. *Epilepsy Res*. 2008;79(2-3):173-186.

31. Antony AR, Alexopoulos A V., González-Martínez JA, et al. Functional connectivity estimated from intracranial EEG predicts surgical outcome in intractable temporal lobe epilepsy. *PLoS One*. 2013;8(10):1-7.

32. Petkov G, Goodfellow M, Richardson MP, Terry JR. A critical role for network structure in seizure onset: A computational modeling approach. *Front Neurol*. 2014;5(DEC):1-7.

33. van Wijk BCM, Stam CJ, Daffertshofer A. Comparing brain networks of different size and connectivity density using graph theory. *PLoS One*. 2010;5(10). doi:10.1371/journal.pone.0013701

34. Ponten SC, Bartolomei F, Stam CJ. Small-world networks and epilepsy: Graph theoretical analysis of intracerebrally recorded mesial temporal lobe seizures. *Clin Neurophysiol*. 2007;118(4):918-927.

35. Onnela JP, Saramäki J, Kertész J, Kaski K. Intensity and coherence of motifs in weighted complex networks. *Phys Rev E - Stat Nonlinear, Soft Matter Phys*. 2005;71(6).

36. Huang LC, Wu PA, Lin SZ, Pang CY, Chen SY. Graph theory and network topological metrics may be the potential biomarker in Parkinson’s disease. *J Clin Neurosci*. 2019;68:235-242.

37. Rubinov M, Sporns O. Complex network measures of brain connectivity: Uses and interpretations. *Neuroimage*. 2010;52(3):1059-1069.

38. Bullmore E, Sporns O. The economy of brain network organization. *Nat Rev Neurosci*. 2012;13(5):336-349.

For Review Only

39. Newman MEJ. Assortative Mixing in Networks. *Phys Rev Lett*. 2002;89(20):1-4.

40. Lim S, Radicchi F, van den Heuvel MP, Sporns O. Discordant attributes of structural and functional brain connectivity in a two-layer multiplex network. *Sci Rep*. 2019;9(1):1-13.

41. de Haan W, Pijnenburg YAL, Strijers RLM, et al. Functional neural network analysis in frontotemporal dementia and Alzheimer's disease using EEG and graph theory. *BMC Neurosci*. 2009;10:1-12.

42. Vaessen MJ, Braakman HMH, Heerink JS, et al. Abnormal modular organization of functional networks in cognitively impaired children with frontal lobe epilepsy. *Cereb Cortex*. 2013;23(8):1997-2006.

43. Godwin D, Barry RL, Marois R. Breakdown of the brain's functional network modularity with awareness. *Proc Natl Acad Sci U S A*. 2015;112(12):3799-3804.

44. Newman MEJ. Modularity and community structure in networks. *Proc Natl Acad Sci U S A*. 2006;103(23):8577-8582.

45. Reichardt J, Bornholdt S. Statistical mechanics of community detection. *Phys Rev E - Stat Nonlinear, Soft Matter Phys*. 2006;74(1):1-14.

46. Restrepo JG, Ott E, Hunt BR. Onset of synchronization in large networks of coupled oscillators. *Phys Rev E - Stat Nonlinear, Soft Matter Phys*. 2005;71(3):1-12.

47. Meghanathan N. Spectral Radius as a Measure of Variation in Node Degree for Complex Network Graphs. *Proc - 7th Int Conf u- e-Service, Sci Technol UNESST 2014*. Published online 2015:30-33.

48. Jamakovic A, Kooij RE, Van Mieghem P, Van Dam ER. Robustness of networks against viruses: The role of the spectral radius. *Proc - 2006 Symp Commun Veh Technol IEEE SCVT 2006; 13th Annu Symp Commun Veh Technol Benelux*. 2006;(3):35-38.

49. Wang Y, Chakrabarti D, Wang C, Faloutsos C. Epidemic spreading in real networks: an eigenvalue viewpoint. *22nd Symp Reliab Distrib Comput Florence, Italy*. Published online 2003.

50. van Dam ER, Kooij RE. The minimal spectral radius of graphs with a given diameter. *Linear Algebra Appl*. 2007;423(2-3):408-419.

For Review Only

51. Wang R, Zhang ZZ, Ma J, Yang Y, Lin P, Wu Y. Spectral properties of the temporal evolution of brain network structure. *Chaos*. 2015;25(12). doi:10.1063/1.4937451

52. Chen G, Duan Z. Network synchronizability analysis: A graph-theoretic approach. *Chaos*. 2008;18(3):1-10. doi:10.1063/1.2965530

53. Jirsa VK, Stacey WC, Quilichini PP, Ivanov AI, Bernard C. On the nature of seizure dynamics. *Brain*. 2014;137(8):2210-2230. doi:10.1093/brain/awu133

54. Gliske S V., Irwin ZT, Chestek C, et al. Variability in the location of high frequency oscillations during prolonged intracranial EEG recordings. *Nat Commun*. 2018;9(1). doi:10.1038/s41467-018-04549-2

55. Efron B, Tibshirani RJ. *An Introduction to the Bootstrap*.; 1994. doi:10.1201/9780429246593

56. Good PI. *Resampling Mehtods: A Practical Guide to Data Analysis*. 3rd ed. Birkhauser Boston, c/o Springer Science and Business Media Inc.; 2005.

57. Thompson B. Effect sizes, confidence intervals, and confidence intervals for effect sizes. *Psychol Sch*. 2007;44(5):423-432. doi:10.1002/pits.20234

58. Kramer MA, Cash SS. Epilepsy as a disorder of cortical network organization. *Neuroscientist*. 2012;18(4):360-372.

59. Jenssen S, Gracely EJ, Sperling MR. How long do most seizures last? A systematic comparison of seizures recorded in the epilepsy monitoring unit. *Epilepsia*. 2006;47(9):1499-1503.

60. Noldus R, Mieghem P Van. Assortativity in complex networks. *J Complex Networks*. 2014;3(4):507-542.

61. Sone D, Matsuda H, Ota M, et al. Graph theoretical analysis of structural neuroimaging in temporal lobe epilepsy with and without psychosis. *PLoS One*. 2016;11(7).

62. Fukushima M, Betzel RF, He Y, et al. Fluctuations between high- and low-modularity topology in time-resolved functional connectivity. *Neuroimage*. 2018;180(July 2017):406-416.

63. Chavez M, Valencia M, Navarro V, Latora V, Martinerie J. Functional modularity of background activities in normal and epileptic brain networks. *Phys Rev Lett*.

For Review Only

2010;104(11).

64. Goulas A, Betzel RF, Hilgetag CC. Spatiotemporal ontogeny of brain wiring. *Sci Adv*. 2019;5(6).

65. Chen H, Zhao X, Liu F, Xu S, Lu W. Optimizing interconnections to maximize the spectral radius of interdependent networks. *Phys Rev E*. 2017;95(3):1-15.

66. Lainscsek C, Rungratsameetaweemana N, Cash SS, Sejnowski TJ. Cortical chimera states predict epileptic seizures. *Chaos*. 2019;29(12).

67. Blumenfeld H. Cellular and network mechanisms of electrographic seizures. *Epilepsia*. 2005;46(9):21-33.

68. Bansal K, Medaglia JD, Bassett DS, Vettel JM, Muldoon SF. Data-driven brain network models differentiate variability across language tasks. *PLoS Comput Biol*. 2018;14(10):1-25. doi:10.1371/journal.pcbi.1006487

69. Xia CH, Ma Z, Ciric R, et al. Linked dimensions of psychopathology and connectivity in functional brain networks. *Nat Commun*. 2018;9(1):1-14.

70. Satterthwaite TD, Feczko E, Kaczkurkin AN, Fair DA. Parsing Psychiatric Heterogeneity Through Common and Unique Circuit-Level Deficits. *Biol Psychiatry*. 2020;88(1):4-5.

71. Cornblath EJ, Li HL, Changolkar L, et al. Computational modeling of tau pathology spread reveals patterns of regional vulnerability and the impact of a genetic risk factor. 2021;(June):1-16.

72. Frankle WG, Narendran R. Distinguishing Schizophrenia Subtypes: Can Dopamine Imaging Improve the Signal-to-Noise Ratio? *Biol Psychiatry*. 2020;87(3):197-199.

73. Karalunas SL, Nigg JT. Heterogeneity and Subtyping in Attention-Deficit/Hyperactivity Disorder—Considerations for Emerging Research Using Person-Centered Computational Approaches. *Biol Psychiatry*. 2020;88(1):103-110.

For Review Only

Figure legends

Figure 1 Emergence of distinct seizure propagation patterns in a single patient. (**A**) During a clinical monitoring procedure to identify a seizure onset zone of patients with medication-refractory (drug-resistant) epilepsy, intracranial recording electrodes are implanted. (**B**) Intracranial activity during two sample seizures recorded from a single patient, which exhibit distinct propagation dynamics. On the left, the seizure activity originates from a few electrodes and persists in the localized area within a single hemisphere (i.e., focal seizure that remains focal). On the right, the seizure activity originates from a few electrodes but diffuses bilaterally to involve electrodes in both hemispheres. This type of seizure is known as focal to bilateral tonic-clonic seizure or focal seizure with bilateral spread. Despite their similarly focal origin, these seizure types induce drastically differential clinical manifestations such that focal to bilateral tonic-clonic seizures are associated with more severe cognitive and behavioral deficits. We hypothesize that such heterogeneity in seizure dynamics emerges from distinct and measurable temporal alterations in the functional brain connectivity networks.

Figure 2 Schematic of graph theoretical analysis of functional brain dynamics. (**A**) Locations of implanted intracranial electrodes of a sample patient. (**B**) We use electrocorticography (ECoG) time-series data from all intracranial electrodes from each patient recorded during a clinical monitoring procedure to locate the seizure onset zone. We estimate the instantaneous functional connectivity of the underlying brain network by computing pairwise correlations of ECoG data across electrodes in a sliding-window manner. The magnitudes of these correlations (restricted between 0 and 1) reflect the strength of connections between each pair of electrodes and are represented by a weighted adjacency or connectivity matrix (see Materials and methods). (**C**) To investigate time-varying changes in the functional brain connectivity during temporal evolution of each seizure type, we compute a series of connectivity matrices over time and use these as bases to construct functional connectivity networks. (**D**) A schematic of sample constructed networks, consisting of nodes (electrodes) and edges (connection strength). To quantify alterations within these complex networks over time, we evaluate changes of a series of graph theoretical attributes which describe globally- and locally- defined properties of the constructed networks.

For Review Only

Figure 3 Clustering coefficient and characteristic path length track diffusivity of seizure activity. Focal to bilateral tonic-clonic seizures (*n* = 18) display simultaneous increase in the clustering coefficient (CC) and decrease in the characteristic path length (PL) than focal seizures that remain localized within one hemisphere (*n* = 49). (**A**) Averages of CC associated with each seizure type are plotted separately for preictal, ictal (during seizure), and postictal periods. CC of interictal (seizure-free) networks are also plotted as a baseline. (**B**) PL is plotted in the same manner. (**C**) CC of focal to bilateral tonic-clonic seizures is higher than that of focal seizures that remain focal, 2.25-10 minutes after seizure onset. (**D**) PL of focal to bilateral tonic-clonic seizures is lower than that of focal seizures that remain focal, 2-10 minutes after seizure onset. Statistical comparisons of network measures as a function of seizure types were computed through a bootstrapping procedure where the underlying data distribution of each network measure was resampled at the level of individual seizures to established 95% confidence intervals (CIs). Error bars indicate 95% CIs across individual seizures in each condition and solid bars show resampled $P < 0.05$.

Figure 4 Various features of functional connectivity networks display distinct temporal changes as a function of seizure propagation dynamics. Left panels illustrate a series of graph theoretical measures computed from networks of focal seizures that remain localized (*n* = 49) and from networks of focal to bilateral tonic-clonic seizures (*n* = 18). The time-varying differences observed in each of these features as a function of seizure types are plotted in the corresponding right panels. (**A**) The density of focal to bilateral tonic-clonic seizures is higher than that of focal seizures that remain focal, 1.75-10 minutes after seizure onset. (**B**) The assortativity, a measure of network robustness, is lower for focal to bilateral tonic-clonic seizures relative to focal seizures that remain focal, 7.5-9.25 minutes after seizure onset. (**C**) The modularity, which captures efficient network integration and global segregation, is higher for focal to bilateral tonic-clonic seizures when compared to focal seizures that remain focal during temporal windows between 14.75 to 5.75 minutes before seizure onset and 0.75-1.50 minutes after the onset. (**D**) The spectral radius, which relates to the global spread of synchronization in a network, is also higher for focal to bilateral tonic-clonic seizures as compared to focal seizures that remain focal during temporal windows between 14.75 to 3.75 minutes before seizure onset and 0.75-1.50 minutes after the onset. (**E**) The synchronizability, which estimates the propensity of information to diffuse in a network, shows an increasing trend post seizure onset for unconstrained seizure dynamics. However, no statistically significant differences were observed in synchronizability across seizure types. Statistical

For Review Only

comparisons of network measures as a function of seizure types were computed through a bootstrapping procedure where the underlying data distribution of each network measure was resampled at the level of individual seizures to established 95% confidence intervals (CIs). Error bars indicate 95% CIs across individual seizures in each condition and solid bars show resampled $P < 0.05$.

Figure 5 Summary of graph theoretical attributes probed across seizure types. The network features investigated can be categorized into 2 groups based on the temporal windows at which differential changes in these features emerge as a function of seizure propagation patterns. The time windows where such differences are observed are plotted separately for each of the network measures (resampled $P < 0.05$). Global features, i.e., the modularity and spectral radius, primarily capture network alterations that occur prior to and shortly after seizure onset. In contrast, the density, assortativity, clustering coefficient and characteristic path length characterize post-onset network reconfigurations induced by different types of propagation dynamics.

Figure 6 Distinct patterns of network properties across seizure types at a single-seizure level. Modularity and spectral radius extracted from networks associated with three seizures that share similar onset regions recorded from a sample patient. Seizure 1 and seizure 3 are categorized by an epileptologist as focal seizures that remain focal (sample recordings of seizure 1 is illustrated in Fig. 1B (left)), whereas seizure 2 is categorized as a focal to bilateral tonic-clonic seizure (sample recordings of seizure 2 is also illustrated in Fig. 1B (right)). Seizure 1 and seizure 3 exhibit similar patterns of modularity and spectral radius overtime, and these temporal dynamics differ from those of seizure 2. The grey shaded region highlights the robust signatures associated with the bilateral spread of a focal seizure (i.e., increase in modularity and decrease in spectral radius) and is followed by network changes which are likely seizure- and/or patient-specific.

For Review Only

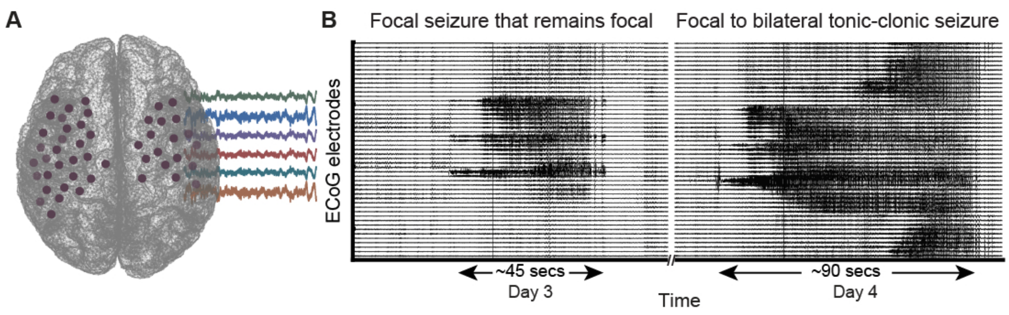

Figure 1 Emergence of distinct seizure propagation patterns in a single patient. (A) During a clinical monitoring procedure to identify a seizure onset zone of patients with medication-refractory (drug-resistant) epilepsy, intracranial recording electrodes are implanted. (B) Intracranial activity during two sample seizures recorded from a single patient, which exhibit distinct propagation dynamics. On the left, the seizure activity originates from a few electrodes and persists in the localized area within a single hemisphere (i.e., focal seizure that remains focal). On the right, the seizure activity originates from a few electrodes but diffuses bilaterally to involve electrodes in both hemispheres. This type of seizure is known as focal to bilateral tonic-clonic seizure or focal seizure with bilateral spread. Despite their similarly focal origin, these seizure types induce drastically differential clinical manifestations such that focal to bilateral tonic-clonic seizures are associated with more severe cognitive and behavioral deficits. We hypothesize that such heterogeneity in seizure dynamics emerges from distinct and measurable temporal alterations in the functional brain connectivity networks.

469x148mm (59 x 59 DPI)

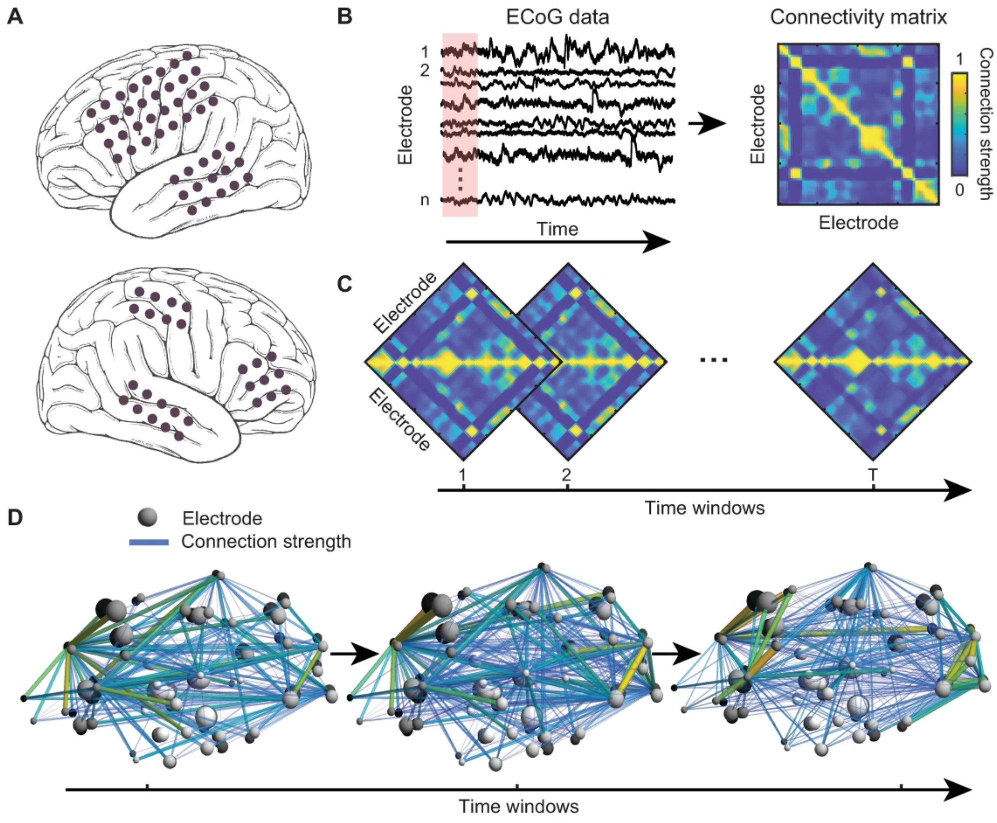

Figure 2 Schematic of graph theoretical analysis of functional brain dynamics. (A) Locations of implanted intracranial electrodes of a sample patient. (B) We use electrocorticography (ECoG) time-series data from all intracranial electrodes from each patient recorded during a clinical monitoring procedure to locate the seizure onset zone. We estimate the instantaneous functional connectivity of the underlying brain network by computing pairwise correlations of ECoG data across electrodes in a sliding-window manner. The magnitudes of these correlations (restricted between 0 and 1) reflect the strength of connections between each pair of electrodes and are represented by a weighted adjacency or connectivity matrix (see Materials and methods). (C) To investigate time-varying changes in the functional brain connectivity during temporal evolution of each seizure type, we compute a series of connectivity matrices over time and use these as bases to construct functional connectivity networks. (D) A schematic of sample constructed networks, consisting of nodes (electrodes) and edges (connection strength). To quantify alterations within these complex networks over time, we evaluate changes of a series of graph theoretical attributes which describe globally- and locally- defined properties of the constructed networks.

184x151mm (600 x 600 DPI)

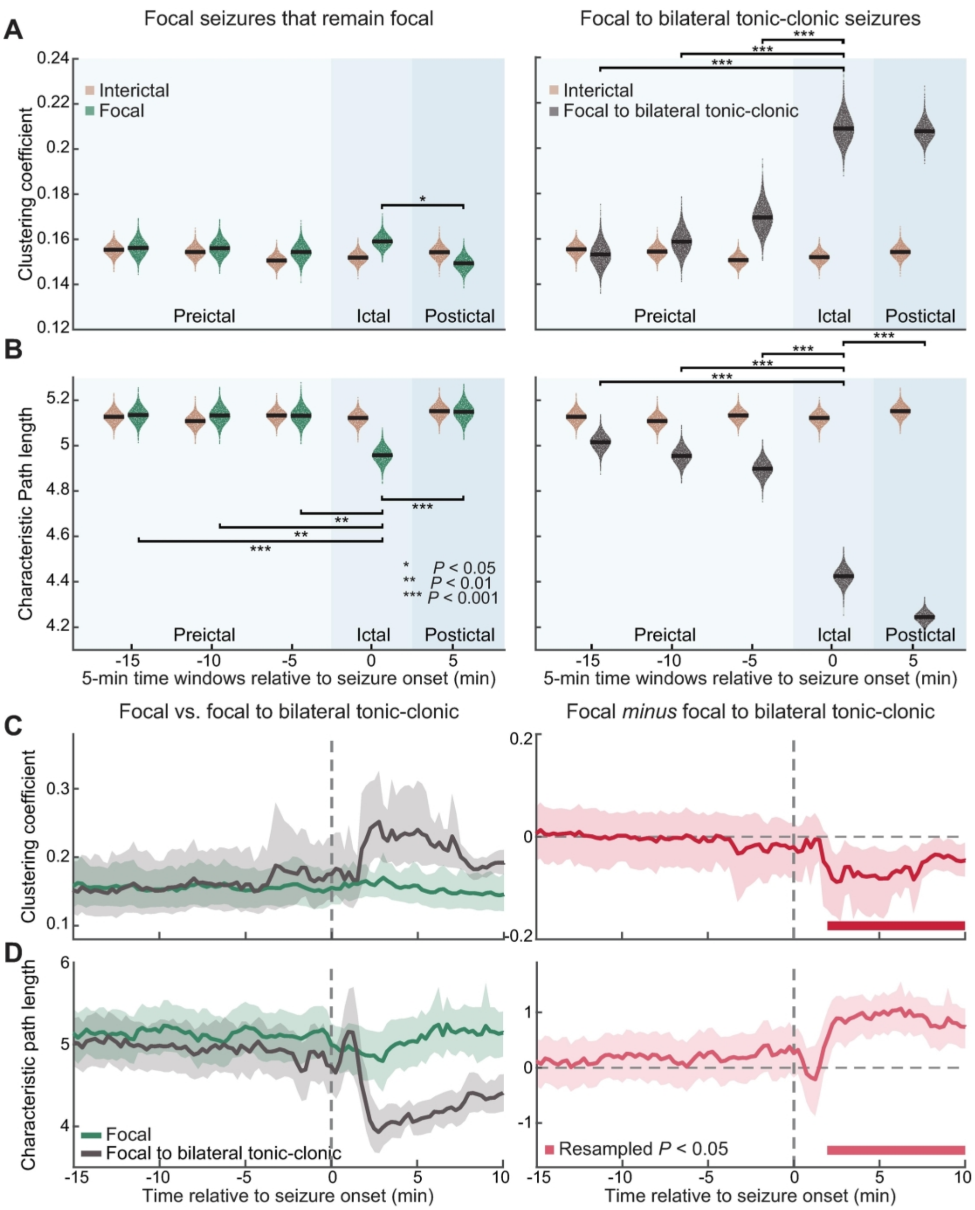

Figure 3 Clustering coefficient and characteristic path length track diffusivity of seizure activity. Focal to bilateral tonic-clonic seizures (n = 18) display simultaneous increase in the clustering coefficient (CC) and decrease in the characteristic path length (PL) than focal seizures that remain localized within one hemisphere (n = 49). (A) Averages of CC associated with each seizure type are plotted separately for preictal, ictal (during seizure), and postictal periods. CC of interictal (seizure-free) networks are also plotted as a baseline. (B) PL is plotted in the same manner. (C) CC of focal to bilateral tonic-clonic seizures is higher than that of focal seizures that remain focal, 2.25-10 minutes after seizure onset. (D) PL of focal to bilateral tonic-clonic seizures is lower than that of focal seizures that remain focal, 2-10 minutes after seizure onset. Statistical comparisons of network measures as a function of seizure types were computed through a bootstrapping procedure where the underlying data distribution of each network measure was resampled at the level of individual seizures to established 95% confidence intervals (CIs). Error bars indicate 95% CIs across individual seizures in each condition and solid bars show resampled P < 0.05.

182x226mm (300 x 300 DPI)

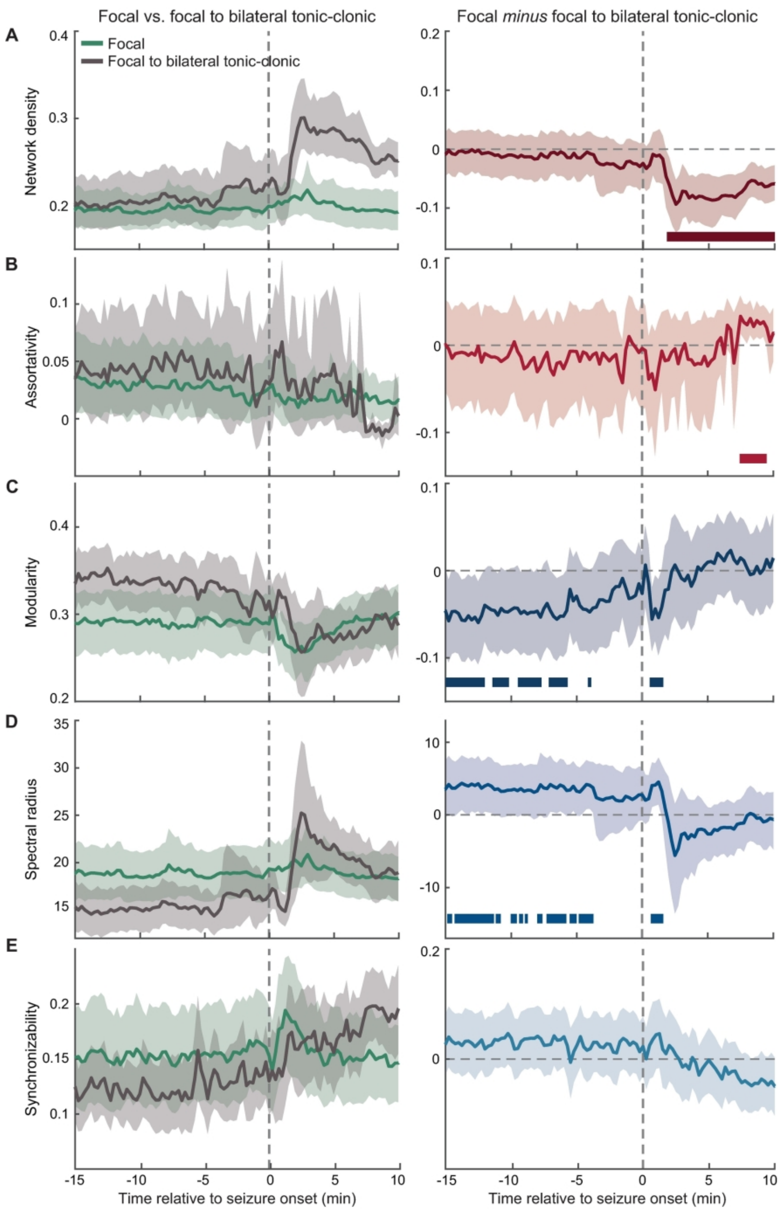

Figure 4 Various features of functional connectivity networks display distinct temporal changes as a function of seizure propagation dynamics. Left panels illustrate a series of graph theoretical measures computed from networks of focal seizures that remain localized (n = 49) and from networks of focal to bilateral tonic-clonic seizures (n = 18). The time-varying differences observed in each of these features as a function of seizure types are plotted in the corresponding right panels. (A) The density of focal to bilateral tonic-clonic seizures is higher than that of focal seizures that remain focal, 1.75-10 minutes after seizure onset. (B) The assortativity, a measure of network robustness, is lower for focal to bilateral tonic-clonic seizures relative to focal seizures that remain focal, 7.5-9.25 minutes after seizure onset. (C) The modularity, which captures efficient network integration and global segregation, is higher for focal to bilateral tonic-clonic seizures when compared to focal seizures that remain focal during temporal windows between 14.75 to 5.75 minutes before seizure onset and 0.75-1.50 minutes after the onset. (D) The spectral radius, which relates to the global spread of synchronization in a network, is also higher for focal to bilateral tonic-clonic seizures as compared to focal seizures that remain focal during temporal windows between 14.75 to 3.75 minutes before seizure onset and 0.75-1.50 minutes after the onset. (E) The synchronizability, which estimates the

propensity of information to diffuse in a network, shows an increasing trend post seizure onset for unconstrained seizure dynamics. However, no statistically significant differences were observed in synchronizability across seizure types. Statistical comparisons of network measures as a function of seizure types were computed through a bootstrapping procedure where the underlying data distribution of each network measure was resampled at the level of individual seizures to established 95% confidence intervals (CIs). Error bars indicate 95% CIs across individual seizures in each condition and solid bars show resampled $P < 0.05$.

178x277mm (300 x 300 DPI)

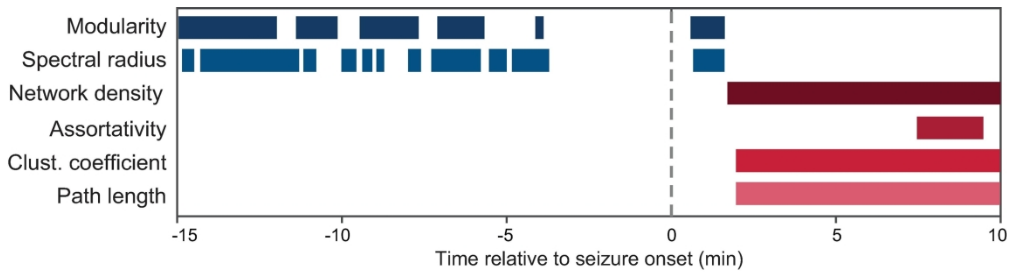

Figure 5 Summary of graph theoretical attributes probed across seizure types. The network features investigated can be categorized into 2 groups based on the temporal windows at which differential changes in these features emerge as a function of seizure propagation patterns. The time windows where such differences are observed are plotted separately for each of the network measures (resampled $P < 0.05$). Global features, i.e., the modularity and spectral radius, primarily capture network alterations that occur prior to and shortly after seizure onset. In contrast, the density, assortativity, clustering coefficient and characteristic path length characterize post-onset network reconfigurations induced by different types of propagation dynamics.

182x48mm (300 x 300 DPI)

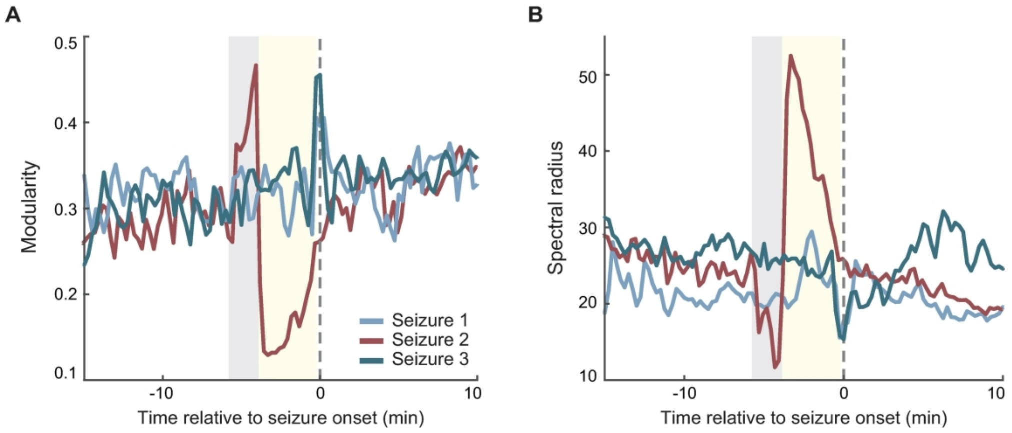

Figure 6 Distinct patterns of network properties across seizure types at a single-seizure level. Modularity and spectral radius extracted from networks associated with three seizures that share similar onset regions recorded from a sample patient. Seizure 1 and seizure 3 are categorized by an epileptologist as focal seizures that remain focal (sample recordings of seizure 1 is illustrated in Fig. 1B (left)), whereas seizure 2 is categorized as a focal to bilateral tonic-clonic seizure (sample recordings of seizure 2 is also illustrated in Fig. 1B (right)). Seizure 1 and seizure 3 exhibit similar patterns of modularity and spectral radius overtime, and these temporal dynamics differ from those of seizure 2. The grey shaded region highlights the robust signatures associated with the bilateral spread of a focal seizure (i.e., increase in modularity and decrease in spectral radius) and is followed by network changes which are likely seizure- and/or patient-specific.

191x82mm (300 x 300 DPI)

Brain network dynamics codify heterogeneity in seizure evolution

Nuttida Rungratsameetaweemana, Claudia Lainscsek, Sydney S. Cash, Javier O. Garcia,
Terrence J. Sejnowski[†], Kanika Bansal[*,†]

Table I Patient Profiles

Patient	Sex	Age at onset/ surgery	Etiology	Seizure type (#)	Seizure onset zone	Electrode type (#)	Resection areas	Outcome
1	F	15/46	Dysplasia	focal to bilateral (5)	Anterior temporal	Depths/Grids (113)	Right anterior temporal	I
2	F	42/55	n.a	focal to bilateral (3)	Temporal	Depths (56)	None	I
3	F	17/45	n.a	focal (1); focal to bilateral (2)	Temporal	Depths (40)	None	n.a
4	M	8/23	n.a	focal (10)	Frontal	Depths (80)	None	III
5	M	14/35	n.a	focal (9); focal to bilateral (2)	Temporal	Depths (80)	Right anterior temporal	n.a
6	F	12/32	n.a	focal (15)	Temporal	Depths (112)	Right anterior temporal	II
7	F	7/23	n.a	focal (6)	Frontal	Depths (80)	Left frontal	IV
8	F	10/27	n.a	focal (1)	Unknown	Depths (80)	Left frontal	IV
9	F	8/19	MTS	focal (1)	Anterior temporal	Grids (60)	Left anterior temporal	III
10	F	14/31	n.a	focal to bilateral (2)	Temporal	Depths (48)	Right anterior temporal	I
11	F	1/21	Stroke	focal (2)	Temporal	Depths (118)	Left temporal	IV
12	F	9/42	n.a	focal (2)	Frontal	Depths (76)	None	II
13	M	39/47	n.a	focal to bilateral (3)	Posterior temporal	Depths (48)	Right temporal	I
14	F	50/59	n.a	focal (2); focal to bilateral (1)	Posterior temporal	Depths (80)	Left temporal	I

For Review Only

Clinical characteristics of the patients. For each patient, we report sex, age at first reported seizures onset, as well as age at the monitoring phase and surgery. We also report the seizure etiology, which was clinically determined through medical history, imaging, and long-term invasive monitoring. Additionally, we indicate the number of observed seizures associated with the two different types of seizures which originated from one hemisphere: focal seizures that remained localized within the same hemisphere (focal seizure; focal) and focal seizures that propagate bilaterally to both hemispheres (focal to bilateral tonic-clonic seizure; focal to bilateral). Surgical outcome (outcome) was based on Engel score: seizure freedom to no improvement (I-V), and no follow-up (NF). Legend: M = male; F = female; MTS = Mesial Temporal Sclerosis; n.a = not applicable.

For Review Only

Brain network dynamics codify heterogeneity in seizure evolution

Nuttida Rungratsameetaweemana, Claudia Lainscsek, Sydney S. Cash, Javier O. Garcia, Terrence J. Sejnowski[†], Kanika Bansal[*,†]

Supplementary Figures

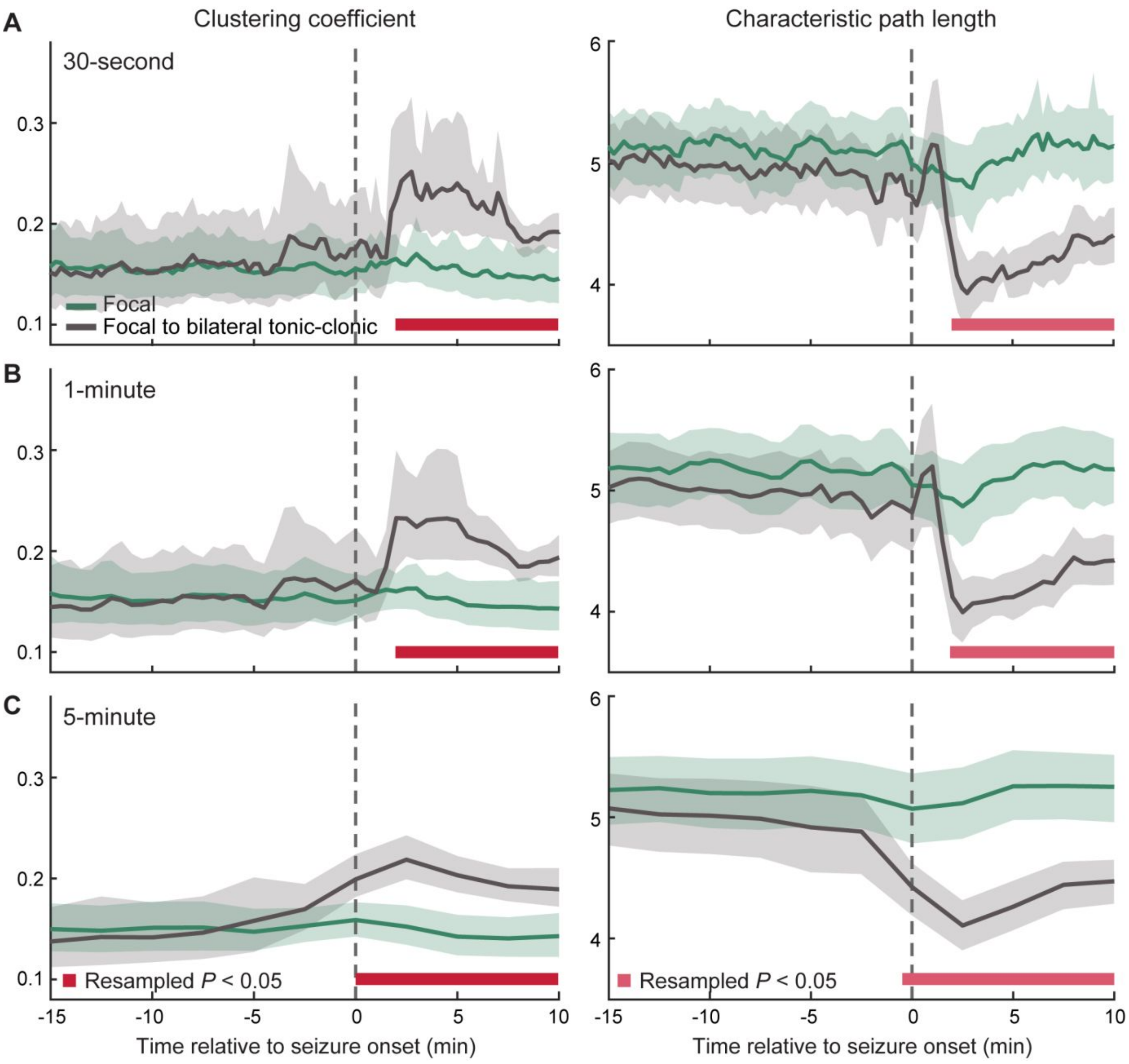

Fig. S1. The effects of temporal smoothing on sample network measures. Prior to computing graph theoretical features, a temporal smoothing parameter is first applied to the functional connectivity networks to approximate stationary pairwise connection strength signals. (**A**) The clustering coefficient (CC) and characteristic path length (PL) as computed after a smoothing parameter of 30 seconds has been applied to the adjacency matrices in a time-resolved fashion. These network measures are calculated separately for focal seizures

that remain focal ($n = 49$) and focal to bilateral tonic-clonic seizures ($n = 18$). (**B**) Same network measures computed based on a temporal smoothing parameter of 1 minute. (**C**) Same network measures computed based on a temporal smoothing parameter of 5 minutes. CC of focal to bilateral tonic-clonic seizures are higher than that of focal seizures that remain focal (2-10 minutes; 2-10 minutes; seizure onset to 10 minutes after seizure onset for a smoothing parameter of 30 seconds, 1 minute, and 5 minutes, respectively). PL of focal to bilateral tonic-clonic seizures are lower than that of focal seizures that remain focal (2-10 minutes; 2-10 minutes; 1 minutes before seizure onset to 10 minutes after seizure onset for a smoothing parameter of 30 seconds, 1 minute, and 5 minutes, respectively). Statistical comparisons of network measures as a function of seizure types were computed through a bootstrapping procedure where the underlying data distribution of each network measure was resampled at the level of individual seizures to established 95% confidence intervals (CIs). Error bars indicate 95% CIs across individual seizures in each condition and solid bars show resampled $P < 0.05$. All reported results in the main text are achieved based on a smoothing parameter of 30 seconds and the comparisons illustrated here suggest that our reported findings are robust and largely unaffected by the choice of this parameter.

For Review Only

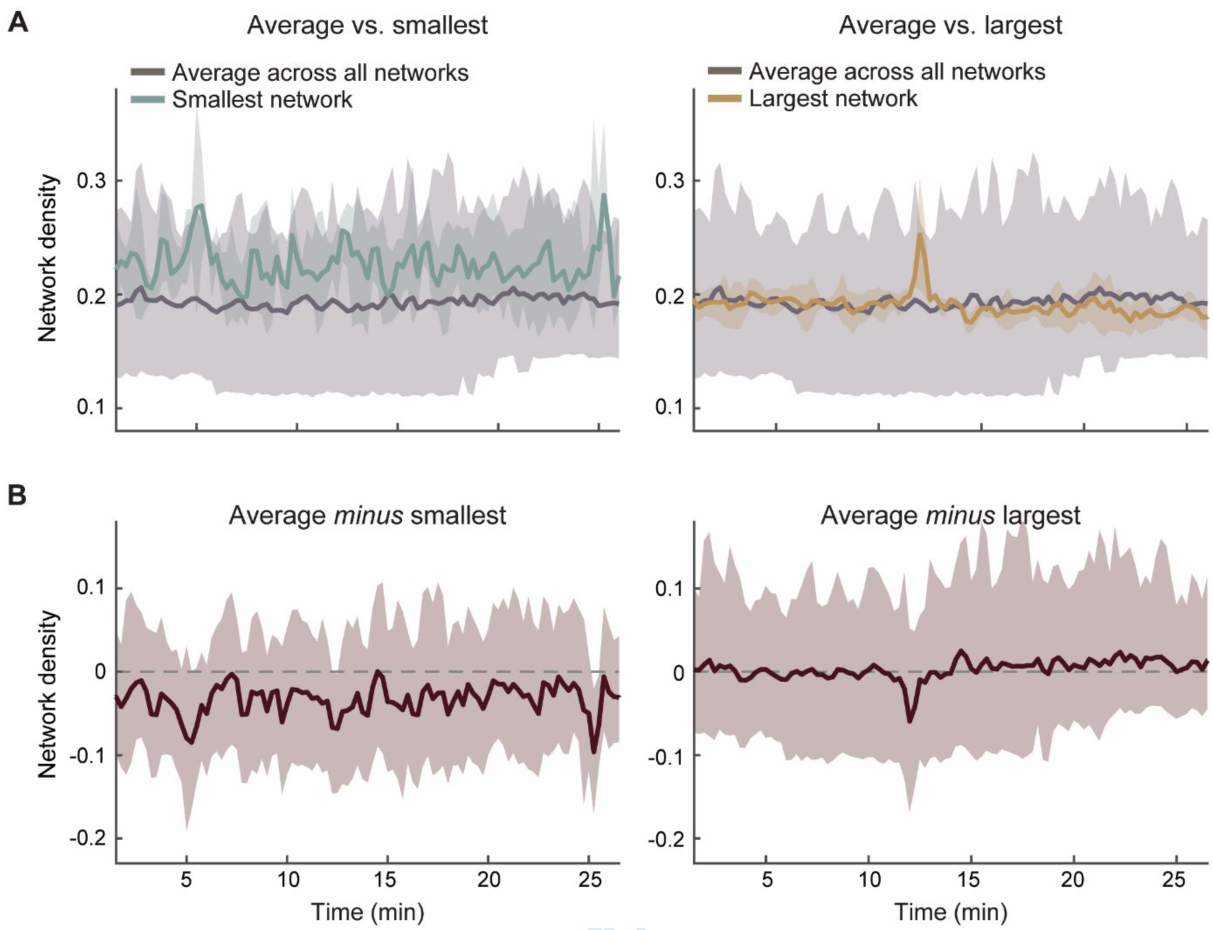

Fig. S2. Density extracted from interictal networks of different sizes. The density of smallest (i.e., 40 nodes; left panels) and largest (i.e., 118 nodes; right panels) is illustrated with the average density computed across all functional network. The density of the smallest interictal network did not differ from the average density. Similarly, the density of the largest interictal network also did not differ from that average density computed across interictal networks of different sizes. Statistical comparisons of density as a function of network sizes were computed through a bootstrapping procedure where the underlying data distribution of each network size was resampled at the level of individual seizures to established 95% confidence intervals (CIs).

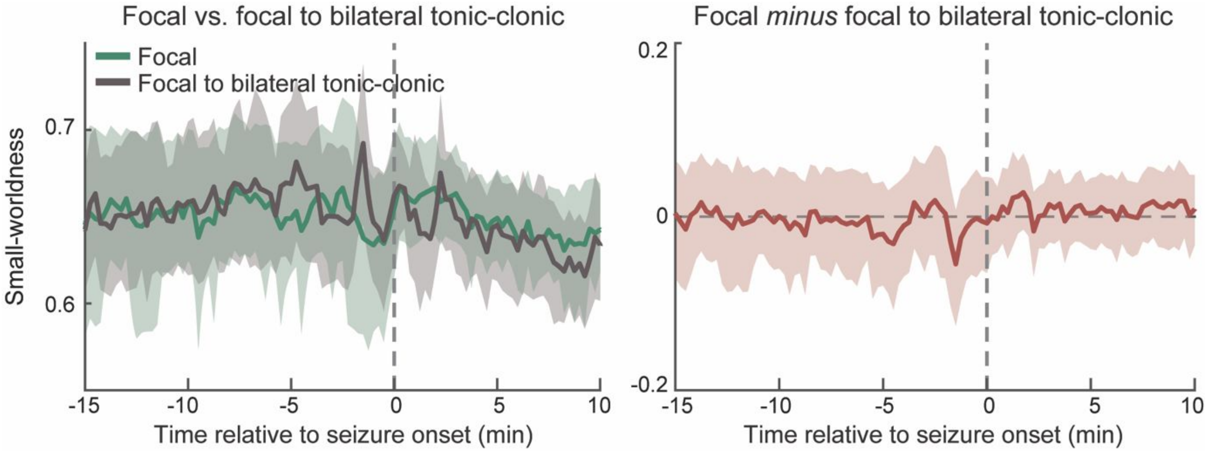

Fig. S3. Small-world characteristics of focal seizure subtypes. We computed small-worldness as the ratio of CC and PL relative to that of a random network. Random networks were generated at each time point by redistributing the edge weights of the original network such that the average degree is preserved. Focal to bilateral tonic-clonic seizures exhibit similar temporal patterns of small-world characteristics as focal seizures that remain localized. Statistical comparisons of small-world characteristics as a function of seizure types were computed through a bootstrapping procedure where the underlying data distribution was resampled at the level of individual seizures to established 95% confidence intervals (CIs).

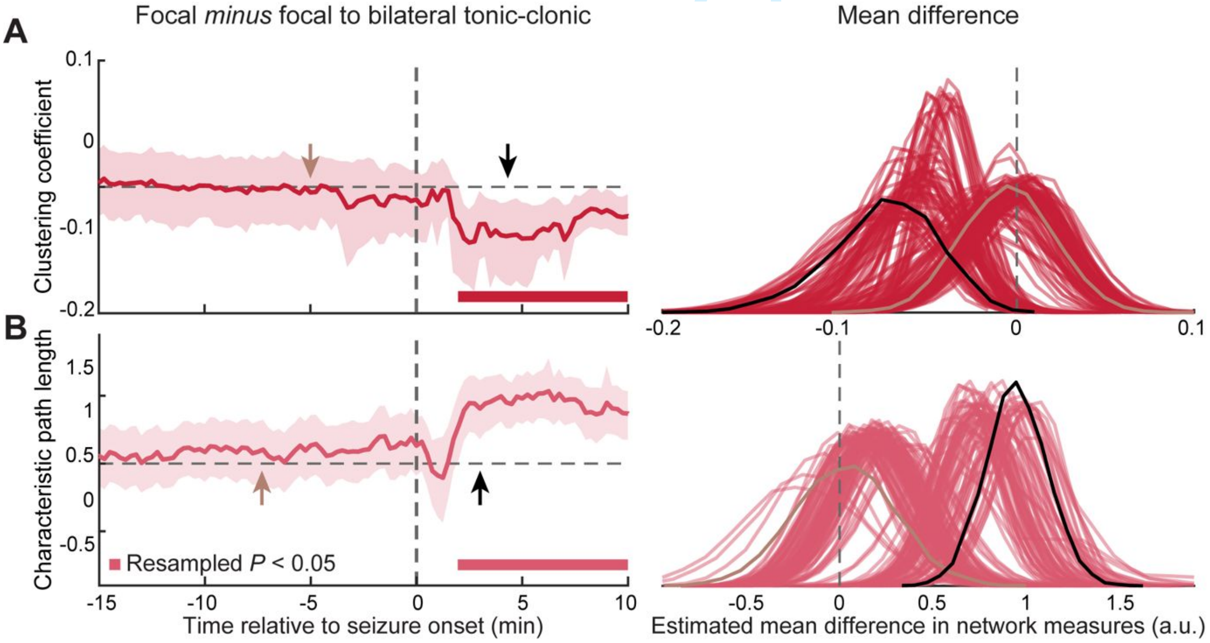

Fig. S4. Effect sizes of seizure-type specific reconfigurations in clustering coefficient and characteristic path length. To visualize the effect sizes associated with seizure-type specific

differences in clustering coefficient (**A**) and characteristic path length (**B**) overtime (left panels), the bootstrapped distributions associated with the mean difference of individual timepoints are plotted (right panels). For each of the network measure, the brown and black arrows indicated a sample timepoint where the mean difference between seizure types were not significantly different and significantly different from the bootstrapped null difference of zero, respectively. The distributions of these mean differences are illustrated in the corresponding right panel. Statistical comparisons of network measures as a function of seizure types were computed through a bootstrapping procedure where the underlying data distribution of each network measure was resampled at the level of individual seizures to establish 95% confidence intervals (CIs). Error bars indicate 95% CIs across individual seizures in each condition and solid bars show resampled $P < 0.05$.

For Review Only

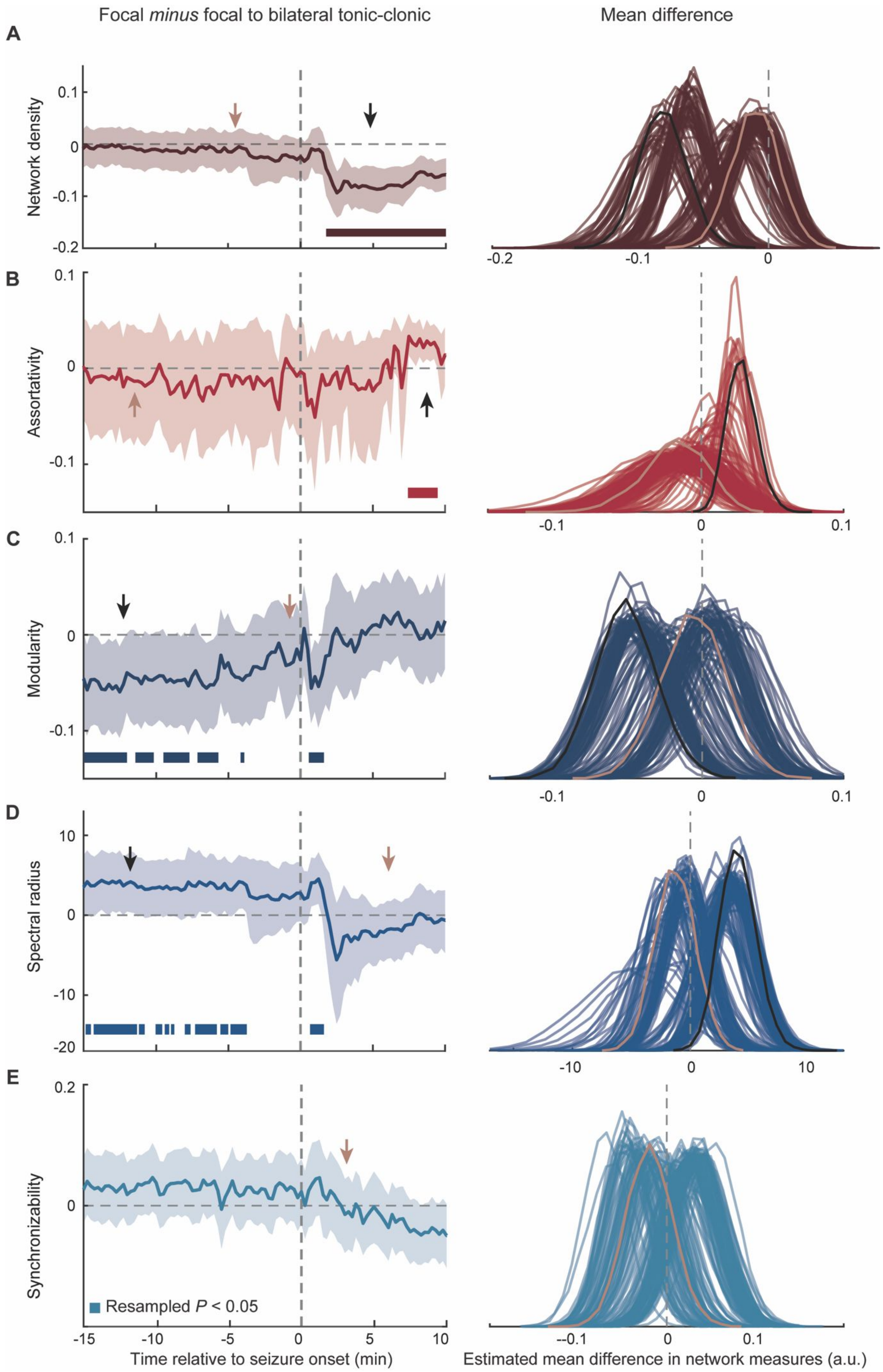

Focal minus focal to bilateral tonic-clonic
Mean difference
A
Network density
B
Assortativity
C
Modularity
D
Spectral radius
E
Synchronizability
Resampled P < 0.05
Time relative to seizure onset (min)
Estimated mean difference in network measures (a.u.)

Fig. S5. Effect sizes of seizure-type specific reconfigurations in various features of functional connectivity networks. Left panels illustrate the seizure-type specific differences in network density (**A**), associativity (**B**), modularity (**C**), spectral radius (**D**), and synchronizability (**E**). The bootstrapped distributions associated with the mean difference of individual timepoints are plotted in the corresponding right panels. For each of the network measure, the brown and black arrows indicated a sample timepoint where the mean difference between seizure types were not significantly different and significantly different from the bootstrapped null difference of zero, respectively. The distributions of these mean differences are illustrated in the corresponding right panel. Statistical comparisons of network measures as a function of seizure types were computed through a bootstrapping procedure where the underlying data distribution of each network measure was resampled at the level of individual seizures to establish 95% confidence intervals (CIs). Error bars indicate 95% CIs across individual seizures in each condition and solid bars show resampled $P < 0.05$.

For Review Only

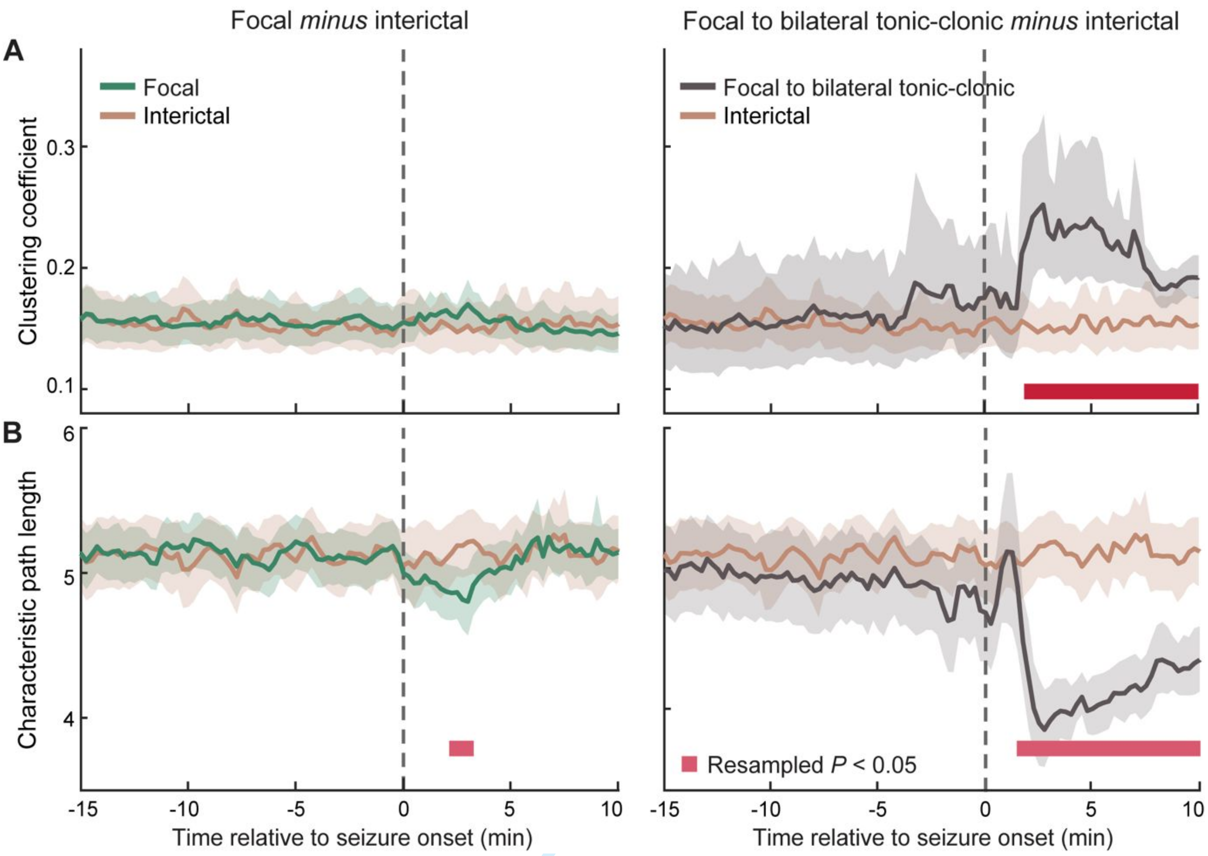

Fig. S6. **Clustering coefficient and characteristic path length of focal seizures with constrained and unconstrained propagation dynamics relative to those of interictal periods.** (**A**) The clustering coefficient (CC) of focal seizures that remain focal (n = 49) is not different from that of interictal networks (n = 49). CC of focal to bilateral tonic-clonic seizures (n = 18) is higher than that of interictal networks, 2-10 minutes after seizure onset. (**B**) PL of focal seizures that remain focal is lower than that of interictal networks, 2.50-3.50 minutes after seizure onset. PL of focal to bilateral tonic-clonic seizures is lower than that of interictal networks, 1.75-10 minutes after seizure onset. Statistical comparisons of network measures as a function of seizure types were computed through a bootstrapping procedure where the underlying data distribution of each network measure was resampled at the level of individual seizures (or interictal epochs) to established 95% confidence intervals (CIs). Error bars indicated 95% CIs across individual seizures (or interictal epochs) in each condition and solid bars show resampled $P < 0.05$.

view Only

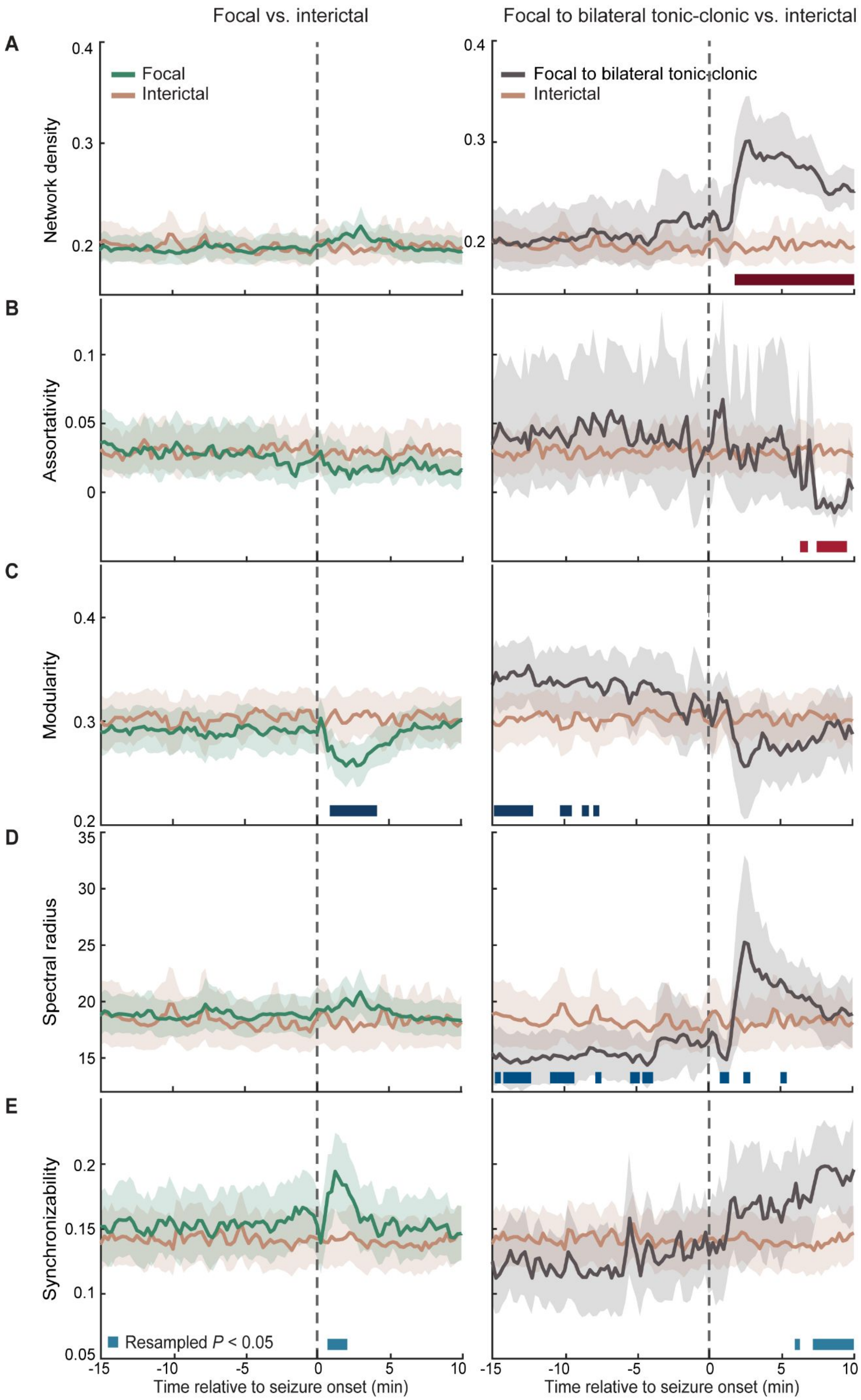
Focal vs. interictal
Focal to bilateral tonic-clonic vs. interictal
A
B
C
D
E
Focal
Interictal
Focal to bilateral tonic-clonic
Interictal
Network density
Assortativity
Modularity
Spectral radius
Synchronizability
Resampled P < 0.05
Time relative to seizure onset (min)
Time relative to seizure onset (min)

Fig. S7. Network features of focal seizures with constrained and unconstrained dynamics in comparison to those of interictal activity. (A) The density of focal seizures that remain focal (n = 49) is not different from that of interictal networks (n = 49). The density of focal to bilateral tonic-clonic seizures (n = 18) is higher than that of interictal networks, 1.75-10 minutes after seizure onset. **(B)** The assortativity of focal seizures that remain focal is not different from that of interictal networks. The assortativity of focal to bilateral tonic-clonic seizures is higher than that of interictal networks, 6.50-7 and 7.50-9.75 minutes after seizure onset. **(C)** The modularity of focal seizures that remain focal is lower than that of inter-ictal networks, 1-4.25 minutes after seizure onset. The modularity of focal to bilateral tonic-clonic seizures is higher than that of interictal networks, 14.75-12, 10.25-10, 9.75-9.5, and 8.5-8.25 minutes before seizure onset **(D)** The spectral radius of focal seizures that remain focal is not different from that of interictal networks. The spectral radius of focal to bilateral tonic-clonic seizures is lower than that of interictal networks, 14.75-14.50, 14-12.25, 11-9.5, 7.75-7.50, 5.25-4.75, and 4.50-3.75 minutes before seizure onset; and 1-1.50, 2.50-3, and 5-5.50 minutes after seizure onset. **(E)** The synchronizability of focal seizures that remain focal is higher than that of interictal networks, 0.75-2 minutes after seizure onset. The synchronizability of focal to bilateral tonic-clonic seizures is higher than that of interictal networks, 6-6.25 and 7.25-10 minutes after seizure onset. Statistical comparisons of network measures as a function of seizure types were computed through a bootstrapping procedure where the underlying data distribution of each network measure was resampled at the level of individual seizures (or interictal epochs) to established 95% confidence intervals (CIs). Error bars indicated 95% CIs across individual seizures (or interictal epochs) in each condition and solid bars show resampled $P < 0.05$.

For Review Only
