# Supplementary material for: Brain network dynamics codify heterogeneity in seizure evolution
Source: Brain Commun. 2022 Sep 16;4(5):fcac234. doi: 10.1093/braincomms/fcac234 (PMC9527667; doi:10.1093/braincomms/fcac234)
Supplement: fcac234_Supplementary_Data [file fcac234_supplementary_data.zip › Supplementary Material..docx]

Brain network dynamics codify heterogeneity in seizure evolution

Nuttida Rungratsameetaweemana, Claudia Lainscsek, Sydney S. Cash, Javier O. Garcia,

Terrence J. Sejnowski^†^, Kanika Bansal^*,†^

Supplementary Figures

**
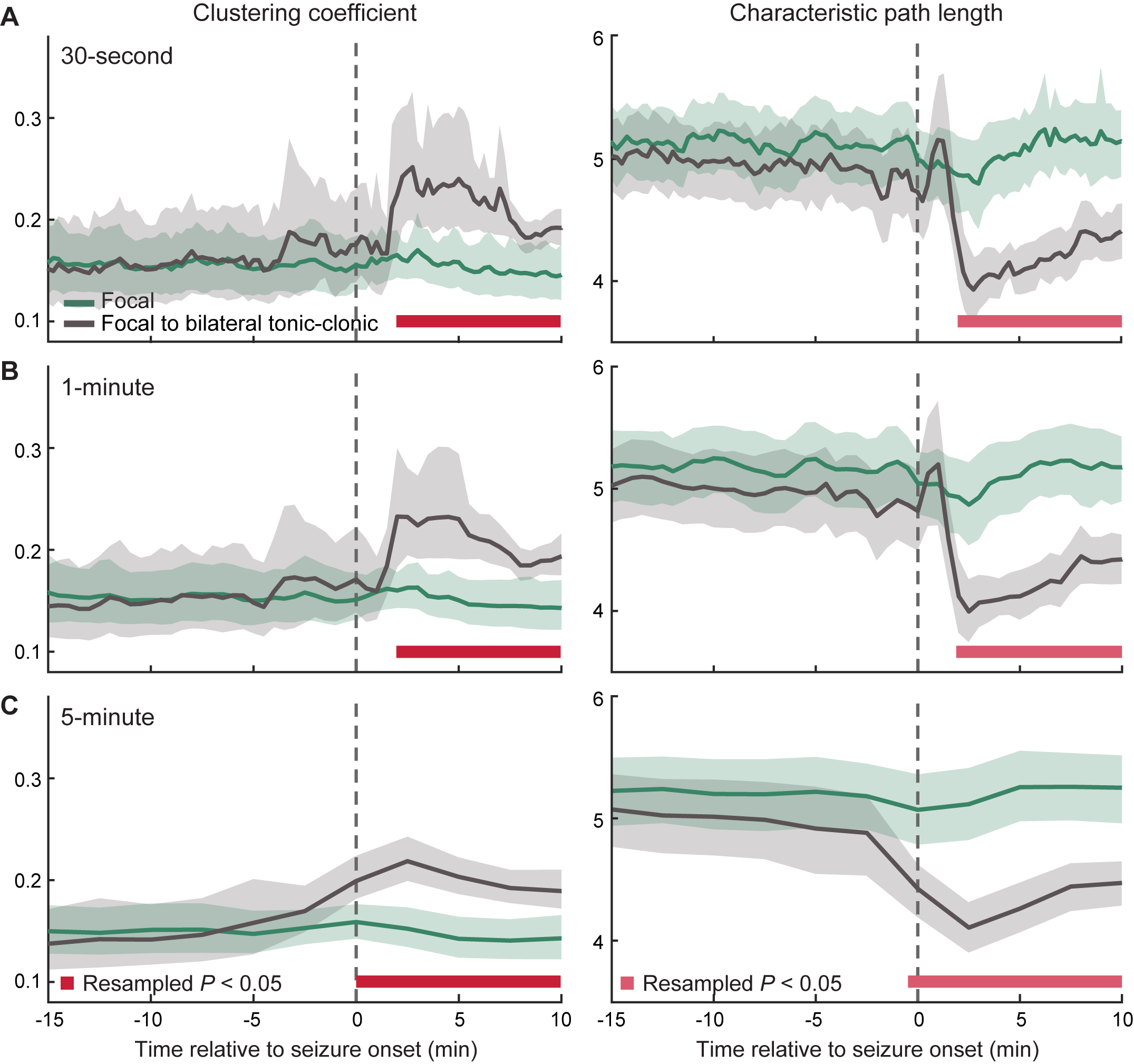
**

**Supplementary Figure 1. The effects of temporal smoothing on sample network measures.** Prior to computing graph theoretical features, a temporal smoothing parameter is first applied to the functional connectivity networks to approximate stationary pairwise connection strength signals. (**A**) The clustering coefficient (CC) and characteristic path length (PL) as computed after a smoothing parameter of 30 seconds has been applied to the adjacency matrices in a time-resolved fashion. These network measures are calculated separately for focal seizures that remain focal (*n* = 49) and focal to bilateral tonic-clonic seizures (*n* = 18). (**B**) Same network measures computed based on a temporal smoothing parameter of 1 minute. (**C**) Same network measures computed based on a temporal smoothing parameter of 5 minutes. CC of focal to bilateral tonic-clonic seizures are higher than that of focal seizures that remain focal (2-10 minutes; 2-10 minutes; seizure onset to 10 minutes after seizure onset for a smoothing parameter of 30 seconds, 1 minute, and 5 minutes, respectively). PL of focal to bilateral tonic-clonic seizures are lower than that of focal seizures that remain focal (2-10 minutes; 2-10 minutes; 1 minutes before seizure onset to 10 minutes after seizure onset for a smoothing parameter of 30 seconds, 1 minute, and 5 minutes, respectively). Statistical comparisons of network measures as a function of seizure types were computed through a bootstrapping procedure where the underlying data distribution of each network measure was resampled at the level of individual seizures to established 95% confidence intervals (CIs). Error bars indicate 95% CIs across individual seizures in each condition and solid bars show resampled *P* < 0.05. All reported results in the main text are achieved based on a smoothing parameter of 30 seconds and the comparisons illustrated here suggest that our reported findings are robust and largely unaffected by the choice of this parameter.

**
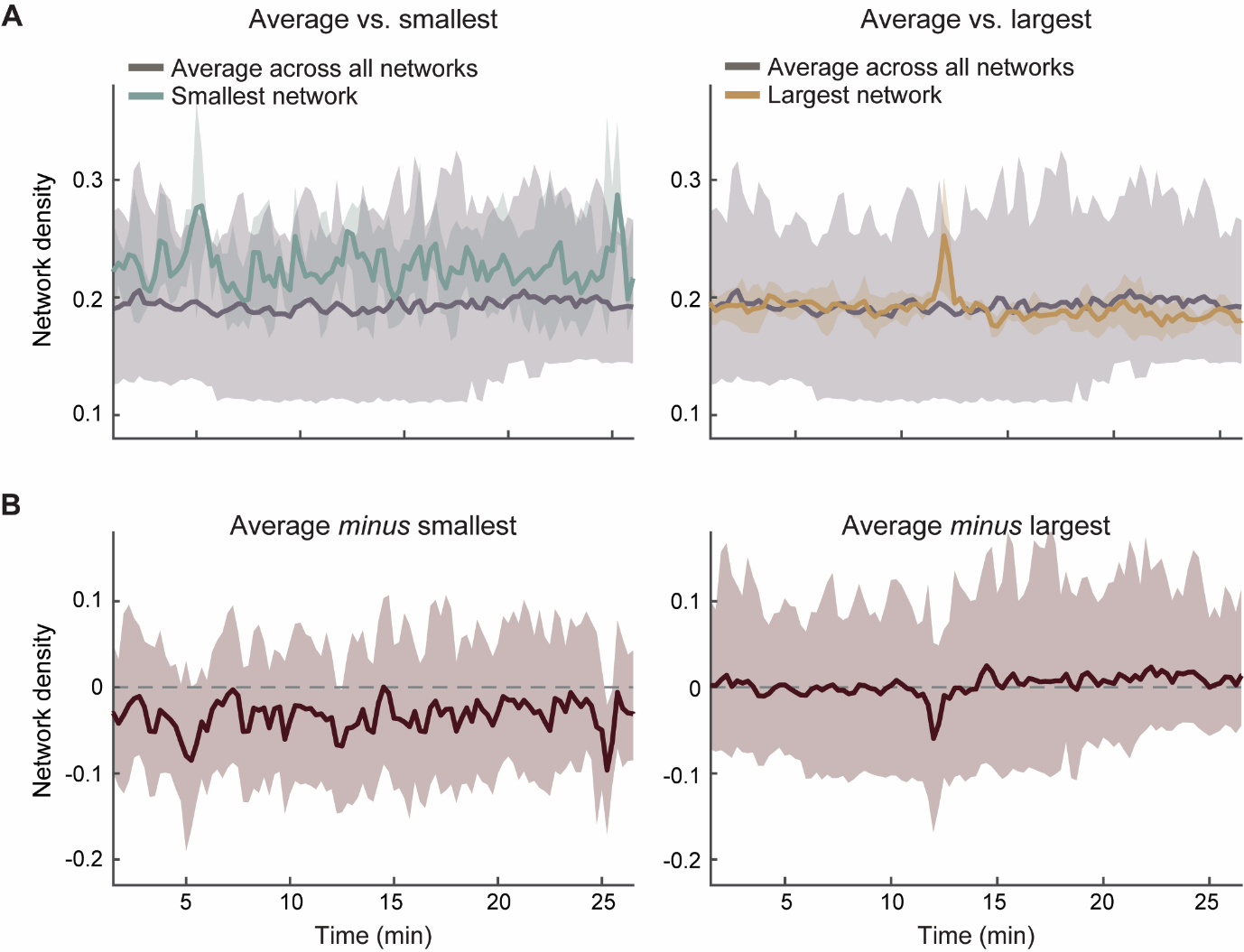
**

**Supplementary Figure 2. Density extracted from interictal networks of different sizes.** The density of smallest (i.e., 40 nodes; left panels) and largest (i.e., 118 nodes; right panels) network is illustrated with the average density computed across all functional network. The density of the smallest interictal network did not differ from the average density. Similarly, the density of the largest interictal network also did not differ from that average density computed across interictal networks of different sizes. Statistical comparisons of density as a function of network sizes were computed through a bootstrapping procedure where the underlying data distribution of each network size was resampled at the level of individual seizures to established 95% confidence intervals (CIs).

**
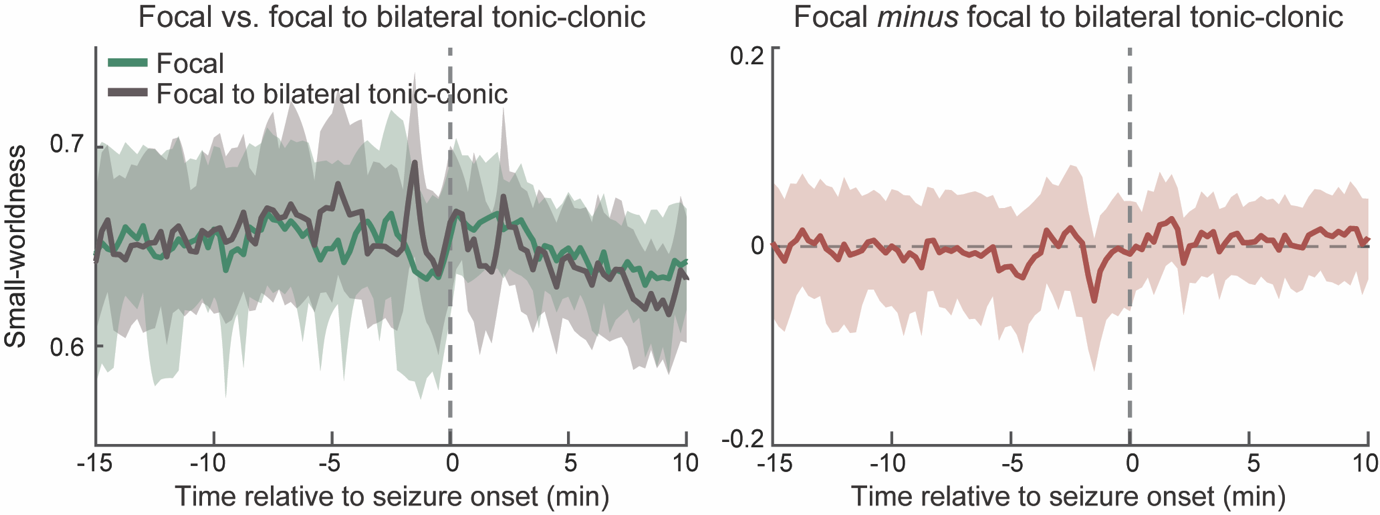
**

**Supplementary Figure 3. Small-world characteristics of focal seizure subtypes.** We computed small-worldness as the ratio of CC and PL relative to that of a random network. Random networks were generated at each time point by redistributing the edge weights of the original network such that the average degree is preserved. Focal to bilateral tonic-clonic seizures exhibit similar temporal patterns of small-world characteristics as focal seizures that remain localized. Statistical comparisons of small-world characteristics as a function of seizure types were computed through a bootstrapping procedure where the underlying data distribution was resampled at the level of individual seizures to established 95% confidence intervals (CIs).

**
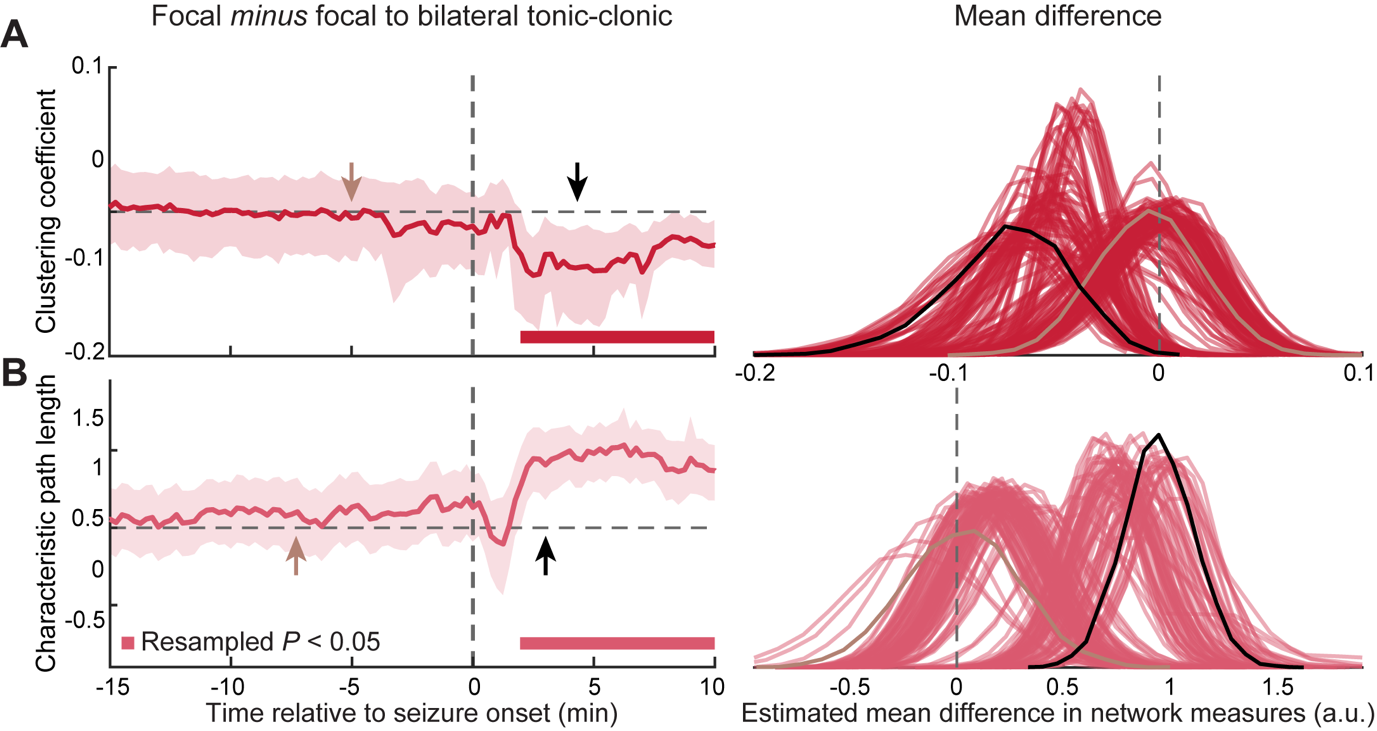
**

**Supplementary Figure 4. Effect sizes of seizure-type specific reconfigurations in clustering coefficient and characteristic path length.** To visualize the effect sizes associated with seizure-type specific differences in clustering coefficient (**A**) and characteristic path length (**B**) overtime (left panels), the bootstrapped distributions associated with the mean difference of individual timepoints are plotted (right panels). For each of the network measure, the brown and black arrows indicated a sample timepoint where the mean difference between seizure types were not significantly different and significantly different from the bootstrapped null difference of zero, respectively. The distributions of these mean differences are illustrated in the corresponding right panel. Statistical comparisons of network measures as a function of seizure types were computed through a bootstrapping procedure where the underlying data distribution of each network measure was resampled at the level of individual seizures to establish 95% confidence intervals (CIs). Error bars indicate 95% CIs across individual seizures in each condition and solid bars show resampled *P* < 0.05.

**
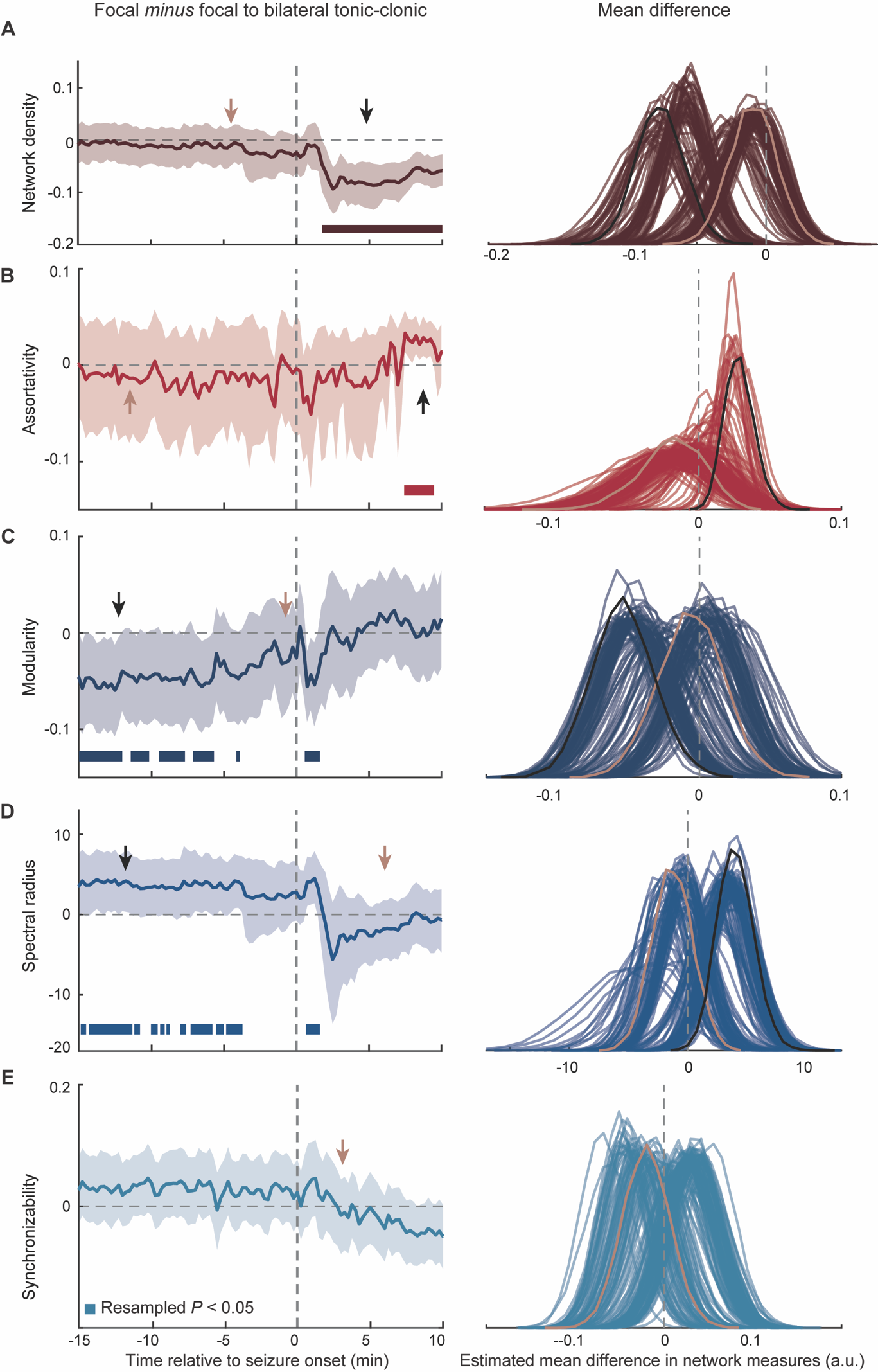
**

**Supplementary Figure 5. Effect sizes of seizure-type specific reconfigurations in various features of functional connectivity networks.** Left panels illustrate the seizure-type specific differences in network density (**A**), associativity (**B**), modularity (**C**), spectral radius (**D**), and synchronizability (**E**). The bootstrapped distributions associated with the mean difference of individual timepoints are plotted in the corresponding right panels. For each of the network measure, the brown and black arrows indicated a sample timepoint where the mean difference between seizure types were not significantly different and significantly different from the bootstrapped null difference of zero, respectively. The distributions of these mean differences are illustrated in the corresponding right panel. Statistical comparisons of network measures as a function of seizure types were computed through a bootstrapping procedure where the underlying data distribution of each network measure was resampled at the level of individual seizures to establish 95% confidence intervals (CIs). Error bars indicate 95% CIs across individual seizures in each condition and solid bars show resampled *P* < 0.05.


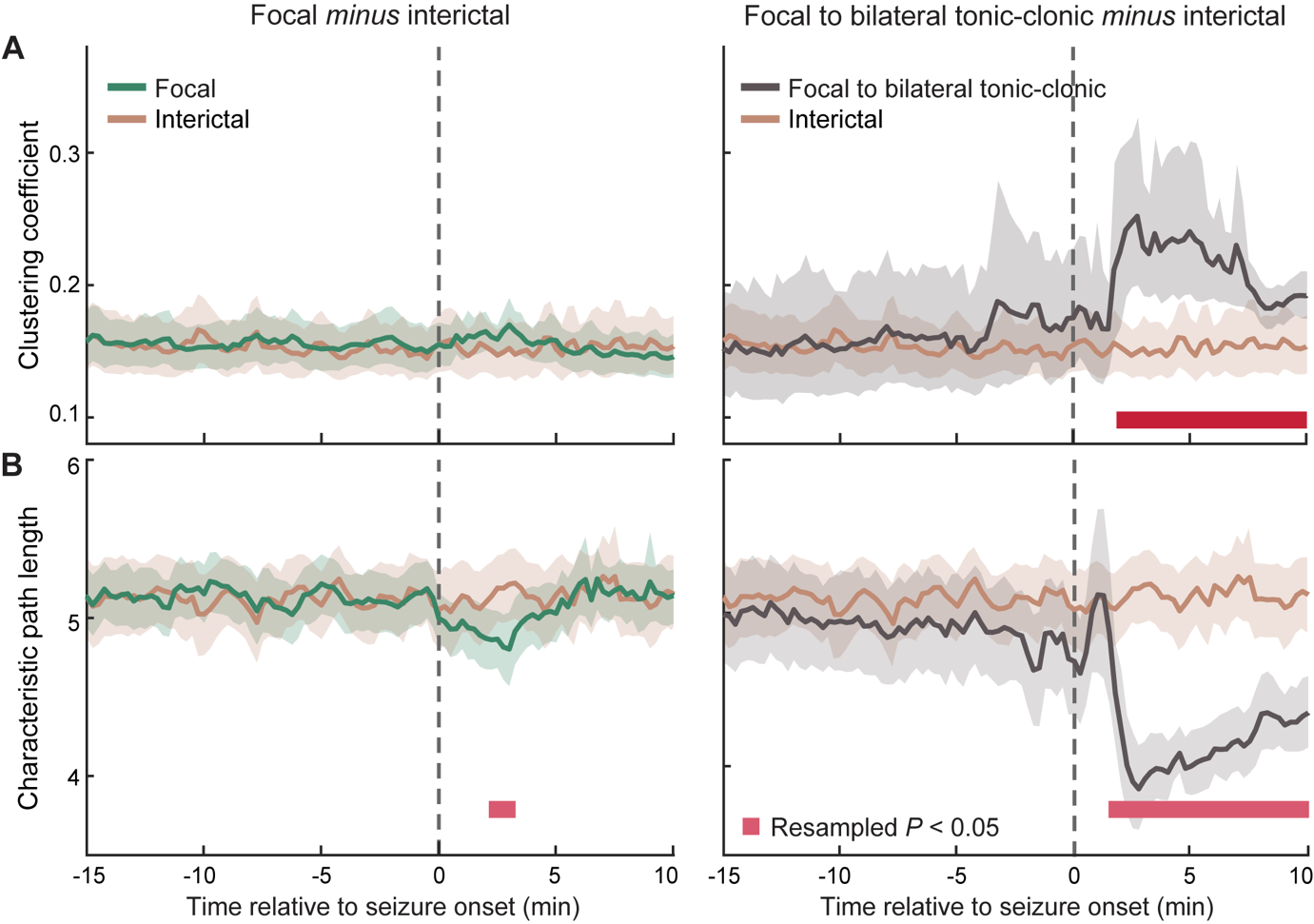


**Supplementary Figure 6. Clustering coefficient and characteristic path length of focal seizures with constrained and unconstrained propagation dynamics relative to those of interictal periods.** (**A**) The clustering coefficient (CC) of focal seizures that remain focal (*n* = 49) is not different from that of interictal networks (*n* = 49). CC of focal to bilateral tonic-clonic seizures (*n* = 18) is higher than that of interictal networks, 2-10 minutes after seizure onset. (**B**) PL of focal seizures that remain focal is lower than that of interictal networks, 2.50-3.50 minutes after seizure onset. PL of focal to bilateral tonic-clonic seizures is lower than that of interictal networks, 1.75-10 minutes after seizure onset. Statistical comparisons of network measures as a function of seizure types were computed through a bootstrapping procedure where the underlying data distribution of each network measure was resampled at the level of individual seizures (or interictal epochs) to established 95% confidence intervals (CIs). Error bars indicated 95% CIs across individual seizures (or interictal epochs) in each condition and solid bars show resampled *P* < 0.05.


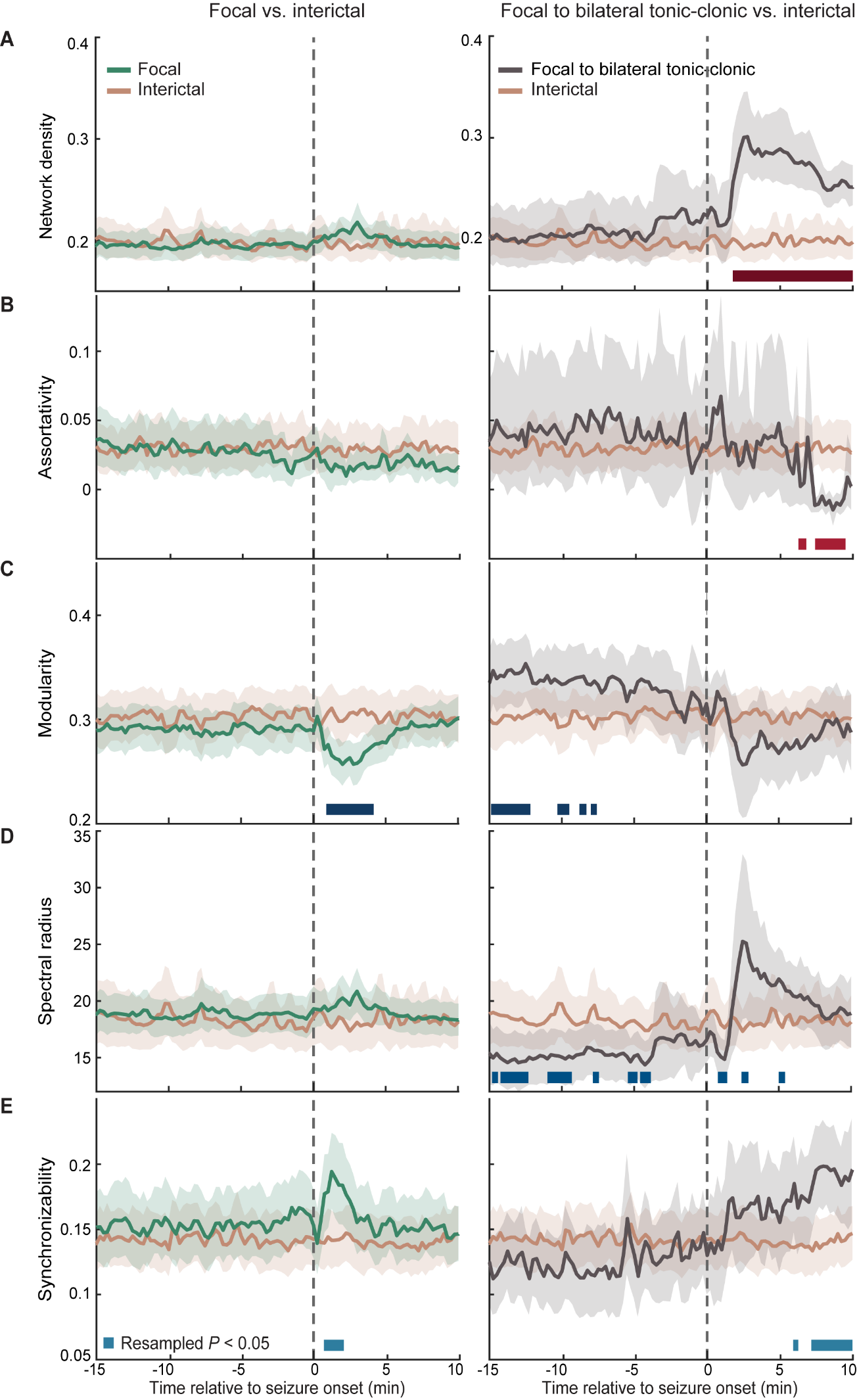


**Supplementary Figure 7. Network features of focal seizures with constrained and unconstrained dynamics in comparison to those of interictal activity. (A)** The density of focal seizures that remain focal (*n* = 49) is not different from that of interictal networks (*n* = 49). The density of focal to bilateral tonic-clonic seizures (*n* = 18) is higher than that of interictal networks, 1.75-10 minutes after seizure onset. **(B)** The assortativity of focal seizures that remain focal is not different from that of interictal networks. The assortativity of focal to bilateral tonic-clonic seizures is higher than that of interictal networks, 6.50-7 and 7.50-9.75 minutes after seizure onset. **(C)** The modularity of focal seizures that remain focal is lower than that of inter-ictal networks, 1-4.25 minutes after seizure onset. The modularity of focal to bilateral tonic-clonic seizures is higher than that of interictal networks, 14.75-12, 10.25-10, 9.75-9.5, and 8.5-8.25 minutes before seizure onset **(D)** The spectral radius of focal seizures that remain focal is not different from that of interictal networks. The spectral radius of focal to bilateral tonic-clonic seizures is lower than that of interictal networks, 14.75-14.50, 14-12.25, 11-9.5, 7.75-7.50, 5.25-4.75, and 4.50-3.75 minutes before seizure onset; and 1-1.50, 2.50-3, and 5-5.50 minutes after seizure onset. **(E)** The synchronizability of focal seizures that remain focal is higher than that of interictal networks, 0.75-2 minutes after seizure onset. The synchronizability of focal to bilateral tonic-clonic seizures is higher than that of interictal networks, 6-6.25 and 7.25-10 minutes after seizure onset. Statistical comparisons of network measures as a function of seizure types were computed through a bootstrapping procedure where the underlying data distribution of each network measure was resampled at the level of individual seizures (or interictal epochs) to established 95% confidence intervals (CIs). Error bars indicated 95% CIs across individual seizures (or interictal epochs) in each condition and solid bars show resampled *P* < 0.05.
