# Supplementary material for: Brain network dynamics codify heterogeneity in seizure evolution
Source: Brain Commun. 2022 Sep 16;4(5):fcac234. doi: 10.1093/braincomms/fcac234 (PMC9527667; doi:10.1093/braincomms/fcac234)
Supplement: fcac234_Supplementary_Data [file Original_submission_decision_letter.pdf › Original_submission_decision_letter.pdf]

Dear Dr. Bansal,

Thank you for your recent submission BRAINCOM-2021-255 entitled "Brain network dynamics codify heterogeneity in seizure evolution" to Brain Communications.

Your manuscript has been reviewed, and while the reviewers and editorial team appreciate many aspects of the paper, they raise some concerns that you will need to address in order for the paper to be considered for publication.

If you can address these concerns, I invite you to submit a revised manuscript and a response to each of the reviewers' comments. The reviewer(s)' comments are included at the bottom of this message.

In addition to the reviewers' comments, please also address our Scientific Editor's comments added below to comply with our requirements for presentation of scientific data at Brain Communications.

Please format your revised manuscript according to our Instructions to Authors https://academic.oup.com/braincomms/pages/General_Instructions – a summary of key points is attached to help with this.

To revise your manuscript, click the link below or log into https://mc.manuscriptcentral.com/braincom and enter your Author Centre, where you will find your manuscript title listed under "Manuscripts with Decisions." Under "Actions," click on "Create a Revision." Your manuscript number will be appended to denote a revision.

*** PLEASE NOTE: This is a two-step process. After clicking on the link, you will be directed to a webpage to confirm. ***

https://mc.manuscriptcentral.com/braincom?URL_MASK=afbc2950af524e829537c6b2d5e21db5

Please upload a revised manuscript with changes tracked with track changes or in coloured text.

When submitting your revised manuscript, you will be asked to respond to the reviewer(s) comments. Please be as specific as possible in your response to all of the comments.

The editorial team at Brain Communications aims to increase transparency in scientific publishing and to demonstrate the value that peer review brings to the scientific process. Please therefore consider whether you are willing to have reviewer(s)' comments on your paper and your response published as supplementary data with the paper if it is accepted. This is entirely optional and will only be done if both the authors and reviewers agree. While submitting your revision there will be an opportunity to review or update the answer you gave previously, if necessary.

IMPORTANT: Your original files are available to you when you upload your revised manuscript. Please delete any redundant files before completing the submission.

Because we are trying to facilitate timely publication of manuscripts submitted to Brain Communications, we would appreciate your revised manuscript as soon as possible. If it is not possible for you to resubmit within 3 months, the option to submit a revision will expire, and we would then consider your revised paper as a new submission. However, if you need a little more time, please get in touch and we can extend this deadline for you.

Once again, thank you for submitting your manuscript to Brain Communications and I look forward to receiving your revision.

Best wishes

Prof. Richard Chin
Associate Editor, Brain Communications

Professor Tara Spires-Jones
Founding Editor, Brain Communications

Reviewer(s)' Comments to Author:

Reviewer: 1

Comments to the Author
Brain network dynamics codify heterogeneity in seizure evolution

This study aims to comprehensively describe changes in graph properties before, during, and after focal seizures. The authors mainly desribe how these properties differentiate focal seizures that remain focal from focal seizures that undergo bi-lateral, tonic-clonic transition. They find that global network properties may predict the eventual type of seizure, before they happen, and more local network properties may encode seizure dynamics after they have begun and well-after they have finished. Though many prior studies have examined the network properties that are studied here, this paper excels at evaluating the putative roles of a whole library of local and global topological features. I found this very interesting. However, the paper is ambitious and I wonder whether the large number of graph features ensures that a positive result is found. Would a feature selection approach have helped select features more parsimoniously? At some level, many of the network properties studied here likely have inherent correlations amongst one another. Finally, I would have appreciated a more nuanced look at the actual dynamic differences between seizure types – leveraging the high temporal resolution of the electrophysiology signal. Despite these pitfalls, I thought the study was rigorous and comprehensive. If these features are validated, in larger datasets and at the patient level (not treating seizures independently) they could have high clinical utility. However, I would like to see some methodological concerns addressed before I can recommend the paper for publication.

1. I felt the introduction could benefit from more clinical insight into the importance of taking a network perspective. Why is this helpful above conventional epileptiform biomarker mapping approaches? What utility is there in distinguishing these seizure types as they are happening?

2. The issue of computing a Pearson correlation / cross-correlation measure of functional connectivity is well known, and particularly for electrophysiology signals that follow a 1/f power distribution. A couple important pitfalls exist: (i) these measures are biased to low-frequency components in the signal, and (ii) the underlying signals are time-series whose observations are not statistically independent (i.e. have strong autocorrelation such that t is correlated to t+1). Spectral approaches (frequency-band coherence, phase-locking value, phase-lag index, etc.) mitigate these issues because they specifically analyze different regimes of the signal's spectrum. I don't necessarily think the authors need to go this route, if they wish to use correlation-based approach, at minimum I recommend they use a pre-whitening step to flatten the underlying power-spectrum and reduce autocorrelation.

3. I felt that the authors could practice more caution in over-interpreting the function of the FC measure. I have the specific following concerns:
a. Use of terms such as "long-range inhibition" and "direct inhibitory connections". Correlations do not imply direct connections and negative correlations cannot imply inhibition – they are simply a result of two signals being 180-degrees out-of-phase in their fluctuation (negative/positive voltage values do not imply excitation/inhibition).

b. Graph measures that assess synchronization (including but not limited to synchronizability and "spread of synchrony") are difficult to interpret in the context of the connectivity metric – which is itself a measure of correlation/synchrony/similarity between signals. If a graph has high connectivity (high edge weight density) between nodes, doesn't that imply it is synchronized? Moreover, if the graph increases in its edge density from one timepoint to the next, does that not imply that synchrony has spread? Some rationale would be helpful.

4. In general, have the authors controlled for differences in the overall edge weight density (mean correlation across all edges) when comparing metrics between time points and between seizure types? It seems likely that a more severe generalized seizure would have overall higher correlation/synchrony due to more synchronous spiking. Are the metrics that are tracked in this study simply picking up on gross-changes in edge weights?

5. I found it odd that the heavily time-resolved networks were both smoothed across time and then also averaged – producing one network for the ictal period. I worry that by doing so, the authors miss out on the truly informative state changes / transition points that occur during the seizure (Fig 3A-B).

6. How were differences in seizure durations handled? Seizures of one type may be generally of different lengths than seizures of another type. The analysis time-locks to the onset, but then it's unclear when the seizure is finished. Could a complimentary analysis time-lock to the termination/offset?

7. Similarly, how can the authors be sure they are performing an apples-to-apples comparison of the

distinct seizure phases. If one seizure spreads after 10 seconds, another seizure spreads at 30 seconds, and a focal seizure exhibited spread to adjacent electrodes after some other delay. Does the averaging approach dilute these changes?

Reviewer: 2 - Sinha, Nishant

Comments to the Author
This study compares the time-evolving topological differences between focal to bilateral tonic colonic seizures (FBTCS) and focal only seizures. The authors use 25 mins window—15 mins before seizure onset and 10 mins after seizure onset—to compare graph theory metrics between the two seizure types. They compute time-varying functional connectivity in each patient and derive different graph theory metrics for epochs within 25 mins time window. The authors perform a group analysis to determine if the graph theory metrics are different between FBTCS and focal only seizures. They report that the average clustering coefficient, average path length, average degree, and assortativity coefficients differed after seizure onset whilst modularity, synchonizability, and spectral radius differed before seizure onset.
However, I have concerns on the methodological approach and overinterpretation of results in the manuscript beyond what is shown by the data. These must be addressed before I can make a recommendation for publication. My comments are chronologically listed below:

A major limitation of the study is that the authors are comparing graphs of different sizes and densities. Each patient will have a different number of electrodes implanted; therefore, network size and density in each patient will be different. Please include electrode types and the number of impanated electrodes as an additional column in Table 1.
Graph metrics scale with network size and density, and it is well established that without incorporating appropriate controls, comparing networks of different sizes and densities induce biases. Please refer to van Wijk BCM, Stam CJ, Daffertshofer A (2010) Comparing Brain Networks of Different Size and Connectivity Density Using Graph Theory. PLoS ONE 5(10): e13701.
https://doi.org/10.1371/journal.pone.0013701 The authors are suggested to correct for this statistical bias in their analysis.

Introduction:
“differential neural dynamics as well as the distinct cognitive and behavioral changes associated” There are no data in this manuscript to support any association with cognition or behavior. Focal only seizures also associate with cognition and behavior. I suggest removing all claims about association with cognition/behavior from the manuscript.

Data preprocessing:
“For comparison with relatively ‘seizure-free’ (interictal) activity, we extracted an equal number of interictal activity windows with the same duration.” Is this correct? Equal number means 67 interictal activity preceding 67 seizures. Please clarify was the same interictal epoch also used with other seizures.

Functional connectivity networks:
Negative Pearson correlation coefficient does not necessary imply long-range inhibitions. Negative values could also appear due to phase lags. It is a simply a choice to set negative values to zero, but they can’t be justified as having no contributions to epileptogenic effect. Authors are suggested to reword this sentence.

Assessing small-world architecture:
Authors are not computing the small world metric anywhere in their analysis. As such, a qualitative comparison by observing clustering and path length is not suffice. I suggest to either remove all qualitative references to small work architecture or quantify it.

Modularity:
Please add more details on modularity. What algorithm was used? How was the optimal modularity decided?

Spectral radius and Synchronizability:
Can the authors plot a scatter plot between Spectral radius and synchronizability metrics? These measures seem to be highly correlated.

Statistical analysis:
What checks were incorporated for dealing with multiple comparisons? Authors are suggested to report effect size and its 95% CI in addition to p-values.

Results: More prominent small-world connectivity links to bilateral spread of seizure activity

“increased small-world connectivity has been proposed as a potential driver for the propagation of pathological synchronous activity”
I don’t think that the interpretation about small-world connectivity as a potential driver for the propagation is correct. Can the authors clarify what do they mean?

Fig. 3 A and B: Please show the data points on the plot.

Re: “Our results reveal that both focal seizures that remain focal and focal to bilateral tonic-clonic seizures displayed higher small-world connectivity during ictal periods when compared to seizure-free (interictal) activity…” and “Notably, the observed differences regarding the manner in which the small-world architecture increased in the networks of focal seizures with constrained and unconstrained dynamics were our first evidence in support of the notion that there may exist network-level signatures that contained information about the distinct propagation mechanisms of focal seizures.”
See my comment above, there are no computations for small world index. Please state the results directly in terms of clustering coefficient and path length.

“…..to an increase in the efficiency of network communication, as illustrated by the…” why not use the network communicability metric to study this?

“These results suggested that close to seizure termination, the networks of focal seizures with unconstrained dynamics underwent reduced robustness, rendering them more susceptible to network disruptions. These findings could potentially account for the more extensive behavioral abnormalities and cognitive deficits often observed after patients experience episodes of focal to bilateral tonic-clonic seizures.” This is an overinterpretation of the results. How do you compute robustness? Behavioral abnormalities and cognitive deficits are also common in focal seizures. This statement should be removed.

Results: Alterations in global network features preceding the onset predict propagation dynamics of focal seizures
“predictive manner” there are no predictions anywhere in this manuscript. The authors show associations and they should be reported as such.

Figure 4D and E are exact same graph. Is spectral radius redundant?

Figure 6 is totally unclear to me. Can the authors plot the graphs in the same axis to facilitate visual comparison? The different visualization might be more suitable.

Discussion:
“predicted the extent of diffusion dynamics of an impending seizure as well as isolated architectural changes” The manuscript doesn’t make any predictions; the results illustrates associations/correlates. There is also no way to determine the extent of seizure using average network measures; FBTCS have different extent. Please reword this statement.

“Furthermore, our findings provide a rationale for utilizing network features to guide clinical diagnosis of seizure subtypes and effective intervention strategies to constrain propagation of seizure activity.” Overstatement about seizure subtypes. The authors are comparing only two seizure types FBTCS and focal only. The argument about intervention strategies is purely speculative and is not supported by data in this manuscript.

Network alterations track temporal evolution of focal seizures:
“Specifically, our findings revealed a relationship between the extent of small-world architecture and the diffusivity of seizure activity after the onset.” There is no data to quantify small-world architecture and diffusivity of seizure activity: it is only FBTCS or focal only distinction. These statements should be tempered/corrected.

“We argue that our approach provides an objective framework not only for better understanding the neural dynamics underlying evolution of seizures but also for determining whether and when a clinical intervention should be implemented to manage and control a spread of an impending seizure.” The statement about clinical intervention is purely speculative. Please discuss how the data in this manuscript support a tailored intervention guiding clinical intervention.

The discussion section on “Bilateral propagation of focal seizures reflects imbalance in global integration and segregation in the connectivity brain network” is very unclear to me. The authors should discuss their findings in context of existing mechanisms of FBTCS like thalamic—basal ganglia circuits.

The discriminatory network level metrics cannot be considered as biomarkers unless they are cross validated.

Conclusions: Authors are suggested to temper down their claims specific to the data shown in this manuscript. The speculative aspects should be stated clearly as plausible directions.

Abstract
The three points in abstract needs to be tempered:
"…that are predictive of the extent…"— associate with FBTCS or focal only seizures.
"…could aid successful intervention strategies…" — speculative. The results do not support this statement.
"…focal seizure subtypes…" again please be specific. You are only comparing FBTCS with focal only seizure not all focal seizure subtypes.

Comments from the Scientific Editor:

-You have supplied a thumbnail when originally submitting to Brain. Brain Communications uses graphical abstracts. Please confirm whether your thumbnail is suitable or whether you would like to supply something different?
A graphical abstract is a visual representation of the central finding or methodology of the paper. It will be displayed in the article and in the table of contents, and used to promote your article on Twitter. Authors should strive to make the graphical abstract informative, interesting, visually appealing, and straightforward (no caption needed, no abbreviations except gene/protein names). Please upload it as a separate high resolution image file (tiff preferred). The technical requirements for graphical abstracts are:
-size of the submitted image: 1200 pixels square at 300dpi
-font of the text: Arial 12-16 points (smaller fonts will not be legible online)

-Please add an Abbreviated Summary (up to 50 words) of your paper that captures the main purpose and conclusions of the work. The summary should state the main results written in the third person ('[1st author surname] et al. report that …') and conclusion. This summary will be used in the contents list online and should not contain any abbreviations other than those on the accepted abbreviations list and accepted gene/protein names, making it accessible to the non-specialist. Please upload it as a Word document. Allowed abbreviations: AIDS; ANOVA; AMPA; ATP; A,T,C,G; CNS; CSF; CT; DNA; ECG; EEG; EMG; GABA; HIV; MRI; NMDA; PET; PCR; RNA.

- To comply with our ethical standards, the appropriate checklist must be included (see Reporting Guideline section within 'Instructions to authors').

-Fig3, Fig4 and Supp Fig1-3: Please provide details of the experimental unit (n= cells, wells, plate downs, etc)) and of the statistical analysis performed in the figure legends.
