# Supplementary material for: Brain network dynamics codify heterogeneity in seizure evolution
Source: Brain Commun. 2022 Sep 16;4(5):fcac234. doi: 10.1093/braincomms/fcac234 (PMC9527667; doi:10.1093/braincomms/fcac234)
Supplement: fcac234_Supplementary_Data [file Response_to_reviewers_comments_original.pdf › Response_to_reviewers_comments_original.pdf]

Dear Editor,

Thank you for giving us the opportunity to submit a revised draft of our manuscript entitled "Brain network dynamics codify heterogeneity in seizure evolution" to *Brain Communications*. We are grateful for the time and effort that you and the reviewers have dedicated to providing valuable and insightful feedback on our manuscript. We have included a point-by-point response to each reviewer's comment in the response letter below. The comments are in black, and our replies are in blue. All the additions within the revised manuscripts are also in blue.

Sincerely,

Dr. Kanika Bansal, corresponding author

Comments from Editor:

1. You have supplied a thumbnail when originally submitting to Brain. Brain Communications uses graphical abstracts. Please confirm whether your thumbnail is suitable or whether you would like to supply something different? A graphical abstract is a visual representation of the central finding or methodology of the paper. It will be displayed in the article and in the table of contents, and used to promote your article on Twitter. Authors should strive to make the graphical abstract informative, interesting, visually appealing, and straightforward (no caption needed, no abbreviations except gene/protein names). Please upload it as a separate high resolution image file (tiff preferred). The technical requirements for graphical abstracts are:
- size of the submitted image: 1200 pixels square at 300dpi
- font of the text: Arial 12-16 points (smaller fonts will not be legible online)

We would like to use an alternative thumbnail as a graphical abstract and have uploaded the file along with the revised manuscript.

2. Please add an Abbreviated Summary (up to 50 words) of your paper that captures the main purpose and conclusions of the work. The summary should state the main results written in the third person ('[1st author surname] et al. report that …') and conclusion. This summary will be used in the contents list online and should not contain any abbreviations other than those on the accepted abbreviations list and accepted gene/protein names, making it accessible to the non-specialist. Please upload it as a Word document. Allowed abbreviations: AIDS; ANOVA; AMPA; ATP; A,T,C,G; CNS; CSF; CT; DNA; ECG; EEG; EMG; GABA; HIV; MRI; NMDA; PET; PCR; RNA.

The abbreviated summary has been attached as a Word document.

3. To comply with our ethical standards, the appropriate checklist must be included (see Reporting Guideline section within 'Instructions to authors').

We have included the checklist as part of our resubmission package.

4. Fig3, Fig4 and Supp Fig1-3: Please provide details of the experimental unit (n= cells, wells, plate downs, etc)) and of the statistical analysis performed in the figure legends.

The details of the experimental unit (i.e., the number of each seizure type and interictal periods) has been indicated in the figure legends of Fig 3, Fig 4, and Supp Fig 1-3 in the previous

version of the manuscript in the following format: (Fig 4) "… focal seizures that remain localized (n = 49) and from networks of focal to bilateral tonic-clonic seizures (n = 18)". In terms of the statistical analysis performed, we have now added additional text to each of the figure legends, by writing: "Statistical comparisons of network measures as a function of seizure types were computed through a bootstrapping procedure where the underlying data distribution of each network measure was resampled at the level of individual seizures to established 95% confidence intervals (CIs). Error bars indicate 95% CIs across individual seizures in each condition and solid bars show resampled $P < 0.05$." for Fig 3, Fig 4, Fig S1, S4, and S5.

We have also added similar text to the legends of Fig S6 and Fig S7, by writing: "Statistical comparisons of network measures as a function of seizure types were computed through a bootstrapping procedure where the underlying data distribution of each network measure was resampled at the level of individual seizures (or interictal epochs) to established 95% confidence intervals (CIs). Error bars indicated 95% CIs across individual seizures (or interictal epochs) in each condition and solid bars show resampled $P < 0.05$."

Comments from Reviewer 1:

This study aims to comprehensively describe changes in graph properties before, during, and after focal seizures. The authors mainly desribe how these properties differentiate focal seizures that remain focal from focal seizures that undergo bi-lateral, tonic-clonic transition. They find that global network properties may predict the eventual type of seizure, before they happen, and more local network properties may encode seizure dynamics after they have begun and well-after they have finished. Though many prior studies have examined the network properties that are studied here, this paper excels at evaluating the putative roles of a whole library of local and global topological features. I found this very interesting. However, the paper is ambitious and I wonder whether the large number of graph features ensures that a positive result is found. Would a feature selection approach have helped select features more parsimoniously? At some level, many of the network properties studied here likely have inherent correlations amongst one another. Finally, I would have appreciated a more nuanced look at the actual dynamic differences between seizure types – leveraging the high temporal resolution of the electrophysiology signal. Despite these pitfalls, I thought the study was rigorous and comprehensive. If these features are validated, in larger datasets and at the patient level (not treating seizures independently) they could have high clinical utility. However, I would like to see some methodological concerns addressed before I can recommend the paper for publication.

We thank the reviewer for their positive feedback and thoughtful comments regarding our analytical techniques and reported findings. While we agree that a feature selection approach would be helpful in identifying network features that best differentiate between seizure types, our study was motivated by the fact that assessing a blend of different local and global features associated with each seizure type could provide a more comprehensive insight into *how* diverse seizure activity emerges from the brain. With that in mind, here we selected network features that reflect unique and distinct aspects of the underlying brain dynamics. We demonstrate that evaluating a host of features together provides a more complete picture of the distinct nature of each seizure type. We agree that our reported findings need to be validated through studies with larger datasets at the patient level to gain better clinical utility. To this end, we believe that the

analytical frameworks and findings presented in this manuscript will serve as a basis to motivate such studies in future. Again, we thank the reviewer for their comments which have helped us improve the clarity of our methodological approach and in the following, provide a point-by-point response to their concerns.

1. I felt the introduction could benefit from more clinical insight into the importance of taking a network perspective. Why is this helpful above conventional epileptiform biomarker mapping approaches? What utility is there in distinguishing these seizure types as they are happening?

Thank you for this suggestion. We have now expanded the introduction to highlight the utility and benefit of taking a network perspective. Here is the relevant text reproduced from the revised manuscript:

"…Objective biomarkers that can identify whether or not an impending seizure will spread bilaterally could allow clinicians to implement an appropriate intervention strategy, thereby minimizing the severity of adverse cognitive impacts.

For the past several years, epilepsy has more frequently been viewed as a manifestation of network reorganizations and not just hypersynchrony, and a graph theoretical framework in which brain dynamics is visualized as temporally evolving graphs or networks composed of nodes and edges that represent brain regions and their pairwise associations, respectively,[14–16] has been useful in quantifying and understanding these network reorganizations.[17–19]"

2. The issue of computing a Pearson correlation / cross-correlation measure of functional connectivity is well known, and particularly for electrophysiology signals that follow a 1/f power distribution. A couple important pitfalls exist: (i) these measures are biased to low-frequency components in the signal, and (ii) the underlying signals are time-series whose observations are not statistically independent (i.e. have strong autocorrelation such that t is correlated to t+1). Spectral approaches (frequency-band coherence, phase-locking value, phase-lag index, etc.) mitigate these issues because they specifically analyze different regimes of the signal's spectrum. I don't necessarily think the authors need to go this route, if they wish to use correlation-based approach, at minimum I recommend they use a pre-whitening step to flatten the underlying power-spectrum and reduce autocorrelation.

We agree that computing a correlation/ cross-correlation of functional connectivity has important pitfalls that emerge when conclusions are drawn from a statistical comparison of cross-correlations associated with related time series using the conventional 95% significance limits (Dean and Dunsmuir, *Behavior Research Methods*, 2016). We understand that to draw such conclusions, one would need to consider the degree of autocorrelation and adjust the significance limits accordingly. Our analyses were agnostic to this fact, and do not draw any direct conclusions based on the computed correlation measures. Instead, we merely calculated windowed-correlations across each pair of recording electrodes to estimate how these electrodes (i.e., the underlying cortical regions) are functionally related to one another and how this relationship evolves during seizure onset.

With that said, we have incorporated a prewhitening step during preprocessing similar to the method implemented by past studies of neural time series and recomputed all of the network measures. The figures and statistical values reported in the revised manuscript are constructed from the prewhitened signals. Specifically, we have added additional text describing the prewhitening step in the data analyses section by writing:

"Prewhitening was then performed using a first-order autoregressive model to account for slow dynamics and correct for autocorrelation in the time series signals.[25–28]"

3. I felt that the authors could practice more caution in over-interpreting the function of the FC measure. I have the specific following concerns:

a. Use of terms such as "long-range inhibition" and "direct inhibitory connections". Correlations do not imply direct connections and negative correlations cannot imply inhibition – they are simply a result of two signals being 180-degrees out-of-phase in their fluctuation (negative/positive voltage values do not imply excitation/inhibition).

Thank you for your feedback. We have gone through the manuscript and carefully addressed this concern by eliminating wordings that imply causal interpretations of the functional connectivity measures.

b. Graph measures that assess synchronization (including but not limited to synchronizability and "spread of synchrony") are difficult to interpret in the context of the connectivity metric – which is itself a measure of correlation/synchrony/similarity between signals. If a graph has high connectivity (high edge weight density) between nodes, doesn't that imply it is synchronized? Moreover, if the graph increases in its edge density from one timepoint to the next, does that not imply that synchrony has spread? Some rationale would be helpful.

This is a great point — we appreciate the reviewer for bringing it up. For complex networks, global network features, such as synchronizability spectral radius, and modularity, quantify different aspects of the complex connectivity pattern which emerges through the relative distribution of edge weights. Generally, an increase in edge weight density would imply spread of 'synchronization'; however, the metric synchronizability (and other global features such as spectral radius and modularity) can change due to network reconfigurations even when the overall values of edge weights remain largely similar (please refer to Chen and Duan, Network synchronizability analysis: A graph-theoretic approach, *Chaos* **18**, 037102 (2008)). To quickly demonstrate this here, in the following figure we construct two different networks (by randomly redistributing the edge weights between nodes) from the exact same edge weight distribution (i.e., same density) and observe different values of synchronizability, spectral radius, and modularity. Such metrics are sensitive not only to the overall values of edge weights, but also to the relative distribution of these edge weights within the network.

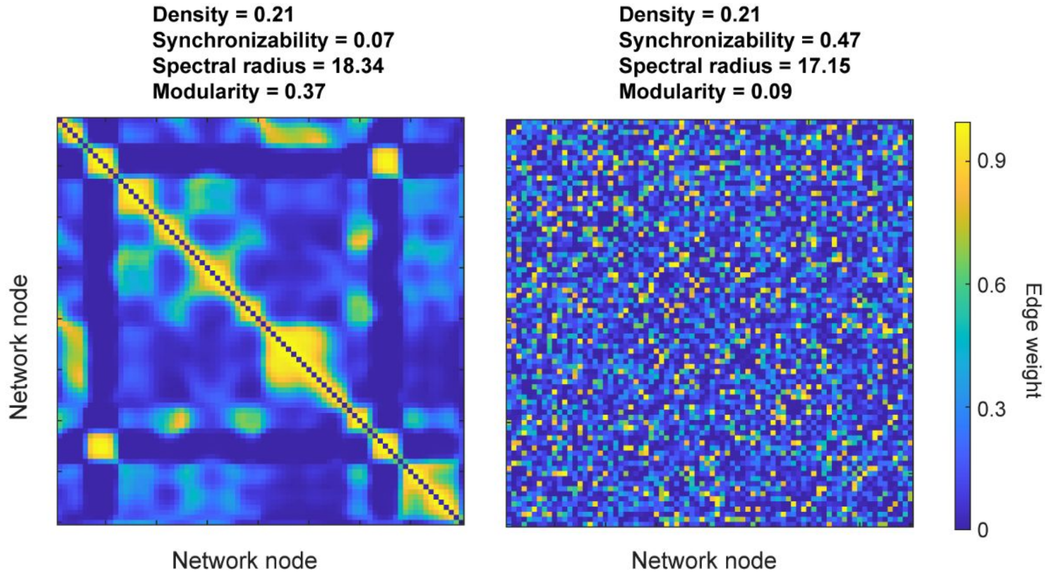

With that said, we understand that the nomenclature of these complex network features (particularly synchronizability) can be confusing. Thus, we have added an explanation in the **Material and methods** section highlighting that the global network features we focused on in the present study are sensitive not only to the overall values of edge weights, but also to the relative distribution of these edge weights within the network, which can get reconfigured as a result of various neurophysiological processes. Examining and quantifying various aspects of these network reconfigurations that differentiate (or do not differentiate) the two types of focal seizures with different propagation dynamics could elucidate the underlying mechanisms that govern bilateral spread of focal seizures. For ease of review, we reproduce here the text we added to the manuscript:

"…We focused our analyses on complex network measures that are sensitive not only to the overall extent of connectivity within a network (i.e., density; see below), but also to the relative distribution of connectivity weights within the network which reconfigure to enable various neurophysiological processes. Investigating and quantifying these network reconfigurations as a function of seizure types allows for a better understanding of the underlying processes that govern the bilateral spread of a focal seizure."

4. In general, have the authors controlled for differences in the overall edge weight density (mean correlation across all edges) when comparing metrics between time points and between seizure types? It seems likely that a more severe generalized seizure would have overall higher correlation/synchrony due to more synchronous spiking. Are the metrics that are tracked in this study simply picking up on gross-changes in edge weights?

We did control for the overall edge weight density and now have added a new Supplementary Fig. 2 and have modified the Fig. 4 to explicitly discuss the effect of density.

First, the Supplementary Fig. 2 (shown below) demonstrates that for interictal periods, functional connectivity networks extracted from different patients with different number of recording electrodes (i.e., different network sizes) did not show any significant difference in the density. This comparison ensured that our reported findings are truly driven by the underlying

changes in the functional connectivity as a function of seizure types and are free of spurious results due to different network sizes and densities across patients.

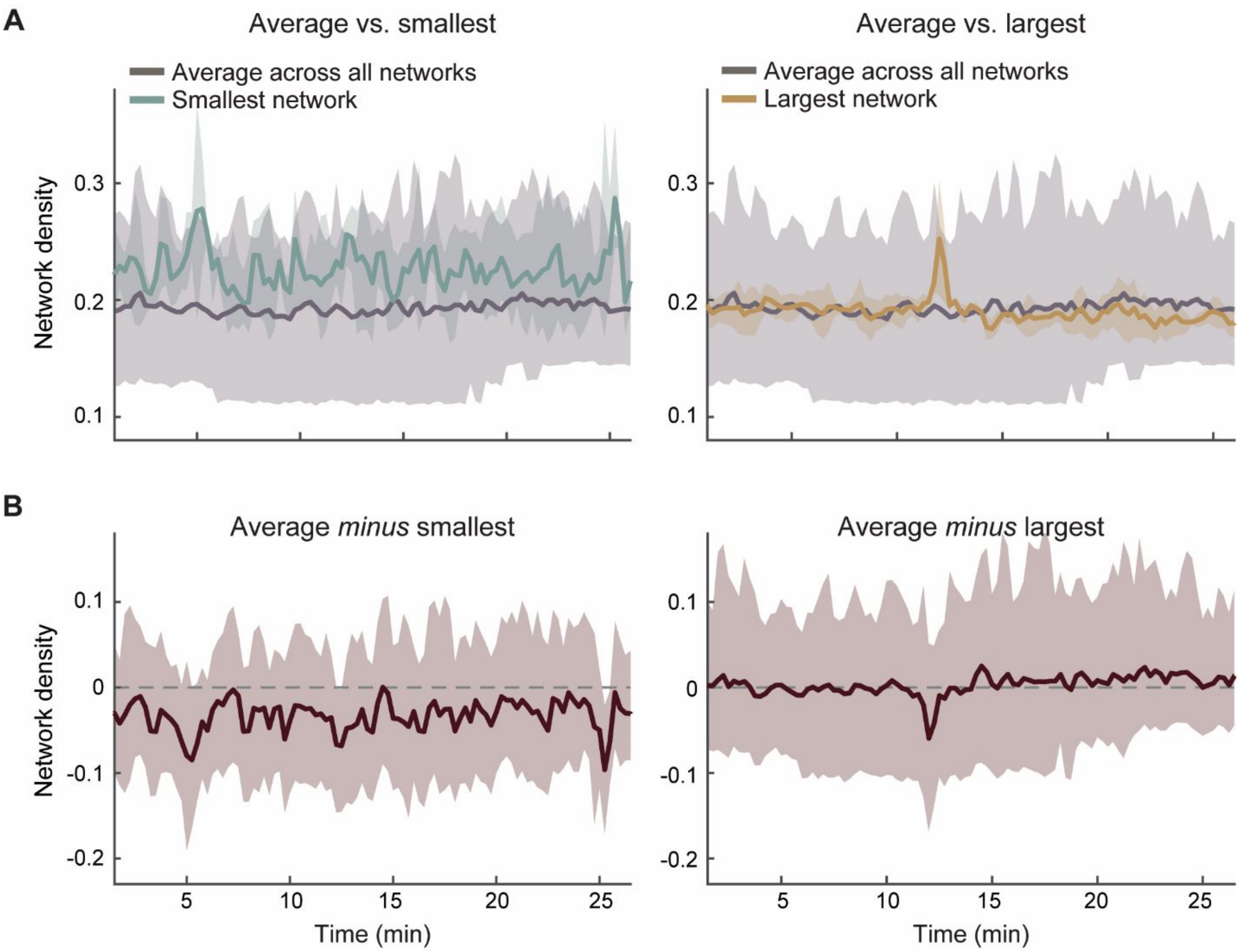

Fig. S2. Density extracted from interictal networks of different sizes. The density of smallest (i.e., 40 nodes; left panels) and largest (i.e., 118 nodes; right panels) is illustrated with the average density computed across all functional network. The density of the smallest interictal network did not differ from the average density. Similarly, the density of the largest interictal network also did not differ from that average density computed across interictal networks of different sizes. Statistical comparisons of density as a function of network sizes were computed through a bootstrapping procedure where the underlying data distribution of each network size was resampled at the level of individual seizures to established 95% confidence intervals (CIs).

Additionally, we added network density as one of the network features compared between the two seizure types and modified the Fig. 4 to include the density results (the added panel is reproduced below). As the reviewer pointed out, a more severe generalized seizure would have an overall higher connectivity due to more synchronous spiking; accordingly, for seizures that spread bilaterally, we do observe higher density (Fig. 4A) than the seizures that remain focal shortly after the seizure onset. However, during the pre-onset period, the density does not differentiate the two types of seizures. Thus, one would require more sophisticated

quantification, beyond the simplistic feature of density, to assess the network reconfigurations that contain the information regarding the possibility that an impending would bilaterally propagate. Importantly, two of the features we studied, i.e., spectral radius and modularity, differentiate the two seizure types several minutes prior to seizure onset. These findings suggest that these metrics do not simply pick up on an increase or decrease in the edge weights. Having said that, these metrics are not completely independent of the edge weights and are driven by their relative configuration within the network.

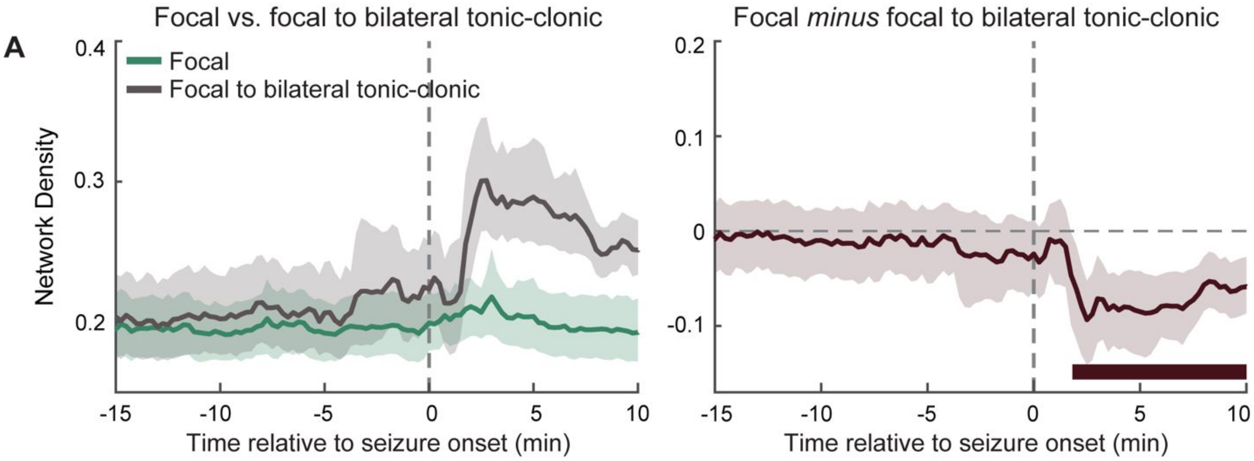

"**Figure 4 Various features of functional connectivity networks display distinct temporal changes as a function of seizure propagation dynamics**. Left panels illustrate a series of graph theoretical measures computed from networks of focal seizures that remain localized (n = 49) and from networks of focal to bilateral tonic-clonic seizures (n = 18). The time-varying differences observed in each of these features as a function of seizure types are plotted in the corresponding right panels. (A) The density of focal to bilateral tonic-clonic seizures is higher than that of focal seizures that remain focal, 1.75-10 minutes after seizure onset…"

We have made several modifications to the revised manuscript to incorporate the findings discussed above. Specifically, we have added details of the network density calculation in the **Materials and methods** section (see below) and have also revised the **Results** and **Discussion** sections accordingly.

"**Network density**

Network density describes the extent of connectedness in a network and is calculated as the ratio of actual and possible connections in a network. A density of 1 describes a fully connected network and a density of 0 describes no connectivity. Seizures have been shown to alter the synchronization, and therefore, overall connectivity and density of the functional network. We calculated network density as the ratio of total edge weights and number of possible edges. For a functional connectivity matrix A representing an undirected network with N nodes, the total number of possible edges is $N(N-1)/2$ and the total edge weight is the sum of the elements in the upper triangle of the connectivity matrix.

Networks with different density (and/or size) can have different network features[33]. Therefore, we compared network density across individual patients during interictal period to ensure that our findings are truly driven by the underlying changes in the functional connectivity

as a function of seizure types and are free of spurious results due to different network sizes and densities across patients (Supplementary Fig. 2).”

5. I found it odd that the heavily time-resolved networks were both smoothed across time and then also averaged – producing one network for the ictal period. I worry that by doing so, the authors miss out on the truly informative state changes / transition points that occur during the seizure (Fig 3A-B).

Thank you for your thoughtful comment. We first would like to clarify that the main analyses of our manuscript were computed based on temporally smoothed across 30-sec windows **without** additional averaging (see the subsection Functional connectivity networks in the Materials and methods). We agree with the reviewer that averaging of the temporally smoothed data to generate one network for the ictal period (as shown in Fig. 3A-B; copied below) could obscure important information concerning the underlying state changes and transitional activity that may have occurred during the seizure. With this concern in mind, we evaluated all of the network measures, including clustering coefficient and characteristic path length, in a sliding-windowed manner from 15 minutes before to 10 minutes after each seizure (Fig. 3C-D, Fig. 4-5).

We chose to perform the complementary analysis with additional averaging after temporal smoothing only for clustering coefficient (Fig. 3A) and characteristic path length (Fig. 3B) because we were interested in visualizing the temporal patterns of these two network features as the neural activity went into preictal, ictal, and postictal period. This analysis helped place our findings regarding changes in clustering coefficient and characteristic path length in context of past literature where these features were evaluated at different periods relative to seizure onsets (Kramer et al., 2010; Stam, 2014). By plotting these network features associated with focal seizures that stay focal against those of interictal activity (baselined condition; Fig. 3A-B, left panels), we can see an increase in clustering coefficient and a decrease in the characteristic path length following the onset of focal seizures that remain focal. Likewise, by overlaying these network features associated with focal to bilateral tonic-clonic seizures with those of interictal activity (Fig. 3A-B, right panels), we observe similar but more prominent patterns of results in the focal to bilateral tonic-clonic seizures. The method adopted in our study is similar to that adopted by past empirical work. For example, Sinha et al (*Brain*, 2017) used a similar analytical method for computing time-resolved network features that were shown to reflect information about the underlying state changes associated with seizure activity. Importantly, the authors demonstrated that such network features, computed in this manner, could inform clinicians about the likely origins of seizure activity.

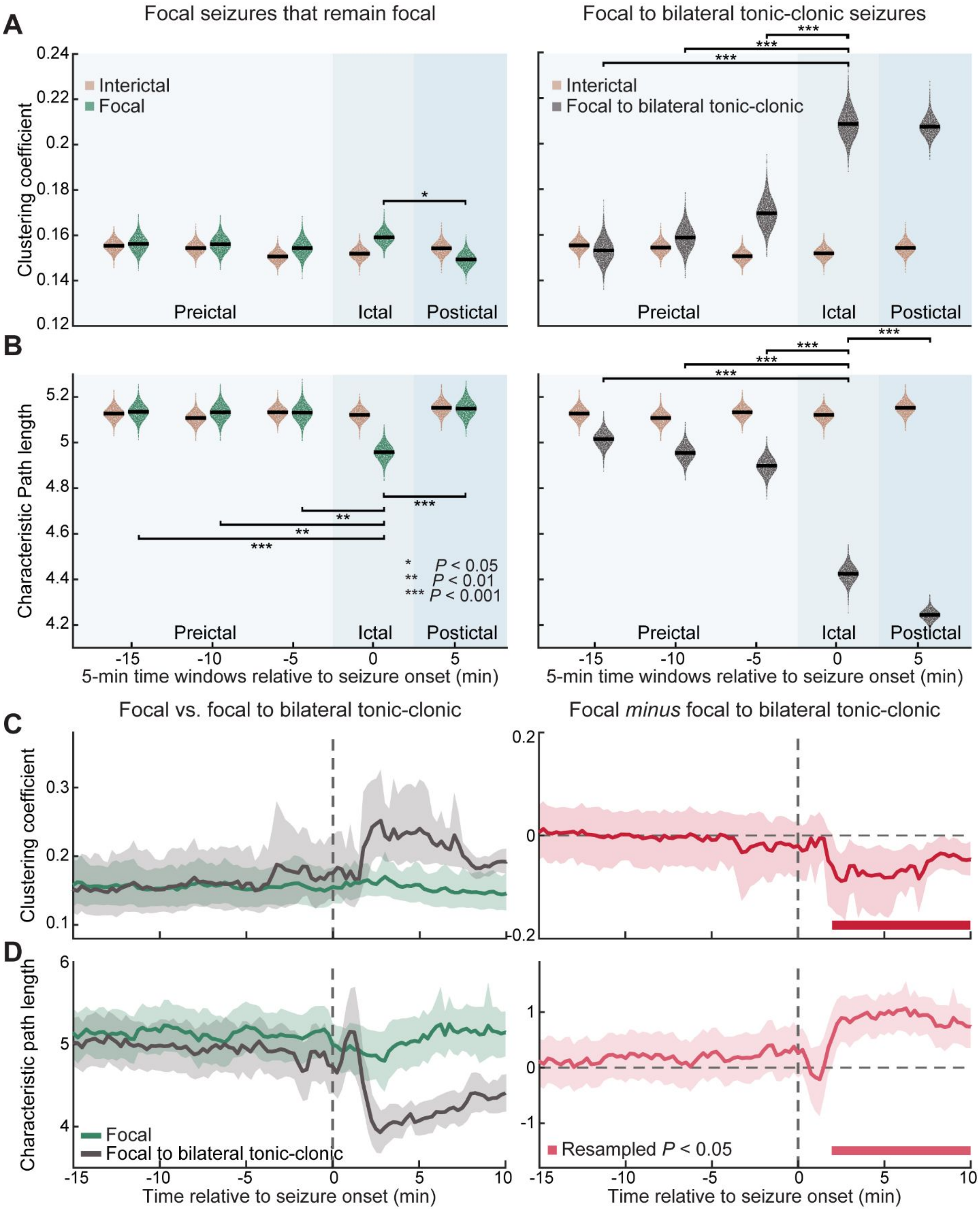

6. How were differences in seizure durations handled? Seizures of one type may be generally of different lengths than seizures of another type. The analysis time-locks to the onset, but then

it's unclear when the seizure is finished. Could a complimentary analysis time-lock to the termination/offset?

Traditionally, seizure durations are identified by clinicians while the ECoG signals are being recorded during clinical evaluations. However, such identification only occasionally occurred as clinicians generally place more emphasis on seizure onsets. When recorded, these clinically identified seizure durations are treated as ground truth and can be used as part of analyses of interest including one similar to what the reviewer suggested. One caveat that is worth noting regarding such analyses concerns the degree of reliability of reported seizure durations as precise identification is challenging. Reported durations across different clinicians are also often variable due to the complexity and heterogeneity concerning the nature of seizure termination.

In our data, seizure durations were only recorded for 7 (out of 49) focal seizures that stay localized and 9 (out of 18) focal to bilateral tonic-clonic seizures. In an attempt to address the reviewer's comment with this data limitation in mind, we computed durations across seizure types based on these available data points and found that average durations were 87.84 sec and 102.55 sec for focal seizures that remain localized and focal to bilateral tonic-clonic seizures, respectively. While this result is consistent with past reports (Mormann et al., *Clin Neurophysiol*, 2005; Jiruska et al., *J Physiol*, 2013), we did not include such information in our revised manuscript due to a large number of unreported durations as previously discussed.

We acknowledge such limitation and agree with the reviewer that complementary analyses where the ECoG signals are time-locked to seizure offset could be illuminating. Relatedly, mechanisms of seizure termination are currently not well understood and better insights into such process will be necessary for precise identification of seizure offset. Several computational tools, such as those grounded in dynamics systems, have also recently been developed to help determine seizure offset and duration. Such investigation is beyond the scope of this study, and we focused our analyses on the dynamic changes that occur around the onset of each seizure in order to gain insights into the mechanisms by which seizure activity of different types initializes. With this goal in mind, we addressed the difference in durations across individual seizures and across seizure types as follows. 1) For each individual seizure regardless of its type, we created a 25-minute data segment that include 10-minute worth of signals after its onset. Because seizures generally terminate within 3 minutes from the onsets, our methods ensure that all active seizure-related activity is contained in the data segments. 2). To address seizure durations (and other properties such as the number of recording electrodes) that inherently varied across individual recorded seizures, we chose to perform non-parametric statistical tests over traditional parametric tests as several assumptions required by the parametric tests are likely violated in our dataset (see **Statistical analysis** in **Materials and methods**). Notably, this is true for most, if not all ECoG datasets, and we constructed and employed a series of non-parametric, resampling analyses which were agnostic to variability across individual seizures. Specifically, for each temporal window of interest, we constructed data distributions of focal seizures that stay localized and focal to bilateral tonic-clonic seizures through a bootstrapping method (Efron & Tibshirani, *An Introduction to the Bootstrap*, 1994; Thompson, *Psychol Sch*, 2007). Resampling-based statistical comparisons were then computed on these seizure distributions. A distribution of 'seizure-free' data was also constructed in a similar manner for each temporal window from interictal data.

As extensively discussed in the field of statistics and computational neuroscience, resampling-based methods similar to the one adopted in our study are more conservative than

traditional tests and yield statistical results that likely capture the true differences of the underlying data. We have included a more detailed description of the performed bootstrapping procedure in the **Statistical analysis** section of the revised manuscript by writing:

"Given the diverse nature of seizure activity and the patient-specific procedures by which the placement and number of ECoG electrodes is determined, the recorded signals associated with individual seizures are inherently variable. It is well-documented that even within a single patient, different occurrences of seizure activity can vary in their durations, phases, and latencies of spread[11,53,54]. To account for such variability across individual seizures and to minimize statistical biases that could arise, for each network feature we used a bootstrapping procedure[55] to generate estimates of the mean and 95% confidence intervals (CIs), and made comparisons across seizure types in a time resolved manner. To perform these comparisons, we employed a rigorous statistical analysis as described in the following.
First, for each network measure and for each timepoint, we resampled with replacement at the level of individual seizures 10,000 times. Through these bootstrapping iterations, we generated an empirical estimate of the variability associated with each network measure of each seizure type over time (e.g., a bootstrapped distribution of the clustering coefficient associated with focal seizures that remain focal, a bootstrapped distribution of the clustering coefficient associated with focal to bilateral tonic-clonic seizures, and a bootstrapped distribution of the clustering coefficient associated with interictal activity). Next, to compare a particular network feature between conditions (e.g., focal seizures that remain focal vs. focal to bilateral tonic-clonic seizures), we calculated the difference at each time point between these bootstrapped data associated with each condition to obtain distributions of "differences" with respective means and CIs specific to each time point for each comparison (e.g., difference between the clustering coefficient of focal seizures that remain focal and that of focal to bilateral tonic-clonic seizures; Supplementary Fig. 4, Supplementary Fig. 5) . These distributions of "differences" were used to assess the *P* values and effect sizes, and to determine if a network feature significantly differentiates seizure types at a given time point.

To assess significance (though *P* values), we evaluated the observed distribution of bootstrapped "differences" against chance. To establish chance levels, we constructed null distributions non-parametrically for each complex network measure and for each time point by shuffling the condition labels 10,000 times, each time recomputing the difference between the seizure conditions on the shuffled data. Consequently, each null distribution centered at zero (e.g., the clustering coefficient does not differ between focal seizures that stay focal and focal to bilateral tonic-clonic seizures; difference of zero). We compared the bootstrapped empirical and null distributions by conducting 2 one-tailed tests against zero, i.e., mean[difference in clustering coefficients] < 0 and mean[difference in clustering coefficients] > 0) and then doubling the smaller *P* value. As the CIs for each comparison at each time point was achieved based on 10,000 bootstrapping iterations, the resolution of *P* values was constrained to a lower limit of $P \leq 0.0001$. A *P* values < 0.05 was deemed significant.

For each comparison, we estimated effect sizes in two different ways: (i) by calculating the mean of the bootstrapped "difference" distributions (e.g., mean[the clustering coefficient of focal seizures that remain focal *minus* the clustering coefficient of focal to bilateral tonic-clonic seizures]; Supplementary Fig. 4 and Supplementary Fig. 5); and (ii) by directly computing

Cohen's d[56,57] for the bootstrapped distributions of each network feature (e.g., the clustering coefficient of focal seizures that remain focal vs the clustering coefficient of focal to bilateral tonic-clonic seizures). For each of the significantly different statistical comparisons the smallest Cohen's d (Cohen's d_{min}) is reported. For each network measure, this value indicates the effect size associated with the temporal window at which the difference between seizure types were statistically significant but smallest. For example, if the clustering coefficient is lower in focal seizures that remain focal than in a focal to bilateral tonic-clonic seizures from time t_1 to t_2, the reported Cohen's d_{min} signifies the smallest effect size within this $[t_1\ t_2]$ period. The 95% CI associated with each Cohen's d_{min} is also reported."

7. Similarly, how can the authors be sure they are performing an apples-to-apples comparison of the distinct seizure phases. If one seizure spreads after 10 seconds, another seizure spreads at 30 seconds, and a focal seizure exhibited spread to adjacent electrodes after some other delay. Does the averaging approach dilute these changes?

We agree with the reviewer that this is an interesting and important conceptual question, however, information concerning the phases and latencies of seizure spread is rarely provided by clinicians. Without this ground truth, we were not able to include this level of investigation to our analyses of the current dataset. With this consideration in mind, we aligned all seizures to their corresponding onsets and focused our analyses and narratives on the network differences as a function of seizure types that emerged *before* and *after* the onset without making specific claims about the 'spread' period. We also believed that the use of resampling statistical analyses as discussed above sufficiently mitigated any biases in our findings that could otherwise emerge as a result of variability in the latencies of seizure spread.

While further research is needed to construct a universal framework in which seizure spread (and seizure offset) can be systematically indexed, some recent work from our group (Lainscsek et al., *Chaos*, 2019) and others has demonstrated promising findings where the spread of some seizures across electrodes can be assessed computationally (Carney et al., *Epilepsy Behav*, 2011; Cousyn et al., *Epilepsia*, 2021).

Comments from Reviewer 2

This study compares the time-evolving topological differences between focal to bilateral tonic colonic seizures (FBTCS) and focal only seizures. The authors use 25 mins window—15 mins before seizure onset and 10 mins after seizure onset—to compare graph theory metrics between the two seizure types. They compute time-varying functional connectivity in each patient and derive different graph theory metrics for epochs within 25 mins time window. The authors perform a group analysis to determine if the graph theory metrics are different between FBTCS and focal only seizures. They report that the average clustering coefficient, average path length, average degree, and assortativity coefficients differed after seizure onset whilst modularity, synchonizability, and spectral radius differed before seizure onset. However, I have concerns on the methodological approach and overinterpretation of results in the manuscript beyond what is shown by the data. These must be addressed before I can make a recommendation for publication. My comments are chronologically listed below:

We would like to thank the reviewer for their constructive and encouraging comments which have helped us rectify and improve the clarity of our methodological approach. In the following, we provide a point-by-point response to their concerns.

1. A major limitation of the study is that the authors are comparing graphs of different sizes and densities. Each patient will have a different number of electrodes implanted; therefore, network size and density in each patient will be different. Please include electrode types and the number of impanated electrodes as an additional column in Table 1. Graph metrics scale with network size and density, and it is well established that without incorporating appropriate controls, comparing networks of different sizes and densities induce biases. Please refer to van Wijk BCM, Stam CJ, Daffertshofer A (2010) Comparing Brain Networks of Different Size and Connectivity Density Using Graph Theory. PLoS ONE 5(10): e13701. https://doi.org/10.1371/journal.pone.0013701 The authors are suggested to correct for this statistical bias in their analysis.

This is a great point— we appreciate the reviewer for bringing it up. We agree that here we are comparing networks with different number of nodes which is a clinical decision. While ECoG is instrumental in epilepsy studies, unlike other imaging modalities, the number of electrodes is restricted to be as few as possible and also varies patient to patient. This puts an inherent challenge while working with the ECoG data and for full transparency, in the revised manuscript, we have now added the information on number of nodes for each patient in Table 1 (reproduced below). However, to ensure the validity of our findings: (i) we did control for the density and in the revised manuscript have now added a new supplementary Fig. 2 and have modified the Fig. 4 to explicitly discuss the effect of density; and (ii) opted for a rigorous statistical approach to perform non-parametric statistical tests.

In the following, we elaborate on these two points.

(i) First, the Supplementary Fig 2 (shown below) demonstrates that for interictal periods, functional connectivity networks extracted from different patients with different number of recording electrodes (i.e., different network sizes) did not show any significant difference in the network density. This comparison ensured that our reported findings are truly driven by the underlying changes in the functional connectivity as a function of seizure types and are free of spurious results due to different network sizes and densities across patients.

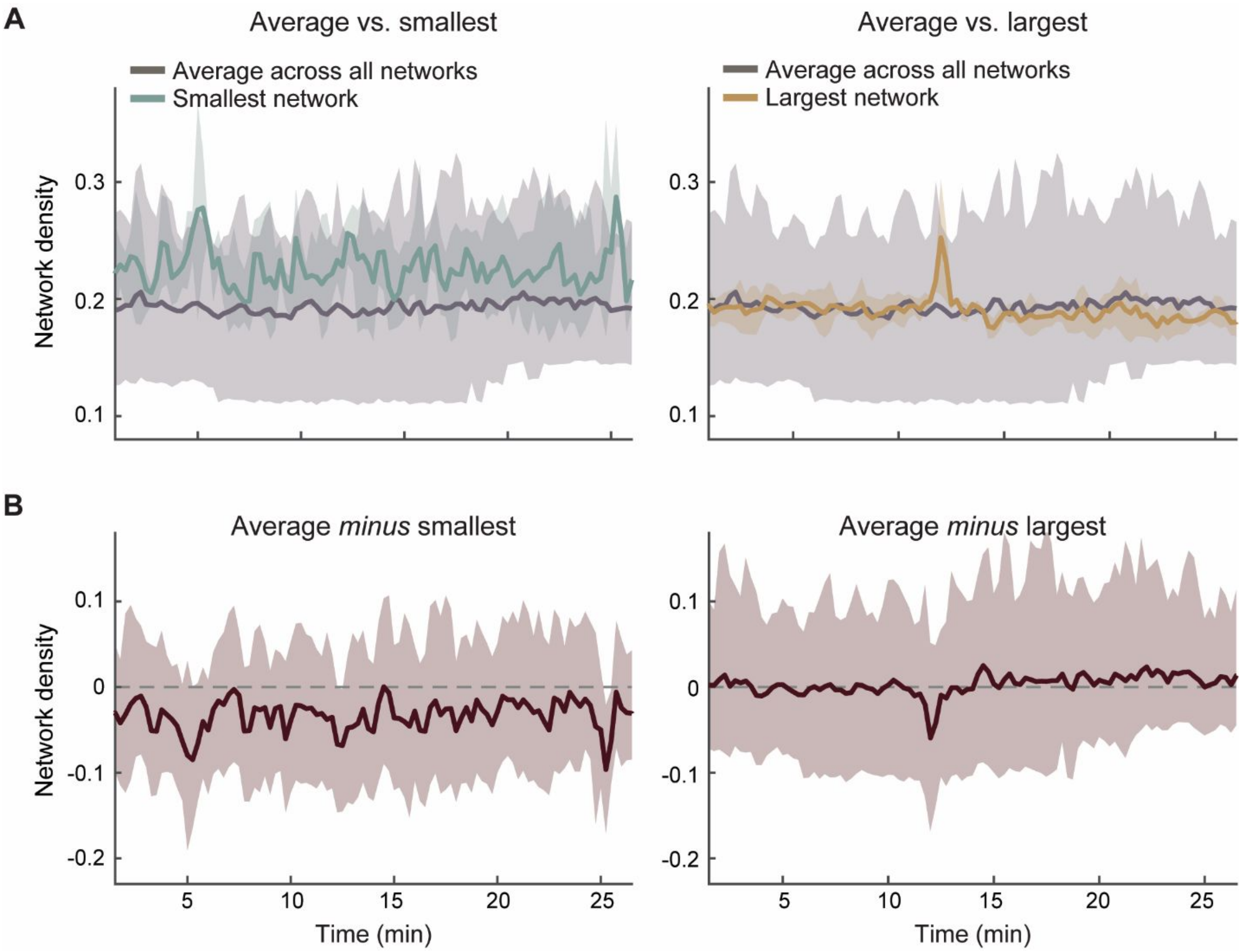

Fig. S2. Density extracted from interictal networks of different sizes. The density of smallest (i.e., 40 nodes; left panels) and largest (i.e., 118 nodes; right panels) is illustrated with the average density computed across all functional network. The density of the smallest interictal network did not differ from the average density. Similarly, the density of the largest interictal network also did not differ from that average density computed across interictal networks of different sizes. Statistical comparisons of density as a function of network sizes were computed through a bootstrapping procedure where the underlying data distribution of each network size was resampled at the level of individual seizures to established 95% confidence intervals (CIs).

Additionally, we added the density as one of the network features compared between the two seizure types and modified the Fig. 4 (the added panel is reproduced below) to include our findings. For seizures that spread bilaterally, we observe higher density (Figure 4A) than the seizures that remain focal shortly after the seizure onset. However, during the pre-onset period, the density does not differentiate the two types of seizures.

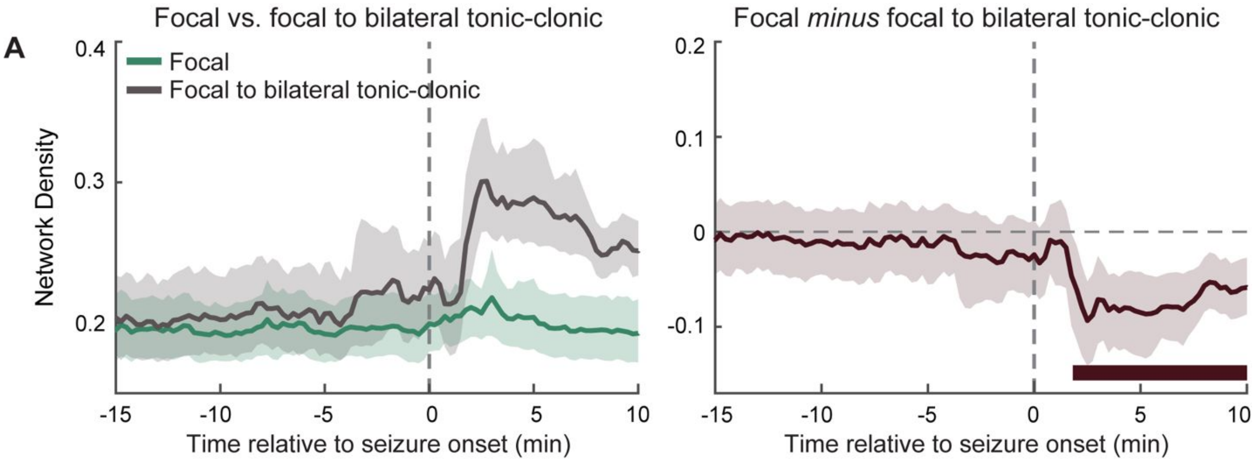

"**Figure 4 Various features of functional connectivity networks display distinct temporal changes as a function of seizure propagation dynamics**. Left panels illustrate a series of graph theoretical measures computed from networks of focal seizures that remain localized ($n = 49$) and from networks of focal to bilateral tonic-clonic seizures ($n = 18$). The time-varying differences observed in each of these features as a function of seizure types are plotted in the corresponding right panels. (A) The density of focal to bilateral tonic-clonic seizures is higher than that of focal seizures that remain focal, 1.75-10 minutes after seizure onset…"

We have made several modifications to the revised manuscript to include the findings discussed above. Specifically, we have added details of the density calculation in the **Materials and methods** section (see below) and have also revised the **Results** and **Discussion** sections accordingly.

"**Network density**

Network density describes the extent of connectedness in a network and is calculated as the ratio of actual and possible connections in a network. A density of 1 describes a fully connected network and a density of 0 describes no connectivity. Seizures have been shown to alter the synchronization, and therefore, overall connectivity and density of the functional network. We calculated network density as the ratio of total edge weights and number of possible edges. For a functional connectivity matrix A representing an undirected network with N nodes, the total number of possible edges is $N(N-1)/2$ and the total edge weight is the sum of the elements in the upper triangle of the connectivity matrix.

Networks with different density (and/or size) can have different network features[33]. Therefore, we compared network density across individual patients during interictal period to ensure that our findings are truly driven by the underlying changes in the functional connectivity as a function of seizure types and are free of spurious results due to different network sizes and densities across patients (Supplementary Fig. 2)."

(ii) To address the concern regarding the number of recording electrodes (and other properties such as seizure durations) that inherently varied across individuals, we chose to perform non-parametric statistical tests over traditional parametric tests as several assumptions required by the parametric tests are likely violated in our dataset

(see **Statistical analysis** in **Materials and methods**). Notably, this is true for most, if not all ECoG datasets, and we constructed and employed a series of non-parametric, resampling analyses which were agnostic to variability across individual seizures. Specifically, for each temporal window of interest, we constructed data distributions of focal seizures that stay localized and focal to bilateral tonic-clonic seizures through a bootstrapping method (Efron & Tibshirani, An Introduction to the Bootstrap, 1994; Thompson, *Psychol Sch*, 2007). Resampling-based statistical comparisons were then computed on these seizure distributions. A distribution of 'seizure-free' data was also constructed in a similar manner for each temporal window from interictal data. As extensively discussed in the field of statistics and computational neuroscience, resampling-based methods similar to the one adopted in our study are more conservative than traditional tests and yield statistical results that likely capture the true differences of the underlying data. We have included a more detailed description of the performed bootstrapping procedure in the **Statistical analysis** section of the revised manuscript.

Table I Patient Profiles

Patient	Sex	Age at onset/ surgery	Etiology	Seizure type (#)	Seizure onset zone	Electrode type (#)	Resection areas	Outcome
1	F	15/46	Dysplasia	focal to bilateral (5)	Anterior temporal	Depths/Grids (113)	Right anterior temporal	I
2	F	42/55	n.a	focal to bilateral (3)	Temporal	Depths (56)	None	I
3	F	17/45	n.a	focal (1); focal to bilateral (2)	Temporal	Depths (40)	None	n.a
4	M	8/23	n.a	focal (10)	Frontal	Depths (80)	None	III
5	M	14/35	n.a	focal (9); focal to bilateral (2)	Temporal	Depths (80)	Right anterior temporal	n.a
6	F	12/32	n.a	focal (15)	Temporal	Depths (112)	Right anterior temporal	II
7	F	7/23	n.a	focal (6)	Frontal	Depths (80)	Left frontal	IV
8	F	10/27	n.a	focal (1)	Unknown	Depths (80)	Left frontal	IV
9	F	8/19	MTS	focal (1)	Anterior temporal	Grids (60)	Left anterior temporal	III
10	F	14/31	n.a	focal to bilateral (2)	Temporal	Depths (48)	Right anterior temporal	I
11	F	1/21	Stroke	focal (2)	Temporal	Depths (118)	Left temporal	IV
12	F	9/42	n.a	focal (2)	Frontal	Depths (76)	None	II
13	M	39/47	n.a	focal to bilateral (3)	Posterior temporal	Depths (48)	Right temporal	I
14	F	50/59	n.a	focal (2); focal to bilateral (1)	Posterior temporal	Depths (80)	Left temporal	I

2. Introduction:
"differential neural dynamics as well as the distinct cognitive and behavioral changes associated" There are no data in this manuscript to support any association with cognition or behavior. Focal only seizures also associate with cognition and behavior. I suggest removing all claims about association with cognition/behavior from the manuscript.

We have addressed this concern by removing all text associated with cognitive and behavioral changes from the revised manuscript.

3. Data preprocessing:
"For comparison with relatively 'seizure-free' (interictal) activity, we extracted an equal number of interictal activity windows with the same duration." Is this correct? Equal number means 67 interictal activity preceding 67 seizures. Please clarify was the same interictal epoch also used with other seizures.

This is correct. As previously discussed as part of our response to comment #1, 67 interictal activity windows (with the same length in duration) preceding 67 seizures were used for baseline comparison purposes. Thus, these resulted in 67 individual interictal graph networks from which network measures (e.g., modularity) were computed. Differences between each network measure associated with interictal activity (e.g., the modularity of interictal activity) and seizures (e.g., modularity of focal seizures that remain focal; and modularity of focal to bilateral tonic-clonic seizures) were evaluated using a bootstrapping procedure.

We have further clarified this point in the revised manuscript by writing:

"Specifically, for each of the 67 seizures (49 focal and 18 focal to bilateral tonic-clonic) from which we extracted a 25-minute segment (from 15 minutes before to 10 minutes after the onset), we also epoched a 25-minute segment of data that was at least 60 minutes away from the seizure onset and offset." in the **Data preprocessing** subsection and by writing:

"For each of the seizures (49 focal and 18 focal to bilateral tonic-clonic) and interictal activity (67 epochs), we constructed a series of weighted, symmetric (undirected) connectivity matrices A representing functional correlations across all recording electrodes." In the **Graph theoretical network analysis** subsection.

4. Functional connectivity networks:
Negative Pearson correlation coefficient does not necessary imply long-range inhibitions. Negative values could also appear due to phase lags. It is a simply a choice to set negative values to zero, but they can't be justified as having no contributions to epileptogenic effect. Authors are suggested to reword this sentence.

We appreciate the reviewer's suggestion and have rephrased this sentence by writing:

"Negative correlation values were then set to zero following traditional modeling choices adopted in previous studies[3,32] examining simulation of the epileptogenic effect."

5. Assessing small-world architecture:
Authors are not computing the small world metric anywhere in their analysis. As such, a qualitative comparison by observing clustering and path length is not suffice. I suggest to either remove all qualitative references to small work architecture or quantify it.

We would like to apologize for this oversight. We agree that in the previous version of the manuscript, we did not calculate small-worldness, as defined mathematically by comparing clustering coefficient and path length of a given network to a comparable random network. We also agree that it is not sufficient to infer the extent of small-world characteristic simply through evaluation of clustering coefficients and characteristic path lengths.

In the revised manuscript, we have added a new Supplementary Fig. 3, where we explicitly illustrated the computed small-worldness overtime from which a comparison of small-world characteristics between the two seizure types can be made.

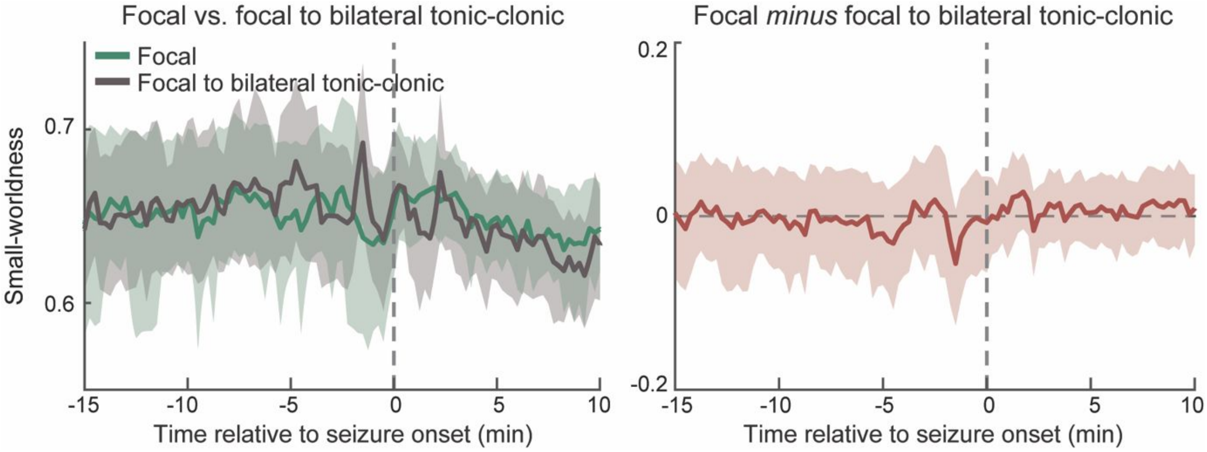

Fig. S3. Small-world characteristics of focal seizure subtypes. We computed small-worldness as the ratio of CC and PL relative to that of a random network. Random networks were generated at each time point by redistributing the edge weights of the original network such that the average degree is preserved. Focal to bilateral tonic-clonic seizures exhibit similar temporal patterns of small-world characteristics as focal seizures that remain localized. Statistical comparisons of small-world characteristics as a function of seizure types were computed through a bootstrapping procedure where the underlying data distribution was resampled at the level of individual seizures to established 95% confidence intervals (CIs).

We did not find the measure of small-worldness to be an effective candidate for comparing network reconfigurations associated with focal seizures that remain focal and focal seizures with bilateral spread. We have modified the manuscript to include this finding and have removed all qualitative references to small-world architecture in the revised manuscript.

6. Modularity:
Please add more details on modularity. What algorithm was used? How was the optimal modularity decided?

To address these points, we have added the following text to the revised manuscript:

"Mathematically, the modularity metric (Q) represents the number of edges falling within modules minus the expected number in an equivalent network with edges placed at random. It is estimated as follows: $Q = \frac{1}{2m}\sum_{ij}\left(A_{ij} - \gamma\frac{k_i k_j}{2m}\right)\delta(c_i, c_j)$, where A_{ij} represents the connectivity between node i and j, k_i represents the degree of the node i, m represents the total number of edges in the network, γ represents a resolution parameter, and $\delta(c_i, c_j)$ equals 1 if nodes i and j

are in the same community, and equals 0 otherwise[51]. Here, to detect modules and obtain a value of modularity for functional brain networks, we used the spectral approach described by Newman and implemented it through Brain Connectivity Toolbox function *modularity_und*.[51,52] The resolution parameter γ was chosen to be 1."

7. Spectral radius and Synchronizability:
Can the authors plot a scatter plot between Spectral radius and synchronizability metrics? These measures seem to be highly correlated.

We are very thankful to the reviewer for drawing our attention to the similarity between these two metrics in the previous version if the manuscript. We included synchronizability in our analysis to draw a comparison with the findings from Khambhati et al. (Virtual Cortical Resection Reveals Push-Pull Network Control Preceding Seizure Evolution, Neuron, Volume 91, Issue 5, 7 September 2016, Pages 1170-1182 https://doi.org/10.1016/j.neuron.2016.07.039), however, found out that our implementation of synchronizability was different from that used in this previous study. In the revised manuscript, we corrected the synchronizability computations, accordingly. Here, we have included the revised Fig. 4 with corrected synchronizability calculations. As one may expect, spectral radius and synchronizability are not correlated and they exhibit different temporal profiles.

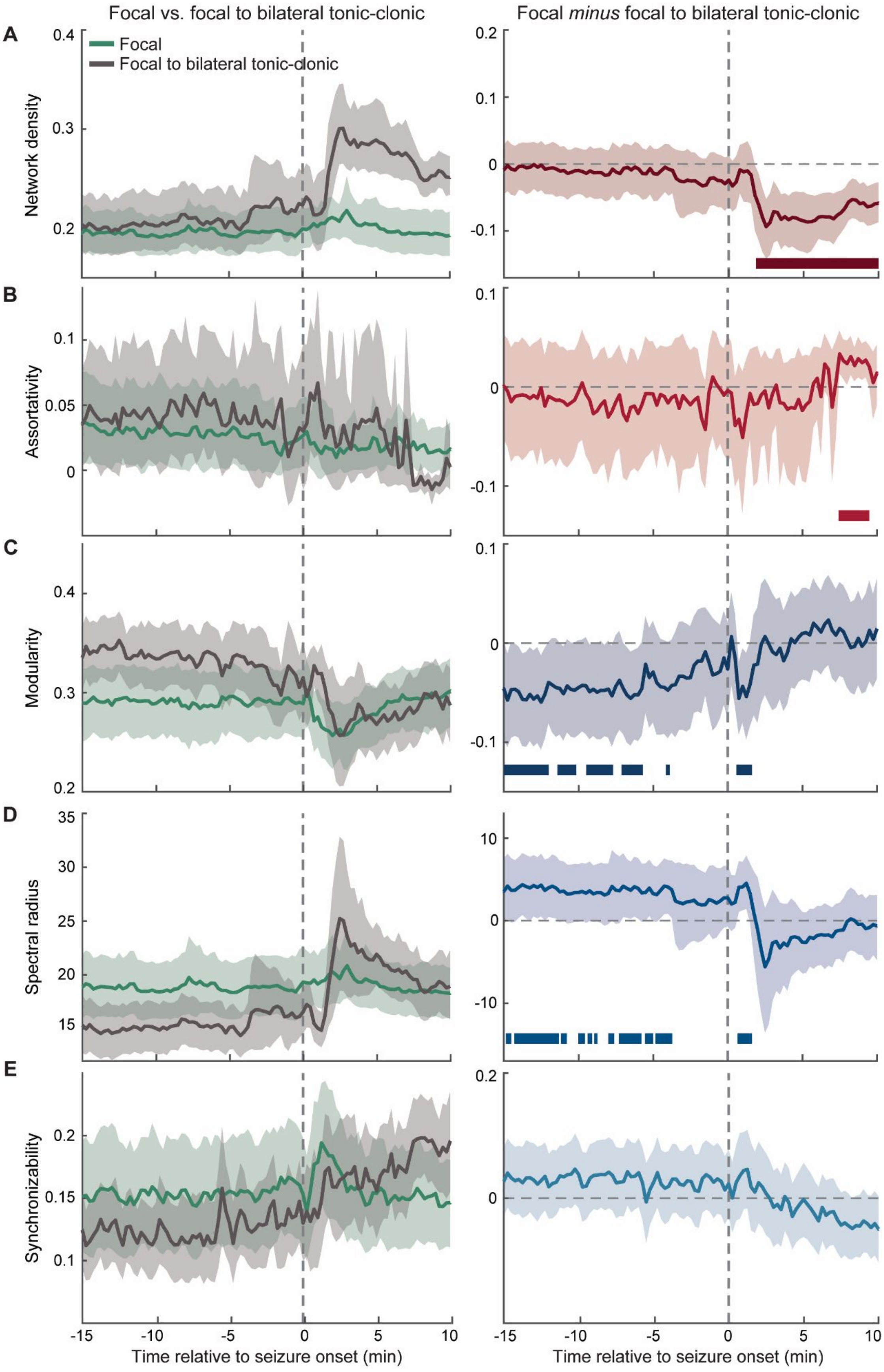

Focal vs. focal to bilateral tonic-clonic
Focal *minus* focal to bilateral tonic-clonic
A
B
C
D
E
Focal
Focal to bilateral tonic-clonic
Network density
Assortativity
Modularity
Spectral radius
Synchronizability
Time relative to seizure onset (min)
Time relative to seizure onset (min)

Below we have also included the scatter plots to further illustrate the relationship between these metrics as a function of different seizure types.

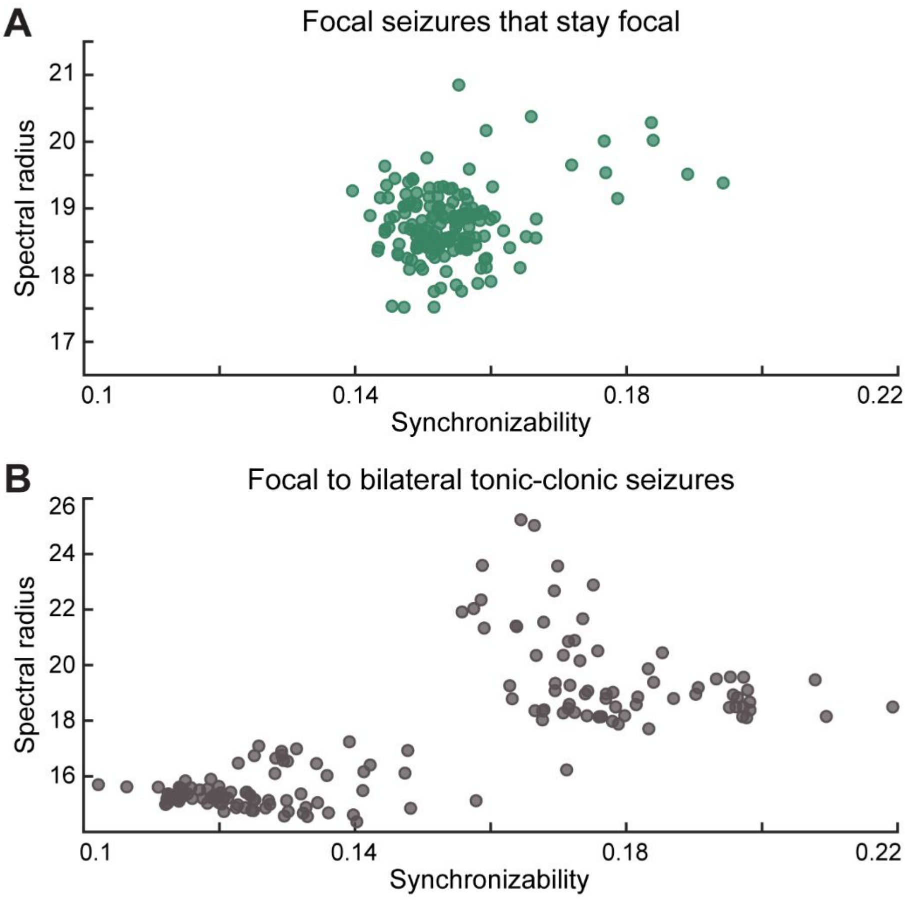

Finally, we have also revised the **Results** and **Discussion** sections to reflect these synchronizability results.

8. Statistical analysis:
What checks were incorporated for dealing with multiple comparisons? Authors are suggested to report effect size and its 95% CI in addition to p-values.

We apologize for our insufficient explanation regarding the statistical analyses performed in our study. We performed resampling statistical tests instead of traditional parametric tests because our dataset does not meet the assumptions of normality, equal variance, independence, and no outliers. Notably, this is true for most, if not all ECoG datasets, and we constructed and employed a series of non-parametric, resampling analyses that were agnostic to variability across individual seizures of each type. Specifically, for each of 158 individual temporal windows of interest from 15 minutes before to 10 minutes after seizure onsets, we constructed data distributions of focal seizures that stay localized and focal to bilateral tonic-clonic seizures through a bootstrapping method (Mooney & Duval, 1993; Kelly, 2005). Resampling-based statistical comparisons were then computed on these seizure distributions with respect to the null distribution of zeros (i.e., the two seizure distributions completely overlapped yielding a difference of zero). Distributions of 'seizure-free' data associated with individual temporal

windows were also constructed in a similar manner from interictal data. In this way, resampled data distributions from which statistical differences were computed were independently drawn which minimize temporal dependence and biases. Importantly, resampling statistics were computed in a pairwise manner separately for each temporal window (i.e., focal seizures that stay focal vs. focal to bilateral tonic-clonic seizures; focal seizures that stay focal vs. interictal activity; and focal to bilateral tonic-clonic seizures vs. interictal activity). Together, these analytical steps circumvented the need for multiple comparison procedures while minimizing possible biases and ensuring rigorous and conservative evaluations of group differences in our dataset (Westfall & Young, 1993). As extensively discussed in the field of statistics and computational neuroscience, resampling-based methods similar to the one adopted in our study are more conservative than conventional parametric tests and yield statistical results that likely capture the true differences of the underlying data (Mooney & Duval, 1993; Kelly, 2005; Fieberg et al., 2020). To better convey these points, we have added more information to the **Statistical analysis** section of the revised manuscript by writing:

"Given the diverse nature of seizure activity and the patient-specific procedures by which the placement and number of ECoG electrodes is determined, the recorded signals associated with individual seizures are inherently variable. It is well-documented that even within a single patient, different occurrences of seizure activity can vary in their durations, phases, and latencies of spread[11,53,54]. To account for such variability across individual seizures and to minimize statistical biases that could arise, for each network feature we used a bootstrapping procedure[55] to generate estimates of the mean and 95% confidence intervals (CIs), and made comparisons across seizure types in a time resolved manner. To perform these comparisons, we employed a rigorous statistical analysis as described in the following.
First, for each network measure and for each timepoint, we resampled with replacement at the level of individual seizures 10,000 times. Through these bootstrapping iterations, we generated an empirical estimate of the variability associated with each network measure of each seizure type over time (e.g., a bootstrapped distribution of the clustering coefficient associated with focal seizures that remain focal, a bootstrapped distribution of the clustering coefficient associated with focal to bilateral tonic-clonic seizures, and a bootstrapped distribution of the clustering coefficient associated with interictal activity). Next, to compare a particular network feature between conditions (e.g., focal seizures that remain focal vs. focal to bilateral tonic-clonic seizures), we calculated the difference at each time point between these bootstrapped data associated with each condition to obtain distributions of "differences" with respective means and CIs specific to each time point for each comparison (e.g., difference between the clustering coefficient of focal seizures that remain focal and that of focal to bilateral tonic-clonic seizures; Supplementary Fig. 4, Supplementary Fig. 5) . These distributions of "differences" were used to assess the *P* values and effect sizes, and to determine if a network feature significantly differentiates seizure types at a given time point.

To assess significance (though *P* values), we evaluated the observed distribution of bootstrapped "differences" against chance. To establish chance levels, we constructed null distributions non-parametrically for each complex network measure and for each time point by shuffling the condition labels 10,000 times, each time recomputing the difference between the seizure conditions on the shuffled data. Consequently, each null distribution centered at zero (e.g., the clustering coefficient does not differ between focal seizures that stay focal and focal to bilateral

tonic-clonic seizures; difference of zero). We compared the bootstrapped empirical and null distributions by conducting 2 one-tailed tests against zero, i.e., mean[difference in clustering coefficients] < 0 and mean[difference in clustering coefficients] > 0) and then doubling the smaller P value. As the CIs for each comparison at each time point was achieved based on 10,000 bootstrapping iterations, the resolution of P values was constrained to a lower limit of $P \leq 0.0001$. A P values < 0.05 was deemed significant.

For each comparison, we estimated effect sizes in two different ways: (i) by calculating the mean of the bootstrapped "difference" distributions (e.g., mean[the clustering coefficient of focal seizures that remain focal *minus* the clustering coefficient of focal to bilateral tonic-clonic seizures]; Supplementary Fig. 4 and Supplementary Fig. 5); and (ii) by directly computing Cohen's *d*[56,57] for the bootstrapped distributions of each network feature (e.g., the clustering coefficient of focal seizures that remain focal vs the clustering coefficient of focal to bilateral tonic-clonic seizures). For each of the significantly different statistical comparisons the smallest Cohen's *d* (Cohen's d_{min}) is reported. For each network measure, this value indicates the effect size associated with the temporal window at which the difference between seizure types were statistically significant but smallest. For example, if the clustering coefficient is lower in focal seizures that remain focal than in a focal to bilateral tonic-clonic seizures from time t_1 to t_2, the reported Cohen's d_{min} signifies the smallest effect size within this [t_1 t_2] period. The 95% CI associated with each Cohen's d_{min} is also reported."

We also added new Supplementary Fig. 4 and 5 to demonstrate the effect sizes.

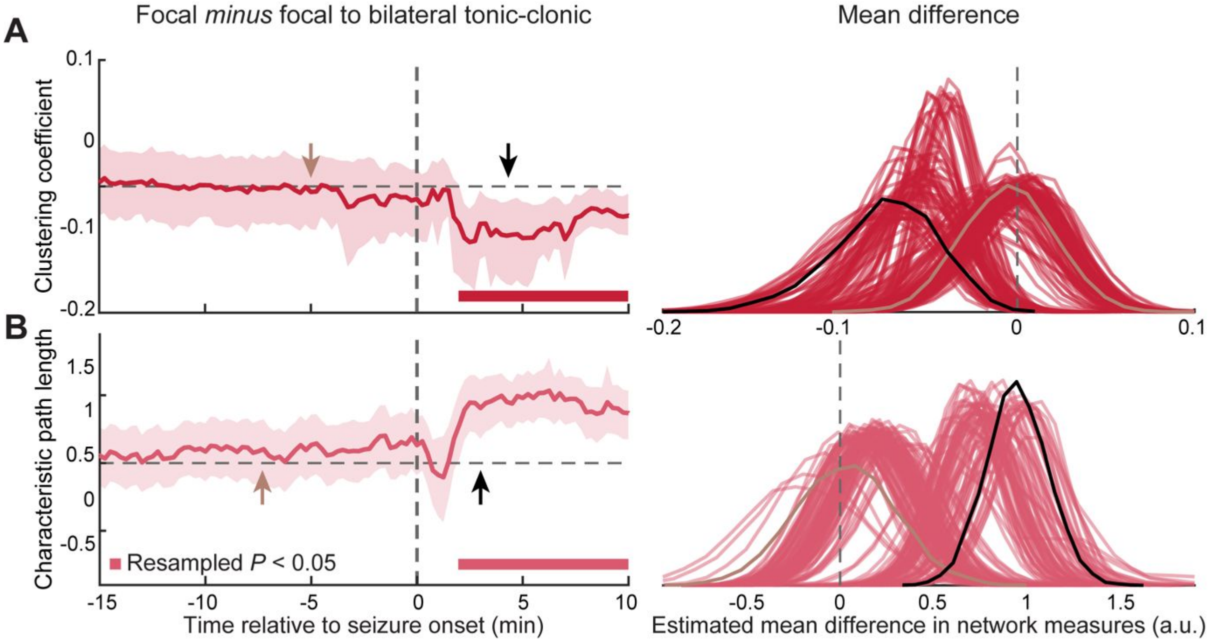

Fig. S4. Effect sizes of seizure-type specific reconfigurations in clustering coefficient and characteristic path length. To visualize the effect sizes associated with seizure-type specific differences in clustering coefficient (A) and characteristic path length (B) overtime (left panels), the bootstrapped distributions associated with the mean difference of individual timepoints are plotted (right panels). For each of the network measure, the brown and black arrows indicated a sample timepoint where the mean difference between seizure types were not significantly different and significantly different from the bootstrapped null difference of zero, respectively. The distributions of these mean differences are

illustrated in the corresponding right panel. Statistical comparisons of network measures as a function of seizure types were computed through a bootstrapping procedure where the underlying data distribution of each network measure was resampled at the level of individual seizures to establish 95% confidence intervals (CIs). Error bars indicate 95% CIs across individual seizures in each condition and solid bars show resampled $P < 0.05$.

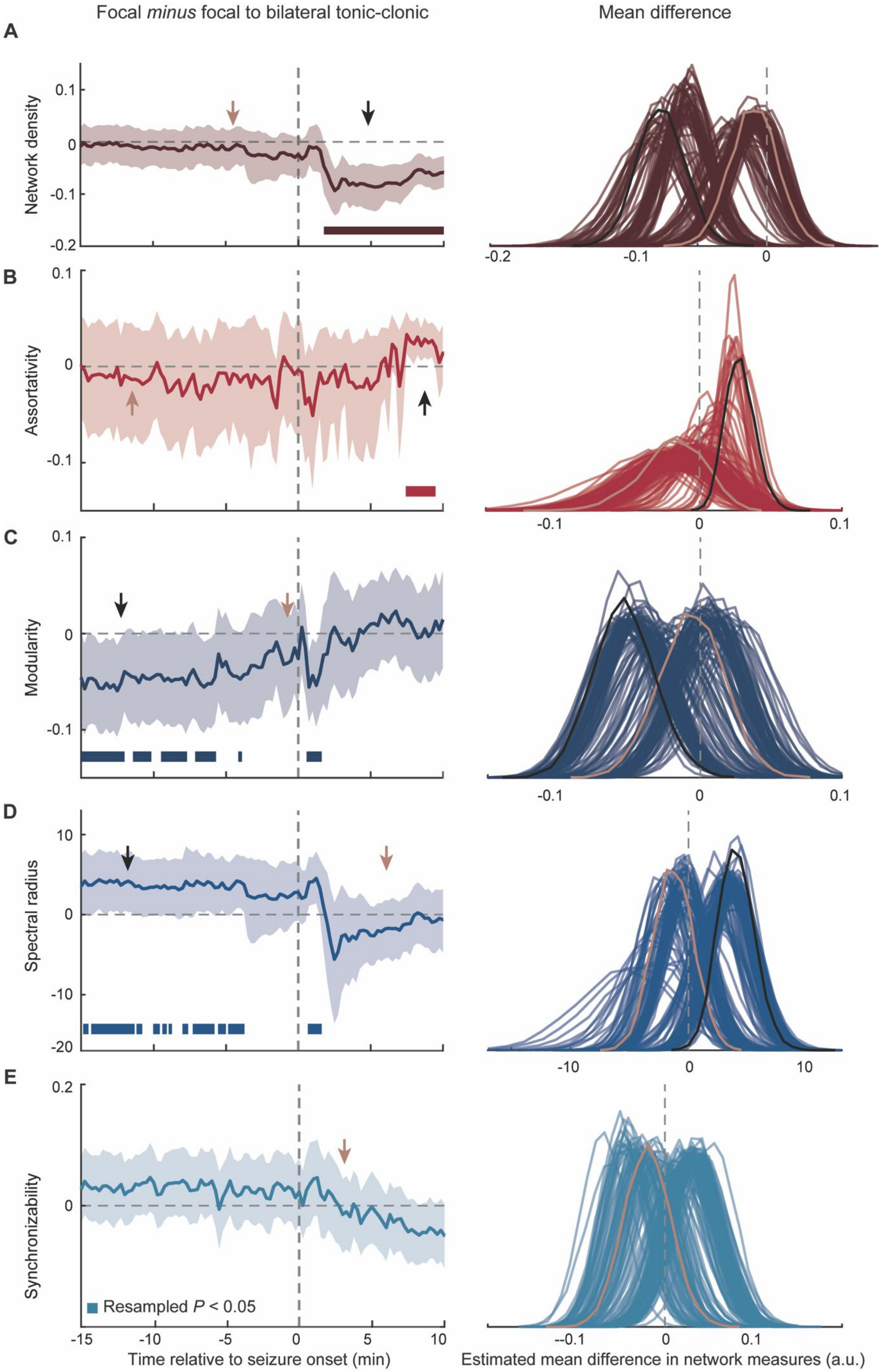
Focal minus focal to bilateral tonic-clonic
Mean difference
A
Network density
B
Assortativity
C
Modularity
D
Spectral radius
E
Synchronizability
Resampled P < 0.05
Time relative to seizure onset (min)
Estimated mean difference in network measures (a.u.)

Fig. S5. Effect sizes of seizure-type specific reconfigurations in various features of functional connectivity networks. Left panels illustrate the seizure-type specific differences in network density (A), associativity (B), modularity (C), spectral radius (D), and synchronizability (E). The bootstrapped distributions associated with the mean difference of individual timepoints are plotted in the corresponding right panels. For each of the network measure, the brown and black arrows indicated a sample timepoint where the mean difference between seizure types were not significantly different and significantly different from the bootstrapped null difference of zero, respectively. The distributions of these mean differences are illustrated in the corresponding right panel. Statistical comparisons of network measures as a function of seizure types were computed through a bootstrapping procedure where the underlying data distribution of each network measure was resampled at the level of individual seizures to establish 95% confidence intervals (CIs). Error bars indicate 95% CIs across individual seizures in each condition and solid bars show resampled $P < 0.05$.

9. Results: More prominent small-world connectivity links to bilateral spread of seizure activity "increased small-world connectivity has been proposed as a potential driver for the propagation of pathological synchronous activity" I don't think that the interpretation about small-world connectivity as a potential driver for the propagation is correct. Can the authors clarify what do they mean?

In addressing the reviewer's previous comment concerning small-world architecture, we have replaced any qualitative references to small-world connectivity in the revised manuscript.

10. Fig. 3 A and B: Please show the data points on the plot.

We have included individual data points to the plots in Fig. 3A and 3B. A copy of the revised panels is shown below. Note that individual data points are difficult to see in these plots because (i) each of the plot was made up of 10,000 resampled data points (see **Statistical Analysis**); and (ii) we prioritized equating the y-axis scales across interictal activity and the two focal seizure types to facilitate visual evaluation of the temporal patterns of the clustering coefficient and the characteristic path length associated with each seizure type.

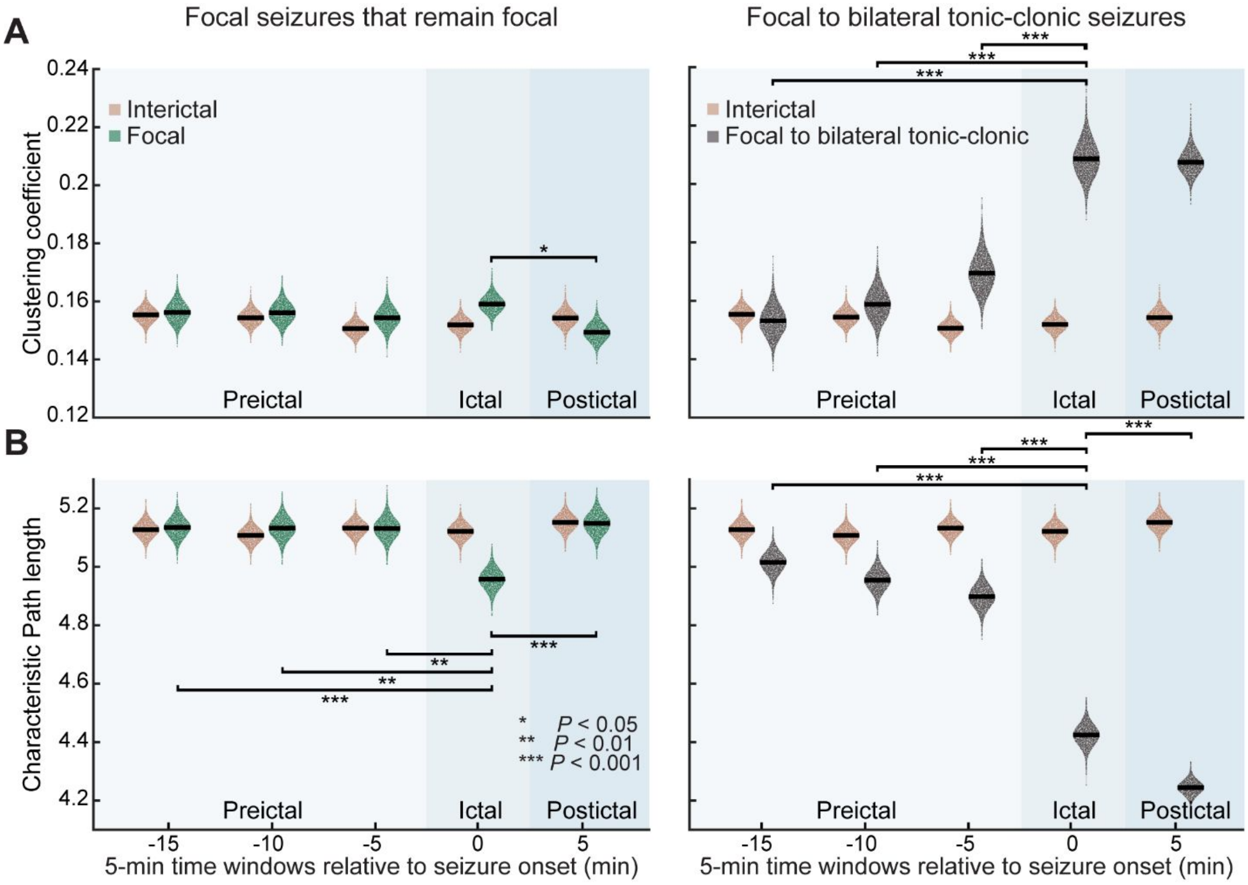

11. Re: "Our results reveal that both focal seizures that remain focal and focal to bilateral tonic-clonic seizures displayed higher small world connectivity during ictal periods when compared to seizure-free (interictal) activity…" and "Notably, the observed differences regarding the manner in which the small-world architecture increased in the networks of focal seizures with constrained and unconstrained dynamics were our first evidence in support of the notion that there may exist network-level signatures that contained information about the distinct propagation mechanisms of focal seizures." See my comment above, there are no computations for small world index. Please state the results directly in terms of clustering coefficient and path length.

We apologize for the oversight and have revised our manuscript accordingly.

12. "…..to an increase in the efficiency of network communication, as illustrated by the…" why not use the network communicability metric to study this?

We used the term 'network communication' in a loose sense to refer to how individual nodes in the functional brain network correlate to one another. We agree with the reviewer that our analyses did not directly probe this quantity and have, therefore, revised this sentence accordingly.

13. "These results suggested that close to seizure termination, the networks of focal seizures with unconstrained dynamics underwent reduced robustness, rendering them more susceptible to network disruptions. These findings could potentially account for the more extensive behavioral

abnormalities and cognitive deficits often observed after patients experience episodes of focal to bilateral tonic-clonic seizures." This is an overinterpretation of the results. How do you compute robustness? Behavioral abnormalities and cognitive deficits are also common in focal seizures. This statement should be removed.

We agree with the reviewer that we did not directly compute robustness and we merely infered such quality through assortativity. We have removed this sentence following the reviewer's suggestion and have now also modified the **Discussion** subsection **Network alterations track temporal evolution of focal seizures** to include a more careful interpretation of the assortativity results. The revised text is reproduced here:

"Assortativity can be directly linked to the robustness of the network, in terms of its ability to remain connected[60]. A failure of a hub node in an assortative network would leave other hub nodes connected to one another, minimizing the chance of the network as a whole to become disconnected. However, in case of a seizure that spreads bilaterally, emergence of a disassortative network likely reflects a mechanism to reduce this "robustness" by restricting the connectivity among network hubs."

14. Results: Alterations in global network features preceding the onset predict propagation dynamics of focal seizures "predictive manner" there are no predictions anywhere in this manuscript. The authors show associations and they should be reported as such.

We have revised the **Results** section by replacing any claims or references associated with the predictive power of our network measures. Instead, we have now clearly stated that we demonstrated associations rather than causality.

15. Figure 4D and E are exact same graph. Is spectral radius redundant?

Again, we are very thankful to the reviewer for drawing our attention to the similarity between these two metrics. As we mentioned in the response to comment #7, we included synchronizability in our analysis to draw a comparison with the findings from Khambhati et al. (Virtual Cortical Resection Reveals Push-Pull Network Control Preceding Seizure Evolution, *Neuron*, Volume 91, Issue 5, 7 September 2016, Pages 1170-1182. https://doi.org/10.1016/j.neuron.2016.07.039). However, we realized that our early implementation of synchronizability was different from the method adopted in this past study. In the revised manuscript, we corrected the computations for synchronizability, accordingly. Fig. 4 now shows the corrected synchronizability calculations, and as one may expect, spectral radius and synchronizability exhibit different dynamics overtime.

16. Figure 6 is totally unclear to me. Can the authors plot the graphs in the same axis to facilitate visual comparison? The different visualization might be more suitable.

Thank you for this suggestion, we have now modified Figure 6 (included below) for a better visualization.

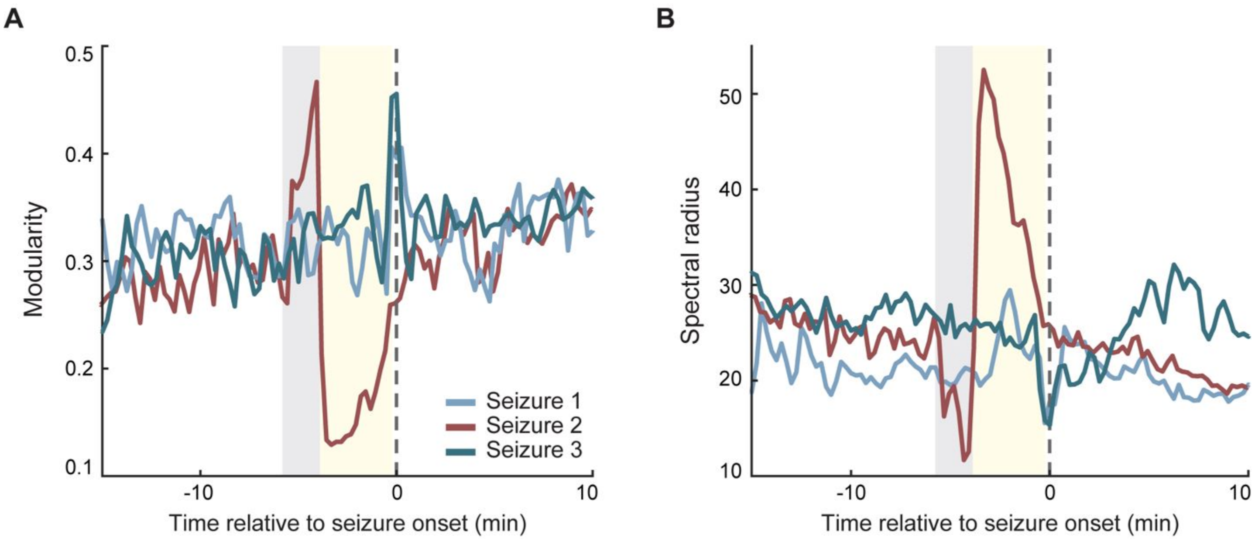

Figure 6 Distinct patterns of network properties across seizure types at a single-seizure level. Modularity and spectral radius extracted from networks associated with three seizures that share similar onset regions recorded from a sample patient. Seizure 1 and seizure 3 are categorized by an epileptologist as focal seizures that remain focal (sample recordings of seizure 1 is illustrated in Fig. 1B (left)), whereas seizure 2 is categorized as a focal to bilateral tonic-clonic seizure (sample recordings of seizure 2 is also illustrated in Fig. 1B (right)). Seizure 1 and seizure 3 exhibit similar patterns of modularity and spectral radius overtime, and these temporal dynamics differ from those of seizure 2. The grey shaded region highlights the robust signatures associated with the bilateral spread of a focal seizure (i.e., increase in modularity and decrease in spectral radius) and is followed by network changes which are likely seizure- and/or patient-specific.

We have also modified the **Results** subsection **Complementary temporal reconfigurations within the functional connectivity networks sculpt seizure dynamics** to better articulate the importance of Fig. 6. We wrote:

"To further highlight the utility and robustness of these features in assessing the likelihood of a focal seizure to spread bilaterally, we evaluated modularity and spectral radius at a single-seizure level in three seizures that shared similar onset regions in the left hemisphere and were recorded from a single patient (Fig.1 and Fig. 6). Specifically, seizure 2 bilaterally propagated after the onset while seizure 1 and 3 remained localized within the left hemisphere. We showed that seizure 1 and 3 exhibit similar dynamics of modularity and spectral radius over time, and these temporal patterns differ from those of seizure 2. For seizure 2, we observe an early sharp increase in modularity and a decrease in spectral radius consistent with the findings across seizures (Fig. 5), further highlighting the robustness of our results (grey shaded area). These patterns were followed by an abrupt decrease in modularity and a sharp increase in spectral radius (Fig. 6; yellow shaded area) which could be seizure- and/or patient-specific features. These results further suggested that network measures could potentially be used to characterize distinct neural dynamics across different types of focal seizures, even on a single-seizure basis."

17. Discussion:

a. "predicted the extent of diffusion dynamics of an impending seizure as well as isolated architectural changes" The manuscript doesn't make any predictions; the results illustrates

associations/correlates. There is also no way to determine the extent of seizure using average network measures; FBTCS have different extent. Please reword this statement.

We have addressed this comment by writing:

"We demonstrated distinct network-level signatures that were associated with the extent of diffusion dynamics of an impending seizure as well as isolated architectural changes within the functional connectivity networks that emerged as the seizures terminated."

b. "Furthermore, our findings provide a rationale for utilizing network features to guide clinical diagnosis of seizure subtypes and effective intervention strategies to constrain propagation of seizure activity." Overstatement about seizure subtypes. The authors are comparing only two seizure types FBTCS and focal only. The argument about intervention strategies is purely speculative and is not supported by data in this manuscript.

We appreciate the reviewer's comment and have addressed this comment by writing:

"Furthermore, our findings provide a rationale for a potential use of network features to help guide clinical diagnosis of focal seizures with different propagation mechanisms (focal seizures that remain local vs. focal to bilateral tonic-clonic seizures). Incorporation of such network measures with other biomarkers of neural state changes[4,54,66] could also potentially lead to improvement of effective intervention strategies to constrain propagation of seizure activity."

c. Network alterations track temporal evolution of focal seizures:
"Specifically, our findings revealed a relationship between the extent of small-world architecture and the diffusivity of seizure activity after the onset." There is no data to quantify small-world architecture and diffusivity of seizure activity: it is only FBTCS or focal only distinction. These statements should be tempered/corrected.

We have addressed this comment by writing:

"Specifically, our findings revealed a relationship between the clustering coefficient, characteristic path length, and a binary measure of diffusivity of seizure activity after the onset (i.e., whether the seizure activity would remain within the same hemisphere or spread transcollosally)"

d. "We argue that our approach provides an objective framework not only for better understanding the neural dynamics underlying evolution of seizures but also for determining whether and when a clinical intervention should be implemented to manage and control a spread of an impending seizure." The statement about clinical intervention is purely speculative. Please discuss how the data in this manuscript support a tailored intervention guiding clinical intervention.

We agree that any statements regarding the potential application of the studied network measures are speculative and have clearly convey this message throughout the revised manuscript.

e. The discussion section on "Bilateral propagation of focal seizures reflects imbalance in global integration and segregation in the connectivity brain network" is very unclear to me. The authors

should discuss their findings in context of existing mechanisms of FBTCS like thalamic—basal ganglia circuits.

We appreciate the reviewer's feedback regarding the clarity of this section and have rewritten this section to include more detail and references to past literature. While we agree with the reviewer regarding the importance of the thalamic-basal ganglia circuits to the emergent dynamics of focal to bilateral tonic-clonic seizures, our analytical frameworks and dataset do not allow for such region-specific (i.e., thalamic and basal ganglia) information to be evaluated. To address this question, future studies will likely need to evaluate seizure dynamics from either 1) intracranial EEG recordings (as opposed to ECoG recordings) where multiple subcortical regions (including the thalamus and basal ganglia) are simultaneously collected; or 2) high resolution fMRI (7T) where activation patterns associated with subcortical regions can be inferred.

f. The discriminatory network level metrics cannot be considered as biomarkers unless they are cross validated.

Thank you for this thoughtful comment. We intended to suggest that the network level metrics examined in our study are potential candidates for biomarkers, either in their own rights or in combination with other neural/computational features. We agree with the reviewer regarding the need for cross-validation as a critical step towards ensuring the use of such metrics as biomarkers for seizure types and have clarified our text throughout the revised manuscript to reflect this point.

18. Conclusions: Authors are suggested to temper down their claims specific to the data shown in this manuscript. The speculative aspects should be stated clearly as plausible directions.

We have gone through the manuscript and ensure that any speculative aspects and interpretation of the observed findings are clearly stated as plausible directions as the reviewer suggested.

19. Abstract
The three points in abstract needs to be tempered:
"…that are predictive of the extent…"— associate with FBTCS or focal only seizures.
"…could aid successful intervention strategies…" — speculative. The results do not support this statement.
"…focal seizure subtypes…" again please be specific. You are only comparing FBTCS with focal only seizure not all focal seizure subtypes.

Following the reviewer's suggestions, we have now rewritten these sentences such that the abstract now reads:

"… Our results characterize network features that quantify the underlying functional dynamics associated with the observed heterogeneity of seizure propagation across these two types of focal seizures. Decoding these network features demonstrate that bilateral propagation of seizure activity is an outcome of the imbalance of global integration and segregation in the brain prior to seizure onset.

We show that there exist intrinsic network signatures preceding seizure onset that are associated with the extent to which an impending seizure will propagate throughout the brain (i.e., staying within one hemisphere vs. spreading transcallosally). Additionally, these features characterize an increase in segregation and a decrease in excitability within the brain network (i.e., high modularity and low spectral radius). Importantly, seizure-type specific differences in these

features emerge several minutes prior to seizure onset, suggesting the potential utility of such measures in intervention strategies. Finally, our results reveal network characteristics *after* the onset that are unique to the propagation mechanisms of two most common focal seizure subtypes, indicative of distinct reconfiguration processes that may assist termination of each seizure type. Together, our findings provide insights into the relationship between the temporal evolution of seizure activity and the underlying functional connectivity dynamics. These results offer exciting avenues where graph theoretical measures could potentially guide personalized clinical interventions for epilepsy and other neurological disorders in which extensive heterogeneity is observed across subtypes as well as across and within individual patients."
