# Supplementary material for: Brain network dynamics codify heterogeneity in seizure evolution
Source: Brain Commun. 2022 Sep 16;4(5):fcac234. doi: 10.1093/braincomms/fcac234 (PMC9527667; doi:10.1093/braincomms/fcac234)
Supplement: fcac234_Supplementary_Data [file Response_to_reviewers_comments_revision 1.pdf › Response_to_reviewers_comments_revision 1.pdf]

Comments from Editor:
Fig3a-b: please report in the figure legend the test used.

We have now included in the legend information regarding the statistical test used for Figure 3a-b by writing: "Statistical comparisons of network measures as a function of seizure types were computed through a bootstrapping procedure where the underlying data distribution of each network measure was resampled at the level of individual seizures to established 95% confidence intervals (CIs). For (A) and (B), significance of changes in the averages of CC and PL across time windows were evaluated by paired *t*-tests (two-tailed). For (C) and (D), error bars indicate 95% CIs across individual seizures in each condition and solid bars show resampled $P < 0.05$."

Comments from Reviewer 2:
The authors have included network density as a new feature in this revision. However, the computation of network density is not clear.
The density of a network is the number of edges present w.r.t. the total number of edges. Authors compute density as the sum of the upper triangle matrix w.r.t. the total number of edges. Can they justify this approach or provide a reference where this computation was applied?

Thank you for this comment. We have now included additional text to clarify that we have calculated weighted network density and a reference to past literature where this computation was applied by writing: "Network density describes the extent of connectedness in a network. Seizures have been shown to alter the synchronization, and therefore, overall connectivity and density of the functional network. In a binary network, where the elements of connectivity matrix are either 1, for a link between the nodes, or 0, for absence of a link, the density is calculated as the ratio of actual and possible connections. A density of 1 describes a fully connected network and a density of 0 describes no connectivity. In a weighted network, if many weak connections are binarized, the density of the network can become misleadingly high. Therefore, we calculated weighted network density as the ratio of total edge weights and number of possible edges. For a functional connectivity matrix A representing an undirected network with N nodes, the total number of possible edges is N(N-1)/2 and the total edge weight is the sum of the elements in the upper triangle of the connectivity matrix. Similar methods have previously been used to evaluate and successfully capture the degree of connectedness in a range of real-world brain networks across species (i.e., humans, macaques, cats, and C. elegans)[33]."
