# Supplementary material for: Brain network dynamics codify heterogeneity in seizure evolution
Source: Brain Commun. 2022 Sep 16;4(5):fcac234. doi: 10.1093/braincomms/fcac234 (PMC9527667; doi:10.1093/braincomms/fcac234)
Supplement: fcac234_Supplementary_Data [file Response_to_reviewers_comments_revision_2.pdf › Response_to_reviewers_comments_revision_2.pdf]

Dear Editor,

Thank you for provisionally accepting our manuscript entitled “Brain network dynamics codify heterogeneity in seizure evolution”. We are grateful for the time and effort that you and the reviewers have dedicated to providing valuable and insightful feedback on our manuscript. We have performed the suggested statistical analysis and incorporated the results in our revision. The additional text changes in the Material and methods, Results, and Fig. 3 caption are in blue.

Sincerely,

Dr. Kanika Bansal, corresponding author
Senior Fellow
Human Research and Engineering Directorate
US DEVCOM Army Research Laboratory &
Department of Biomedical Engineering
Columbia University, New York, NY
917-969-2941

On behalf of
Dr. Nuttida Rungratsameetaweemana
Dr. Claudia Lainscsek
Dr. Sydney S. Cash
Dr. Javier O. Garcia
Dr. Terrence J. Sejnowski
