# Supplementary material for: Brain network dynamics codify heterogeneity in seizure evolution
Source: Brain Commun. 2022 Sep 16;4(5):fcac234. doi: 10.1093/braincomms/fcac234 (PMC9527667; doi:10.1093/braincomms/fcac234)
Supplement: fcac234_Supplementary_Data [file Revision_1_decision_letter.pdf › Revision_1_decision_letter.pdf]

Dear Dr. Bansal,

Thank you for your recent submission BRAINCOM-2021-255.R1
entitled "Brain network dynamics codify heterogeneity in seizure evolution" to Brain Communications.

Your revised manuscript has been reviewed, and while the reviewers had largely positive comments, there is one remaining clarification that I think you could address in a minor revision of the manuscript. Therefore, I invite you to respond to the reviewer(s)' comments and revise your manuscript. The reviewer(s)' comments are included at the bottom of this message.

In addition to the reviewers' comments, please also address our Scientific Editor's comments added below to comply with our requirements for presentation of scientific data at Brain Communications.

Please format your revised manuscript according to our Instructions to Authors https://academic.oup.com/braincomms/pages/General_Instructions – a summary of key points is attached to help with this.

To revise your manuscript, click the link below or log into https://mc.manuscriptcentral.com/braincom and enter your Author Centre, where you will find your manuscript title listed under "Manuscripts with Decisions." Under "Actions," click on "Create a Revision." Your manuscript number will be appended to denote a new revision.

*** PLEASE NOTE: This is a two-step process. After clicking on the link, you will be directed to a webpage to confirm. ***

https://mc.manuscriptcentral.com/braincom?URL_MASK=71d47eaece674e28af0df8abf1a81b0d

Please upload a revised manuscript with changes tracked using track changes or in coloured text.

When submitting your revised manuscript, you will be asked to respond to the reviewer(s) comments. Please be as specific as possible in your response to all of the comments.

The editorial team at Brain Communications also aims to increase transparency in scientific publishing and to demonstrate the value that peer review brings to the scientific process. Please therefore consider whether you are willing to have reviewer comments on your paper and your response published as supplementary data with the paper if it is accepted. This is entirely optional and will only be done if both the authors and reviewers agree. When submitting your revision you will have the opportunity to update the answer you entered previously.

IMPORTANT: Your original files are available to you when you upload your revised manuscript. Please delete any redundant files before completing the submission.

Because we are trying to facilitate timely publication of manuscripts submitted to Brain Communications, we would appreciate your revised manuscript as soon as possible. If it is not possible for you to resubmit within 3 months, the option to submit a revision will expire, and we would then consider your revised paper as a new submission. However, if you need a little more time, please get in touch and we can extend this deadline for you.

Once again, thank you for submitting your manuscript to Brain Communications and I look forward to receiving your revision.

Best wishes

Prof. Richard Chin
Associate Editor, Brain Communications

Professor Tara Spires-Jones
Founding Editor, Brain Communications

Reviewer(s)' Comments to Author:

Reviewer: 2 - Sinha, Nishant

Comments to the Author

The authors have made substantial changes in their manuscript, corrected their methodological approach, and rectified the over-interpretation of results. I am pleased with the authors' efforts to improve this manuscript.

I have only one remaining question:
The authors have included network density as a new feature in this revision. However, the computation of network density is not clear.
The density of a network is the number of edges present w.r.t. the total number of edges. Authors compute density as the sum of the upper triangle matrix w.r.t. the total number of edges. Can they justify this approach or provide a reference where this computation was applied?

Reviewer: 1

Comments to the Author
I thank the authors for very clear and thorough responses to my concerns, which I feel have bee sufficiently addressed. I greatly appreciated the authors' clarity and attention to detail.

Comments from the Scientific Editor:

- Fig3a-b: please report in the figure legend the test used.
